# Hexafluoroisopropanol Solvent Effects on Enantioselectivity of Dirhodium Tetracarboxylate-Catalyzed Cyclopropanation

**DOI:** 10.1021/jacs.5c03007

**Published:** 2025-04-16

**Authors:** Turki M. Alturaifi, Kristin Shimabukuro, Jack C. Sharland, Binh Khanh Mai, Evan A. Weingarten, Mithun C. Madhusudhanan, Djamaladdin G. Musaev, Peng Liu, Huw M. L. Davies

**Affiliations:** † Department of Chemistry, University of Pittsburgh, Pittsburgh, Pennsylvania 15260, United States; ‡ Department of Chemistry, Emory University, 1515 Dickey Drive, Atlanta, Georgia 30322, United States; § Cherry L. Emerson Center for Scientific Computation, Emory University, 1521 Dickey Drive, Atlanta, Georgia 30322, United States

## Abstract

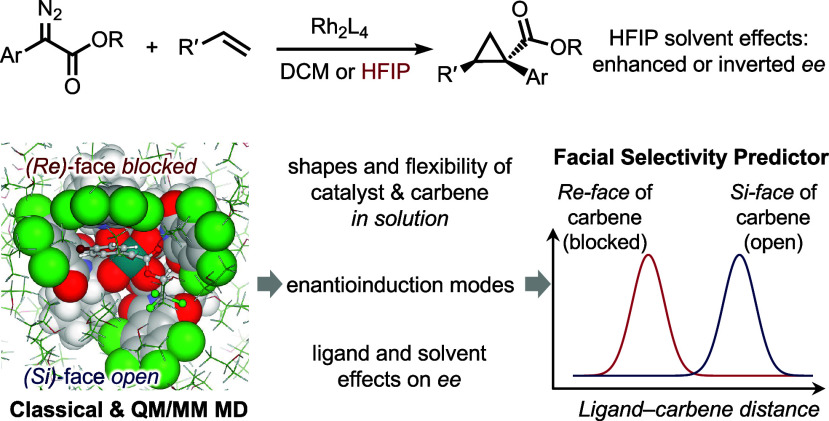

In recent years, additives that modulate both reactivity and selectivity in rhodium-catalyzed reactions of aryldiazoacetates have become increasingly prominent. 1,1,1,3,3,3-Hexafluoroisopropanol (HFIP) has been shown to have a profound effect on rhodium carbene reactivity and selectivity, especially on enabling carbene cyclopropanation in the presence of various nucleophilic poisons. HFIP also has a variable influence on the enantioselectivity of the reactions catalyzed by chiral dirhodium tetracarboxylates, and this study examines the fundamental properties of the rhodium carbene/HFIP system through experimentation, density functional theory (DFT), and molecular dynamics (MD) simulations. These studies revealed that the C_4_-symmetric bowl-shaped catalysts, which have been previously considered to be relatively rigid, experience far greater flexibility in this hydrogen bonding media, resulting in distortion of the bowl-shaped catalysts. These studies explain why even though a majority of the catalysts have a drop in enantioselectivity in HFIP, some catalysts, such as Rh_2_(TCPTAD)_4_, lead to a switch in enantioselectivity, whereas others, such as Rh_2_(NTTL)_4_, lead to a considerably enhanced enantioselectivity.

## Introduction

**Figure 1:**
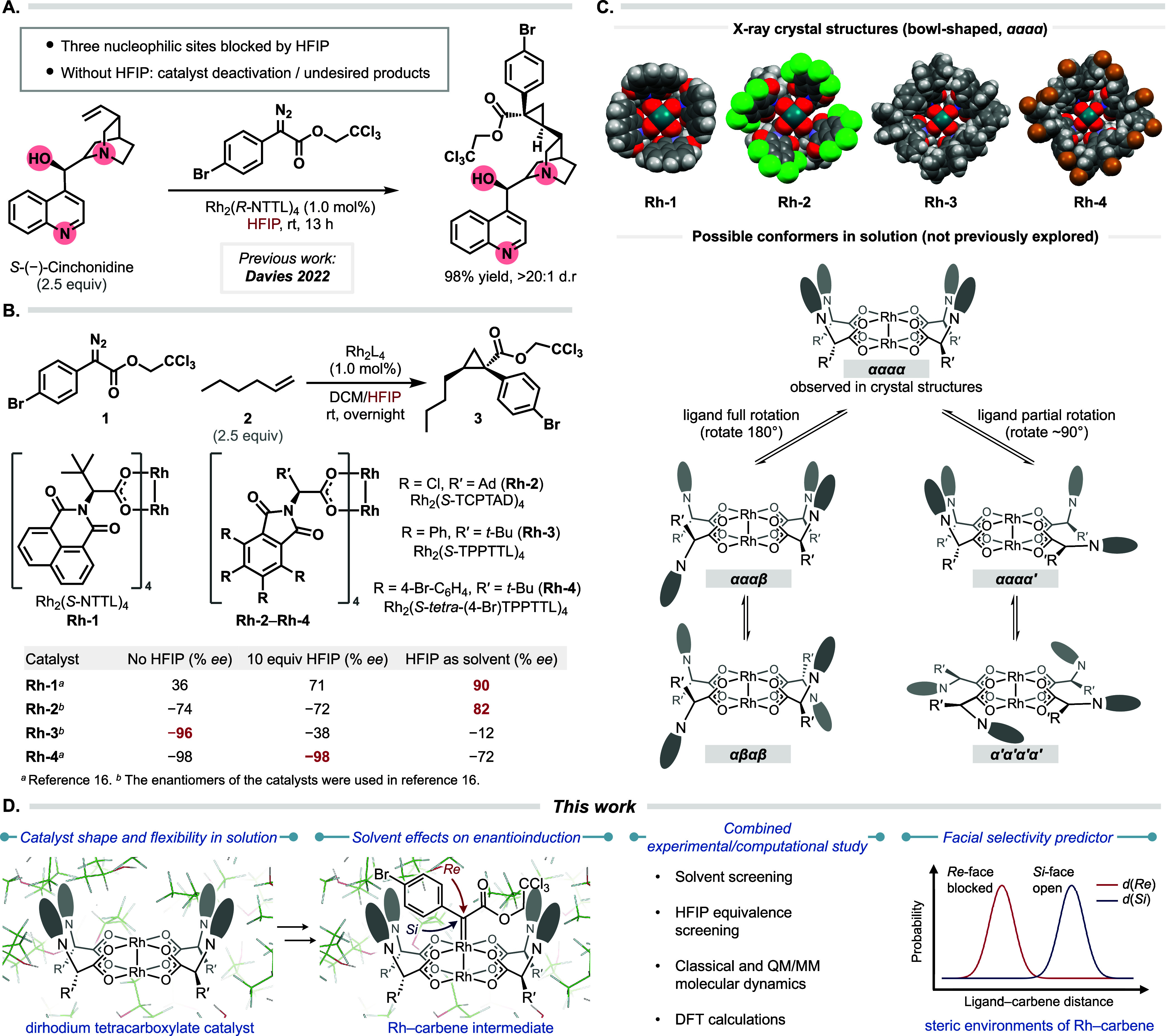
HFIP solvent effects on asymmetric cyclopropanation. (A) Previous study of HFIP in dirhodium tetracarboxylate-catalyzed cyclopropanation;^16^ (B) effect of HFIP equivalents on enantioselectivity; (C) shapes of the dirhodium tetracarboxylate catalysts. X-ray crystal structures: **Rh-1** (CCDC: 749270), **Rh-2** (CCDC: 1535046), **Rh-3** (CCDC: 1855295), and **Rh-4** (CCDC: 2156564); (D) this work: solvent effects on catalyst flexibility and enantioselectivity of dirhodium tetracarboxylate-catalyzed cyclopropanation.

1,1,1,3,3,3-Hexafluoroisopropanol (HFIP) has been shown to have a dramatic influence on a wide range of transition metal-catalyzed reactions.^1−8^ The powerful hydrogen bonding ability of HFIP can promote a variety of catalytic reactions by activating substrates for their subsequent reactions.^1,3−12^ Indeed, the impact of HFIP has been so dramatic and extensive that it has been described as the “magical solvent”.^2^ Occasionally, HFIP has been shown to be effective at coordinating to nucleophilic sites that would otherwise have poisoned transition-metal catalyzed transformations.^13−15^ We have recently shown that HFIP has a major influence on dirhodium tetracarboxylate-catalyzed carbene reactions.^16^ These carbenes undergo a wide variety of synthetically useful reactions with high levels of asymmetric induction, such as cyclopropanation, cyclopropenation, and C–H functionalization, but typically, strongly nucleophilic sites interfere with these reactions by poisoning the catalyst or reacting with the carbene.^17^ The use of HFIP as a solvent enables the reaction to be conducted in the presence of a wide variety of nucleophiles, including heterocycles.^16^ This simple modification of the reaction conditions greatly increases the pharmaceutical relevance of these transformations because a much wider range of functionality can be tolerated. An illustration of this type of transformation is the cyclopropanation between the aryldiazoacetate and (*S*)-cinchonidine (Figure 1A),^16^ formed as a single diastereomer in 98% yield.^16,18,19^

While the initial exploration of the use of HFIP to block nucleophilic sites was largely successful, HFIP did have an unfortunate and unpredictable effect on the asymmetric induction exhibited by many of the chiral dirhodium tetracarboxylate catalysts.^13^ Some of the earlier examples using very small amounts of HFIP (0.1 equiv) increased enantioselectivity.^20^ However, in the reactions using 10 equiv of HFIP or HFIP as solvent, conditions required to block nucleophilic sites, the enantioselectivity exhibited by the majority of the chiral dirhodium tetracarboxylate catalysts is greatly diminished.^16^ This is readily seen in the cyclopropanation of 1-hexene (**2**) with the aryldiazoacetate **1** to form cyclopropane **3** (Figure 1B). Interestingly, HFIP had a positive effect on the reaction with Rh_2_(NTTL)_4_ (**Rh-1**). Even though Rh_2_(NTTL)_4_ has been shown to be an effective chiral catalyst in a variety of group transfer reactions, it has not been commonly used for reactions with aryldiazoacetates because it gives low levels of asymmetric induction. When Rh_2_(NTTL)_4_-catalyzed cyclopropanation was conducted in DCM the enantioselectivity was low (36% ee) but when HFIP was used as solvent, **3** was formed in 90% ee. The most dramatic effect was seen with Rh_2_(TCPTAD)_4_^16^ (**Rh-2**) because HFIP caused a reversal of asymmetric induction with this catalyst. When DCM was used as solvent, the opposite enantiomer of **3** was formed in 74% ee, whereas when the reaction was conducted with HFIP as solvent, cyclopropane **3** was formed in 82% ee. One of the most extensively used catalysts, Rh_2_(TPPTTL)_4_ (**Rh-3**),^21−24^ is normally capable of high levels of asymmetric induction, but when the reaction is conducted with 10 equiv HFIP and with HFIP as solvent, the enantioselectivity drops to 38% ee and 12% ee, respectively.^16^ Finally, one of the best catalysts at retaining high levels of asymmetric induction is Rh_2_(*tetra-*(4*-*Br)TPPTTL)_4_ (**Rh-4**).^16,25^ In the presence of 10 equiv of HFIP, the enantioselectivity was still 98% ee but it decreased to 72% ee when HFIP was used as solvent.

To fully exploit the influence of HFIP on carbene reactions and be able to rationally design new catalysts that have robust asymmetric induction in the presence or absence of HFIP, it will be necessary to understand what causes HFIP to have such varied effects on the asymmetric induction exhibited by the dirhodium catalysts. The pronounced effects of HFIP on enantioselectivity imply that the geometry of the Rh-carbene intermediates, and potentially the mode of the enantioinduction, are affected by both the tetracarboxylate ligands and the solvent. Previous X-ray and NMR studies have demonstrated different degrees of flexibility of the paddlewheel dirhodium catalysts. For example, X-ray studies showed that in Rh_2_(*S*-DOSP)_4_ and Rh_2_(*S*-PTPA)_4_ the arylsulfonyl and phthalimide groups are oriented to different faces of the paddlewheel complex, whereas the more commonly used catalysts for aryldiazoacetates [(**Rh-1**)–(**Rh-4**)] adopt the bowl-shaped *αααα* conformation that orient the four imide groups to the same face (Figure 1C).^26,27^ This bowl-shaped conformation was also observed in the X-ray structure of a Rh_2_(*S*-PTTL)_4_-carbene intermediate.^28^ Previous NMR studies by Charette et al. suggested equilibration between the bowl-shaped conformation and other conformers with one or two of the phthaloyl groups rotated by 180° toward the opposite face of the paddlewheel complex (e.g., *αααβ* and *αβαβ*, Figure 1C).^26^ In addition, structures involving partial carboxylate ligand rotation by ∼90° about the C_carboxylate_–C_α_ bond (e.g., the *αααα′* and *α′α′α′α′* conformers, Figure 1C) have also been observed in X-ray structures of Rh_2_(*S*-PTTL)_4_.^29^

Several previous computational studies investigated the geometries of chiral dirhodium tetracarboxylate complexes,^23,30−32^ and the mechanisms of enantioinduction in cyclopropanation and C–H functionalization.^33−37^ However, most of the previous studies are limited to models without the explicit solvent molecules. While it has been hypothesized that paddlewheel catalysts might exhibit conformational change in solution,^26,29,38−40^ the actual geometries of the dirhodium catalysts and the Rh-carbene intermediates *in solution* remain unclear. Additionally, it remains unknown how the solvent affects the shape and flexibility of these species,^37^ and whether these properties affect the enantioinduction. Computational studies have shown HFIP’s ability to stabilize both positive and negative charges, as well as promote reactivity or selectivity.^4,41,42^ However, the influence of HFIP on the structural flexibility of catalysts and its effects on enantioinduction mechanisms remains less explored.

Here, we present a combined experimental and computational study to understand the origin of HFIP solvent effects on the enantioinduction of the Rh-catalyzed cyclopropanation reactions. Experimental studies were conducted to confirm the general trends in asymmetric induction in a series of cyclopropanation reactions for two of the most distinctive systems, Rh_2_(NTTL)_4_ and Rh_2_(TCPTAD)_4_. To properly account for solvent effects on enantioinduction, we carried out a multiscale computational approach combining classical molecular dynamics (MD), hybrid quantum mechanics/molecular mechanics (QM/MM) MD, and density functional theory (DFT) methods. We show that these catalysts have different degrees of flexibility influenced by both the nature of ligands and the solvent environment. The MD simulations revealed that the steric environments of the Rh-carbene intermediates correlate with the experimentally observed enantioselectivity trends in the DCM and HFIP solvents.

## Results and Discussion

### Experimental Studies of HFIP Solvent Effects on Enantioinduction

**Figure 2:**
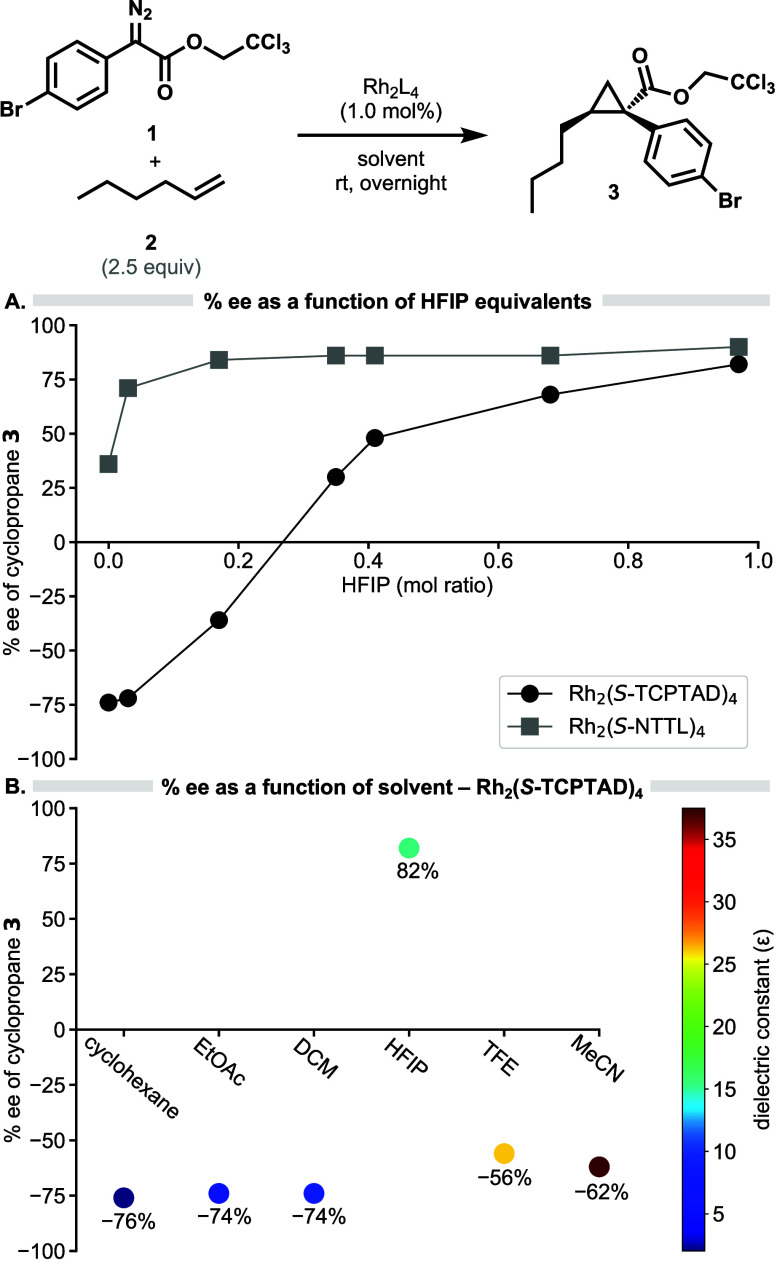
(A) Equivalent screens; (B) solvent screens.

Considering the dramatic influence of HFIP on the performance of the catalysts, further cyclopropanation reactions were conducted to confirm that the variation on the enantioselectivity is a general and broadly occurring influence. The standard cyclopropanation reaction of the aryldiazoacetate **1** with 1-hexene **2** was used to determine what amount of HFIP was necessary to cause the switch in the asymmetric induction in Rh_2_(TCPTAD)_4_-catalyzed reactions. The results summarized in Figure 2A (see also Figure 1B) reveal that a large amount of HFIP is necessary to achieve enantioinversion.

With 10 equiv of HFIP compared to the amount of the aryldiazoacetate (1000 equiv compared to the catalyst), the enantioselectivity dropped very slightly from −74% ee to −72% ee. The switch in the enantioselectivity occurred when between 80 and 100 equiv of HFIP was used, and the maximum value (82% ee) was obtained when HFIP was used as solvent. We consider this behavior to be a possible indication that the HFIP influence may require interactions of the complex with multiple HFIP molecules. HFIP is capable of strong hydrogen bonding and is much more polar than DCM. Previously, it had been shown that one of the first generation chiral dirhodium catalysts, Rh_2_(*S*-DOSP)_4_, was sensitive to the polarity of the solvent and provided higher levels of asymmetric induction when nonpolar hydrocarbons were used as solvent.^43^ The ligands in Rh_2_(*S*-DOSP)_4_, however, are considered to be quite mobile, unlike the bowl-shaped catalysts used in this study.^44^ To test whether solvent polarity has a significant influence, the test reaction with Rh_2_(*S-*TCPTAD)_4_ was conducted in a range of solvents, as shown in Figure 2B. Most of the solvents resulted in very similar levels of enantioselectivity and there is no evidence of a trend dependent on the dielectric constant of the solvent. HFIP has quite a dramatic effect, resulting in a switch in enantioselectivity, which is most likely due to the strong hydrogen bonding influence of the solvent.^45^

**Figure 3:**
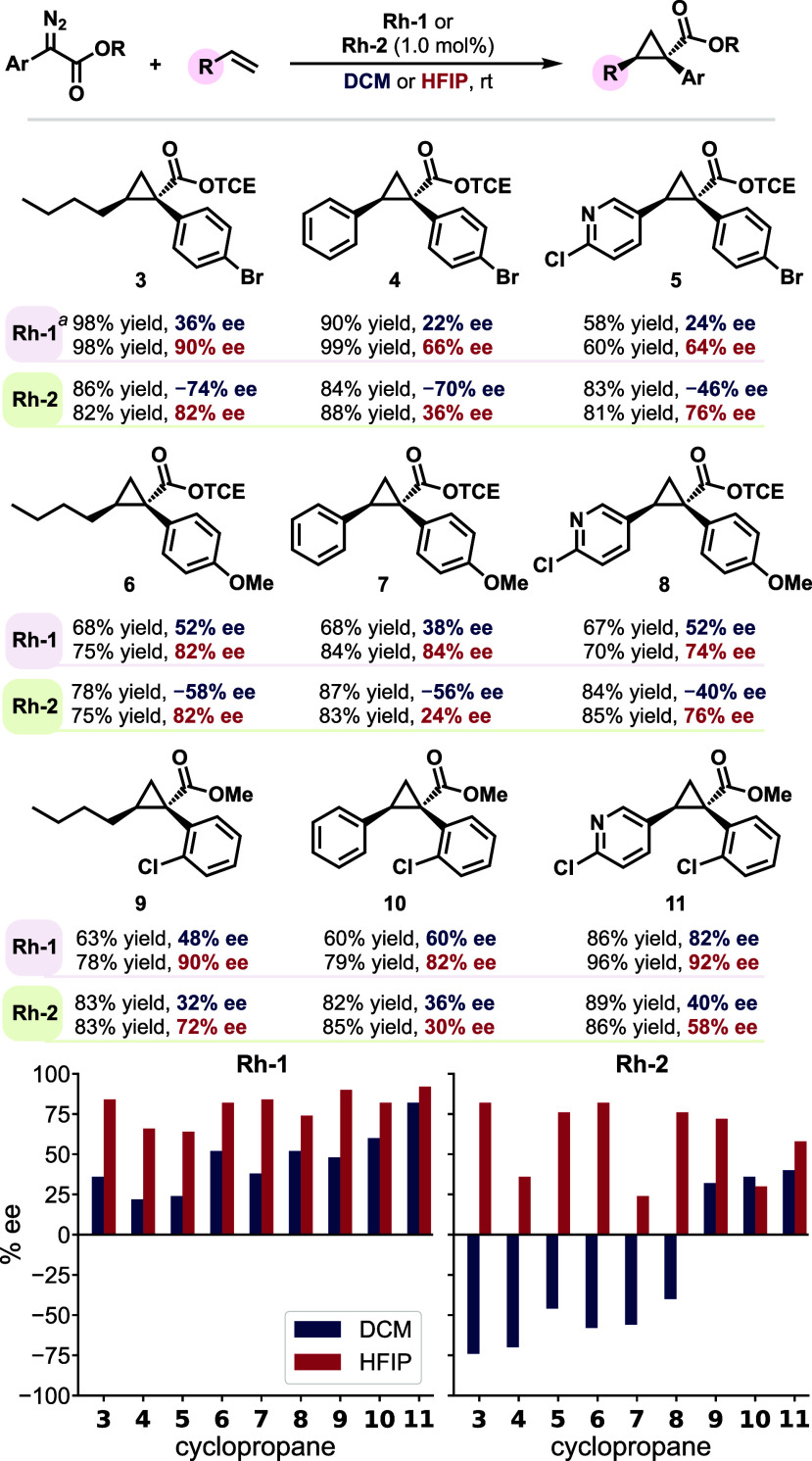
Substrate scope. **Rh-1** = Rh_2_(*S*-NTTL)_4_, **Rh-2** = Rh_2_(*S*-TCPTAD)_4_. *^a^*Results are taken from ref (16). TCE: trichloroethyl.

In the original evaluation of the influence of HFIP, most of the studies were conducted using a single test reaction, the cyclopropanation of 1-hexene with aryldiazoacetate **1**. Therefore, we wished to determine whether the influence of HFIP on enantioselectivity was consistently observed with a range of substrates. Cyclopropanation studies conducted on three representative carbene precursors and three representative alkenes were carried out using Rh_2_(*S*-NTTL)_4_ and Rh_2_(*S*-TCPTAD)_4_ (Figure 3). With Rh_2_(*S*-NTTL)_4_, all the reactions considerably improved in enantioselectivity when HFIP was used instead of DCM as solvent. In the presence of HFIP as solvent, all products were obtained in greater than 73% ee except for **4** and **5,** which were obtained in 66% ee and 64% ee, respectively. In contrast, in the absence of HFIP, the products were obtained in 22–60% ee, except for **11**, which was obtained in 82% ee, still inferior to the asymmetric induction in the presence of HFIP (92% ee). Overall, the cyclopropanation of the *ortho*-substituted aryldiazoacetate in the presence of HFIP gave the highest levels of asymmetric induction (82–92% ee, compounds **9**–**11**). The Rh_2_(*S*-TCPTAD)_4_-catalyzed reactions resulted in a more drastic change in enantioselectivity. All the reactions conducted with *para*-substituted aryldiazoacetates resulted in a switch in enantioselectivity on changing the solvent from DCM to HFIP (compounds **3**–**8**). In contrast, the *ortho*-substituted aryldiazoacetate gave low enantioselectivity in DCM and similar or improved enantioselectivity in HFIP (compounds **9**–**11**). The different behavior of the *ortho*-substituted aryldiazoacetate is consistent with previous studies, which showed that *ortho*-substituted aryldiazoacetates have a dramatically different stereoselectivity profile compared to the *para*-substituted aryldiazoacetates, requiring a different chiral catalyst and the use of additives to achieve high asymmetric induction.^21^ Finally, the other catalysts, Rh_2_(*S*-TPPTTL)_4_ and Rh_2_(*S*-*tetra*-(4-Br)TPPTTL)_4_, which performed extremely well in DCM have drastically lower asymmetric induction in the presence of HFIP. With Rh_2_(*S*-TPPTTL)_4_, the asymmetric induction drops considerably with just 10 equiv of HFIP. With Rh_2_(*S*-*tetra*-(4-Br)TPPTTL)_4_, little change in the asymmetric induction was observed with 10 equiv of HFIP but it did decrease considerably when HFIP was used as solvent.

To summarize, the above presented studies revealed that upon use of Rh_2_(*S-*TCPTAD)_4_ the enantioselectivity switch in the cyclopropanation occurs consistently with the *para*-substituted but not *ortho*-substituted aryldiazoacetates. Enhancement in enantioselectivity by the Rh_2_(*S-*NTTL)_4_ catalyst is observed with all tested aryldiazoacetates when the reactions are conducted in HFIP versus DCM. The influence of HFIP is much more pronounced when high concentrations of HFIP are used. The effect is not due to the change of dielectric constant of the solvent and is more likely due to the hydrogen bonding capabilities of the solvent. HFIP is ideally suited because it can form strong hydrogen bonds with hydrogen-accepting groups but is not sufficiently nucleophilic to react with the rhodium carbene.

### Molecular Dynamics (MD) Simulations of Dirhodium Catalysts and Rh-Carbene Complexes in HFIP and DCM Solvents

**Figure 4:**
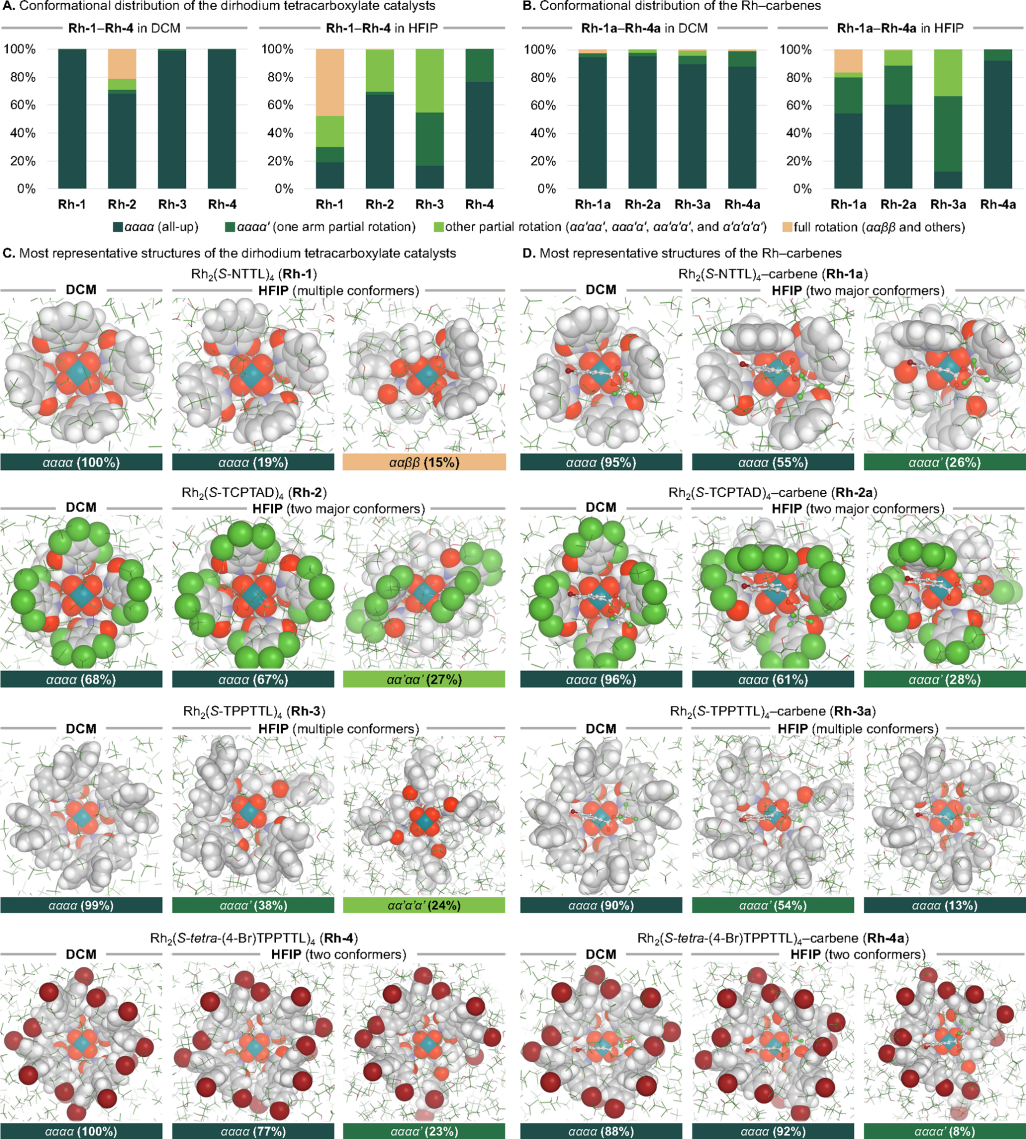
Solvent effects on the shapes of the dirhodium tetracarboxylate catalysts (**Rh-1**–**Rh-4**) and corresponding Rh–carbene complexes (**Rh-1a**–**Rh-4a**). The conformational distributions and snapshots were obtained from three to six replicas of 1,000 ns classical MD simulations.

To elucidate the puzzling effects of HFIP on enantioinduction demonstrated above, we conducted comprehensive multiscale computational studies using (i) classical MD simulations with the general Amber force field (gaff2),^46^ customized force field parameters for Rh generated using the MCPB.py module,^47^ and previously reported HFIP parameters;^48^ (ii) QM/MM MD simulations where the dirhodium tetracarboxylate catalyst and carbene (up to 204 atoms) were treated using the semiempirical GFN1-xTB^49^ method and solvent molecules were modeled using molecular mechanics; and (iii) DFT modeling incorporating several explicit solvent molecules into the calculations performed at the B3LYP-D3(BJ)/[6-311+G(d,p)–SDD(Rh)]/SMD(DCM or HFIP)//B3LYP-D3(BJ)/[6-31G(d)–SDD(Rh)] level of theory (see Supporting Information for more details of each utilized approach). Three to six replicas of independent 1,000 ns classical MD simulations were performed on the four catalysts (**Rh-1**–**Rh-4**) and their respective Rh–carbene complexes (**Rh-1a**–**Rh-4a**) in DCM and HFIP. The MD simulations revealed conformational changes of the dirhodium catalysts in both solvents (Figure 4), which deviate from the C_4_-symmetric geometry observed in their X-ray crystal structures (Figure 1C).

As it was hypothesized, these conformational changes are more pronounced in HFIP than in DCM. For example, although all four dirhodium catalysts favored the bowl-shaped *αααα* conformation in DCM with relatively small changes of the dihedral angles about the C_carboxylate_–C_α_ bond (θ) from those in the crystal structures (Figure S1), they are considerably deformed in HFIP (Figure 4A). Among the four dirhodium catalysts, the *S*-NTTL-supported **Rh-1** is the most flexible in HFIP: the bowl-shaped structure of **Rh-1** was only observed in 19% of the MD frames (see the most populated conformer, *αααα,* in Figure 4C), whereas in the rest of the sampled structures, at least one *N*-1,8-naphthaloyl group undergoes partial or full rotation to the opposite face of the paddlewheel complex (see the second most populated conformer, *ααββ*, in Figure 4C and other representative structures of the same complex in Figure S7). On the other hand, other catalysts (**Rh-2**–**Rh-4**) largely maintained the bowl shape or only exhibited *partial* carboxylate ligand rotation,^17,27,50^ in HFIP (Figure 4C). The different catalyst flexibilities suggest that the intramolecular noncovalent interactions (NCIs) between adjacent carboxylate ligand arms (e.g., T-shaped π/π interactions and other dispersion interactions) compete with the intermolecular NCIs with solvent molecules (Figure S14). Hydrogen bond interactions with HFIP may disrupt the bowl shape of the catalyst when the intramolecular NCIs are not strong enough, such as in **Rh-1**.

Introduction of the carbene into the reactive pocket of catalyst results in additional conformational changes in all studied catalysts. In DCM, these changes are not pronounced, except for **Rh-2a**. In all systems, the presence of carbene destabilizes the catalyst’s most populated bowl-shaped *αααα* conformation, while in **Rh-2a**, it significantly stabilizes the *αααα* conformation. In contrast, these conformational changes are larger in HFIP. In all systems, the presence of carbene stabilizes the bowl-shaped *αααα* conformation; again, except for **Rh-2a** (Figure 4B). The stabilization of the bowl-shaped *αααα* conformers is the result of the π-π interactions between the 4-Br-Ph group of the carbene and aryl substituents of the carboxylate ligands. Thus, the nature of the carboxylate ligands and solvent significantly impacts shapes and flexibilities of the Rh–carbenes. **Rh-1a**, **Rh-2a**, and **Rh-3a** are all much more flexible in HFIP than in DCM, whereas the most rigid carbene complex, **Rh-4a**, largely maintained the bowl-shaped *αααα* conformation in both solvents (Figure 4B).

Although **Rh-1a** and **Rh-2a** preferred the bowl-shaped *αααα* conformation in both DCM and HFIP, the geometries of these complexes are different in different solvents. For **Rh-1a** and **Rh-2a** in HFIP, one of the carboxylate ligand arms close to the TCE group is distorted away from the carbene. This partial ligand arm rotation creates space to allow another ligand arm to form shorter π-π interactions with the 4-Br-Ph group on the carbene. In both **Rh-1a** and **Rh-2a**, this π-π interaction was only observed at the (*Re*)-face of the carbene, indicating a unique mode of enantioinduction that favors the alkene substrate addition to the less hindered (*Si*)-face of the carbene. The *S*-TPPTTL-supported **Rh-3a** is the most flexible Rh–carbene in HFIP, in which multiple conformers with ligand arm rotations were observed. This observation is consistent with the low level of enantioinduction with **Rh-3** in HFIP (−12% *ee*).

Taken together, the molecular dynamics simulations indicated that the solvent and the carboxylate ligands both significantly affect the shapes and flexibilities of the dirhodium catalysts and the Rh–carbene complexes. Because the shapes of the Rh–carbenes are different from those of the dirhodium catalysts, the catalyst enantioinduction model should be based on the steric environments of the Rh–carbenes (**Rh-1a**–**Rh-4a**), whereas the shapes and flexibilities of the dirhodium catalysts themselves would not directly govern the enantioselectivity.

### Enantioinduction Models

**Scheme 1:**
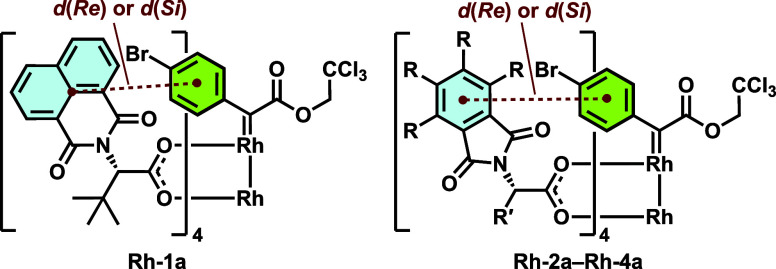
Defining the Ligand–Carbene Distances *d*(*Re*) and *d*(*Si*) as a Distance Metric to Describe the Steric Environments at the Prochiral Faces of the Rh–carbene *d*(*Re*): distance to the closest carboxylate ligand on the (*Re*) face of the carbene; *d*(*Si*): distance to the closest carboxylate ligand on the (*Si*) face of the carbene.

We hypothesize that the differences in shapes and flexibilities of the Rh–carbenes (**Rh-1a**–**Rh-4a**) in solution may directly affect the preferred prochiral π-face of the carbene that reacts with the alkene substrate and thus could predict both ligand and solvent effects on enantioselectivity. To quantify the differences of the steric environment between the two prochiral π-faces, we explored a number of structural features as potential metrics for enantioselectivity predictions. These include dihedral angles about the C_carboxylate_–C_α_ bond, percent buried volumes, and distances between the carboxylate ligand arms to the carbene carbon and to the centroid of the benzene ring of the 4-Br-Ph group on the carbene (see Figures S4, S6, and S11). These analyses were performed with both classical MD and QM/MM MD simulations to explore whether the less time-consuming classical MD simulations could provide predictions consistent with QM/MM MD results (Figure S8). After careful examinations of the computed descriptors, we identified the distance between the centroids of the aryl group on the ligand and the benzene ring of the carbene donor group (Scheme 1) as a simple descriptor to describe the magnitude of steric encumbrances at the two prochiral π-faces of the carbene [*d(Re*) and *d*(*Si*) for the distances to the carboxylate ligand on the (*Re*)- and (*Si*)-faces of the carbene, respectively]. We note that because the substituent on the monosubstituted alkene substrate is placed *syn* to aryl group on the carbene in the cyclopropanation transition state^51^ (Figure S3), the steric environments around this aryl group are the most relevant for enantiodiscrimination.

**Figure 5:**
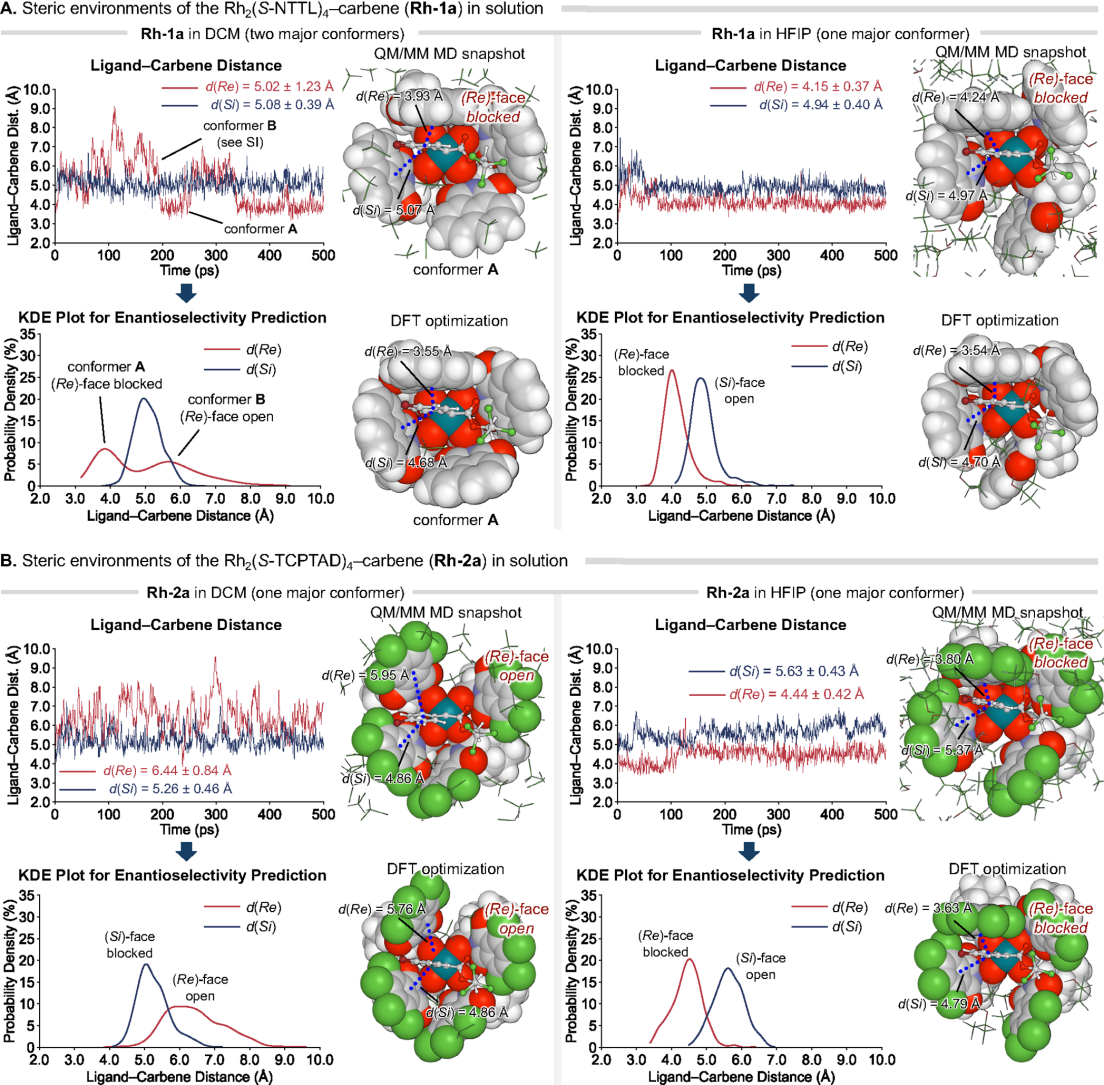
Ligand–carbene distance-based metrics for enantioselectivity prediction. Representative QM/MM MD snapshots and DFT-optimized structures shown to demonstrate preferred conformations of the Rh–carbene complexes and the steric environments at the (*Re*)- and (*Si*)-faces of the carbene. A longer *d*(*Re*) or *d*(*Si*) indicates the less hindered prochiral face of the carbene that preferentially undergoes reaction with the alkene substrate. See Figure S17 for the MD snapshot and DFT-optimized structure of **Rh-1a** conformer B in DCM.

As shown in Figure 5A (right), in the most populated structures of **Rh-1a** from both QM/MM MD simulations and DFT geometry optimization, *d(Re*) is shorter than *d(Si*), indicating that the (*Re*)-face of the carbene is relatively more occupied by the carboxylate ligands. This ligand-induced preference for the alkene addition to the (*Si*) face of the alkene is consistent with the observed major enantiomer **3** in the **Rh-1**-catalyzed cyclopropanation in both DCM and HFIP (Figure 1B). Monitoring the ligand–carbene distances, *d(Re*) and *d*(*Si*), along the QM/MM MD trajectories provided additional insights into the importance of dynamical features on enantiocontrol. In DCM, **Rh-1a** has two distinct conformers that shed light on the enantioinduction: conformer **A**, which represents 52.2% of the structures along the MD trajectories, has the carbene (*Re*)-face blocked by π-π interactions with the carboxylate ligand, whereas conformer **B** that represents 47.8% of structures has the (*Re*)-face more open than the (*Si*)-face (see Figure S17 for the MD snapshot and DFT-optimized structure of conformer **B**). This conformational flexibility is consistent with the low enantioselectivity (36% *ee*) observed experimentally. By contrast, in HFIP, the same complex maintains a consistent conformation in which the (*Re*)-face is blocked by the ligand, as evidenced by a shorter *d*(*Re*) than *d*(*Si*) in most of the structures along the MD trajectories. Overall, the entire catalyst is more distorted from the C4 symmetry in HFIP—one of the carboxylate arms near the (*Si*) face of the carbene almost completely rotates away, which consequentially creates more space to distort the catalyst to form stronger π-π ligand–carbene interactions that block the (*Re*) face of the carbene—a structural feature consistent with the classical MD simulations (Figure 4D). This more distorted geometry led to greater difference between the steric environments at the (*Re*)- and (*Si*)-faces of the carbene, which is consistent with the higher enantioselectivity (90% *ee*) observed in HFIP than in DCM. These HFIP-induced structural features were also observed in the QM/MM MD and DFT-optimized structures of **Rh-2a**, in which the same (*Re*)-face of the carbene is blocked by π–π interactions with the TCPTAD ligand (Figure 5B, right).

We surmised that a kernel density estimation (KDE) plot of *d*(*Re*) and *d*(*Si*) could serve as an effective visualization tool to capture both the shape and flexibility effects of the carboxylate ligands on the enantioinduction. The KDE plots that describe the probability density of ligand–carbene distances *d*(*Re*) and *d*(*Si*) were derived from the QM/MM MD trajectories of **Rh-1a** and **Rh-2a** in both solvents (Figure 5). A high probability density at shorter ligand–carbene distances would indicate the corresponding prochiral face of the carbene is blocked by the ligand. In addition, the plots demonstrate the rigidity of the catalyst–carbene interactions. When multiple peaks are present (e.g., **Rh-1a** in DCM), a lower level of enantiocontrol is expected due to the conformational flexibility of the ligand. On the other hand, a sharp peak at a shorter distance (e.g., **Rh-1a** in HFIP) would indicate more rigid ligand–carbene interactions that could lead to a high level of enantiocontrol.

**Figure 6:**
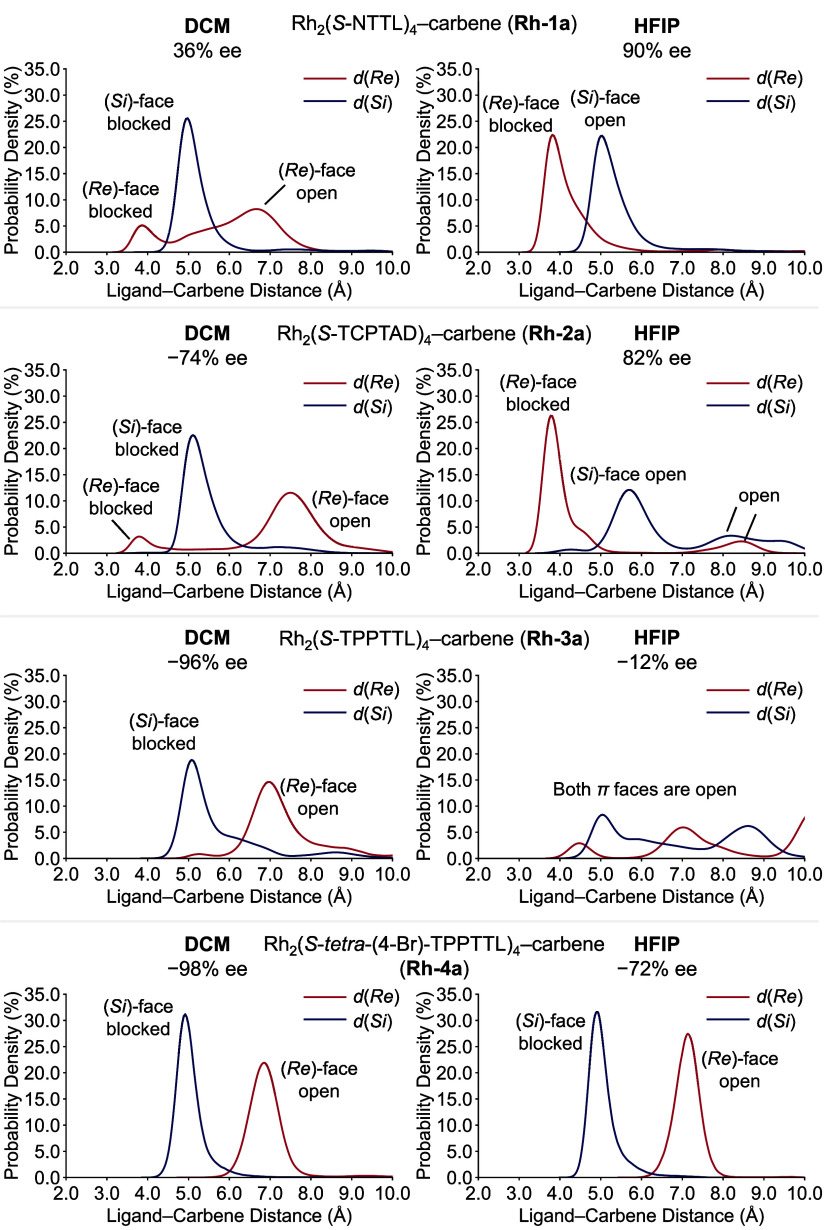
Facial selectivity predictor: a model using steric environments of the Rh–carbene complexes from classical MD simulations to predict ligand and solvent effects on enantioselectivity. Positive experimental *ee* values indicate carbene (*Si*)-face addition is favored; negative experimental *ee* values indicate carbene (*Re*)-face addition is favored.

Next, we used the distance KDE plots to analyze the classical MD trajectories of all four carbene complexes (**Rh-1a**–**Rh-4a**) in both DCM and HFIP (Figure 6). The goal of this work is to determine whether it would be possible to develop a fast facial selectivity predictor that would be generally useful for enantioselectivity predictions of chiral dirhodium tetracarboxylate catalysts. Due to the higher speed of the classical MD simulations, these analyses can be effectively performed on longer time-scale trajectories (1,000 ns, six replicas) and larger catalyst systems (**Rh-3a** and **Rh-4a**) that would be highly resource-intensive for QM/MM MD. The KDE analysis of classical MD trajectories revealed similar patterns to QM/MM MD simulations for **Rh-1a** and **Rh-2a but** also captured additional minor conformers.

While both methods show that **Rh-1a** has a sharp *d*(*Re*) distribution in HFIP (90% *ee*) versus multiple conformers in DCM (36% *ee*), the classical MD reveal additional low-population conformers with shorter *d*(*Re*) distances for **Rh-2a** in both solvents that were not observed in the shorter QM/MM simulations (−74% and 82% *ee* in DCM and HFIP, respectively).

Most notably, **Rh-3a** exhibits a remarkable solvent-dependent behavior—while a sharp *d*(*Si*) distribution at a short distance in DCM leads to high enantioselectivity (−96% *ee*), the switch to HFIP causes complete loss of face selectivity, with both *d*(*Re*) and *d*(*Si*) showing broad distributions. In **Rh-3a**, the diminished facial selectivity is caused by the rotation of the carboxylate ligand arms, which maximizes the hydrogen-bonding interactions with the HFIP solvent. This appears to be more favorable than the catalyst’s own intramolecular interactions that normally maintain the catalyst’s rigid bowl-shaped structure. On the other hand, the bromine substituents in **Rh-4a** make the catalyst much more rigid that shows sharp distance distributions with shorter *d*(*Si*) in both solvents, though with reduced selectivity in HFIP (−98% vs −72% *ee* in DCM and HFIP, respectively). The predicted facial selectivities from our classical MD simulations for all eight catalyst–solvent combinations are consistent with the experimentally observed enantioselectivities.

## Conclusion

The origins of selectivity and reactivity in rhodium carbene transformations in the presence of the coordinating additives can be difficult to rationalize. In this work, the role of HFIP to dramatically change enantioselectivity was evaluated through both experimentation and computational analysis. The experimental results demonstrated the breadth of HFIP’s influence over catalyst enantioselectivity in several chiral systems, which were found to be highly dependent on the concentration of HFIP in solution. The effect of HFIP on both carbene intermediates and chiral catalysts was then evaluated through a combination of molecular dynamics and DFT calculations with explicit solvent molecules. These analyses revealed strong interactions of the catalyst and the carbene intermediates with HFIP, which cause dramatic changes to catalyst geometry and flexibility. MD simulations showed that these classically rigid catalysts become highly flexible and adopt different geometries in the presence of HFIP. These dramatic but transient features of catalyst geometry could be responsible for both enantioinversion and enantioenhancement under this unusual additive paradigm. We have developed a simple metric based on the distances between the carboxylate ligand arms and the carbene from classical MD simulations. This distance metric quantitatively describes both the shape and the flexibility of the Rh–carbene intermediates and was found to correlate with the experimentally observed enantioselectivity.

In the future, we will use this model to elucidate the controlling factors on the dirhodium tetracarboxylate-catalyzed C–H functionalization of complex molecules in the presence of HFIP. We envision this will be a useful technique for predicting the enantioselective performance for newly designed catalysts and carbene intermediates. It is our hope that the facial selectivity predictor developed herein will offer an *in silico* method for evaluation of new catalysts and reagents.

## Supporting Information Available

Experimental procedures, compound characterization, computational details, additional results, and Cartesian coordinates of computed structures (PDF)
