# Supplementary material for: Hexafluoroisopropanol Solvent Effects on Enantioselectivity of Dirhodium Tetracarboxylate-Catalyzed Cyclopropanation
Source: J Am Chem Soc. 2025 Apr 16;147(17):14694–704. doi: 10.1021/jacs.5c03007 (PMC12046558; doi:10.1021/jacs.5c03007)
Supplement: Supplementary file 1 — ja5c03007_si_001.pdf [file ja5c03007_si_001.pdf]

Supplemental Information

Hexafluoroisopropanol Solvent Effects on Enantioselectivity of Dirhodium Tetracarboxylate-Catalyzed Cyclopropanation

Turki M. Alturaifi,[†§] Kristin Shimabukuro,[‡§] Jack C. Sharland,[‡] Binh Khanh Mai,[†] Evan A. Weingarten,[‡]

Mithun C. Madhusudhanan,[†] Djamaladdin G. Musaev,[‡¶*] Peng Liu,[†*] Huw M. L. Davies[‡*]

[†] Department of Chemistry, University of Pittsburgh, Pittsburgh, Pennsylvania 15260, United States. E-mail: pengliu@pitt.edu

[‡] Department of Chemistry, Emory University, 1515 Dickey Drive, Atlanta, GA 30322, United States. E-mail: hmdavie@emory.edu, dmusaev@emory.edu

[¶] Cherry L. Emerson Center for Scientific Computation, Emory University, 1521 Dickey Drive, Atlanta, Georgia 30322, United States.

Table of Contents

1. *General Information* S2
2. *General Procedures* S2
3. *Characterization of reported compounds* S2
4. *HPLC and SFC chromatograms* S7
5. *Computational Methods* S30
6. *Additional Computational Results* S33
7. *References* S55
8. *Cartesian Coordinates* S58

General Considerations

All experiments were carried out in oven-dried glassware under argon atmosphere unless otherwise stated. Flash column chromatography was performed on silica gel. 4Å molecular sieves were activated under vacuum at 300 °C for 4 h. After time elapsed, the flask was cooled to 60 °C under inert nitrogen atmosphere and stored in a 140 °C oven for future use. All solvents were stored over 4Å molecular sieves under argon atmosphere. Unless otherwise noted, all other reagents were obtained from commercial sources (Sigma Aldrich, Fisher, TCI Chemicals, AK Scientific, Combi Blocks, Oakwood Chemicals) and used as received without purification. ^{1}H and ^{13}C NMR spectra were recorded at either 400 MHz (^{13}C at 100 MHz) on Bruker 400 spectrometer or 600 MHz (^{13}C at 151 MHz) on Bruker 600 spectrometer. NMR spectra were run in solutions of deuterated chloroform ($CDCl_3$) with residual chloroform taken as an internal standard (7.26 ppm for ^{1}H, and 77.16 ppm for ^{13}C), and were reported in parts per million (ppm). The abbreviations for multiplicity are as follows: s = singlet, d = doublet, t = triplet, q = quartet, p = pentet, m = multiplet, dd = doublet of doublet, etc. Coupling constants (J values) are obtained from the spectra. Thin layer chromatography (TLC) was performed on aluminum-back silica gel plates with UV light and cerium aluminum molybdate (CAM) or permanganate ($KMnO_4$) stain to visualize. Mass spectra were taken on a Thermo Finnigan LTQ-FTMS spectrometer with APCI, ESI or NSI. IR spectra were collected on a Nicolet iS10 FT-IR spectrometer from Thermo Scientific and reported in unit of cm^{-1}. Enantiomeric excess (% ee) data were obtained on a Varian Prostar chiral HPLC instrument, an Agilent 1100 HPLC, an Agilent 1290 Infinity UHPLC, or a Waters SFC instrument, eluting the purified products using a mixed solution of HPLC-grade 2-propanol (*i*-PrOH) and n-hexane or HPLC-grade MeOH/*i*-PrOH and formic acid as an additive.

General Procedure

An 8- or 16-mL vial containing a stir bar and a few beads of 4Å molecular sieves was flame dried under vacuum. The vial was then evacuated and purged with nitrogen 2 times to establish an inert atmosphere. Then catalyst (1.0 mol%, 0.001 mmol) was added to the vial. The reaction vial bearing catalyst was flushed with nitrogen and olefin (5.0 equiv) was added to the vial via syringe along with 1 mL dry CH_2Cl_2 or HFIP. If 10 equiv HFIP is to be used in the reaction, it is added at this stage to the reaction vial (10 equiv, 0.11ml, 1.0 mmol) via syringe. The nitrogen line attached to the vial was then removed and the reaction mixture was stirred for 5 minutes. Solid diazo compound (1.0 equiv, 0.10 mmol) was weighed out and added to a separate vial (not dried in any way). Diazo compound was dissolved in 1 mL dry CH_2Cl_2 or HFIP and sonicated to ensure all diazo had dissolved before the solution was loaded into a syringe. The syringe was then inserted through the vial septum and the full contents were injected into the vial in one portion over the course of 5 seconds. The reaction was stirred overnight under argon (15 h). After reaction completion, the solution was concentrated via rotovap and resuspended in $CDCl_3$ for analysis by crude ^{1}H NMR to determine product distribution. The crude concentrate was then directly purified by flash column chromatography (0-10% Et_2O/hexanes or EtOAc/hexanes). Fractions containing only product were collected and concentrated via rotovap. Enantioselectivity was determined by chiral HPLC or SFC chromatography.

Characterization of reported compounds

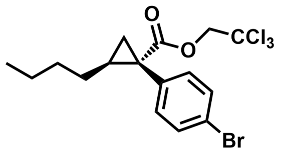

2,2,2-trichloroethyl (1*S*,2*S*)-1-(4-bromophenyl)-2-butylcyclopropane-1-carboxylate (3)

This compound was prepared according to the General Procedure from the reaction between 2,2,2-trichloroethyl 2-(4-bromophenyl)-2-diazoacetate (0.100 mmol, 37.2 mg) and 1-hexene (5.0 equiv, 0.500 mmol, 62.5 μL) in up to 98% yield. Spectra and characterization matched literature reported values.[1]

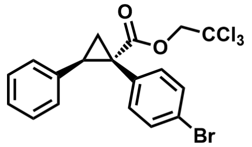

2,2,2-Trichloroethyl (1S,2R)-1-(4-bromophenyl)-2-phenylcyclopropane-1-carboxylate (4)

This compound was prepared according to the General Procedure from the reaction between 2,2,2-trichloroethyl 2-(4-bromophenyl)-2-diazoacetate (0.100 mmol, 37.2 mg) and styrene (5.0 equiv, 0.500 mmol, 57 μL) in up to 99% yield. Spectra and characterization matched literature reported values. [1]

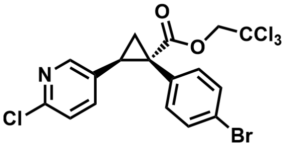

2,2,2-trichloroethyl (1S,2R)-1-(4-bromophenyl)-2-(6-chloropyridin-3-yl)cyclopropane-1-carboxylate (5)

This compound was prepared according to the General Procedure from the reaction between 2,2,2-trichloroethyl 2-(4-bromophenyl)-2-diazoacetate (0.100 mmol, 37.2 mg) and 2-chloro-5-vinylpyridine (5.0 equiv, 0.500 mmol, 69.8 mg) in up to 83% yield. Spectra and characterization matched literature reported values. [2]

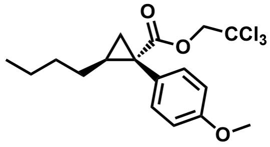

2,2,2-trichloroethyl (1S,2S)-2-butyl-1-(4-methoxyphenyl)cyclopropane-1-carboxylate (6)

This compound was prepared according to the General Procedure from the reaction between 2,2,2-trichloroethyl 2-diazo-2-(4-methoxyphenyl)acetate (0.100 mmol, 32.4 mg) and 1-hexene (5.0 equiv, 0.500 mmol, 62.5 μL) in up to 78% yield. Spectra and characterization matched literature reported values. [1]

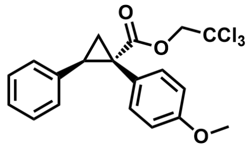

2,2,2-trichloroethyl (1S,2R)-1-(4-methoxyphenyl)-2-phenylcyclopropane-1-carboxylate (7)

This compound was prepared according to the General Procedure from the reaction between 2,2,2-trichloroethyl 2-diazo-2-(4-methoxyphenyl)acetate (0.100 mmol, 32.4 mg) and styrene (5.0 equiv, 0.500 mmol, 57 μL) in up to 87% yield. Spectra and characterization matched literature reported values. [3]

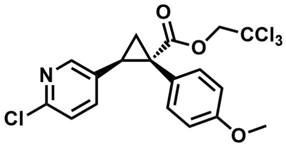

2,2,2-trichloroethyl(1S,2R)-2-(6-chloropyridin-3-yl)-1-(4-methoxyphenyl)cyclopropane-1-carboxylate (8)

This compound was prepared according to the General Procedure from the reaction between 2,2,2-trichloroethyl 2-diazo-2-(4-methoxyphenyl)acetate (0.100 mmol, 32.4 mg) and 2-chloro-5-vinylpyridine (5.0 equiv, 0.500 mmol, 69.8 mg). Product was obtained as a clear colorless oil in up to 85% yield.

^{1}H NMR: (400 MHz, $CDCl_3$) δ 8.02 (d, *J* = 2.5 Hz, 1H), 6.98 (dd, *J* = 9.0, 7.5 Hz, 3H), 6.81 (dd, *J* = 8.3, 2.6 Hz, 1H), 6.75 – 6.67 (m, 2H), 4.84 (d, *J* = 11.9 Hz, 1H), 4.63 (d, *J* = 11.9 Hz, 1H), 3.74 (s, 3H), 3.13 (dd, *J* = 9.4, 7.2 Hz, 1H), 2.31 (dd, *J* = 9.4, 5.3 Hz, 1H), 1.91 (dd, *J* = 7.2, 5.3 Hz, 1H).

^{13}C NMR: (101 MHz, CDCl3) δ 171.84, 159.16, 149.81, 149.76, 137.44, 133.09, 131.30, 124.54, 123.38, 113.79, 95.01, 74.59, 55.27, 36.88, 30.25, 20.75.

IR: 535, 576, 605, 632, 652, 723, 737, 757, 799, 836, 972, 1033, 1057, 1110, 1155, 1209, 1247, 1294, 1348, 1367,1463, 1515, 1560, 1584, 1612, 1733, 2836, 2954.

Chiral SFC: AMY-1, 5 min, 2.5 mL/min, 10%(1:1 mixture of MeOH and *i*-PrOH + 0.2% Formic Acid)/90%CO_2, 55°C column temperature, UV 254 nm; RT: 2.33 min, 3.87 min.

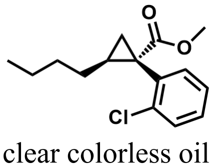

Methyl (1S,2S)-2-butyl-1-(2-chlorophenyl)cyclopropane-1-carboxylate (9)

This compound was prepared according to the General Procedure from the reaction between methyl 2-(2-chlorophenyl)-2-diazoacetate (0.100 mmol, 21.1 mg) and 1-hexene (5.0 equiv, 0.500 mmol, 62.1 μL). After isolation, product was obtained as a clear colorless oil in up to 83% yield.

^{1}H NMR: (600 MHz, $CDCl_3$, 323K) δ 7.39 (dq, *J* = 5.4, 3.1 Hz, 1H), 7.23 (q, *J* = 4.5 Hz, 3H), 3.62 (s, 3H), 2.10 (d, *J* = 13.7 Hz, 1H), 1.74 – 1.59 (m, 2H), 1.45 – 1.19 (m, 6H), 0.84 (t, *J* = 7.2 Hz, 3H)
^{13}C NMR: ^{13}C NMR (151 MHz, $CDCl_3$, 323K) δ 174.26, 137.45, 135.66, 132.22, 129.72, 128.57, 126.63, 52.41, 33.21, 31.56, 28.72, 22.51, 14.09 (Two carbons could not be resolved at 323K).
IR: 698, 731, 751, 778, 963, 1035, 1108, 1173, 1195, 1244, 1267, 1334, 1385, 1434, 1476, 1722, 2860, 2929, 2954
HRMS (APCI): *m/z* calcd for $C_{15}H_{19}ClO_2$ $[M+H]^+$ 266.1074, found 267.1146
Chiral SFC: (S,S-Whelk, 10 min, 2.5 mL/min, 1%(1:1 mixture of MeOH and i-PrOH + 0.2% FA)/CO2, UV 230 nm) RT: 1.81 min, 1.95 min.

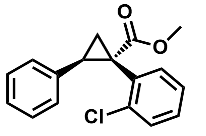

Methyl (1S,2R)-1-(2-chlorophenyl)-2-phenylcyclopropane-1-carboxylate (10)
This compound was prepared according to the General Procedure from the reaction between methyl 2-(2-chlorophenyl)-2-diazoacetate (0.100 mmol, 21.1 mg) and styrene (5.0 equiv, 0.500 mmol, 57 µL) in up to 85% yield. Spectra and characterization matched literature reported values. [2]

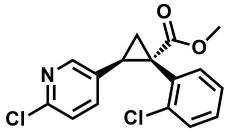

Methyl (1S,2R)-1-(2-chlorophenyl)-2-(6-chloropyridin-3-yl)cyclopropane-1-carboxylate (11)
This compound was prepared according to the General Procedure from the reaction between methyl 2-(2-chlorophenyl)-2-diazoacetate (0.100 mmol, 21.1 mg) and 2-chloro-5-vinylpyridine (5.0 equiv, 0.500 mmol, 69.8 mg) in up to 96% yield. Spectra and characterization matched literature reported values. [2]

NMR Spectra

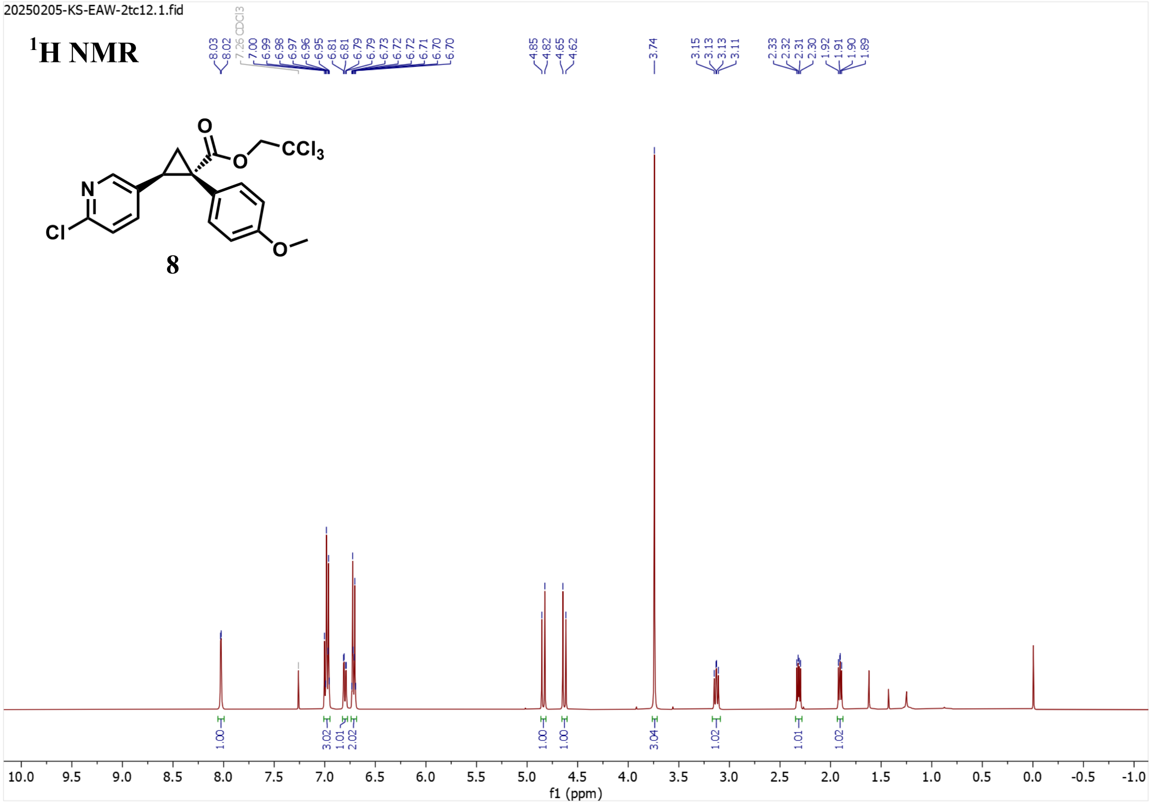
20250205-KS-EAW-2tc12.1.fid
1H NMR
8.03 8.02 7.26 CDCl3 7.00 6.99 6.98 6.97 6.96 6.95 6.81 6.81 6.79 6.73 6.72 6.72 6.71 6.70 6.70
4.85 4.82 4.65 4.62
3.74
3.15 3.13 3.13 3.11
2.33 2.32 2.31 2.30 1.92 1.91 1.90 1.89
O
O
CCl3
N
Cl
O
8
1.00 3.02 1.01 2.02 1.00 1.00 3.04 1.02 1.01 1.02
f1 (ppm)

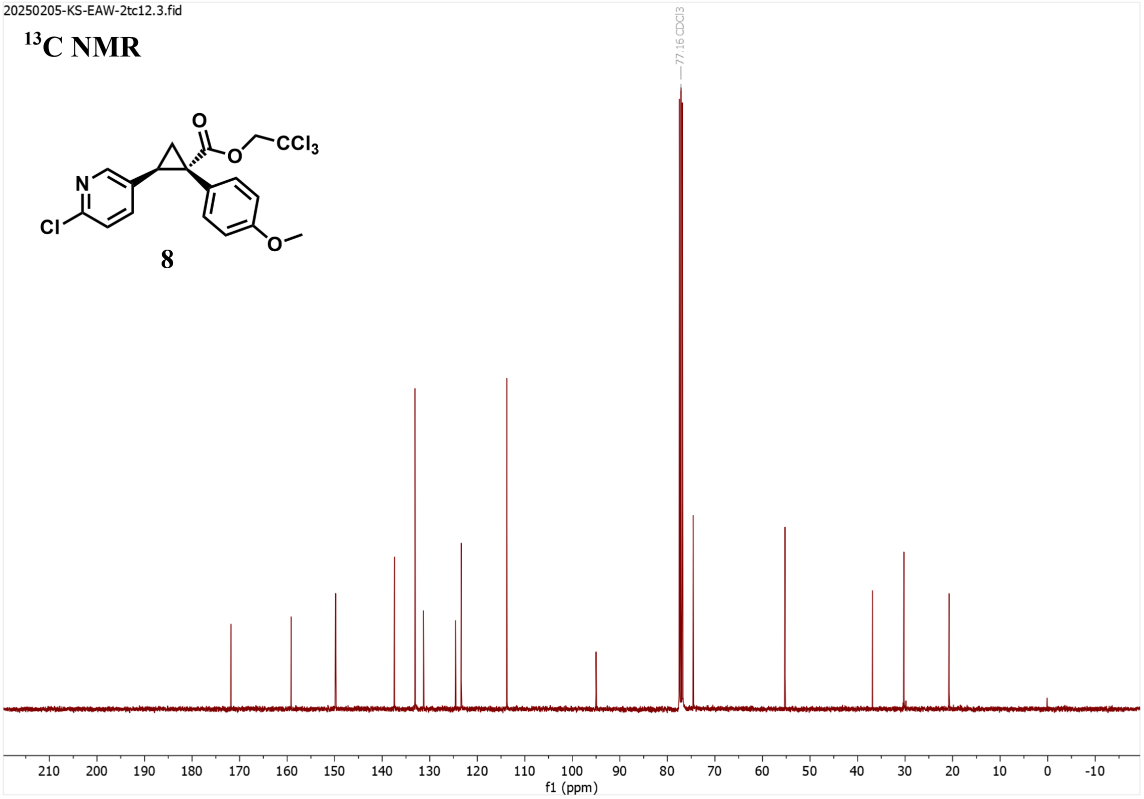
20250205-KS-EAW-2tc12.3.fid
13C NMR
77.16 CDCl3
O
O
CCl3
N
Cl
O
8
f1 (ppm)

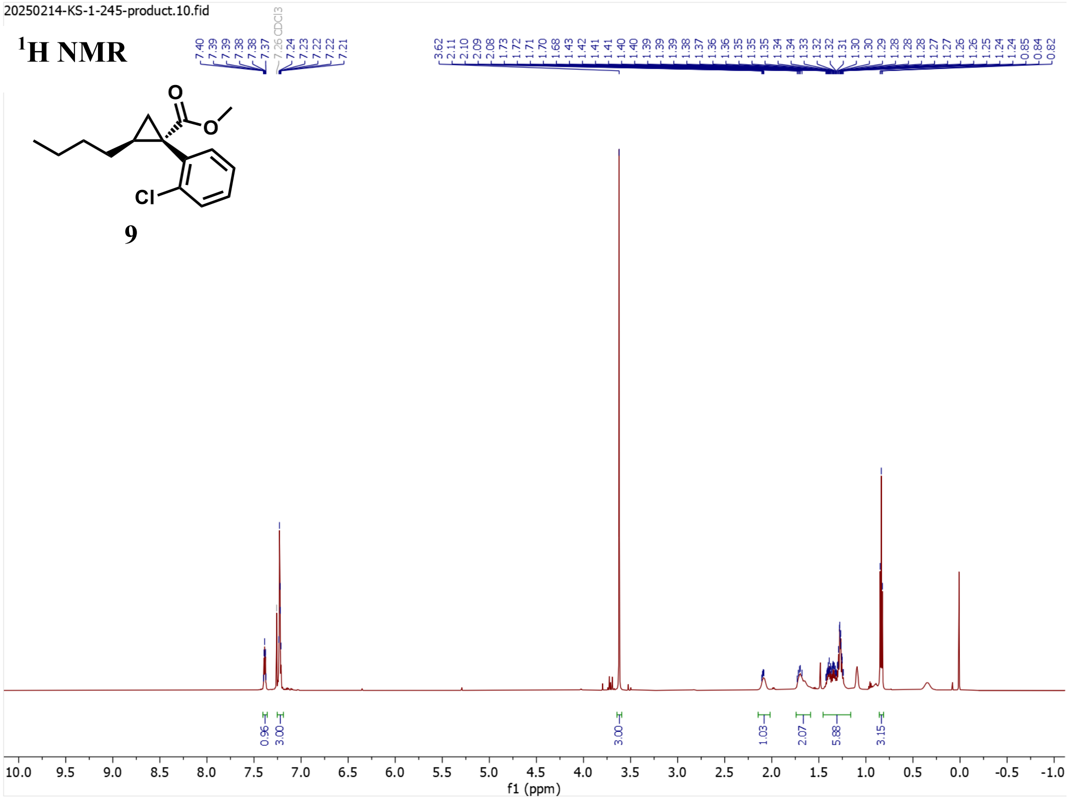
20250214-KS-1-245-product.10.fid
1H NMR
O
O
Cl
9
7.26 CDCl3
0.96
3.00
3.00
1.03
2.07
5.88
3.15
f1 (ppm)

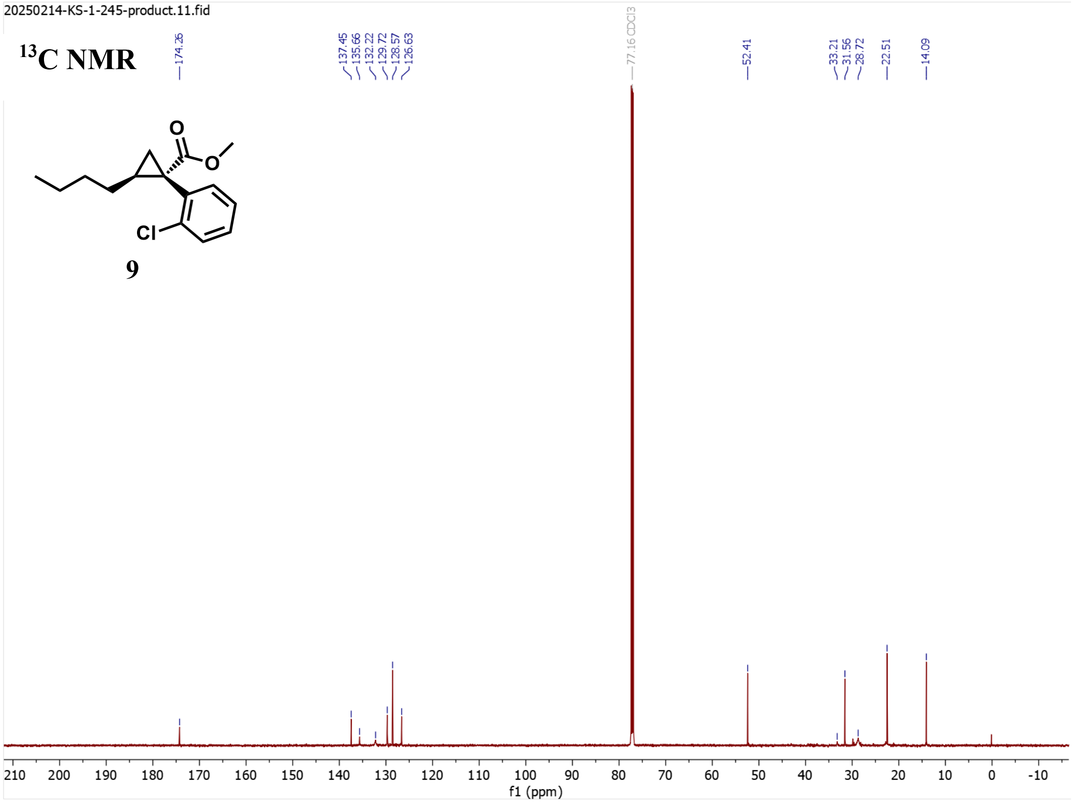
20250214-KS-1-245-product.11.fid
13C NMR
174.26
137.45
135.66
132.22
129.72
128.57
126.63
77.16 CDCl3
52.41
33.21
31.56
28.72
22.51
14.09
O
O
Cl
9
f1 (ppm)

HPLC and SFC Chromatograms

Equivalents screen

S-TCPTAD, 10 equiv HFIP, -72% ee (**3**)

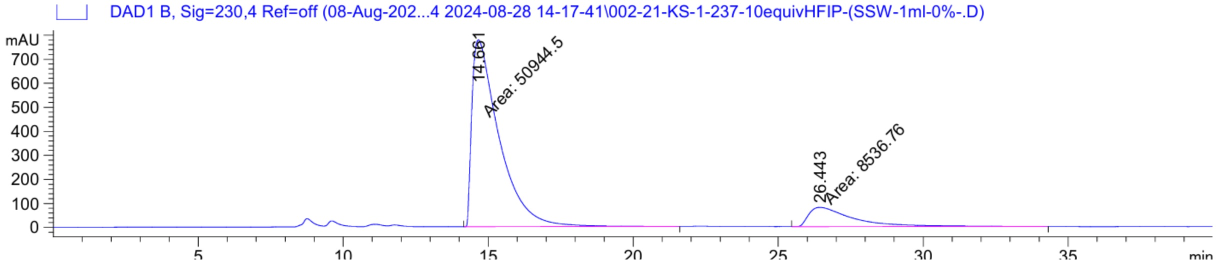

Signal 2: DAD1 B, Sig=230,4 Ref=off

Peak #	RetTime [min]	Type	Width [min]	Area [mAU*s]	Height [mAU]	Area %
1	14.661	MM	1.0921	5.09445e4	777.44159	85.6480
2	26.443	MM	1.7798	8536.76367	79.93974	14.3520
Totals :				5.94813e4	857.38132	

S-TCPTAD, 50 equiv HFIP, -36% ee (**3**)

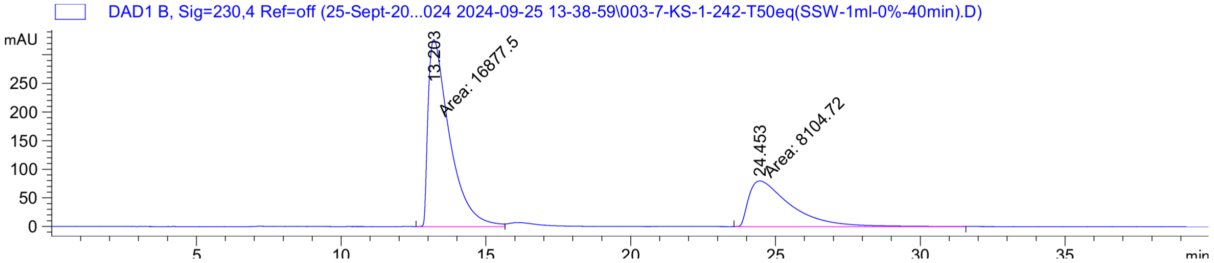

Signal 2: DAD1 B, Sig=230,4 Ref=off

Peak #	RetTime [min]	Type	Width [min]	Area [mAU*s]	Height [mAU]	Area %
1	13.203	MF	0.8606	1.68775e4	326.85016	67.5580
2	24.453	MM	1.6882	8104.71924	80.01279	32.4420
Totals :				2.49822e4	406.86295	

S-TCPTAD, 90 equiv HFIP, 30% ee (**3**)

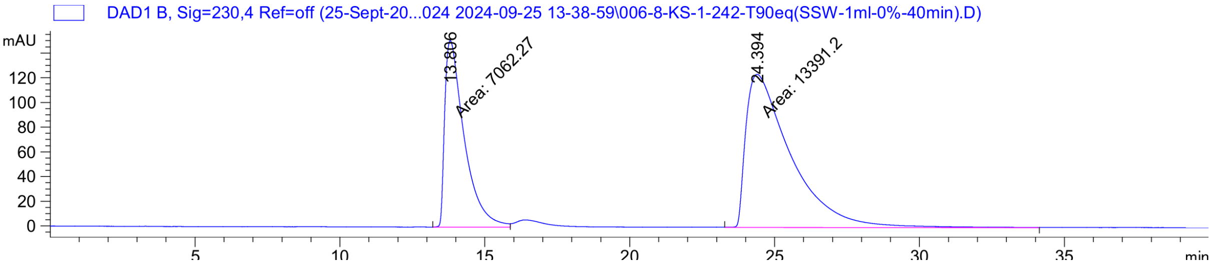

Signal 2: DAD1 B, Sig=230,4 Ref=off

Peak #	RetTime [min]	Type	Width [min]	Area [mAU*s]	Height [mAU]	Area %
1	13.806	MF	0.7795	7062.27393	150.99168	34.5285
2	24.394	MM	1.8006	1.33912e4	123.95123	65.4715
Totals :				2.04535e4	274.94292	

S-TCPTAD, 100 equiv HFIP, 48% ee (**3**)

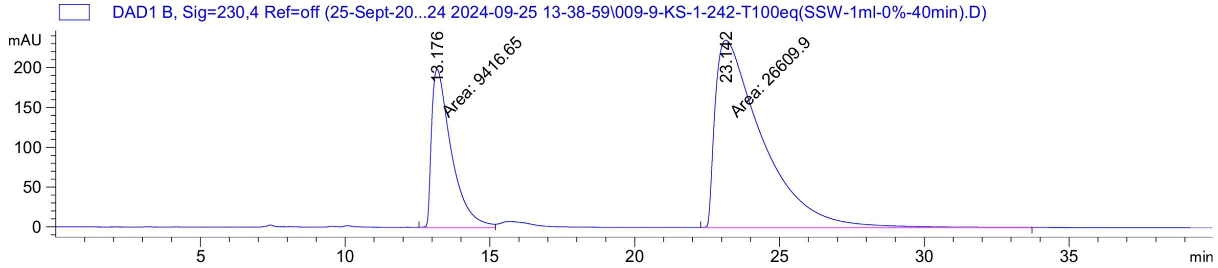

Signal 2: DAD1 B, Sig=230,4 Ref=off

Peak #	RetTime [min]	Type	Width [min]	Area [mAU*s]	Height [mAU]	Area %
1	13.176	MF	0.7901	9416.64648	198.63483	26.1380
2	23.142	MM	1.8839	2.66099e4	235.41595	73.8620
Totals :				3.60266e4	434.05078	

S-TCPTAD, 150 equiv HFIP, 68% ee (**3**)

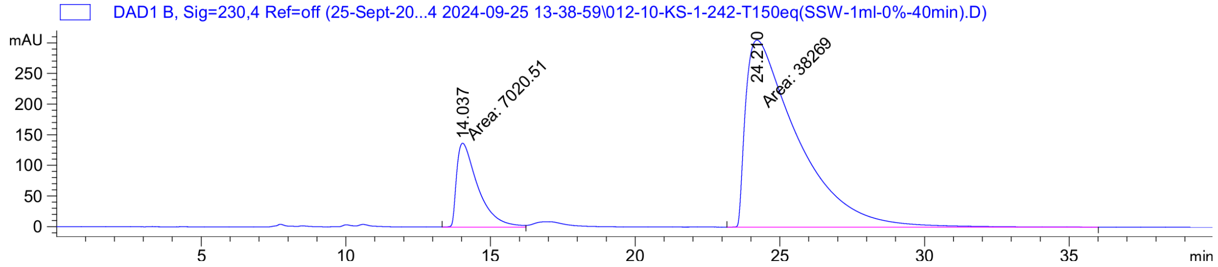

Signal 2: DAD1 B, Sig=230,4 Ref=off

Peak #	RetTime [min]	Type	Width [min]	Area [mAU*s]	Height [mAU]	Area %
1	14.037	MF	0.8547	7020.50928	136.89748	15.5014
2	24.210	MM	2.0891	3.82690e4	305.31146	84.4986
Totals :				4.52895e4	442.20894	

S-NTTL, 50 equiv HFIP, 84% ee (**3**)

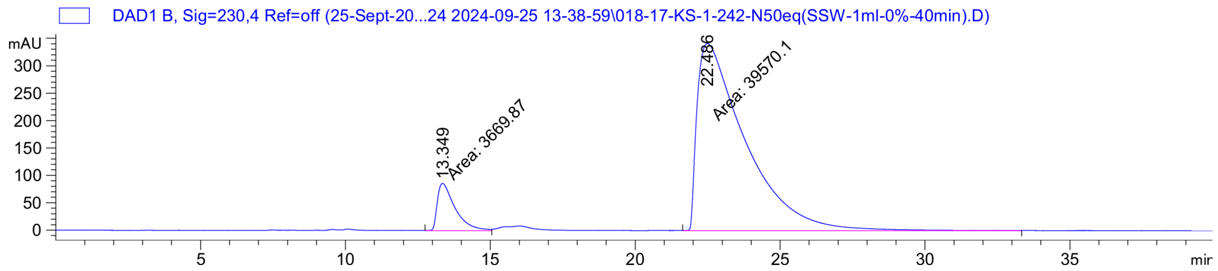

Signal 2: DAD1 B, Sig=230,4 Ref=off

Peak #	RetTime [min]	Type	Width [min]	Area [mAU*s]	Height [mAU]	Area %
1	13.349	MF	0.7110	3669.86963	86.02280	8.4872
2	22.486	MM	1.9330	3.95701e4	341.18475	91.5128
Totals :				4.32400e4	427.20756	

S-NTTL, 90 equiv HFIP, 86% ee (**3**)

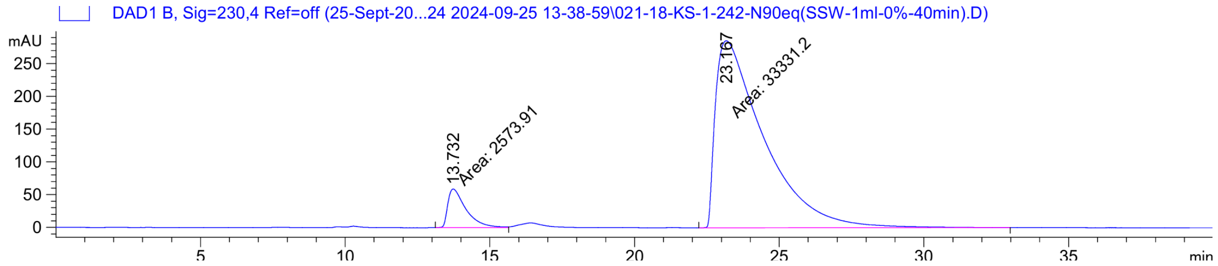

Signal 2: DAD1 B, Sig=230,4 Ref=off

Peak #	RetTime [min]	Type	Width [min]	Area [mAU*s]	Height [mAU]	Area %
1	13.732	MF	0.7297	2573.91235	58.78663	7.1686
2	23.167	MM	1.9456	3.33312e4	285.51950	92.8314
Totals :				3.59051e4	344.30613	

S-NTTL, 100 equiv HFIP, 86% ee (**3**)

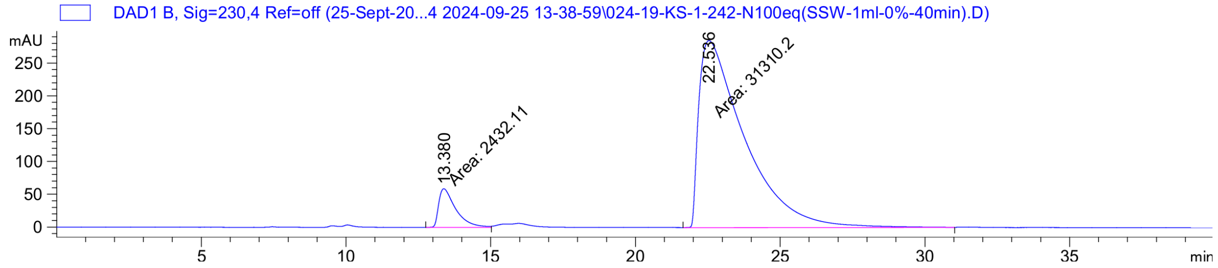

Signal 2: DAD1 B, Sig=230,4 Ref=off

Peak #	RetTime [min]	Type	Width [min]	Area [mAU*s]	Height [mAU]	Area %
1	13.380	MF	0.6865	2432.11133	59.04638	7.2079
2	22.536	MM	1.8308	3.13102e4	285.02771	92.7921
Totals :				3.37423e4	344.07409	

S-NTTL, 150 equiv HFIP, 86% ee (**3**)

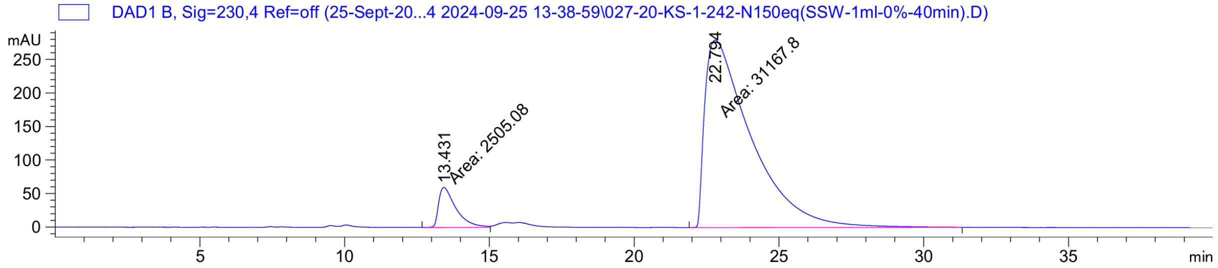


```
Signal 2: DAD1 B, Sig=230,4 Ref=off

Peak RetTime Type  Width     Area      Height     Area
  #    [min]        [min]   [mAU*s]     [mAU]       %
----|-------|----|-------|----------|----------|--------|
   1  13.431 MF    0.6980 2505.08057   59.81762   7.4395
   2  22.794 MM    1.8582 3.11678e4   279.55228  92.5605

Totals :                  3.36729e4   339.36990
```

Table S1: HFIP equivalence screen with **Rh-1** and **Rh-2**. The mole ratio is defined as the ratio of HFIP moles to the moles of aryldiazoacetate **1**. The mole ratio is defined as the ratio of HFIP moles to the moles of aryldiazoacetate **2**

HFIP equiv	mole ratio	% ee with **Rh-1**	% ee with **Rh-2**
0	0.00	36	-74
10	0.03	71	-72
50	0.17	84	-36
90	0.35	86	30
100	0.41	86	48
150	0.68	86	68
200	0.97	90	82

Solvent Screen

S-TPPTTL, DCM, -96% ee (**3**)

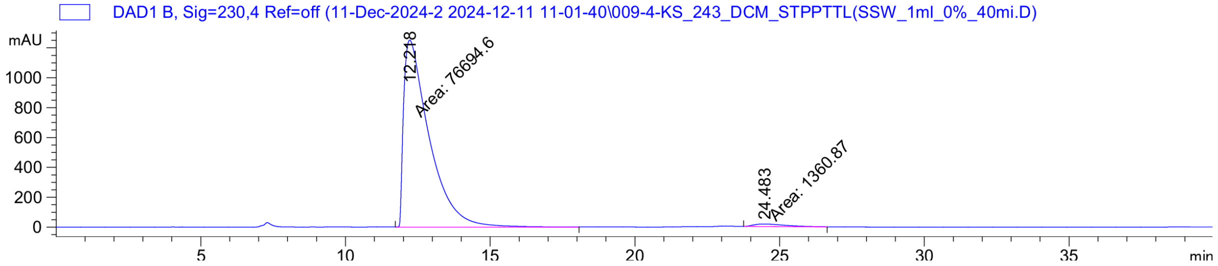

Signal 2: DAD1 B, Sig=230,4 Ref=off

Peak #	RetTime [min]	Type	Width [min]	Area [mAU*s]	Height [mAU]	Area %
1	12.218	MM	1.0216	7.66946e4	1251.23364	98.2565
2	24.483	MF	1.3069	1360.87097	17.35479	1.7435
Totals :				7.80555e4	1268.58844	

S-TPPTTL, 10 equiv HFIP, -38% ee (**3**)

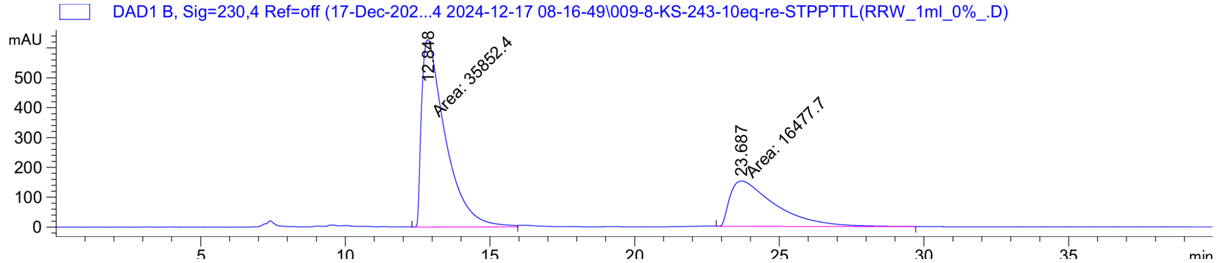

Signal 2: DAD1 B, Sig=230,4 Ref=off

Peak #	RetTime [min]	Type	Width [min]	Area [mAU*s]	Height [mAU]	Area %
1	12.848	MF	0.9521	3.58524e4	627.63147	68.5120
2	23.687	MM	1.8109	1.64777e4	151.65335	31.4880
Totals :				5.23300e4	779.28482	

S-TPPTTL, HFIP, -12% ee (**3**)

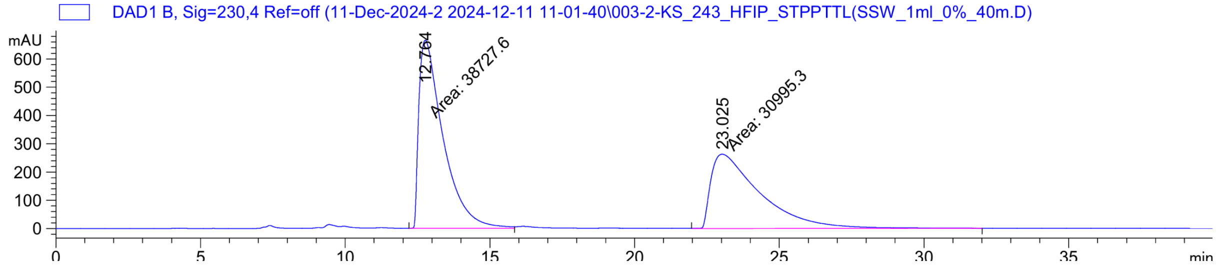

Signal 2: DAD1 B, Sig=230,4 Ref=off

Peak #	RetTime [min]	Type	Width [min]	Area [mAU*s]	Height [mAU]	Area %
1	12.764	MF	0.9700	3.87276e4	665.40076	55.5450
2	23.025	MM	1.9675	3.09953e4	262.56253	44.4550
Totals :				6.97228e4	927.96329	

S-TCPTAD, Cyclohexane, -76% ee (**3**)

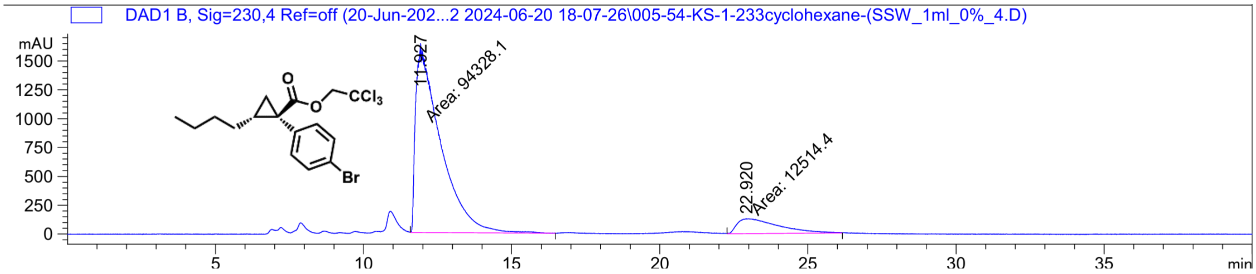

Signal 2: DAD1 B, Sig=230,4 Ref=off

Peak #	RetTime [min]	Type	Width [min]	Area [mAU*s]	Height [mAU]	Area %
1	11.927	MM	0.9593	9.43281e4	1638.84607	88.2871
2	22.920	MM	1.6154	1.25144e4	129.11121	11.7129
Totals :				1.06843e5	1767.95728	

S-TCPTAD, Ethyl Acetate, -74% ee (**3**)

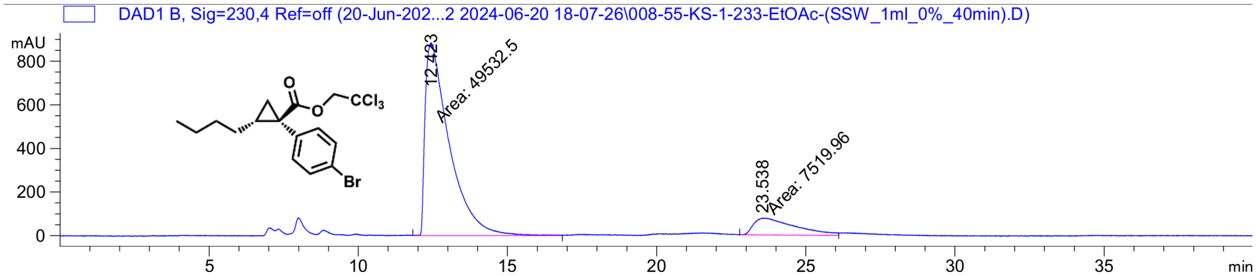

Signal 2: DAD1 B, Sig=230,4 Ref=off

Peak #	RetTime [min]	Type	Width [min]	Area [mAU*s]	Height [mAU]	Area %
1	12.423	MM	0.9345	4.95325e4	883.42401	86.8192
2	23.538	MM	1.6029	7519.96240	78.18930	13.1808
Totals :				5.70525e4	961.61331	

S-TCPTAD, Acetonitrile, -62% ee (**3**)

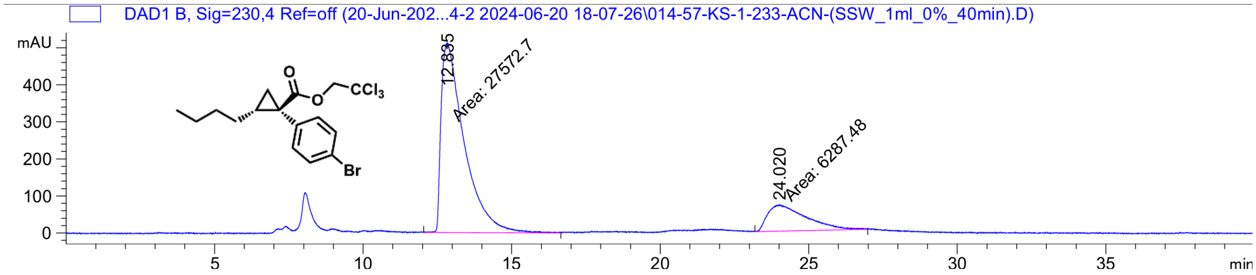

```
Signal 2: DAD1 B, Sig=230,4 Ref=off

Peak RetTime Type  Width     Area       Height     Area
  #    [min]        [min]   [mAU*s]     [mAU]       %
----|-------|----|-------|----------|----------|--------|
   1  12.835 MM    0.8960 2.75727e4   512.87305  81.4311
   2  24.020 MM    1.4774 6287.48438   70.93084  18.5689

Totals :                  3.38602e4   583.80389
```

S-TCPTAD, Trifluoroethanol, -56% ee (**3**)

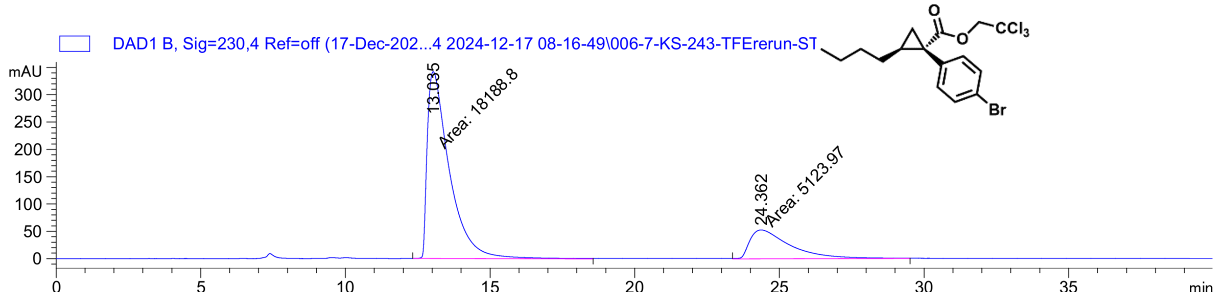


```
Signal 2: DAD1 B, Sig=230,4 Ref=off

Peak RetTime Type  Width     Area       Height     Area
  #    [min]        [min]   [mAU*s]     [mAU]       %
----|-------|----|-------|----------|----------|--------|
   1  13.035 MM    0.8861 1.81888e4   342.11200  78.0207
   2  24.362 MM    1.6179 5123.97168   52.78487  21.9793

Totals :                  2.33127e4   394.89687
```

Experimental Scope

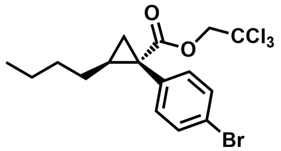

2,2,2-trichloroethyl (1*S*,2*S*)-1-(4-bromophenyl)-2-butylcyclopropane-1-carboxylate (3)

Racemate

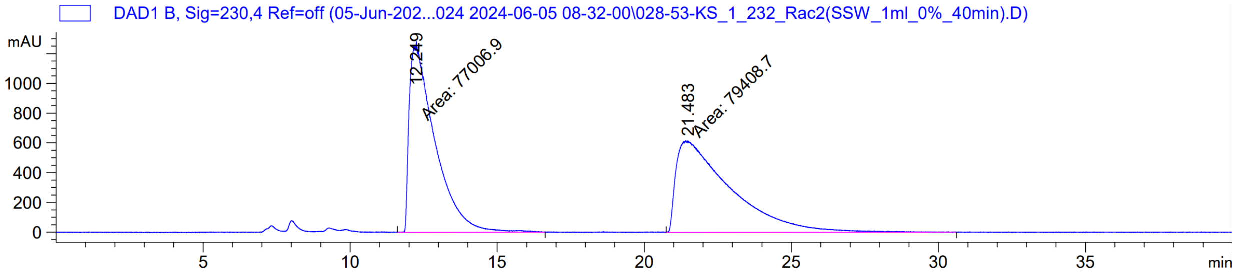


```
Signal 2: DAD1 B, Sig=230,4 Ref=off

Peak RetTime Type  Width     Area       Height     Area
  #    [min]        [min]   [mAU*s]     [mAU]       %
----|-------|----|-------|----------|----------|--------|
   1  12.249 MM    1.0007 7.70069e4  1282.59912  49.2322
   2  21.483 MM    2.1462 7.94087e4   616.66919  50.7678

Totals :                  1.56416e5  1899.26831
```

S-TCPTAD, DCM, -74% ee

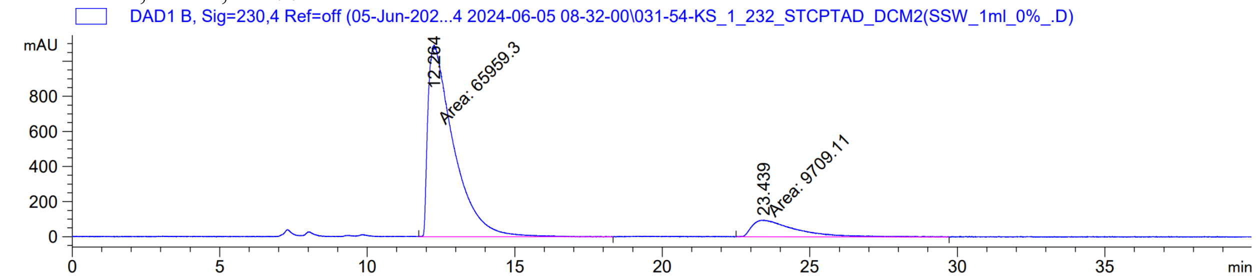

Signal 2: DAD1 B, Sig=230,4 Ref=off

Peak #	RetTime [min]	Type	Width [min]	Area [mAU*s]	Height [mAU]	Area %
1	12.264	MM	1.0053	6.59593e4	1093.52991	87.1689
2	23.439	MM	1.7064	9709.11133	94.82977	12.8311
Totals :				7.56684e4	1188.35968	

S-TCPTAD, HFIP, 82% ee

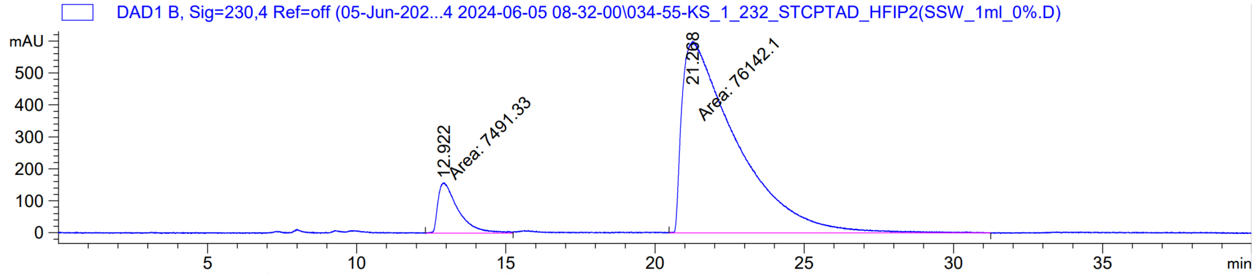

Signal 2: DAD1 B, Sig=230,4 Ref=off

Peak #	RetTime [min]	Type	Width [min]	Area [mAU*s]	Height [mAU]	Area %
1	12.922	MM	0.7919	7491.32666	157.65823	8.9573
2	21.268	MM	2.1149	7.61421e4	600.05365	91.0427
Totals :				8.36335e4	757.71188	

S-NTTL, DCM, 36% ee

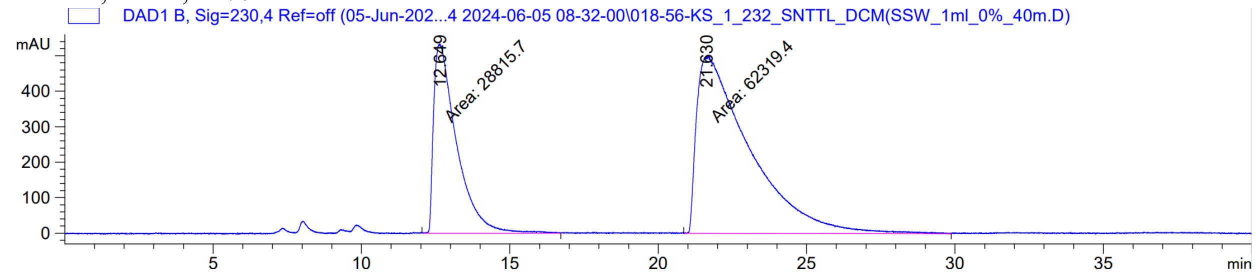

Signal 2: DAD1 B, Sig=230,4 Ref=off

Peak #	RetTime [min]	Type	Width [min]	Area [mAU*s]	Height [mAU]	Area %
1	12.649	MM	0.8999	2.88157e4	533.65753	31.6186
2	21.630	MM	2.0817	6.23194e4	498.95230	68.3814
Totals :				9.11351e4	1032.60983	

S-NTTL, HFIP, 90% ee

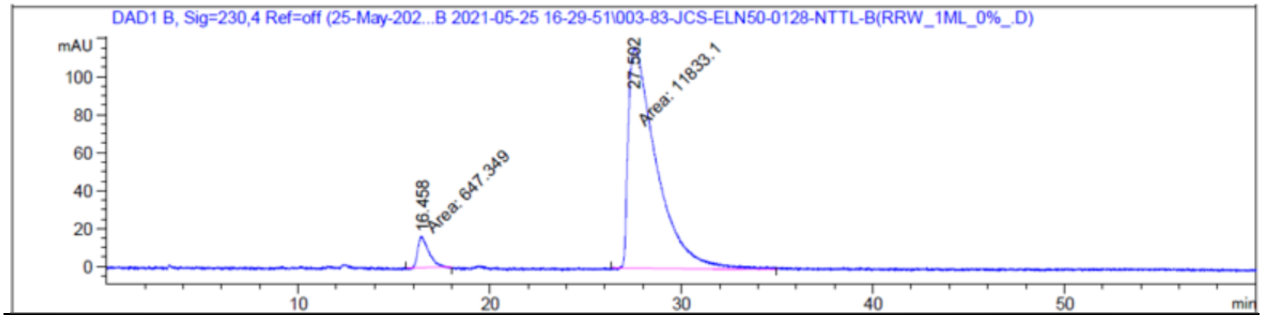

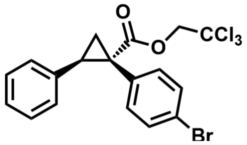

2,2,2-Trichloroethyl (1S,2R)-1-(4-bromophenyl)-2-phenylcyclopropane-1-carboxylate (4)

Racemate

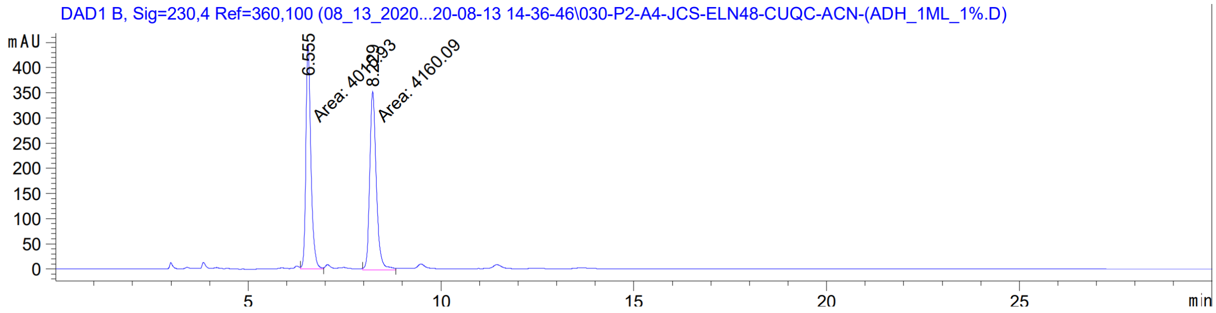

Signal 2: DAD1 B, Sig=230,4 Ref=360,100

Peak #	RetTime [min]	Type	Width [min]	Area [mAU*s]	Height [mAU]	Area %
1	6.555	MM	0.1498	4015.92651	446.79294	49.1184
2	8.229	MM	0.1961	4160.08594	353.50052	50.8816
Totals :				8176.01245	800.29346	

S-TCPTAD, DCM, -70% ee

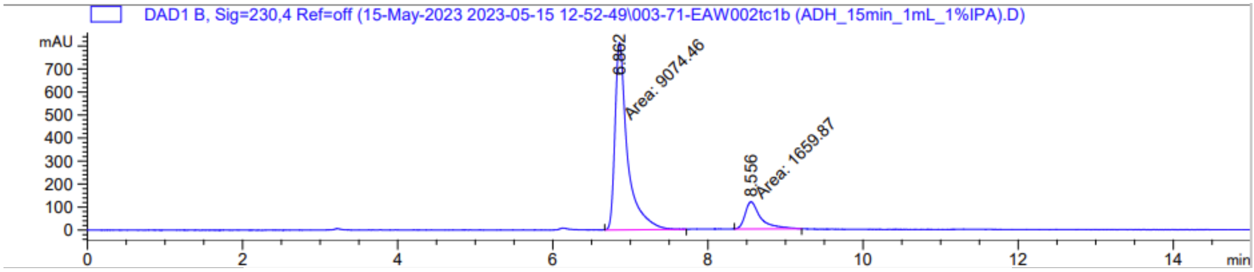

Signal 2: DAD1 B, Sig=230,4 Ref=off

Peak #	RetTime [min]	Type	Width [min]	Area [mAU*s]	Height [mAU]	Area %
1	6.862	MM	0.1854	9074.45996	815.81763	84.5368
2	8.556	MM	0.2327	1659.86560	118.87025	15.4632
Totals :				1.07343e4	934.68788	

S-TCPTAD, HFIP, 36% ee

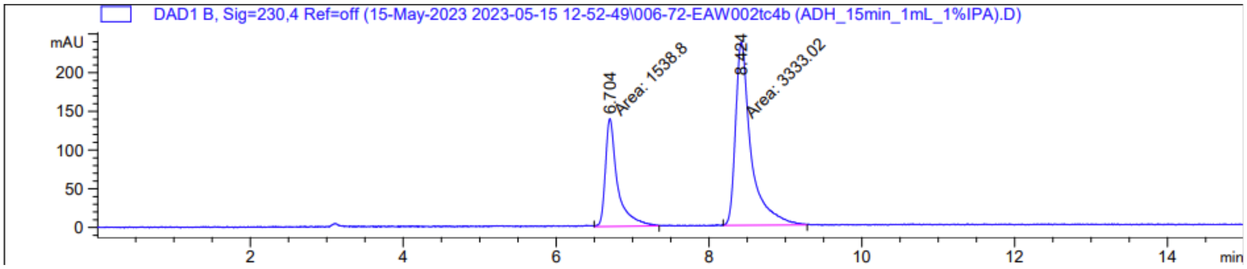

Signal 2: DAD1 B, Sig=230,4 Ref=off

Peak #	RetTime [min]	Type	Width [min]	Area [mAU*s]	Height [mAU]	Area %
1	6.704	MM	0.1843	1538.80432	139.16614	31.5858
2	8.424	MM	0.2343	3333.02490	237.09358	68.4142
Totals :				4871.82922	376.25972	

S-NTTL, DCM, 22% ee

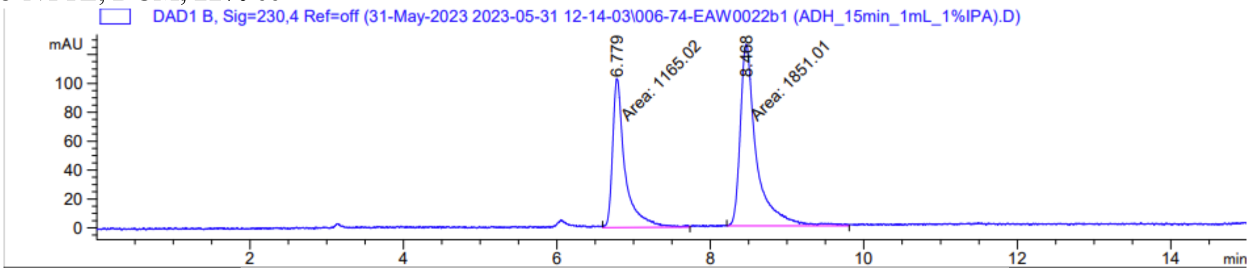

Signal 2: DAD1 B, Sig=230,4 Ref=off

Peak #	RetTime [min]	Type	Width [min]	Area [mAU*s]	Height [mAU]	Area %
1	6.779	MM	0.1881	1165.01611	103.21133	38.6275
2	8.468	MM	0.2450	1851.01147	125.90762	61.3725
Totals :				3016.02759	229.11895	

S-NTTL, HFIP, 66% ee

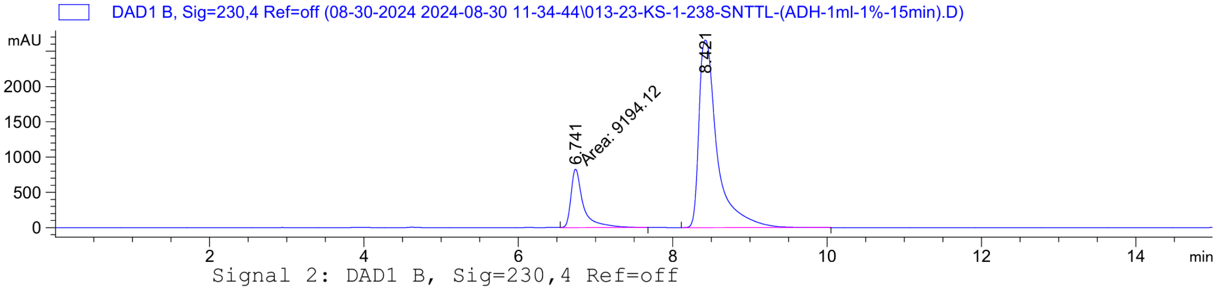

Signal 2: DAD1 B, Sig=230,4 Ref=off

Peak #	RetTime [min]	Type	Width [min]	Area [mAU*s]	Height [mAU]	Area %
1	6.741	FM	0.1851	9194.11621	827.71973	17.4465
2	8.421	BB	0.1941	4.35049e4	2655.90186	82.5535
Totals :				5.26990e4	3483.62158	

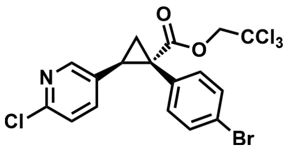

2,2,2-trichloroethyl (1S,2R)-1-(4-bromophenyl)-2-(6-chloropyridin-3-yl)cyclopropane-1-carboxylate (5)

Racemate

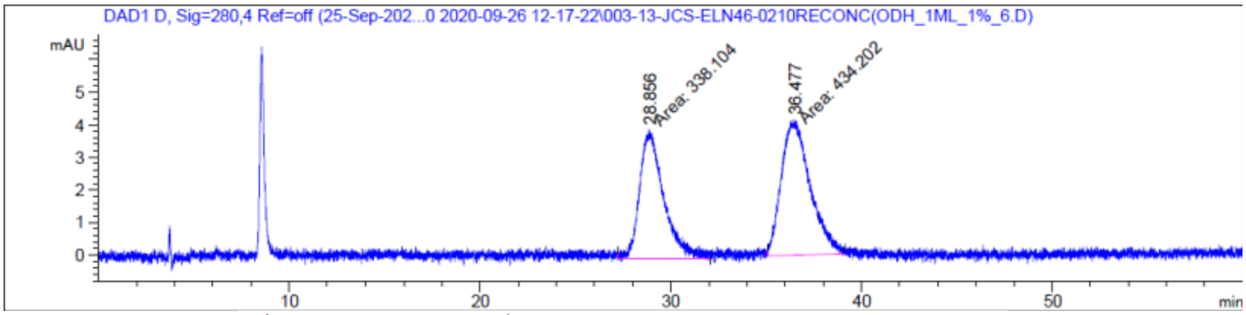

Signal 4: DAD1 D, Sig=280,4 Ref=off

Peak #	RetTime [min]	Type	Width [min]	Area [mAU*s]	Height [mAU]	Area %
1	28.856	MM	1.4272	338.10449	3.94839	43.7785
2	36.477	MM	1.7347	434.20224	4.17165	56.2215
Totals :				772.30673	8.12004	

S-TCPTAD, DCM, -46% ee

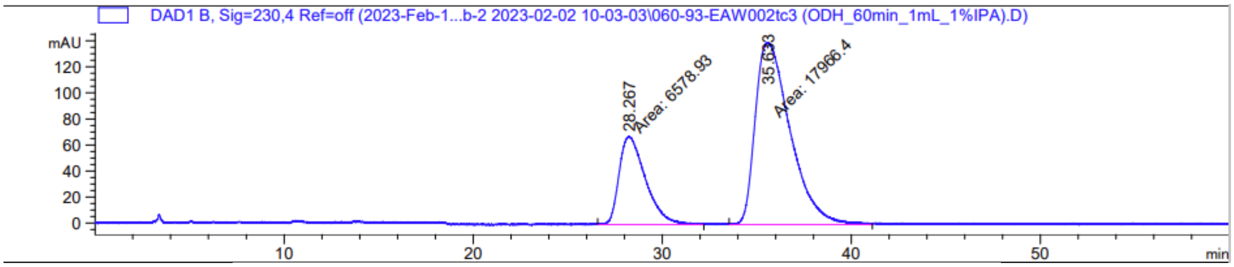

Signal 2: DAD1 B, Sig=230,4 Ref=off

Peak #	RetTime [min]	Type	Width [min]	Area [mAU*s]	Height [mAU]	Area %
1	28.267	MM	1.6141	6578.93018	67.93063	26.8032
2	35.633	MM	2.1412	1.79664e4	139.84868	73.1968
Totals :				2.45453e4	207.77930	

S-TCPTAD, HFIP, 76% ee

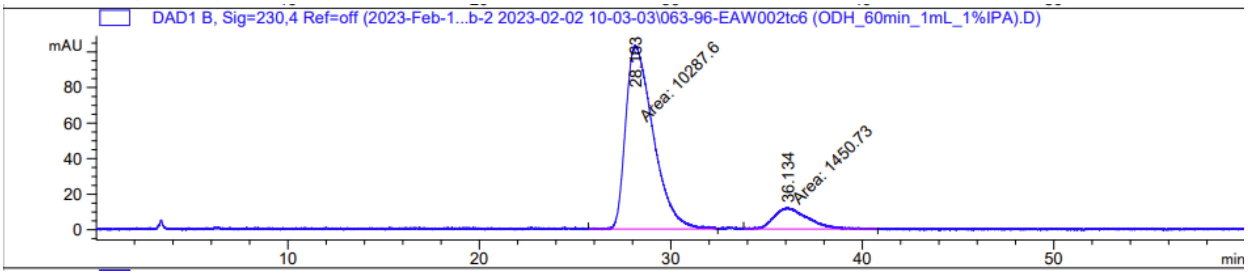

Signal 2: DAD1 B, Sig=230,4 Ref=off

Peak #	RetTime [min]	Type	Width [min]	Area [mAU*s]	Height [mAU]	Area %
1	28.163	MM	1.6608	1.02876e4	103.24177	87.6411
2	36.134	MM	2.0199	1450.73059	11.97038	12.3589
Totals :				1.17383e4	115.21214	

S-NTTL, DCM, 24% ee

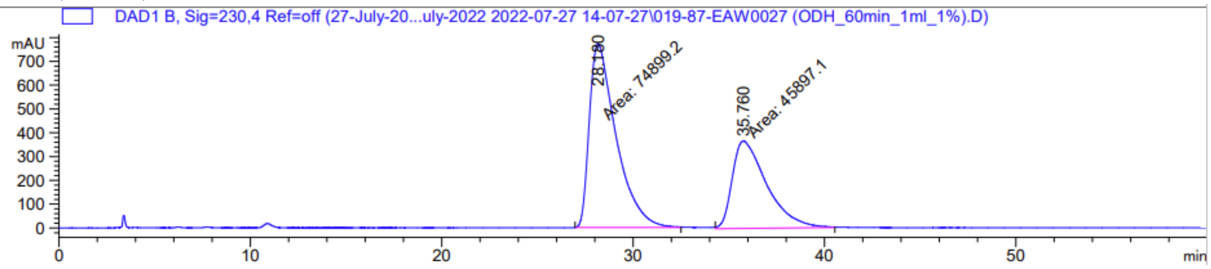

Signal 2: DAD1 B, Sig=230,4 Ref=off

Peak #	RetTime [min]	Type	Width [min]	Area [mAU*s]	Height [mAU]	Area %
1	28.180	MM	1.6168	7.48992e4	772.09351	62.0046
2	35.760	MM	2.0814	4.58971e4	367.50894	37.9954
Totals :				1.20796e5	1139.60245	

S-NTTL, HFIP, 64% ee

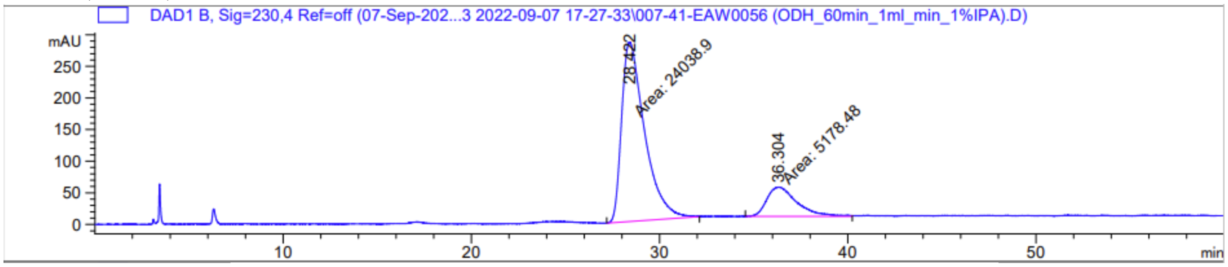

Signal 2: DAD1 B, Sig=230,4 Ref=off

Peak #	RetTime [min]	Type	Width [min]	Area [mAU*s]	Height [mAU]	Area %
1	28.422	MM	1.4124	2.40389e4	283.67029	82.2760
2	36.304	MM	1.8678	5178.47949	46.20899	17.7240
Totals :				2.92174e4	329.87928	

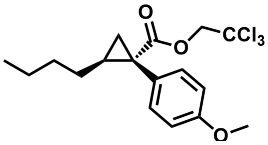

2,2,2-trichloroethyl (1S,2S)-2-butyl-1-(4-methoxyphenyl)cyclopropane-1-carboxylate (6)

Racemate

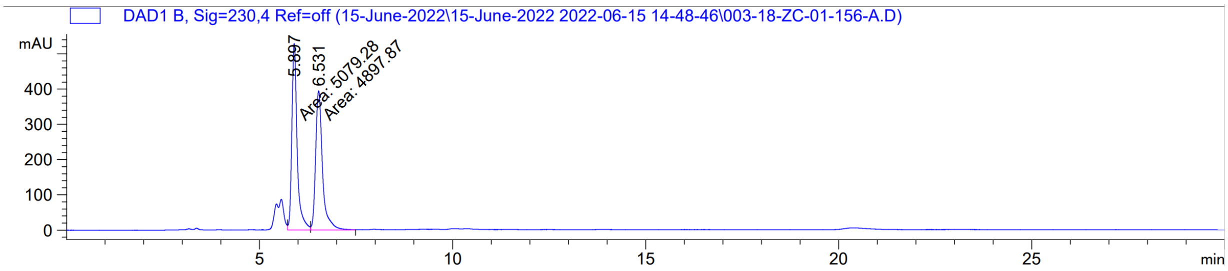

Signal 2: DAD1 B, Sig=230,4 Ref=off

Peak #	RetTime [min]	Type	Width [min]	Area [mAU*s]	Height [mAU]	Area %
1	5.897	MF	0.1601	5079.27783	528.87756	50.9091
2	6.531	FM	0.2068	4897.86963	394.64755	49.0909
Totals :				9977.14746	923.52512	

S-TCPTAD, DCM, -58% ee

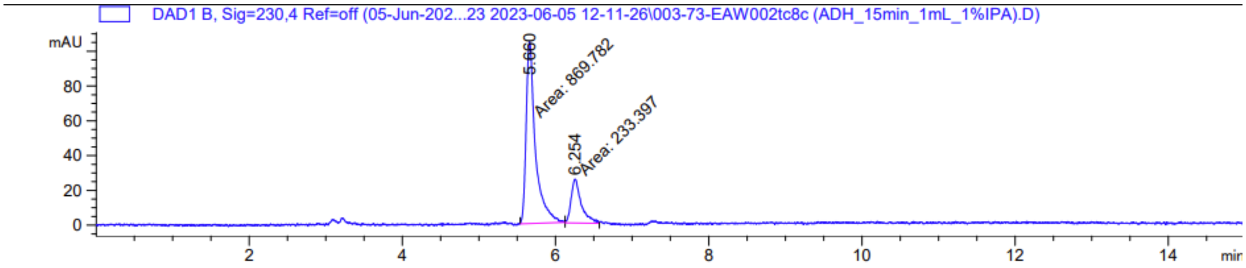

Signal 2: DAD1 B, Sig=230,4 Ref=off

Peak #	RetTime [min]	Type	Width [min]	Area [mAU*s]	Height [mAU]	Area %
1	5.660	MM	0.1386	869.78180	104.58714	78.8432
2	6.254	MM	0.1538	233.39691	25.28528	21.1568
Totals :				1103.17871	129.87242	

S-TCPTAD, HFIP, 82% ee

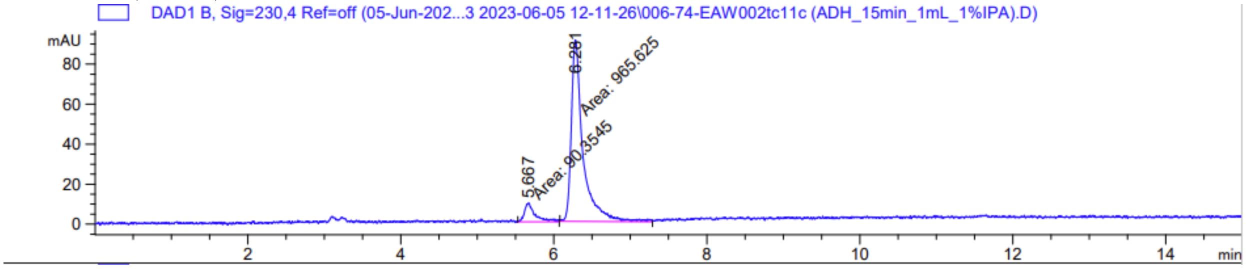

Signal 2: DAD1 B, Sig=230,4 Ref=off

Peak #	RetTime [min]	Type	Width [min]	Area [mAU*s]	Height [mAU]	Area %
1	5.667	MM	0.1603	90.35449	9.39494	8.5565
2	6.281	MM	0.1774	965.62469	90.71745	91.4435
Totals :				1055.97919	100.11238	

S-NTTL, DCM, 52% ee

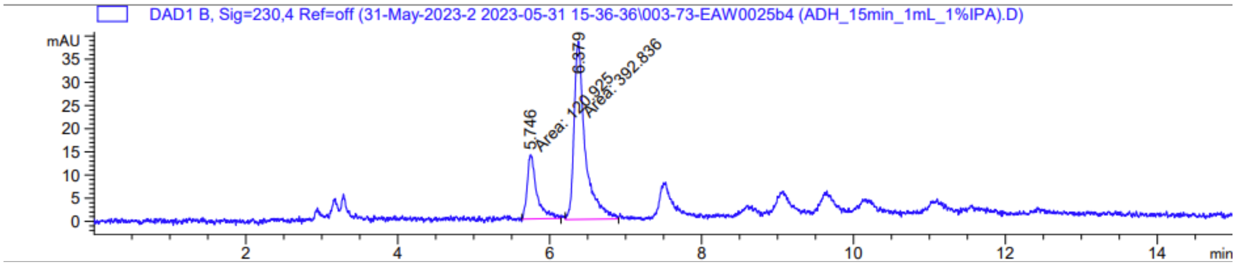

Signal 2: DAD1 B, Sig=230,4 Ref=off

Peak #	RetTime [min]	Type	Width [min]	Area [mAU*s]	Height [mAU]	Area %
1	5.746	MM	0.1456	120.92500	13.84566	23.5372
2	6.379	MM	0.1700	392.83618	38.52043	76.4628
Totals :				513.76118	52.36609	

S-NTTL, HFIP, 82% ee

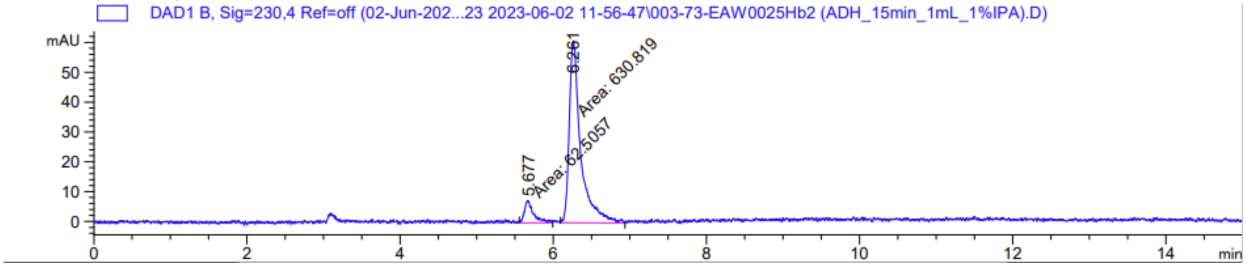

Signal 2: DAD1 B, Sig=230,4 Ref=off

Peak #	RetTime [min]	Type	Width [min]	Area [mAU*s]	Height [mAU]	Area %
1	5.677	MM	0.1407	62.50574	7.40405	9.0154
2	6.261	MM	0.1722	630.81909	61.06207	90.9846
Totals :				693.32484	68.46613	

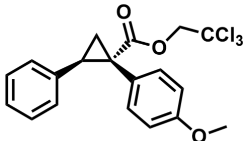

2,2,2-trichloroethyl (1S,2R)-1-(4-methoxyphenyl)-2-phenylcyclopropane-1-carboxylate (7)

Racemate

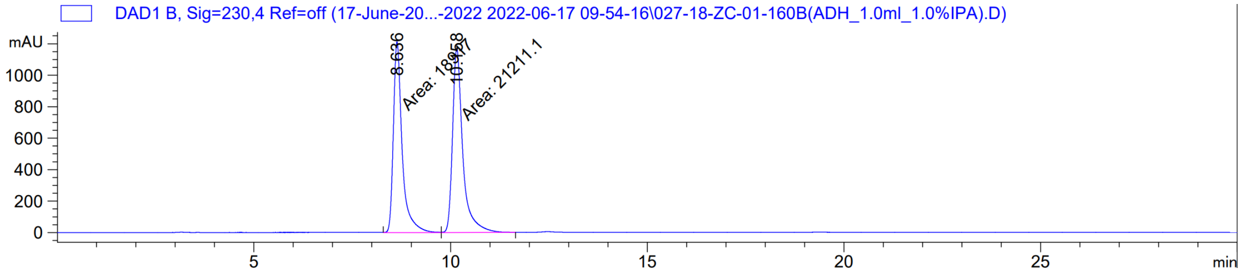

Signal 2: DAD1 B, Sig=230,4 Ref=off

Peak #	RetTime [min]	Type	Width [min]	Area [mAU*s]	Height [mAU]	Area %
1	8.636	MF	0.2599	1.89170e4	1212.95544	47.1415
2	10.158	FM	0.3022	2.12111e4	1169.81982	52.8585
Totals :				4.01281e4	2382.77527	

S-TCPTAD, DCM, -56% ee

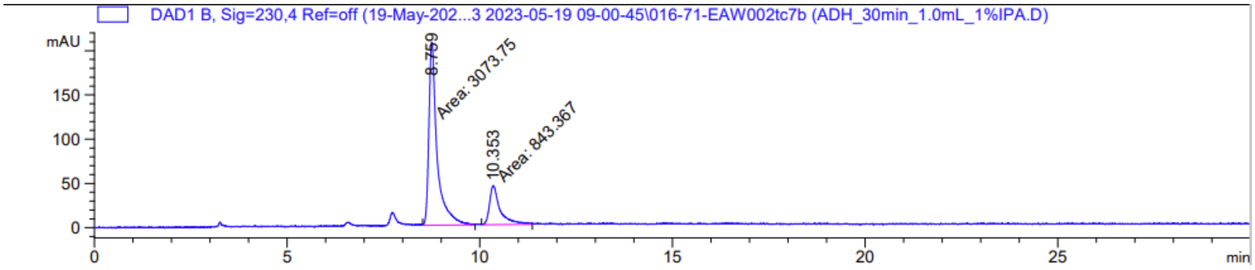

Signal 2: DAD1 B, Sig=230,4 Ref=off

Peak #	RetTime [min]	Type	Width [min]	Area [mAU*s]	Height [mAU]	Area %
1	8.759	MM	0.2474	3073.75220	207.03125	78.4697
2	10.353	MM	0.3168	843.36731	44.36893	21.5303
Totals :				3917.11951	251.40018	

S-TCPTAD, HFIP, 24% ee

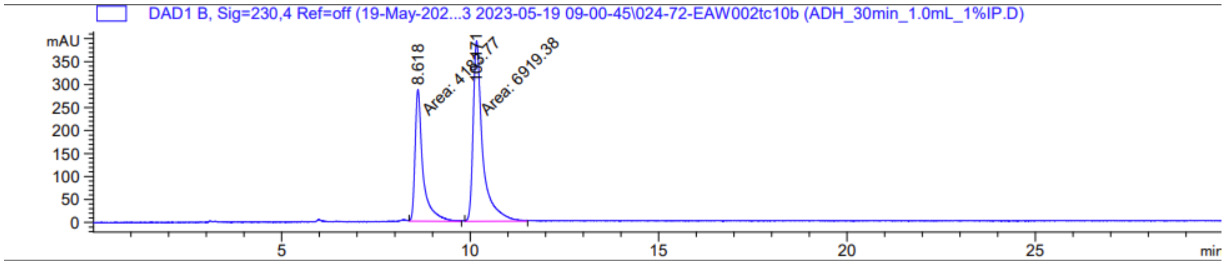

Signal 2: DAD1 B, Sig=230,4 Ref=off

Peak #	RetTime [min]	Type	Width [min]	Area [mAU*s]	Height [mAU]	Area %
1	8.618	MM	0.2442	4183.76563	285.57886	37.6809
2	10.171	MM	0.2935	6919.37842	392.89468	62.3191
Totals :				1.11031e4	678.47354	

S-NTTL, DCM, 38% ee

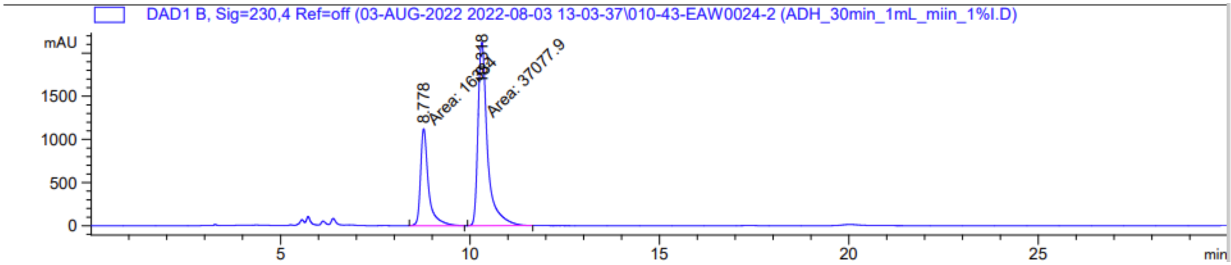

Signal 2: DAD1 B, Sig=230,4 Ref=off

Peak #	RetTime [min]	Type	Width [min]	Area [mAU*s]	Height [mAU]	Area %
1	8.778	MM	0.2430	1.63840e4	1123.59717	30.6461
2	10.318	MM	0.2905	3.70779e4	2127.13452	69.3539
Totals :				5.34619e4	3250.73169	

S-NTTL, HFIP, 84% ee

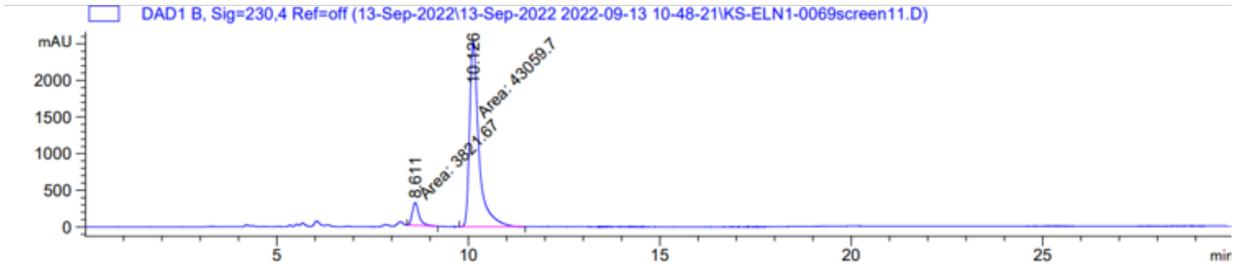

Signal 2: DAD1 B, Sig=230,4 Ref=off

Peak #	RetTime [min]	Type	Width [min]	Area [mAU*s]	Height [mAU]	Area %
1	8.611	MM	0.2046	3821.67236	311.37387	8.1518
2	10.126	MM	0.2833	4.30597e4	2533.48853	91.8482
Totals :				4.68814e4	2844.86240	

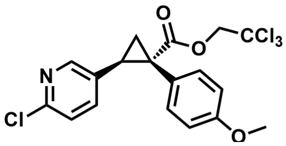

2,2,2-trichloroethyl(1S,2R)-2-(6-chloropyridin-3-yl)-1-(4-methoxyphenyl)cyclopropane-1-carboxylate (8)

Racemate

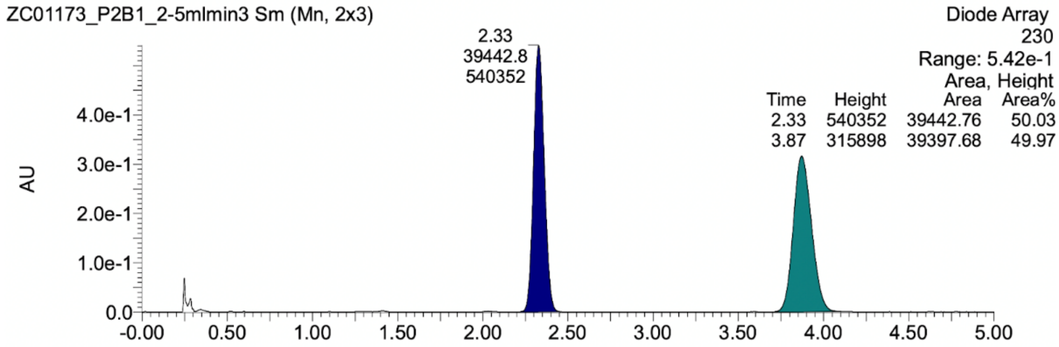

S-TCPTAD, DCM, -40% ee

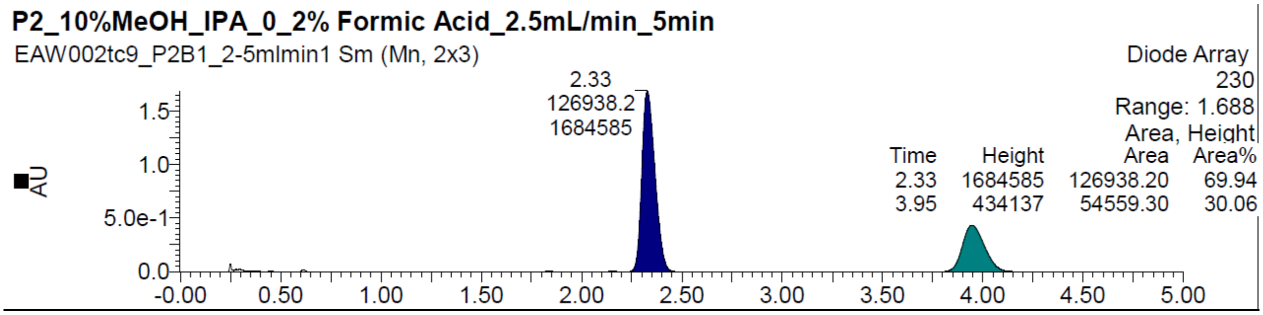

S-TCPTAD, HFIP, 76% ee

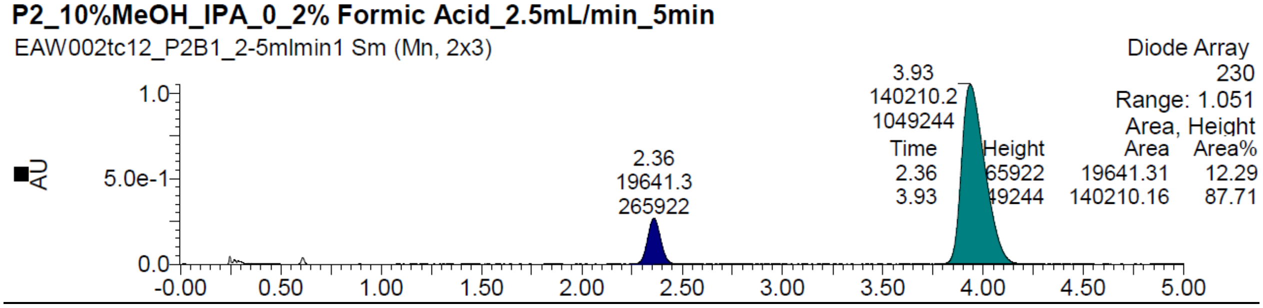

S-NTTL, DCM, 52% ee

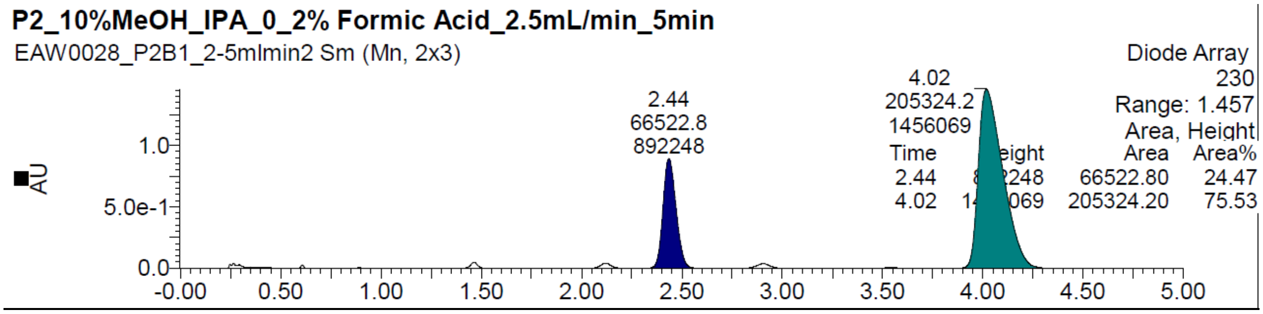

S-NTTL, HFIP, 74% ee

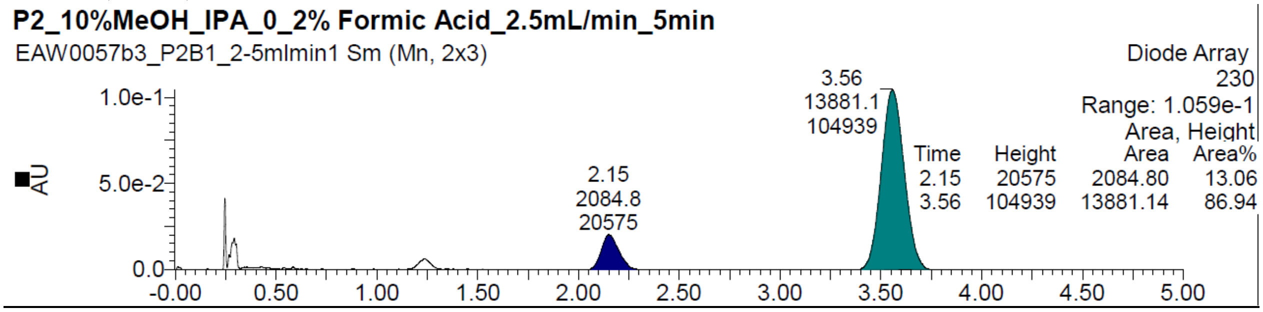

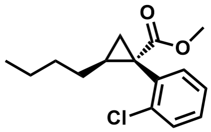

Methyl (1S,2S)-2-butyl-1-(2-chlorophenyl)cyclopropane-1-carboxylate (9)

Racemate

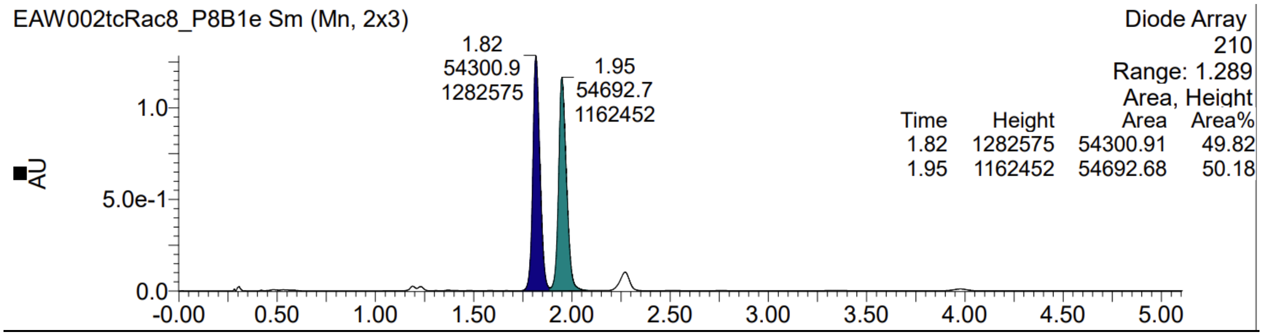

S-TCPTAD, DCM, 32% ee

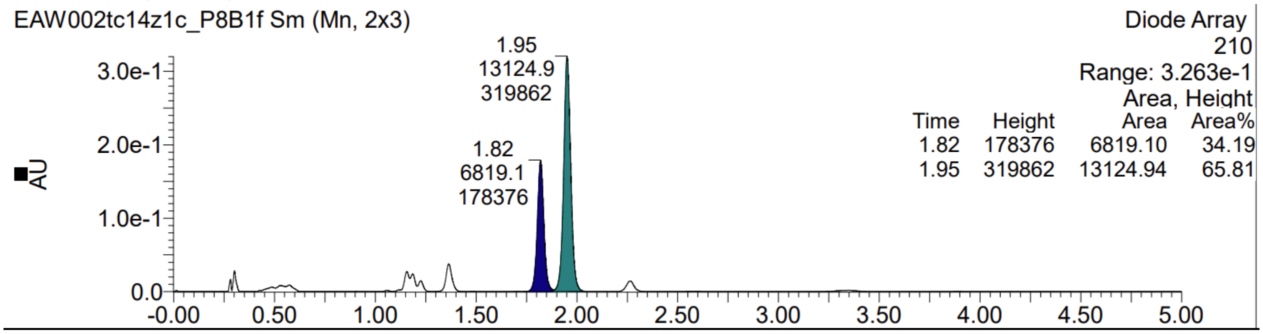

S-TCPTAD, HFIP, 72% ee

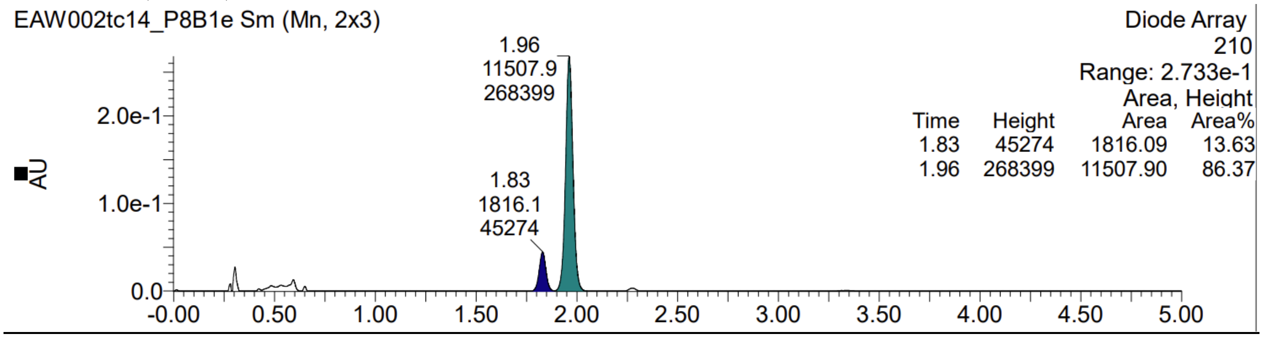

S-NTTL, DCM, 48% ee

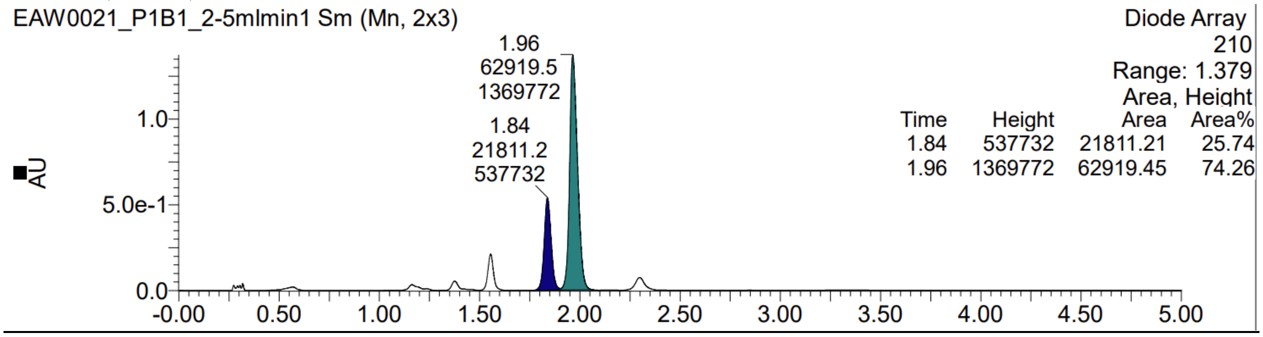

S-NTTL, HFIP, 90% ee

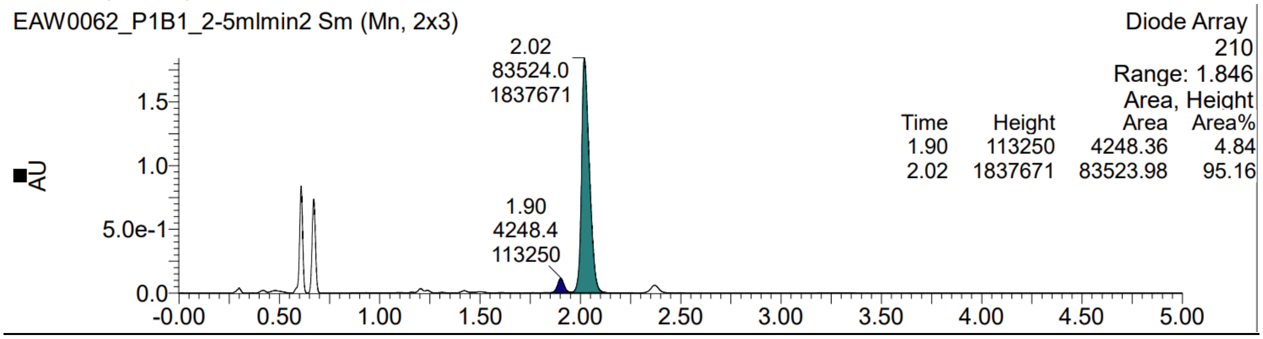

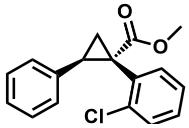

Methyl (1S,2R)-1-(2-chlorophenyl)-2-phenylcyclopropane-1-carboxylate (10)

Racemate

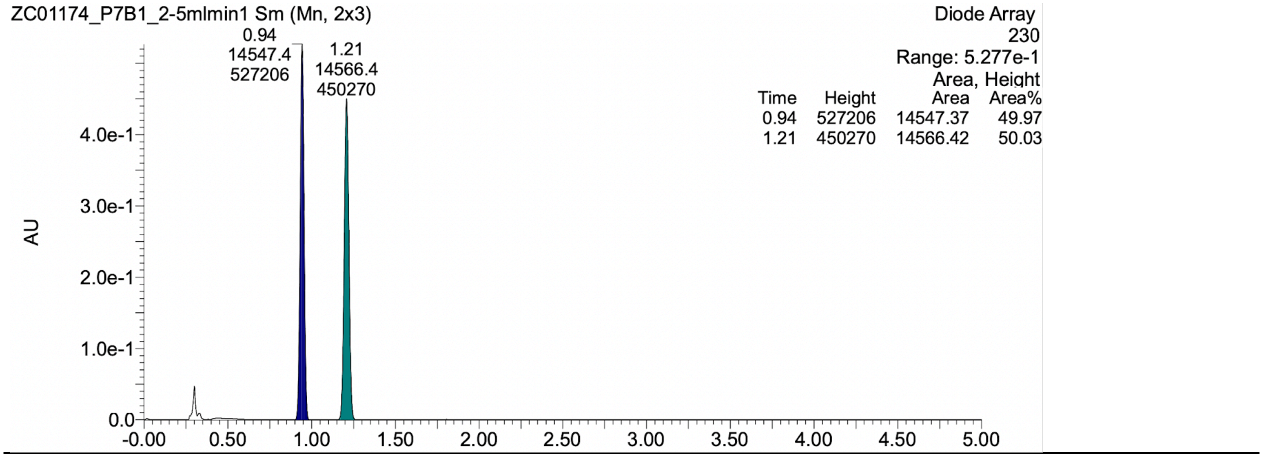

S-TCPTAD, DCM, 36% ee

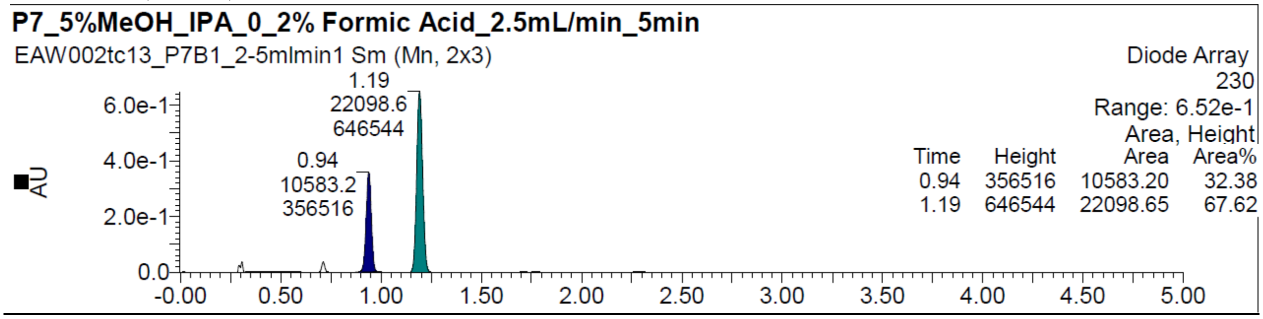

S-TCPTAD, HFIP, 30% ee

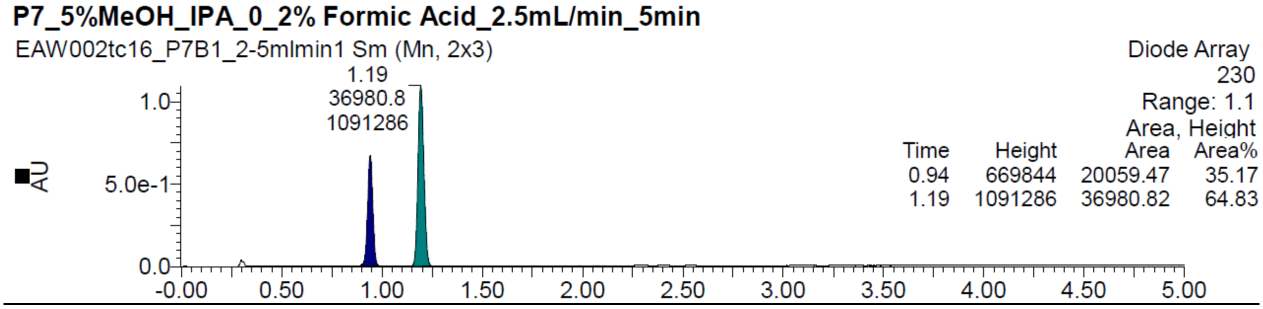

S-NTTL, DCM, 60% ee

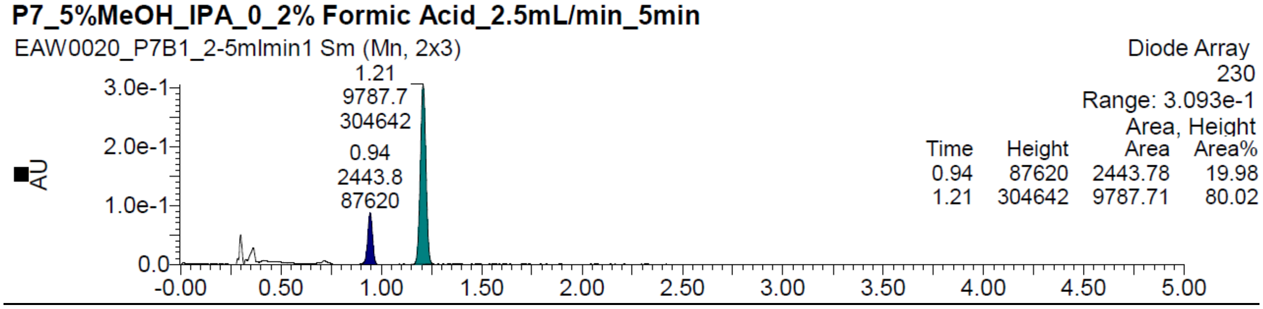

S-NTTL, HFIP, 82% ee

P7_5%MeOH_IPA_0_2% Formic Acid_2.5mL/min_5min

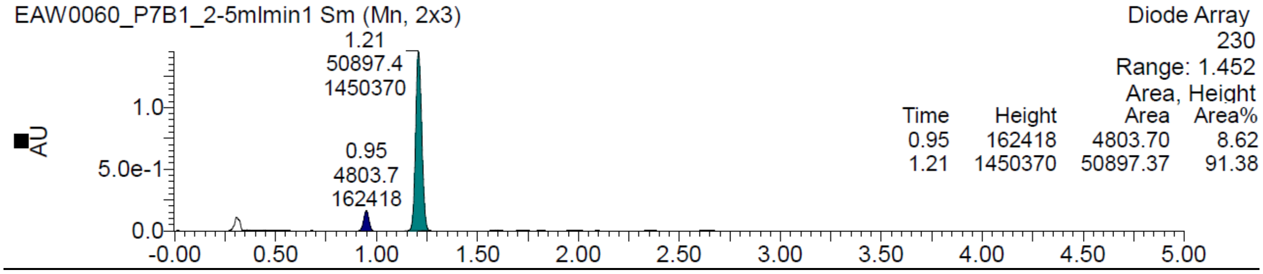

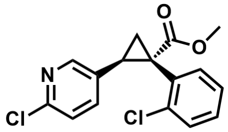

Methyl (1S,2R)-1-(2-chlorophenyl)-2-(6-chloropyridin-3-yl)cyclopropane-1-carboxylate (11)

Racemate

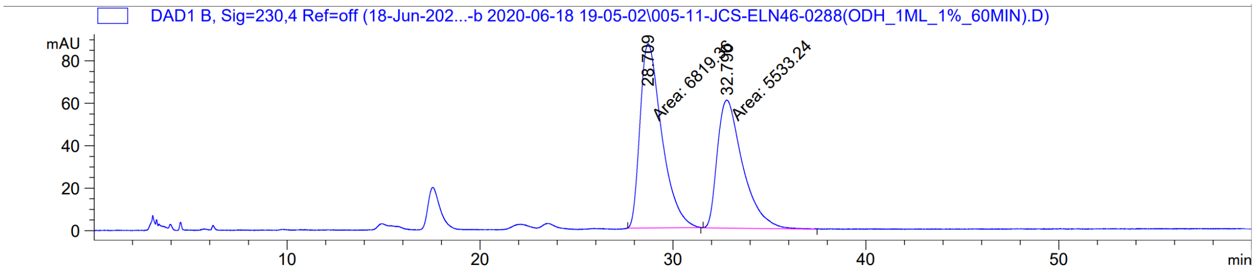

Signal 2: DAD1 B, Sig=230,4 Ref=off

Peak #	RetTime [min]	Type	Width [min]	Area [mAU*s]	Height [mAU]	Area %
1	28.709	MM	1.3082	6819.35693	86.87755	55.2059
2	32.790	MM	1.5261	5533.24121	60.42790	44.7941
Totals :				1.23526e4	147.30545	

S-TCPTAD, DCM, 40% ee

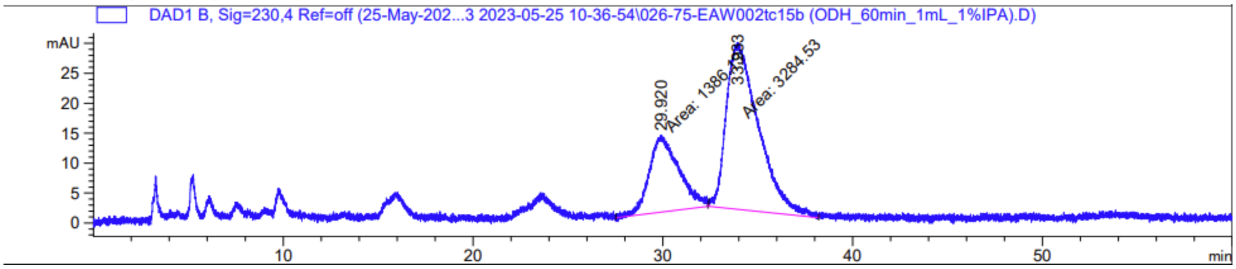

Signal 2: DAD1 B, Sig=230,4 Ref=off

Peak #	RetTime [min]	Type	Width [min]	Area [mAU*s]	Height [mAU]	Area %
1	29.920	MM	1.8069	1386.12671	12.78582	29.6773
2	33.933	MM	1.9641	3284.53369	27.87139	70.3227
Totals :				4670.66040	40.65721	

S-TCPTAD, HFIP, 58% ee

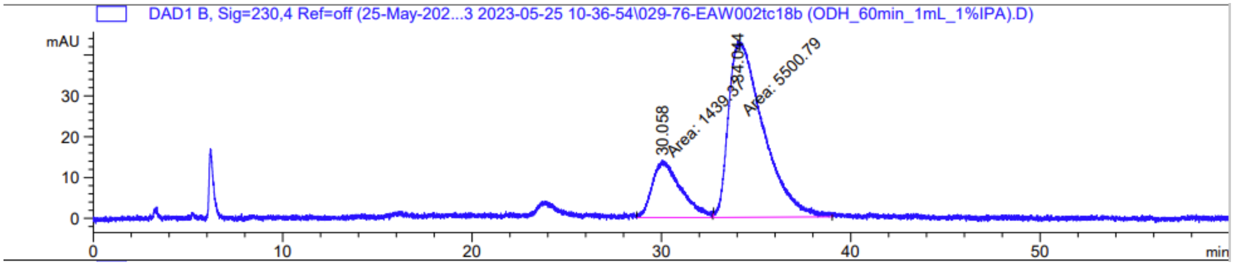

Signal 2: DAD1 B, Sig=230,4 Ref=off

Peak #	RetTime [min]	Type	Width [min]	Area [mAU*s]	Height [mAU]	Area %
1	30.058	MM	1.7083	1439.37280	14.04274	20.7398
2	34.044	MM	2.1234	5500.78613	43.17662	79.2602
Totals :				6940.15894	57.21937	

S-NTTL, DCM, 82% ee

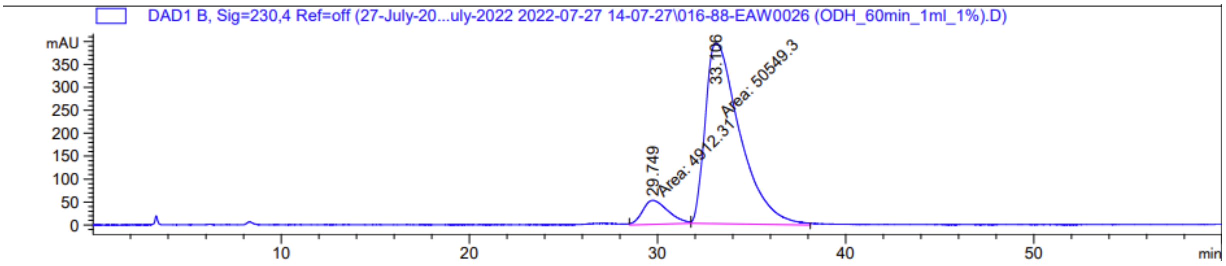

Signal 2: DAD1 B, Sig=230,4 Ref=off

Peak #	RetTime [min]	Type	Width [min]	Area [mAU*s]	Height [mAU]	Area %
1	29.749	MM	1.5662	4912.31494	52.27578	8.8571
2	33.106	MM	2.1403	5.05493e4	393.62222	91.1429
Totals :				5.54616e4	445.89800	

S-NTTL, HFIP, 92% ee

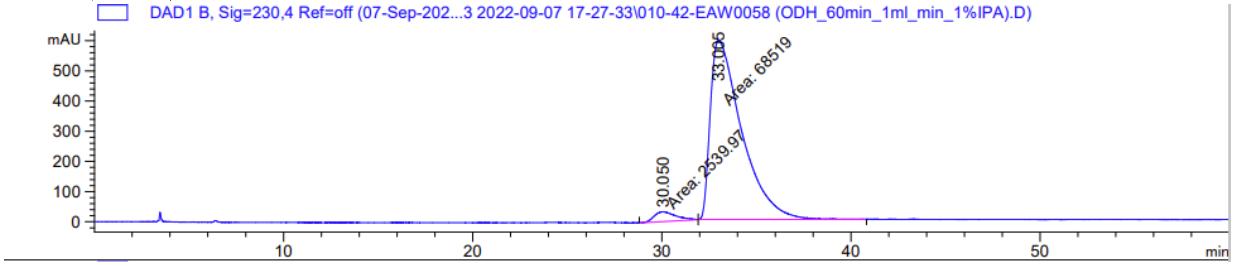

Signal 2: DAD1 B, Sig=230,4 Ref=off

Peak #	RetTime [min]	Type	Width [min]	Area [mAU*s]	Height [mAU]	Area %
1	30.050	MM	1.3065	2539.97046	32.40133	3.5745
2	33.005	MM	1.9193	6.85190e4	595.01489	96.4255
Totals :				7.10590e4	627.41622	

Computational Methods

Classical MD simulations

Classical molecular dynamics (MD) simulations were performed to investigate the solvent effects on the flexibility of the catalysts and their carbene complexes. Initial structures of dirhodium catalysts Rh_2(*S*-NTTL)$_4$ (**Rh-1**), Rh_2(*S*-TCPTAD)$_4$ (**Rh-2**), Rh_2(*S*-TPPTTL)$_4$ (**Rh-3**), and Rh_2(*S*-tetra-4-Br-PPTTL)$_4$ (**Rh-4**) were obtained from crystal structures (CCDC: 749270, 1535046, 1855295, and 2156564, respectively).[4-7] For **Rh-2**, the crystal structure has an *R* absolute configuration (*i.e.*, Rh_2(*R*-TCPTAD)$_4$) and it was mirror inverted to obtain the *S* configuration. The corresponding carbene complexes (**Rh-1a** to **Rh-4a**) were optimized at the B3LYP-D3/6-31G(d)-SDD(Rh) level of theory, where two ester conformations from rotation about the $C_{carbene}$-$C_{carbonyl}$ bond were considered. Classical MD simulations were carried out using the pmemd module[8] of the GPU-accelerated Amber 20 software.[9] Force field parameters for the Rh_2-O_8 core of the catalysts/carbenes and Rh-$C_{carbene}$ bond in the carbene complexes were generated using the MCPB.py module.[10] Force field parameters for HFIP were obtained from the literature[11] and the general Amber force field (gaff2) was used for all other atoms in the catalyst/carbene as well as the DCM solvent.[12,13] Using the Merz-Singh-Kollman scheme,[14,15] RESP (Restrained Electrostatic Potential) charge fitting[16] on the electrostatic potential calculated at the B3LYP-D3/6-31G(d)-SDD(Rh) level of theory was used to derive partial charges. The systems were then placed in periodic boundary conditions with DCM (dichloromethane) or HFIP (1,1,1,3,3,3-hexafluoro-2-propanol) solvents. Long-range electrostatic interactions were calculated using the particle-mesh-Ewald method,[17] with Lennard-Jones and electrostatic interaction cut-offs set to 8.0 Å. The systems first underwent a 30,000-step energy minimization, followed by gradual heating from 0 K to 300 K over 200 ps. This was followed by equilibration using the isothermal–isobaric ensemble (NPT, constant number of particles, pressure, and temperature) for 50 ps. Finally, production simulations were performed for 1,000 ns using a time step of 2 fs, with three independent replicas for each catalyst and six replicas (three with each carbene ester conformation) for each carbene complex.

QM/MM MD simulations

Hybrid Quantum Mechanics/Molecular Mechanics (QM/MM) MD simulations were performed on **Rh-1a** and **Rh-2a** using Grimme's dispersion corrected[18,19] semi-empirical GFN1-xTB (Geometry, Frequency, Noncovalent-Extended Tight Binding) method[20] and the MM driver FIST as implemented in the CP2K v2023.1 package.[21,22] Initial structures of the carbenes complexes as well as their force field parameters were obtained as described in the classical MD section. The solvent systems (DCM and HFIP) were optimized using the LBFGS (Limited Memory Broyden–Fletcher–Goldfarb–Shanno) algorithm[23] while constraining the QM region, followed by NVT (constant number of particles, volume, and temperature) and NPT equilibration. The NVT equilibration was performed for 5 ps and the NPT equilibration was performed for 25 ps at 298 K and 1 bar, using a time step of 0.5 fs for both. Temperature was controlled through CSVR (Canonical Sampling through Velocity Rescaling) thermostat[24] with a time constant of 10 fs, and pressure was controlled using a barostat with a time constant of 100 fs. The averaged cell dimensions from the last 5 ps of the NPT equilibration were used for the subsequent step. From the equilibrated solvent systems, QM/MM MD production simulations were performed for 500 ps. The QM region, which consists of the dirhodium catalysts or the carbene complexes, was treated using the dispersion corrected GFN1-xTB method. A real-space cutoff of 10 Å was used for both Coulomb[25] and exchange

interactions, with the QM cell set to 75% of the full system dimensions. The MM region included the DCM and HFIP solvent molecules. The QM/MM electrostatic coupling was computed using a real-space technique with an Ewald summation using Smooth Particle Mesh Ewald (SPME).[26] The SCF convergence criterion was set to 10^{-6} a.u. with a maximum of 30 SCF cycles and 10 outer SCF iterations. The atomic positions were propagated using the Born−Oppenheimer molecular dynamics (BOMD) scheme[27] with a time step of 0.5 fs in an NVT ensemble at 298 K. The simulation temperature was controlled through a CSVR thermostat with a time constant of 10 fs.

Analysis of MD simulations

All dihedral and distance measurements were performed using the CPPTRAJ tool in AmberTools 20.[28] For the classical MD simulations, MD frames were taken every 0.01 ns for the distance and dihedral measurements, resulting 100,000 frames per replica for the 1,000 ns simulations. For the AIMD simulations, intervals of 0.5 ps were used, corresponding to 1,000 frames per replica of the 500 ps simulations. The ligand–carbene distances [*d*(*Re*) and *d*(*Si*)] were measured from the the distance between the centroids of the aryl group on the ligand and the benzene ring of the carbene donor group based on the definition in Scheme 1. Kernel density estimation (KDE) plots were generated using the Python seaborn library. The KDE plots were used to visualize the distributions of distances and dihedrals observed in the MD trajectories, analogous to histograms. The KDE plots provide smooth representations of the probability density of observed distances and dihedrals. A sharp peak in the KDE plot indicates a high probability density within a small range of distances and dihedrals, suggesting a high level of conformational rigidity of the carboxylate ligand arms.

To understand the flexibility of the dirhodium systems, the four dihedral angles about the $C_{carboxylate}$–C_{α} bonds of each system were monitored throughout the simulations. Each one of the four dihedral angles can adopt one of three orientations: α (60° ± 45°), β (−120° ± 45°), α' (−180° to −165°, −75° to 15°, or 105° to 180°). For each simulation, the dihedral angles were measured in intervals of 0.01 ns and classified into four categories: (i) The $\alpha\alpha\alpha\alpha$ (*i.e.*, all-up or bowl-shape) conformer; (ii) $\alpha\alpha\alpha\alpha'$ in which only one of the ligands partially rotates; (iii) Conformers with more than one partial ligand rotation and no full rotation ($\alpha\alpha'\alpha\alpha'$, $\alpha\alpha\alpha'\alpha'$, $\alpha\alpha'\alpha'\alpha'$, and $\alpha'\alpha'\alpha'\alpha'$); (iv) Conformers with full rotation, where at least one of the ligands fully rotates (*e.g.*, $\alpha\alpha\alpha\beta$, $\alpha\alpha\beta\beta$, and others). For this analysis, conformers are treated as symmetric - for example structures with $\alpha\alpha\beta\beta$ or $\alpha\beta\alpha\beta$ conformation are equivalent to $\beta\beta\alpha\alpha$ or $\beta\alpha\beta\alpha$, respectively.

In addition to the dihedral angles about the $C_{carboxylate}$–C_{α} bonds and the distances between the carboxylate ligand arms to the centroid of the benzene ring of the 4-Br-Ph group on the carbene, we explored a number of other structural features of the Rh-carbene intermediates (**Rh-1a**–**Rh-4a**) as potential metrics in order to quantify the difference of steric environments of the two prochiral π-faces and the flexibility of the carbenoid intermediates. For example, the distances between the carboxylate ligand arms to the carbene carbon from the classical MD simulations (Figure S3) further shed light on the flexibility. Because the steric environment of the two carboxylate ligand arms on the carbene donor side describes which of the two prochiral π-faces will be more open/blocked for the alkene attack, we monitored the octant percent buried volume of the carbene intermediates **Rh-1a** and **Rh-2a** from the QM/MM simulation. The octant percent buried volume is calculated by decomposing the buried volume of the complex into contributions from octants using the MORFEUS package ("octant_analysis" method, see details in the caption of Figure S11). We also monitored the catalyst-solvent and carbene-solvent H-bond interactions from the QM/MM MD simulation to understand the effect of HFIP on the rotation of the ligands (Figure

S10). Root-mean-square fluctuation that describes the movement of the full complex throughout the simulation is shown in Figure S9 was calculated using CPPTRAJ module[28] ("atomicfluct" keyword).

In addition, clustering analysis was carried out using the CPPTRAJ module[28] to identify the most populated structures of each MD simulation. The RMSD (root mean square deviation) values of catalysts/carbene complexes were used as the distance metric for clustering analysis. The resulting most populated structures from K-means clustering are shown in Figure S7.

DFT Methods

Density functional theory (DFT) calculations were carried out using *Gaussian 16* to model the cyclopropanation of alkene **2** with carbene complexes **Rh-1a** and **Rh-2a** to form cyclopropane **3**. Geometries of all stationary points were fully optimized using the dispersion-corrected[18,19] B3LYP-D3(BJ) functional[29,30] with the SDD basis set for rhodium[31,32] and the 6-31G(d) basis set for other atoms. Vibrational frequency calculations at the same level of theory of the geometry optimization were performed to confirm if each structure is a local minimum or a transition state. Quasi-harmonic approximations with Grimme's approach[33] were performed using GoodVibes package using a cut-off vibrational frequency of 100 cm^{-1} in entropy calculations.[34] All Gibbs free energies were calculated at the standard conditions (*i.e.*, 298.15K, 1 mol/L). Single point energy calculations were carried out using the B3LYP-D3(BJ) functional with the SDD basis set for rhodium[31,32] and 6-311+G(d,p) basis set for other atoms. Solvation energy corrections were calculated using the SMD solvation model[35] in single point energy calculations with DCM and HFIP as solvents. According to the recent recommendation from Huber, Truhlar, and Cramer in the SMD18 model,[36] a radius of 2.60 Å was used for the bromine atom and the original SMD radii were used for other elements. Because the solvent parameters for HFIP are not available in *Gaussian 16*, the SMD solvent parameters for HFIP were defined as described in the literature (dielectric constant, ε = 16.7; square of refractive index, n^2 = 1.625625; hydrogen bond acidity, α = 0.77; hydrogen bond basicity, β = 0.10; surface tension at liquid-air interface, γ = 23.23 cal mol^{-1} Å^{-2}; carbon aromaticity, ϕ = 0.00; electronegative halogenicity, ψ = 0.60).[37] For the solvent cluster model, translational entropies in solution were calculated using the free-volume theory proposed by Whitesides[38], implemented in a modified version of the GoodVibes 3.2 package[34], where the molecular volumes (135.6 Å^3 for HFIP, and 101.6 Å^3 for DCM) computed at the single-point level of theory using the "volume=tight" keyword, and the molarities (9.52 mol/L for HFIP, and 15.66 mol/L for DCM), were incorporated into the *thermo.py* script.

Additional computational results

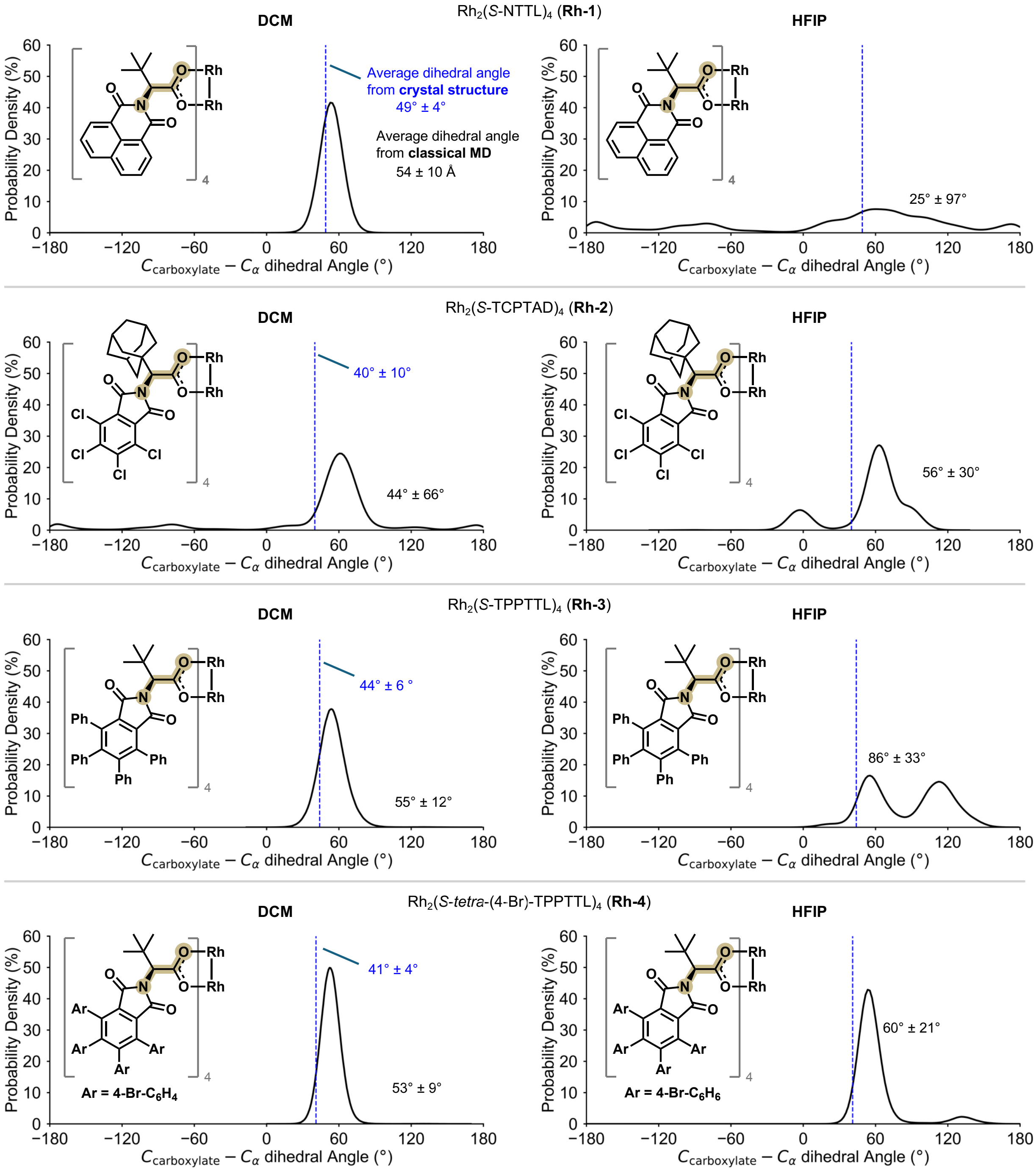

Figure S1. Kernal density estimation (KDE) plots of the dihedral angles about the $C_{carboxylate}$–C_{α} bond of dirhodium catalysts **Rh-1** – **Rh-4** from classical MD simulations. The plots compare conditions in DCM (left) and HFIP (right), with the blue line indicating the average of the four dihedral angles from each catalyst's crystal structure. While HFIP generally increases flexibility compared to DCM, **Rh-4** shows minimal change in flexibility between the two solvents. Data is generated from three 1,000 ns MD replicas.

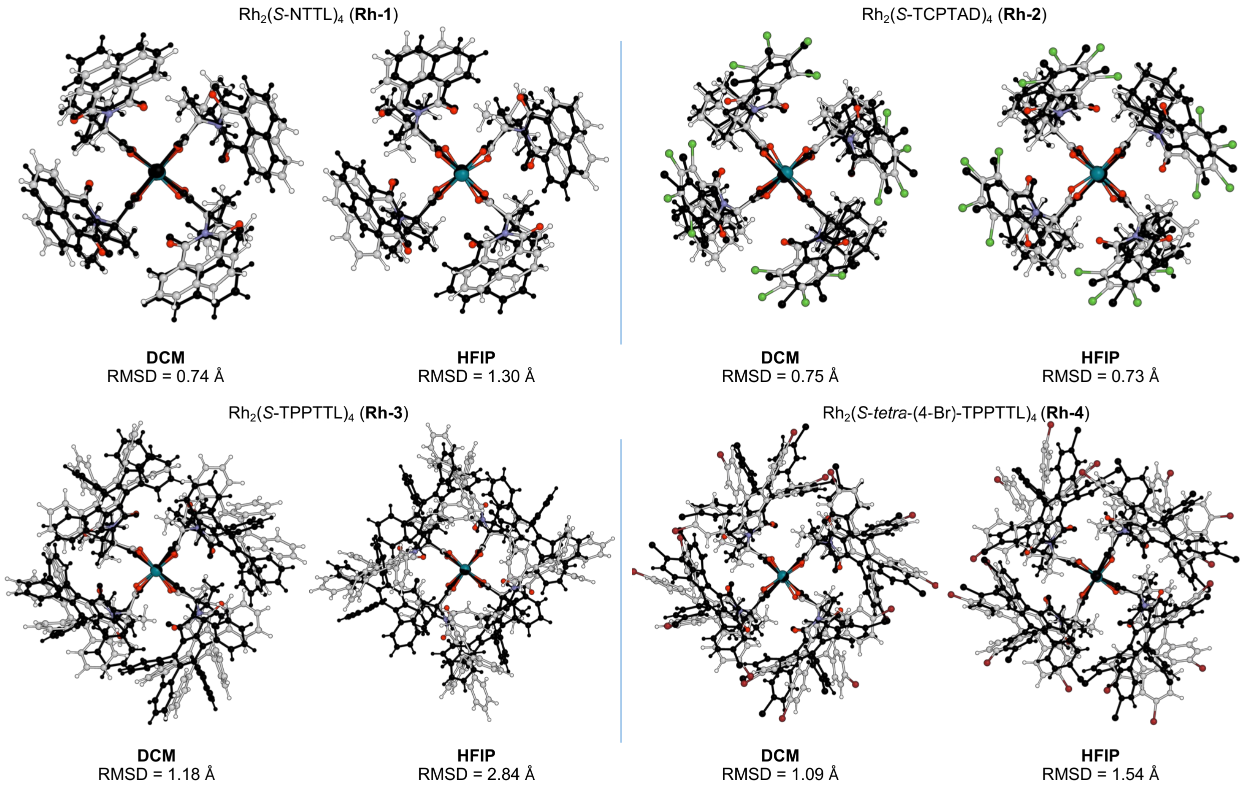

Figure S2. Overlay of the most representative conformation of each catalyst from classical MD simulations (via K-means clustering, gray) with their respective X-ray crystal structures (shown in black). RMSD values are calculated over all non-hydrogen atoms. For the most populated conformer in DCM, there is only a small deviation from the crystal structures of all catalysts (0.74 – 1.18 Å). Larger deviations were observed in HFIP.

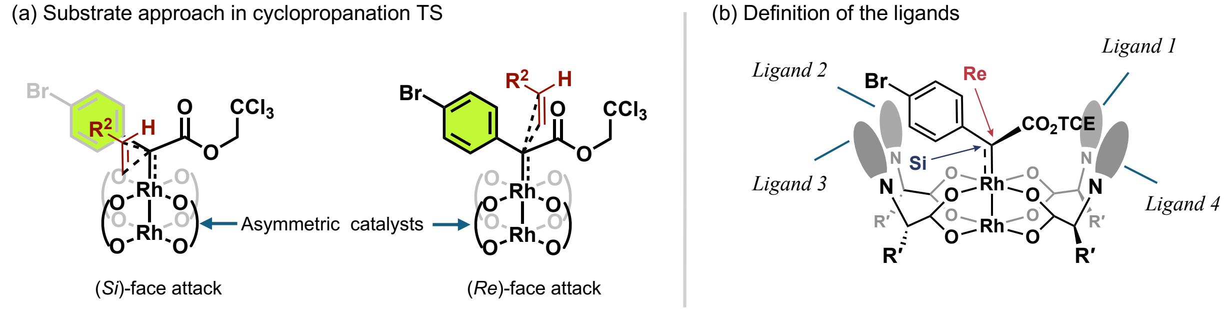

Figure S3. (a) Cyclopropanation transition states, with the substituent (R^2) on the mono-substituted alkene substrate positioned *syn* to the aryl group on the carbene; (b) Definition of the carboxylate ligand arms used in Figures S4–S6 and Tables S2–S9.

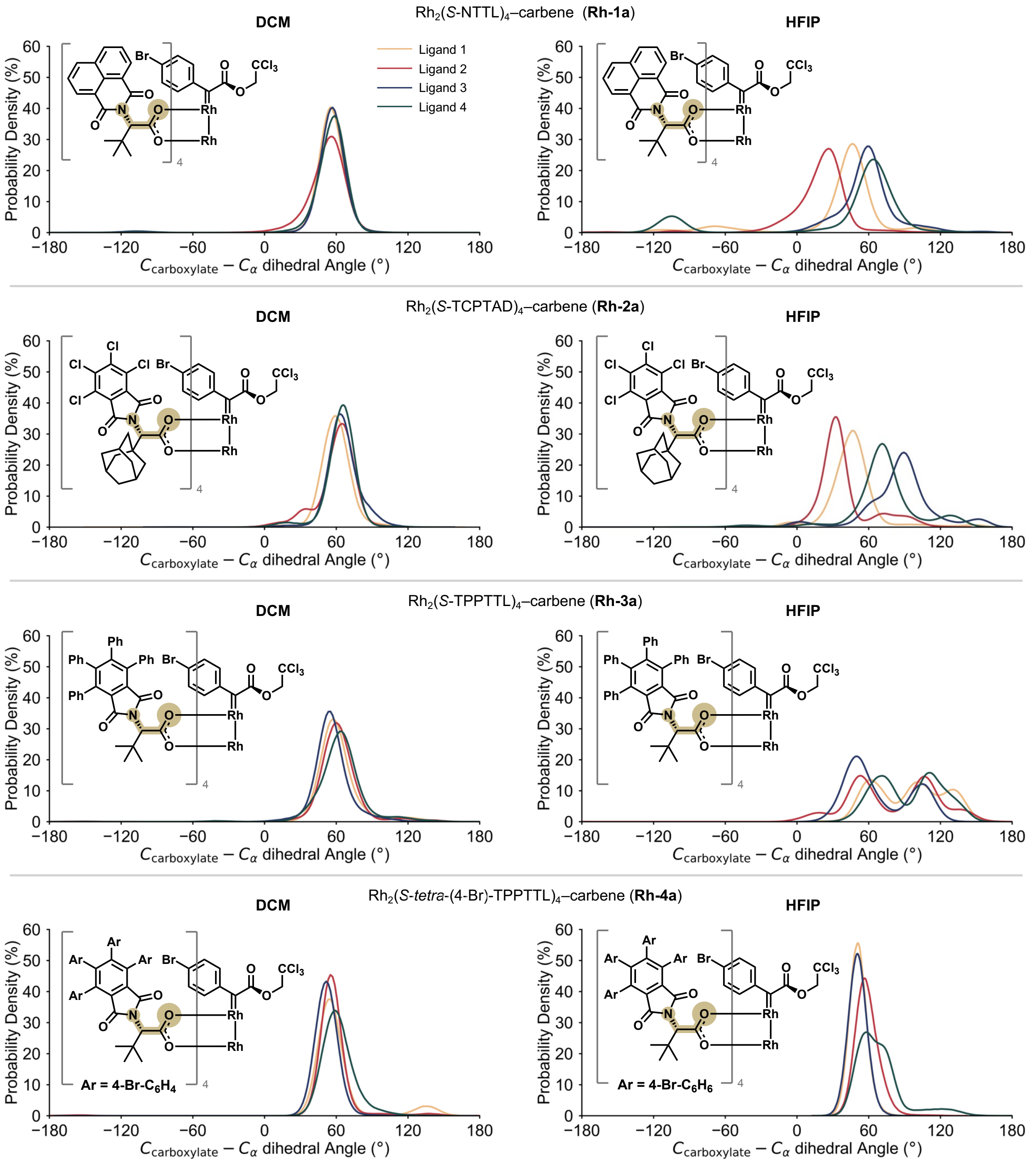

Figure S4. Kernal density estimation (KDE) plots of the dihedral angles about the $C_{carboxylate}$–C_{α} bond of dirhodium carbene complexes **Rh-1a** – **Rh-4a** from classical MD simulations. The plots compare the flexibility in DCM (left) and HFIP (right). Similar to the catalysts, HFIP generally increases the flexibility of the carbene complexes compared to DCM. However, these carbene complexes are more rigid in HFIP than their respective catalysts in the same solvent. Data is generated from six 1,000 ns MD replicas.

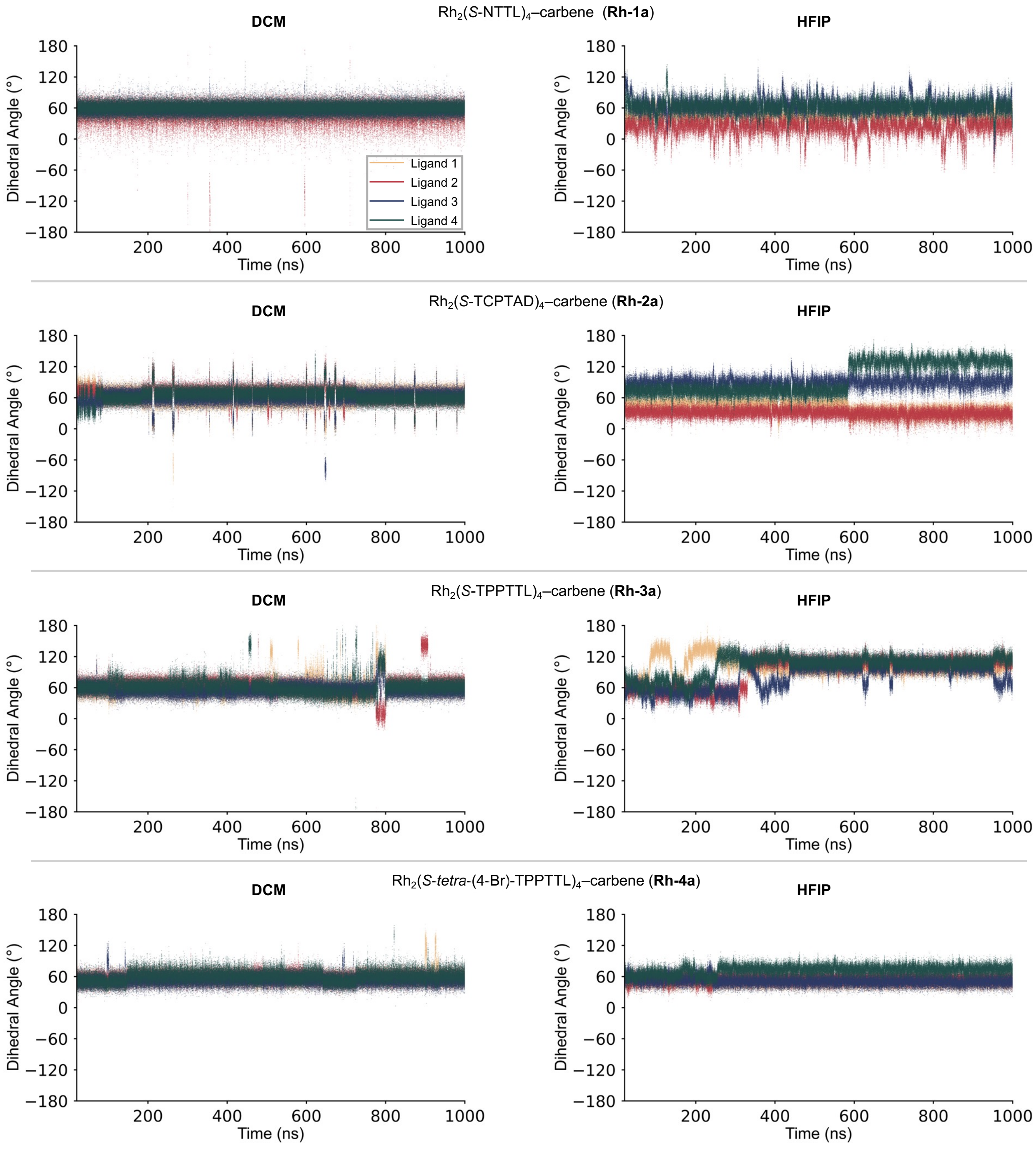

Figure S5. Fluctuation of the dihedral angles about the $C_{carboxylate}$–C_{α} bond as a function of time for one replica in the MD simulations of the carbene complexes, **Rh-1a** – **Rh-4a**, in both DCM and HFIP solvents. See Tables S1–S4 for average values from all replicas.

Table S2: Average dihedral angles about the $C_{carboxylate}$–C_{α} bond (in °) and standard deviation from classical MD simulations of Rh_2(*S*-NTTL)$_4$ carbene complex, **Rh-1a**. "ester1" and "ester2" represent two different initial conformations of the ester group on the carbene.

	DCM				HFIP			
replica	dihedral1	dihedral2	dihedral3	dihedral4	dihedral1	dihedral2	dihedral3	dihedral4
ester1 replica 1	53.3 ± 9.1	49.9 ± 19.3	57.8 ± 10.2	60.2 ± 9.3	45.8 ± 10.0	21.9 ± 15.1	65.3 ± 13.8	62.0 ± 11.8
ester1 replica 2	53.3 ± 9.1	50.9 ± 18.1	57.5 ± 10.1	60.2 ± 9.2	44.9 ± 10.6	23.5 ± 14.9	63.8 ± 11.6	64.3 ± 16.1
ester1 replica 3	53.4 ± 9.1	50.7 ± 18.1	57.4 ± 10.1	60.2 ± 9.3	45.6 ± 10.5	24.7 ± 17.9	64.8 ± 13.7	62.6 ± 13.0
ester2 replica 1	59.0 ± 11.7	54.2 ± 11.9	55.7 ± 19.8	53.0 ± 16.2	65.5 ± 34.4	24.2 ± 24.5	49.1 ± 32.7	58.4 ± 86.8
ester2 replica 2	59.4 ± 11.8	53.8 ± 14.5	55.1 ± 27.7	51.2 ± 17.5	-55.9 ± 57.1	-3.5 ± 43.3	40.1 ± 22.2	-98.3 ± 53.4
ester2 replica 3	59.5 ± 12.0	53.5 ± 13.2	55.5 ± 21.6	51.8 ± 12.4	41.4 ± 32.8	20.8 ± 17.6	64.6 ± 25.7	65.2 ± 23.8
all replicas	56.3 ± 10.5	52.2 ± 15.9	56.5 ± 16.6	56.1 ± 12.5	31.2 ± 26.4	18.6 ± 22.2	57.9 ± 20.0	35.7 ± 34.2

Table S3: Average dihedral angles about the $C_{carboxylate}$–C_{α} bond (in °) and standard deviation from classical MD simulations of Rh_2(*S*-TCPTAD)$_4$ carbene complex, **Rh-2a**. "ester1" and "ester2" represent two different initial conformations of the ester group on the carbene.

	DCM				HFIP			
replica	dihedral1	dihedral2	dihedral3	dihedral4	dihedral1	dihedral2	dihedral3	dihedral4
ester1 replica 1	57.8 ± 14.7	66.9 ± 13.4	60.2 ± 14.1	65.8 ± 12.5	38.9 ± 11.7	29.6 ± 8.5	89.0 ± 10.2	101.5 ± 31.0
ester1 replica 2	58.8 ± 12.3	53.8 ± 16.3	70.7 ± 13.3	64.8 ± 11.8	55.3 ± 26.2	31.3 ± 8.1	83.1 ± 23.9	73.7 ± 15.2
ester1 replica 3	56.5 ± 12.4	55.5 ± 17.3	69.0 ± 15.3	67.0 ± 11.3	36.7 ± 27.6	71.7 ± 30.6	53.8 ± 36.3	80.2 ± 20.8
ester2 replica 1	61.4 ± 10.5	63.4 ± 12.5	64.5 ± 10.4	62.8 ± 12.1	44.9 ± 10.9	32.1 ± 10.2	106.4 ± 29.9	74.8 ± 15.9
ester2 replica 2	59.8 ± 9.9	65.1 ± 10.1	62.9 ± 10.2	64.7 ± 9.4	44.5 ± 11.8	29.7 ± 11.8	107.1 ± 28.5	68.6 ± 28.6
ester2 replica 3	66.0 ± 15.1	54.3 ± 21.6	69.1 ± 16.9	54.8 ± 19.4	57.4 ± 32.9	29.4 ± 11.4	88.5 ± 20.1	63.0 ± 39.1
all replicas	60.1 ± 12.5	59.8 ± 15.2	66.1 ± 13.4	63.3 ± 12.8	46.3 ± 20.2	37.3 ± 13.4	88.0 ± 24.8	77.0 ± 25.1

Table S4: Average dihedral angles about the $C_{carboxylate}$–C_{α} bond (in °) and standard deviation from classical MD simulations of Rh_2(*S*-TPPTTL)$_4$ carbene complex, **Rh-3a**. "ester1" and "ester2" represent two different initial conformations of the ester group on the carbene.

	DCM				HFIP			
replica	dihedral1	dihedral2	dihedral3	dihedral4	dihedral1	dihedral2	dihedral3	dihedral4
ester1 replica 1	59.7 ± 16.5	62.2 ± 17.4	55.6 ± 10.7	63.0 ± 17.0	103.1 ± 13.3	99.2 ± 23.4	88.0 ± 22.1	109.4 ± 13.5
ester1 replica 2	60.2 ± 9.7	60.7 ± 9.1	55.8 ± 10.0	63.4 ± 10.1	93.8 ± 16.1	94.9 ± 28.1	82.5 ± 24.8	115.1 ± 11.6
ester1 replica 3	55.6 ± 12.4	58.8 ± 9.7	53.4 ± 15.4	64.7 ± 15.3	132.2 ± 9.8	79.5 ± 23.6	41.5 ± 9.7	70.2 ± 11.4
ester2 replica 1	61.1 ± 13.1	70.2 ± 19.3	53.6 ± 13.1	59.0 ± 20.3	94.0 ± 25.3	105.2 ± 19.5	98.4 ± 21.1	95.9 ± 20.3
ester2 replica 2	68.5 ± 28.7	65.8 ± 22.4	58.8 ± 19.2	72.9 ± 23.9	69.8 ± 18.3	30.6 ± 20.2	79.6 ± 27.0	124.9 ± 13.2
ester2 replica 3	63.7 ± 14.9	61.0 ± 23.7	61.9 ± 13.7	64.8 ± 19.5	93.2 ± 37.6	95.1 ± 43.9	52.5 ± 11.8	70.8 ± 11.8
all replicas	61.5 ± 15.9	63.1 ± 16.9	56.5 ± 13.7	64.6 ± 17.7	97.7 ± 20.1	84.1 ± 26.5	73.8 ± 19.4	97.7 ± 13.6

Table S5: Average dihedral angles about the $C_{carboxylate}$–C_{α} bond (in °) and standard deviation from classical MD simulations of Rh_2(*S*-*tetra*-(4-Br)TPPTTL)$_4$ carbene complex, **Rh-4a**. "ester1" and "ester2" represent two different initial conformations of the ester group on the carbene.

	DCM				HFIP			
replica	dihedral1	dihedral2	dihedral3	dihedral4	dihedral1	dihedral2	dihedral3	dihedral4
ester1 replica 1	57.9 ± 10.3	57.8 ± 8.7	53.2 ± 9.0	61.4 ± 11.3	49.4 ± 6.3	54.9 ± 7.1	50.9 ± 8.0	73.8 ± 8.3
ester1 replica 2	53.5 ± 8.5	55.5 ± 7.7	49.8 ± 10.3	58.8 ± 14.4	49.6 ± 6.2	54.1 ± 6.9	50.1 ± 7.7	62.9 ± 10.4
ester1 replica 3	54.5 ± 11.0	55.4 ± 8.1	51.0 ± 11.2	57.7 ± 14.5	49.6 ± 6.2	55.1 ± 6.9	49.6 ± 7.7	96.0 ± 26.7
ester2 replica 1	78.8 ± 38.3	60.6 ± 24.3	53.1 ± 9.2	64.7 ± 14.1	51.4 ± 7.2	60.7 ± 7.8	52.9 ± 7.8	62.7 ± 11.8
ester2 replica 2	64.3 ± 36.8	58.0 ± 17.8	49.9 ± 9.0	71.6 ± 15.8	51.4 ± 8.1	64.6 ± 10.3	50.2 ± 7.7	70.2 ± 25.1
ester2 replica 3	57.3 ± 16.6	59.4 ± 18.1	53.5 ± 9.2	62.5 ± 11.8	54.3 ± 8.8	59.2 ± 12.4	50.3 ± 7.6	60.7 ± 10.3
all replicas	61.1 ± 20.3	57.8 ± 14.1	51.8 ± 9.7	62.8 ± 13.7	51.0 ± 7.1	58.1 ± 8.6	50.7 ± 7.8	71.1 ± 15.4

Table S6: Average dihedral angles about the $C_{carboxylate}$–C_{α} bond (in °) and standard deviation from classical MD simulations of Rh_2(*S*-NTTL)$_4$ catalyst, **Rh-1**.

	DCM				HFIP			
replica	dihedral1	dihedral2	dihedral3	dihedral4	dihedral1	dihedral2	dihedral3	dihedral4
replica 1	53.6 ± 10.4	53.8 ± 9.9	53.7 ± 9.7	53.9 ± 9.8	109.8 ± 39.8	96.6 ± 35.4	170.5 ± 61.4	153.0 ± 68.9
replica 2	53.7 ± 10.5	53.9 ± 9.8	53.7 ± 9.8	54.0 ± 9.8	-167.0 ± 52.9	131.5 ± 87.5	-66.3 ± 72.9	81.7 ± 47.6
replica 3	53.7 ± 9.8	53.6 ± 9.8	53.7 ± 9.7	53.9 ± 9.8	127.8 ± 103.9	42.7 ± 27.3	-28.0 ± 86.2	70.0 ± 29.3
all replicas	53.7 ± 10.2	53.8 ± 9.8	53.7 ± 9.7	53.9 ± 9.8	23.5 ± 65.5	90.3 ± 50.1	25.4 ± 73.5	101.6 ± 48.6

Table S7: Average dihedral angles about the $C_{carboxylate}$–C_{α} bond (in °) and standard deviation from classical MD simulations of Rh_2(*S*-TCPTAD)$_4$ catalyst, **Rh-2**.

	DCM				HFIP			
replica	dihedral1	dihedral2	dihedral3	dihedral4	dihedral1	dihedral2	dihedral3	dihedral4
replica 1	61.4 ± 13.9	59.4 ± 11.6	60.9 ± 13.7	59.1 ± 11.7	81.6 ± 16.9	10.0 ± 28.0	83.1 ± 14.3	9.3 ± 30.0
replica 2	-167.0 ± 52.9	131.5 ± 87.5	-66.3 ± 72.9	81.7 ± 47.6	75.8 ± 16.0	32.2 ± 33.2	75.7 ± 14.4	32.3 ± 33.7
replica 3	62.1 ± 12.5	58.9 ± 11.1	61.8 ± 12.5	58.9 ± 11.2	63.1 ± 10.6	58.7 ± 11.6	66.2 ± 10.9	60.6 ± 9.5
all replicas	-14.5 ± 26.4	83.3 ± 36.7	18.8 ± 33.0	66.6 ± 23.5	73.5 ± 14.5	33.6 ± 24.3	75.0 ± 13.2	34.1 ± 24.4

Table S8: Average dihedral angles about the $C_{carboxylate}$–C_{α} bond (in °) and standard deviation from classical MD simulations of Rh_2(*S*-TPPTTL)$_4$ catalyst, **Rh-3**.

	DCM				HFIP			
replica	dihedral1	dihedral2	dihedral3	dihedral4	dihedral1	dihedral2	dihedral3	dihedral4
replica 1	53.2 ± 10.1	55.7 ± 9.6	54.3 ± 9.9	57.3 ± 10.6	85.6 ± 36.4	104.0 ± 14.1	106.3 ± 13.2	106.9 ± 12.2
replica 2	46.3 ± 11.5	60.3 ± 11.5	47.9 ± 12.2	62.6 ± 12.6	113.4 ± 21.2	104.0 ± 28.1	67.2 ± 16.3	120.6 ± 13.1
replica 3	51.1 ± 13.3	54.5 ± 10.2	52.7 ± 10.5	56.8 ± 12.5	120.2 ± 31.7	56.2 ± 15.6	48.2 ± 9.6	61.9 ± 11.4
all replicas	50.2 ± 11.6	56.8 ± 10.4	51.6 ± 10.9	58.9 ± 11.9	106.4 ± 29.8	88.1 ± 19.3	73.9 ± 13.0	96.5 ± 12.2

Table S9: Average dihedral angles about the $C_{carboxylate}$–C_{α} bond (in °) and standard deviation from classical MD simulations of Rh_2(*S*-*tetra*-(4-Br)TPPTTL)$_4$ catalyst, **Rh-4**.

	DCM				HFIP			
replica	dihedral1	dihedral2	dihedral3	dihedral4	dihedral1	dihedral2	dihedral3	dihedral4
replica 1	52.3 ± 8.4	53.6 ± 8.1	53.3 ± 8.2	53.7 ± 8.4	51.4 ± 7.8	54.9 ± 8.2	53.0 ± 8.8	81.0 ± 31.5
replica 2	53.8 ± 12.3	53.1 ± 7.9	53.8 ± 8.2	53.5 ± 8.3	55.8 ± 8.9	55.0 ± 7.9	58.1 ± 9.4	106.2 ± 38.2
replica 3	51.4 ± 8.4	53.9 ± 8.2	52.1 ± 8.3	53.4 ± 8.3	49.6 ± 7.6	53.5 ± 7.3	54.8 ± 9.0	59.1 ± 11.6
all replicas	52.5 ± 9.7	53.5 ± 8.1	53.1 ± 8.2	53.5 ± 8.3	52.3 ± 8.1	54.5 ± 7.8	55.3 ± 9.1	82.1 ± 27.1

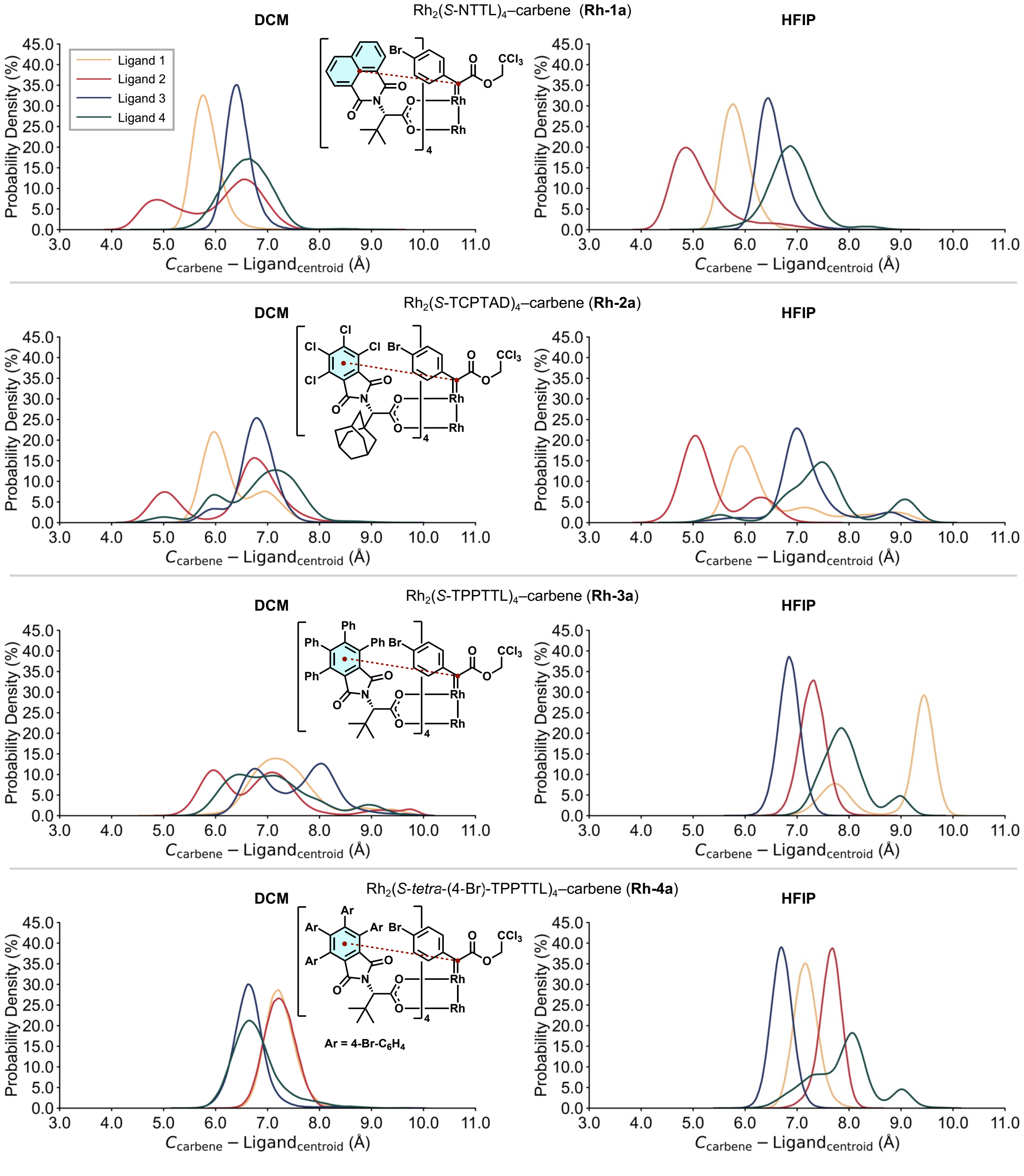

Figure S6: Kernal density estimation (KDE) plots of the $C_{carbene}$–$ligand_{centroid}$ distances of dirhodium carbene complexes **Rh-1a** – **Rh-4a** from classical MD simulations. Data is generated from six 1,000 ns MD replicas.

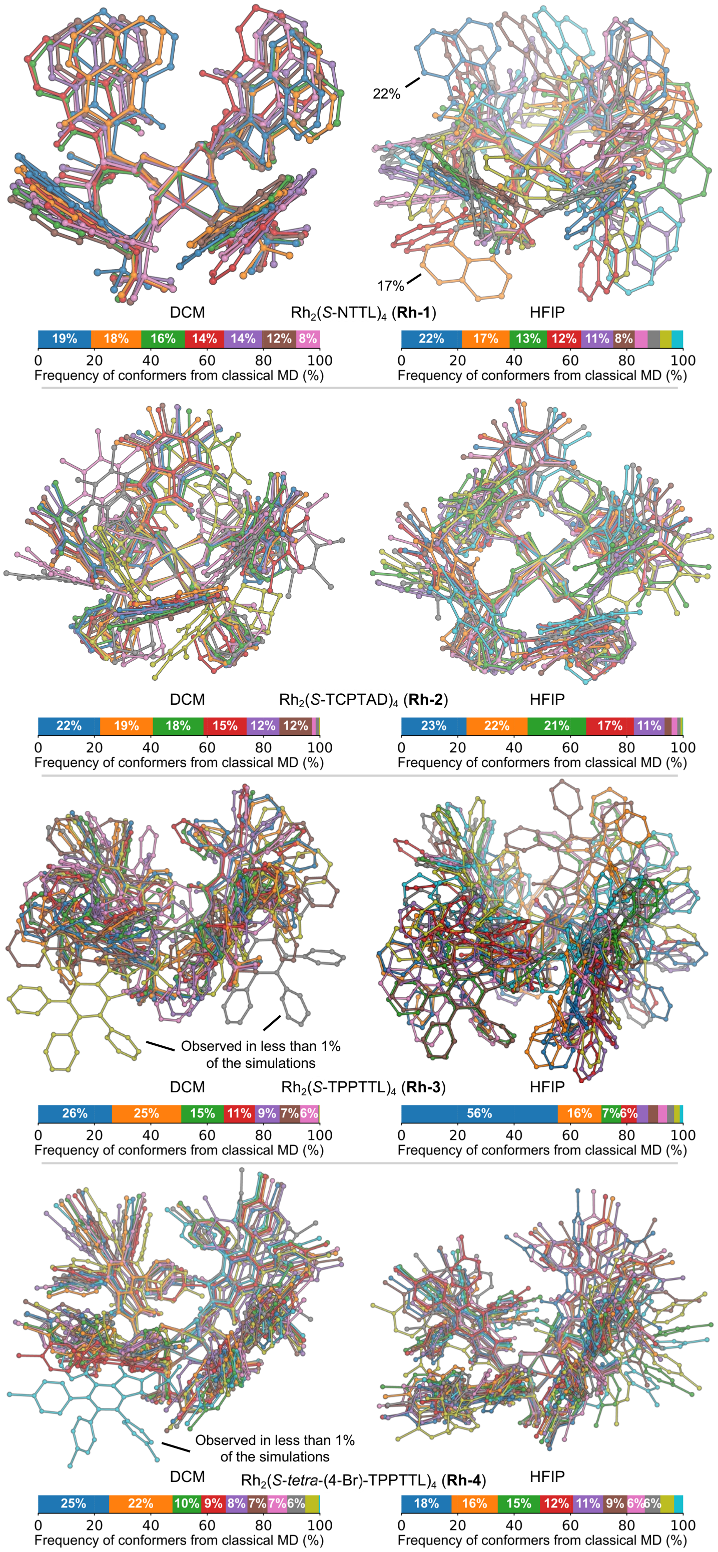

Figure S7: K-means clustering of catalysts **Rh-1** – **Rh-4** from classical MD simulations in DCM and HFIP. In DCM, the catalysts exhibit the all-up bowl-shape conformer that is observed in the crystal structures, whereas the catalysts in HFIP exhibit the all-up conformers as well as conformers with partial or full rotation of the ligands.

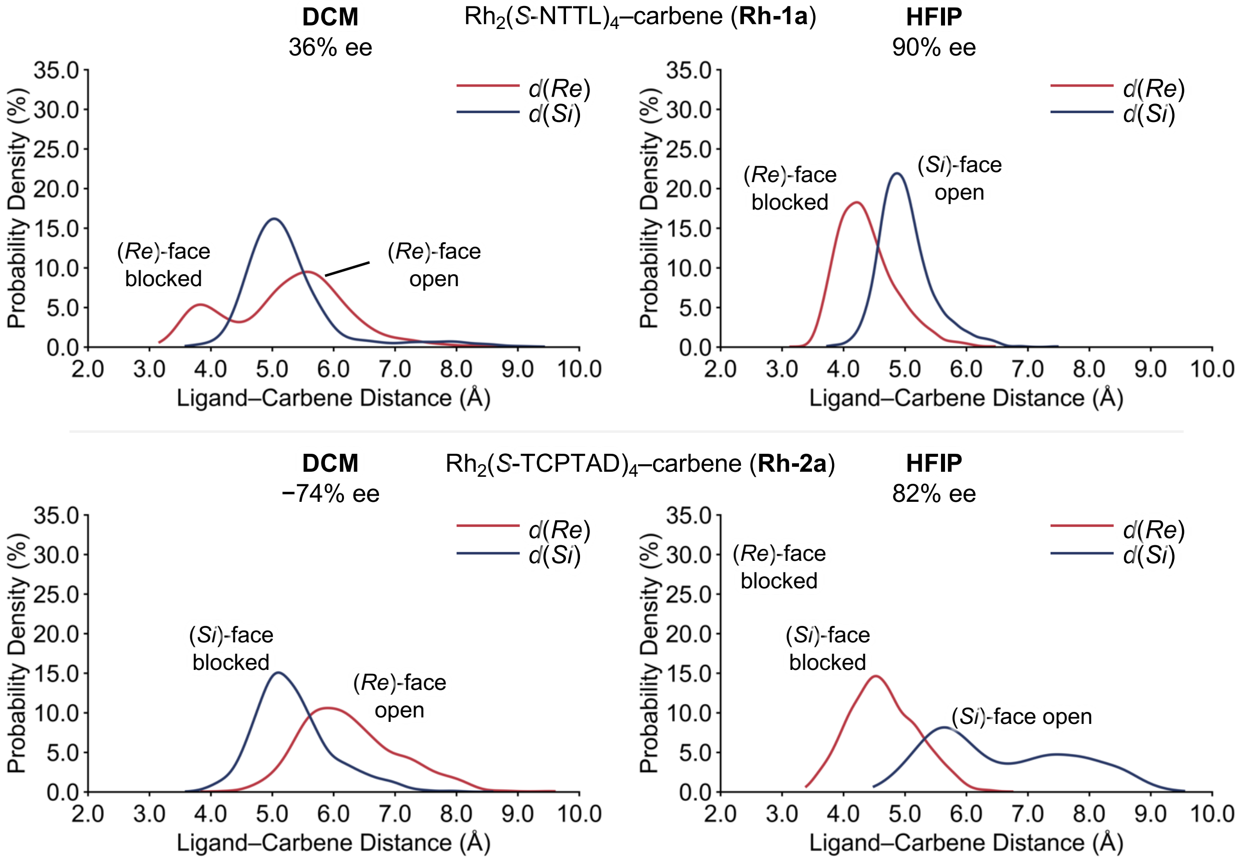

Figure S8: Kernal density estimation (KDE) plots of the distances between the centroids of the aryl group on the ligand and the benzene ring of the carbene donor group (see Scheme 1) of the carbene complexes **Rh-1a** and **Rh-2a** from QM/MM MD simulations in DCM and HFIP. Data is generated from two 500 ps MD of both ester conformations.

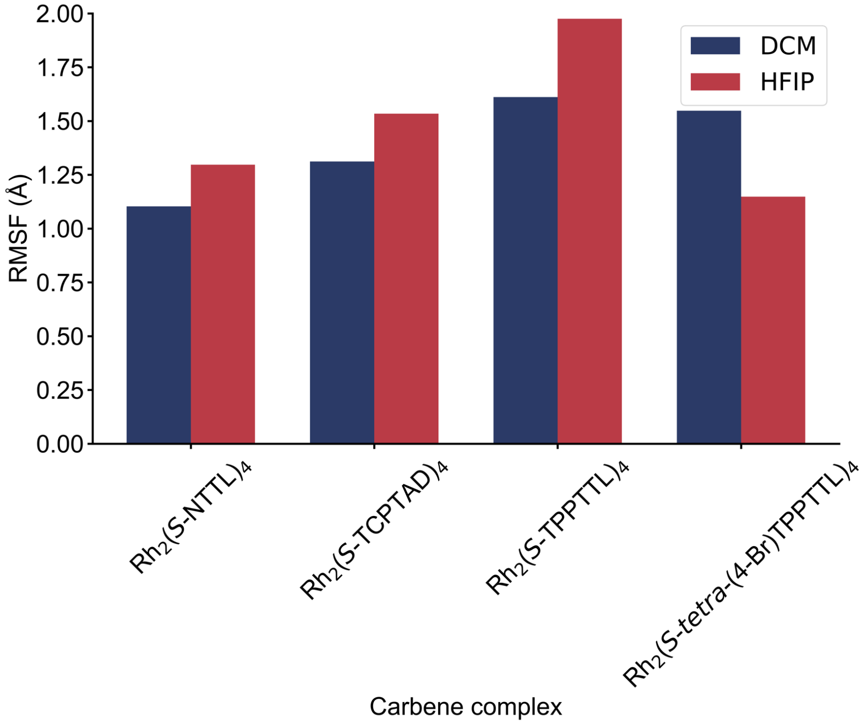

Figure S9: Relative root-mean-square fluctuation (RMSF) of the carbene complexes **Rh-1a** – **Rh-4a** from classical MD simulations in DCM and HFIP. Larger numbers indicate more fluctuation of the overall complex. In all complexes, the overall movement in of the carbenes is larger in HFIP than DCM, except for **Rh-4a**.

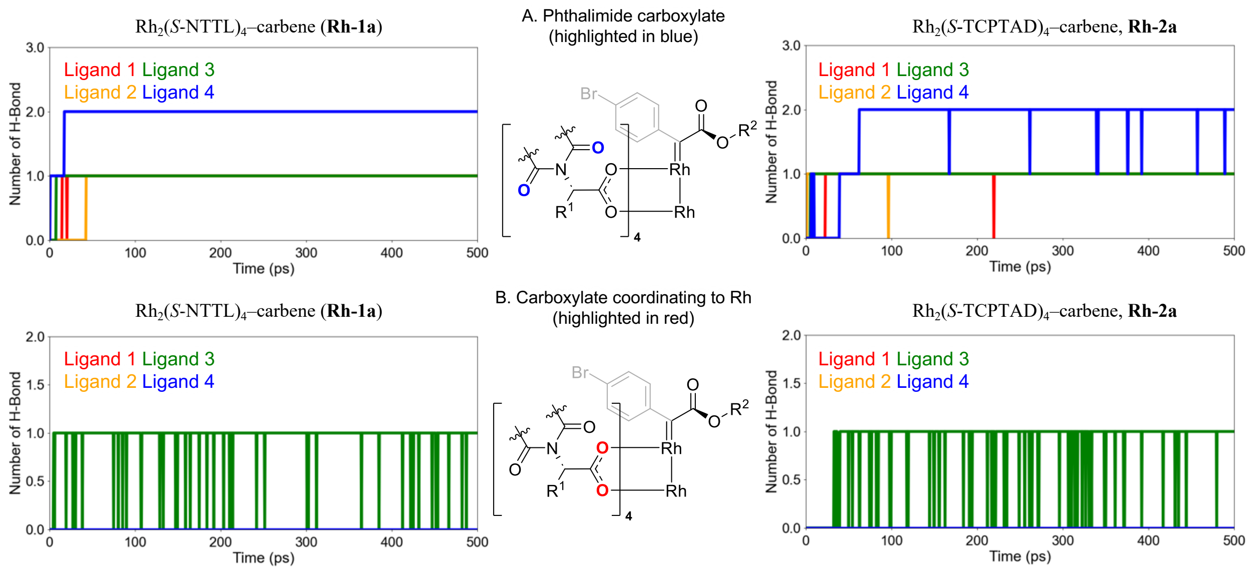

Figure S10: Number of hydrogen bonds with solvent molecules along the QM/MM MD trajectory for the carbene complexes **Rh-1a** and **Rh-2a** in HFIP solvent. The simulation show that most arms can form at least one hydrogen bond with HFIP solvent molecules. The hydrogen bonds between ligand arms and HFIP solvent molecules compete with the interactions between adjacent arms of carboxylate ligands leading to carboxylate rotation.

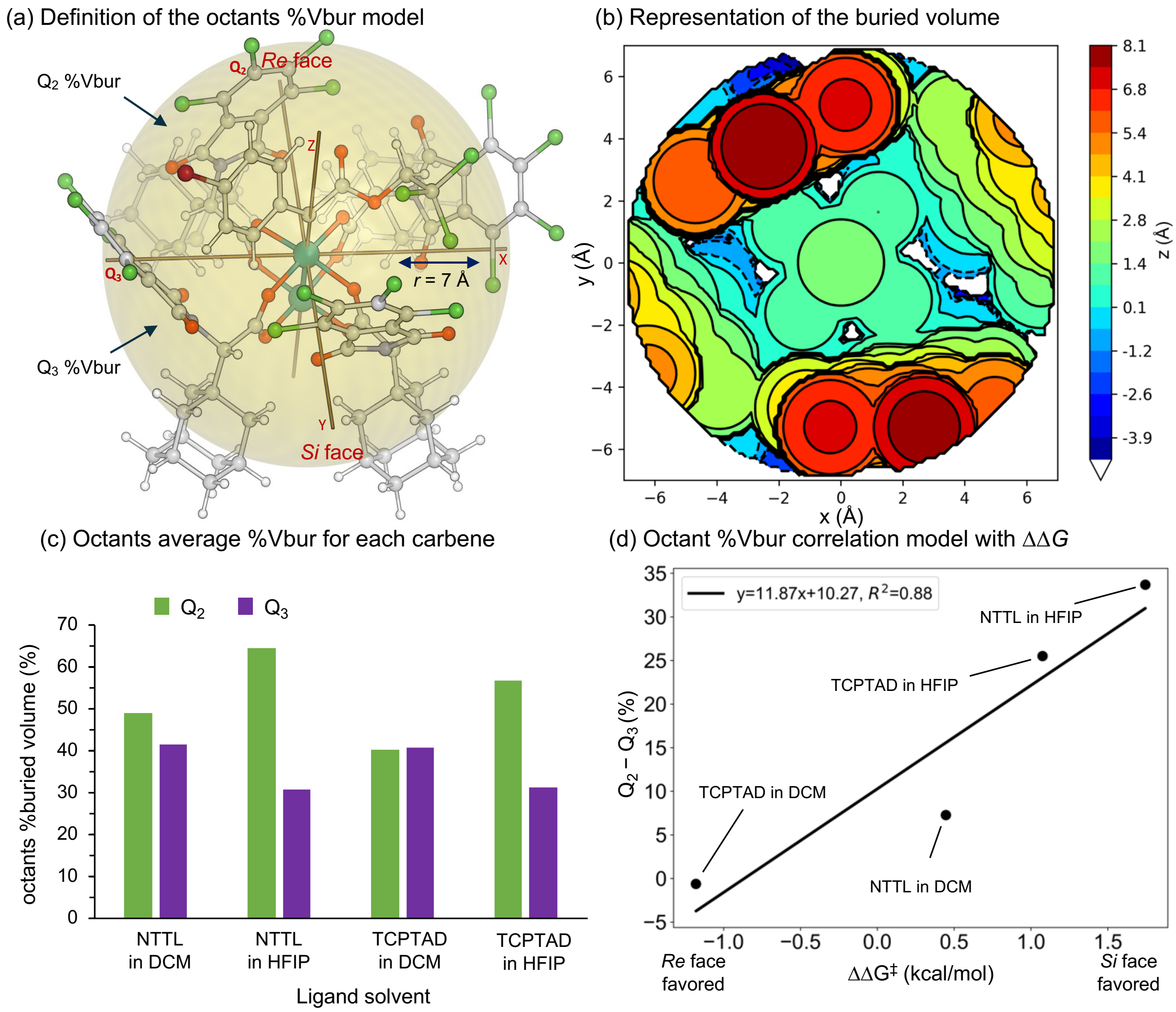

Figure S11: Monitoring octant percent buried volume from QM/MM MD simulations of the carbene complexes **Rh-1a** and **Rh-2a** in DCM and HFIP solvents. (a) Definition of the octants of interest, which are the two octants in the same plane of the aryl group in for the carbene complex. The origin of the 7 Å sphere is centered at the rhodium atom bonded to the carbene, with the positive z axis going through the $C_{carbene}$ atom, and the xz-plane defined as the center between the two oxygens bonded to Rh in Q_1 and Q_4. (b) Plot of the buried volume for a single snapshot using the mentioned definition. (c) The average octant percent buried volume throughout the simulations. (d) A correlation plot between the difference in the buried volume between the octants leading to the *Si* and *Re* face of the carbene ($Q_2 - Q_3$) as function of the experimental $\Delta\Delta G^{\ddagger}$ derived from observed *ee* values.

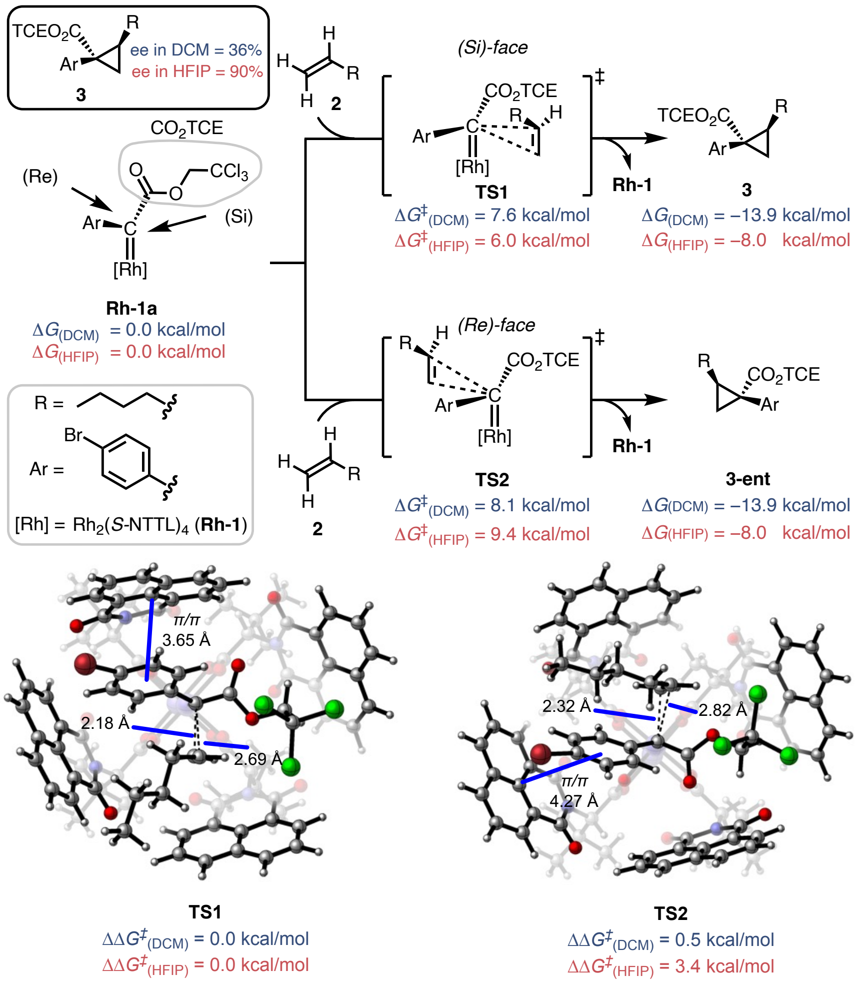

Figure S12: Density functional theory (DFT) calculations of the alkene cycloaddition step with Rh_2(*S*-NTTL)$_4$–carbene complex, **Rh-1a**, at the B3LYP-D3(BJ)/6-311+G(d,p)–SDD(Rh)/SMD(DCM or HFIP)//B3LYP-D3(BJ)/6-31G(d)–SDD(Rh) level of theory. ΔG_{DCM} corresponds to the Gibbs free energy using DCM as implicit solvent, and ΔG_{HFIP} corresponds to the Gibbs free energy using HFIP as implicit solvent. The DFT calculations correctly predicted the formation of the major enantiomer in both solvents via **TS1**, which has stronger π/π interactions between the ligand and the aryl group on the carbene. Additionally, using the SMD implicit solvation model, the computed $\Delta\Delta G^{\ddagger}$ is much larger in HFIP when compared to DCM, at 3.4 and 0.5 kcal/mol, respectively, which is also consistent with the enhanced enantioselectivity observed with Rh_2(*S*-NTTL)$_4$ when switching between DCM and HFIP solvents. All energies are with respect to **Rh-1a** in either DCM (blue) or HFIP (red) as implicit solvent.

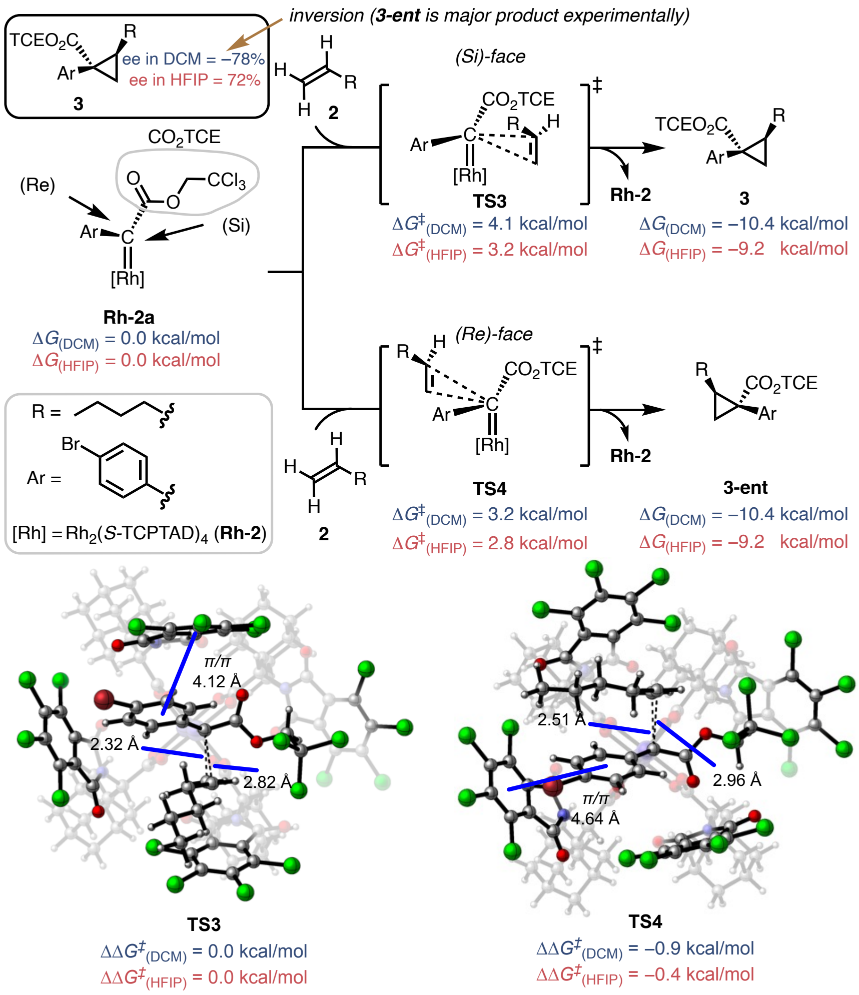

Figure S13: Density functional theory (DFT) calculations of the alkene cycloaddition step with Rh_2(*S*-TCPTAD)$_4$–carbene complex, **Rh-2a**, at the B3LYP-D3(BJ)/6-311+G(d,p)–SDD(Rh)/SMD(DCM or HFIP)//B3LYP-D3(BJ)/6-31G(d)–SDD(Rh) level of theory. ΔG_{DCM} corresponds to the Gibbs free energy using DCM as the implicit solvent, and ΔG_{HFIP} corresponds to the Gibbs free energy using HFIP as the implicit solvent. The DFT calculations correctly predict the formation of the major enantiomer in DCM via **TS4**, and is qualitatively consistent with observed ee% experimentally (moderate ee 78% formation of **3-ent** with $\Delta\Delta G^{\ddagger}$ = 0.9 kcal/mol favoring **TS4**). However, the calculations using HFIP as implicit solvent failed at predicting the formation of the major enantiomer, possibly due to the use of implicit solvent models or not considering adequate conformational space (Boltzmann-contributions). All Gibbs free energies are with respect to **Rh-2a** in either DCM (blue) or HFIP (red) as the implicit solvent.

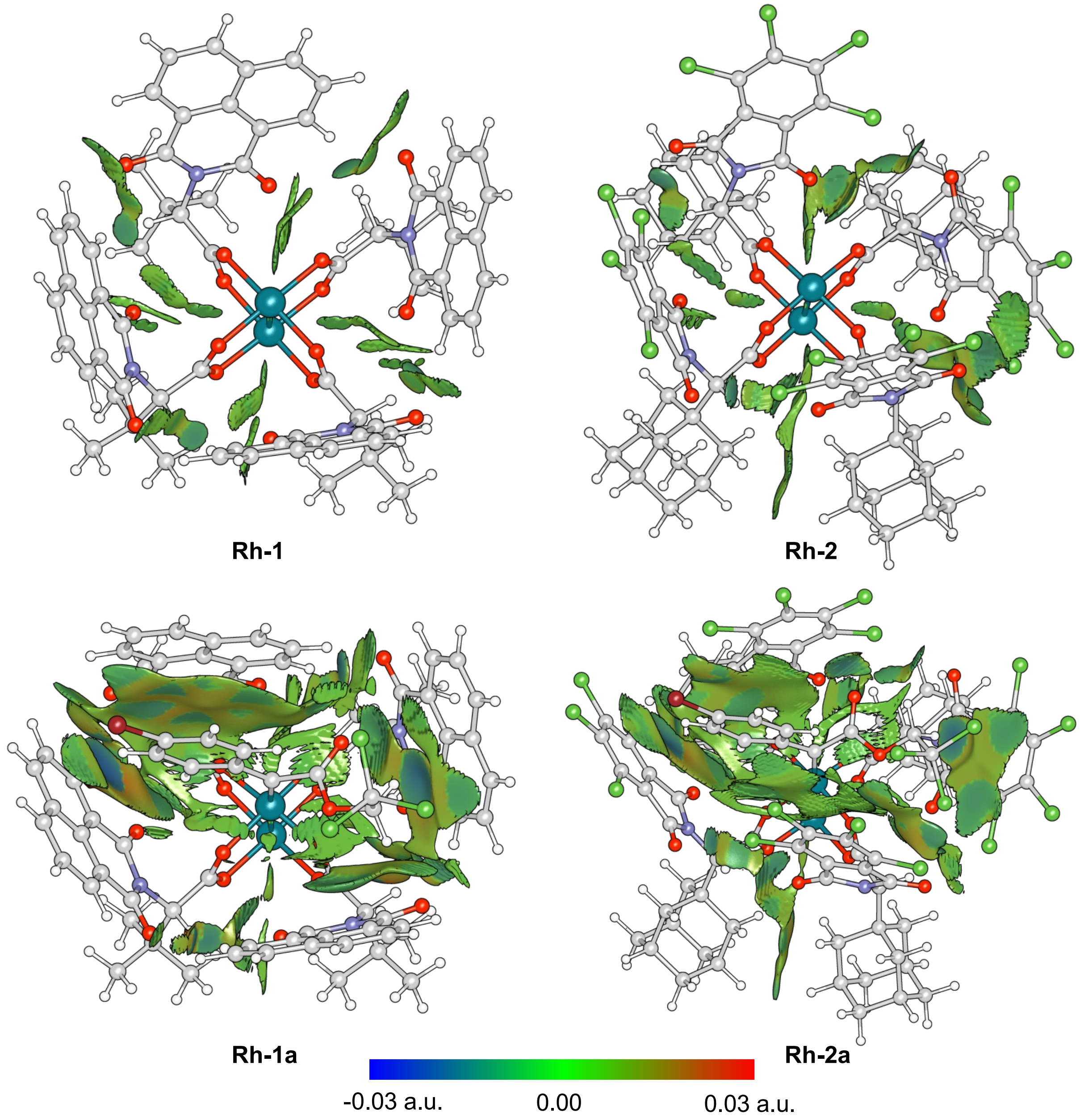

Figure S14: Noncovalent interaction (NCI) analysis of intramolecular interactions of catalysts **Rh-1** and **Rh-2** and their carbene complexes **Rh-1a** and **Rh-2a**. The reduced density gradient (RDG) isosurface of an isovalue of 0.5 was colored on a blue-green-red scale according to values of $sign(\lambda_2)\rho$, ranging from −0.03 to +0.03 a.u. Blue, green, and red indicate strong attractive interactions, weak interactions, and strong repulsions, respectively. The NCIPlot was generated using NCIPLOT 4.2 and PyMOL. The PROMOLECULAR mode was used. Only interfragment interactions (between different ligands or between ligand and carbene) were considered and interactions with the Rh_2-O_8 core were not considered. The addition of the carbene increases the non-covalent interaction significantly when compared to the catalysts, which makes them less susceptible to full ligand arm rotations and H-bond interactions with HFIP when compared to the catalyst themselves.

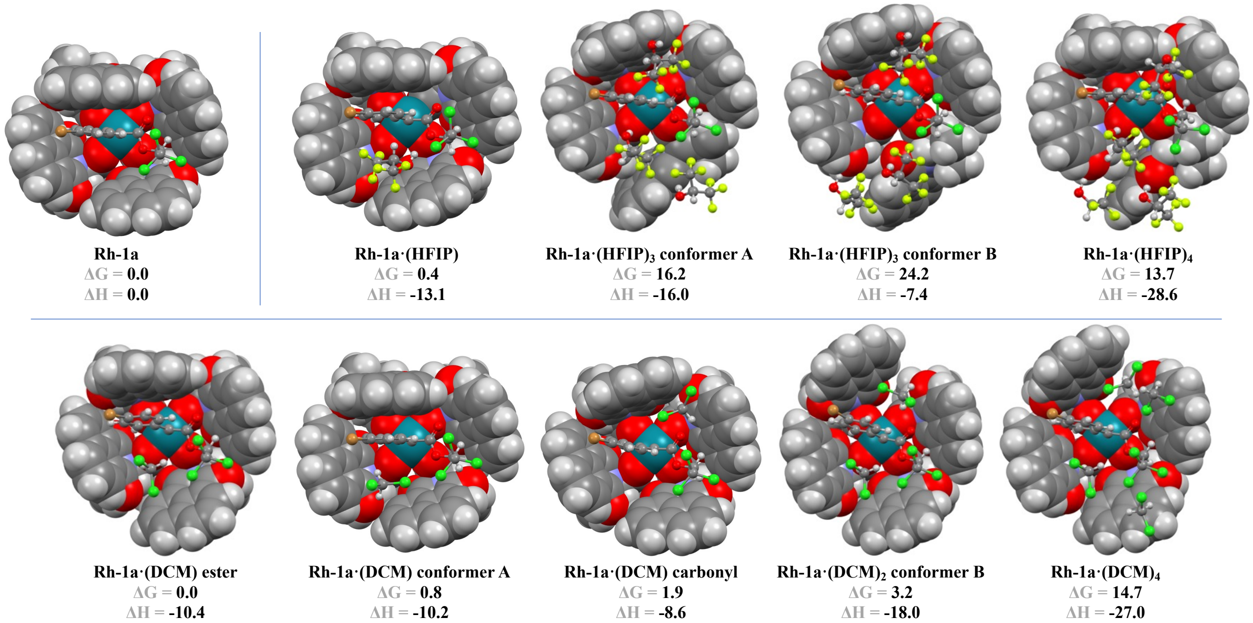

Figure S15: DFT computed solvent-cluster model of **Rh-1a** in HFIP (top) and DCM (bottom). All Gibbs free energies and enthalpies are in kcal/mol with respect to **Rh-1a**.

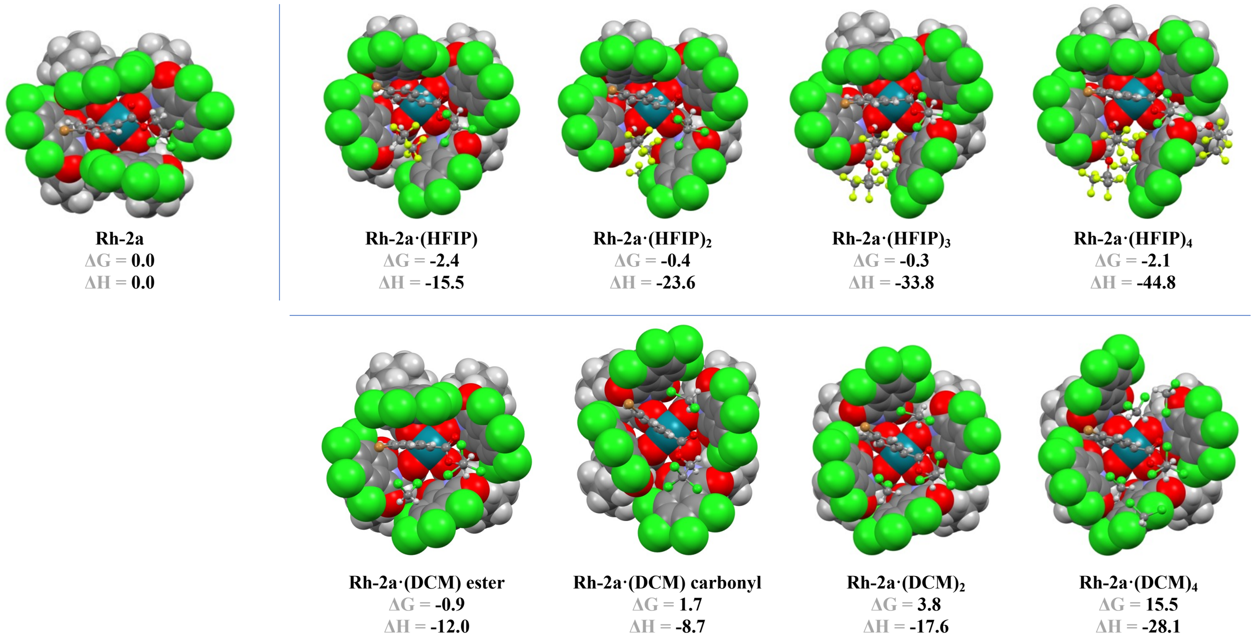

Figure S16: DFT computed solvent-cluster model of **Rh-2a** in HFIP (top) and DCM (bottom). All Gibbs free energies and enthalpies are in kcal/mol with respect to **Rh-2a**.

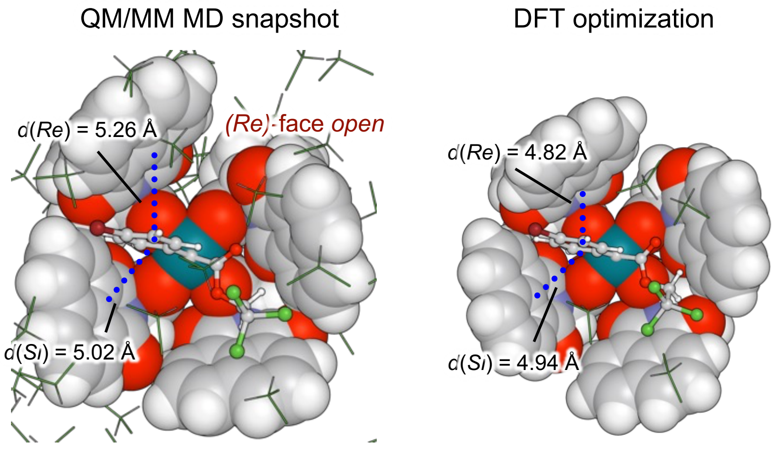

Rh-1a conformer **B**

Figure S17: Snapshot of conformer **B** of Rh_2(*S*-NTTL)$_4$–carbene (**Rh-1a**) in solution from AIMD simulations (left) and DFT optimization (right).

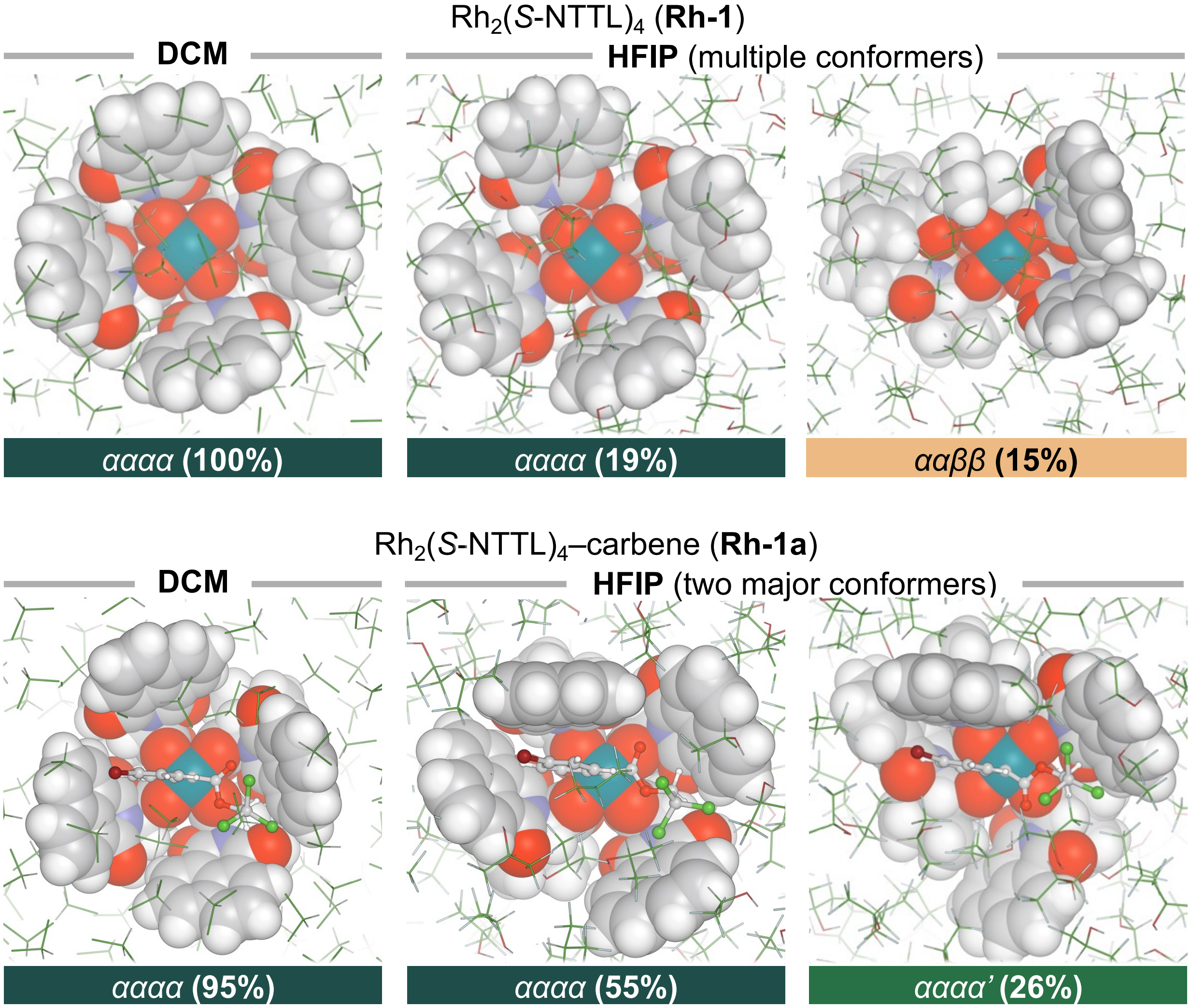

Figure S18: Representative structures of the dirhodium tetracarboxylate catalyst Rh$_2$(*S*-NTTL)$_4$, **Rh-1**, and its carbene complex **Rh-1a** in DCM and HFIP from classical MD simulations.

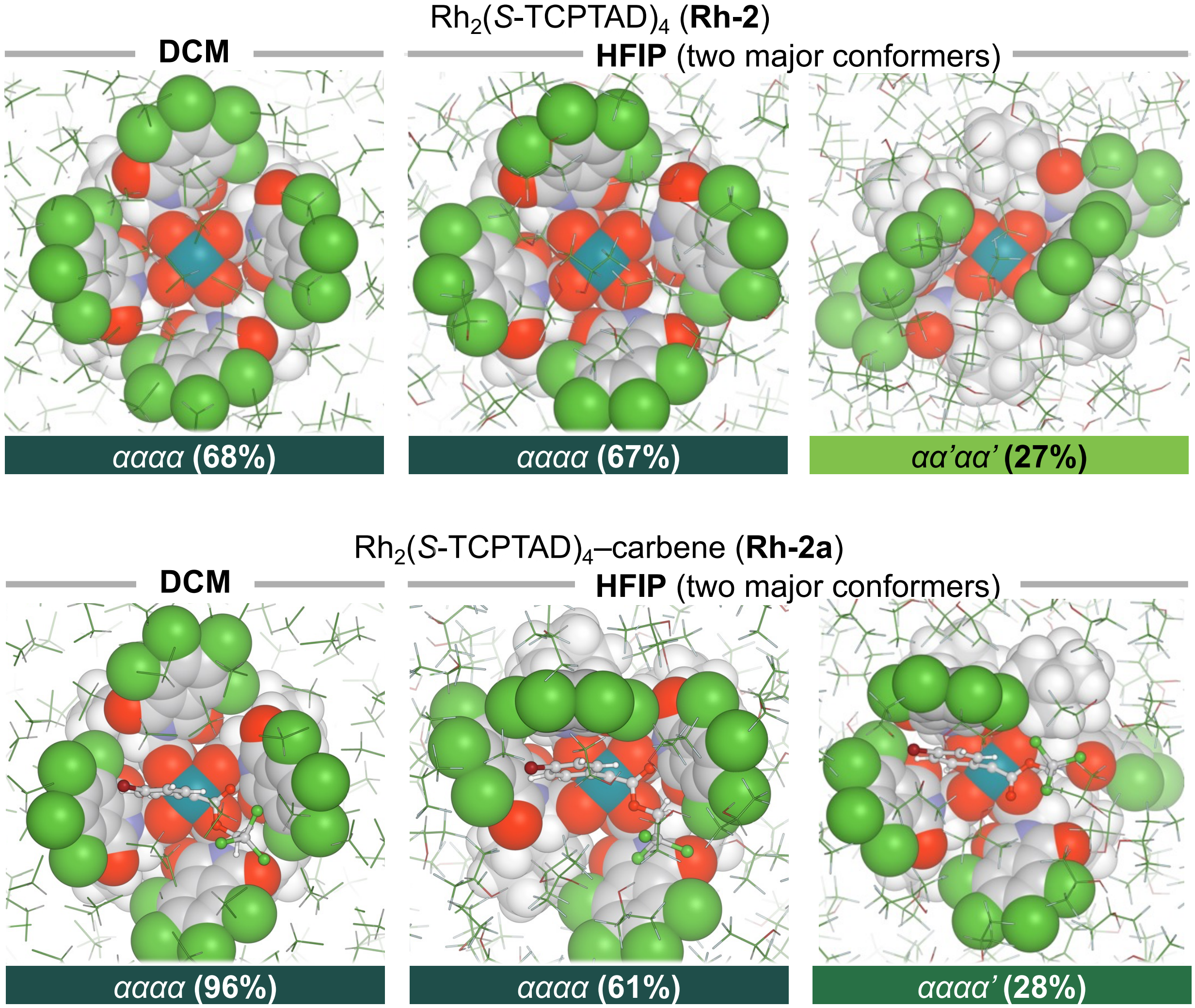

Figure S19: Representative structures of the dirhodium tetracarboxylate catalyst Rh_2(*S*-TCPTAD)$_4$, **Rh-2**, and its carbene complex **Rh-2a** in DCM and HFIP from classical MD simulations.

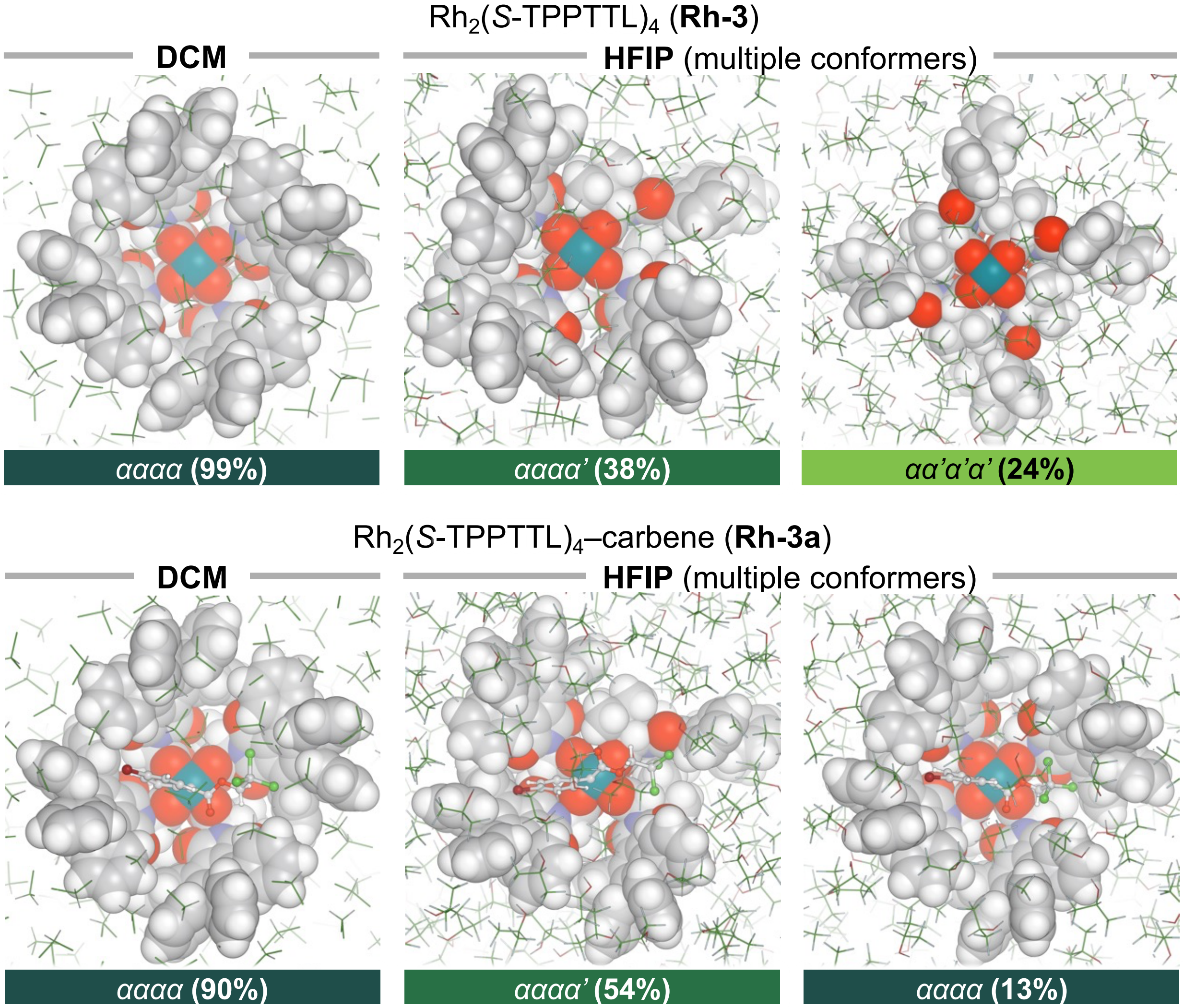

Figure S20: Representative structures of the dirhodium tetracarboxylate catalyst Rh$_2$(*S*-TPPTTL)$_4$, **Rh-3**, and its carbene complex **Rh-3a** in DCM and HFIP from classical MD simulations.

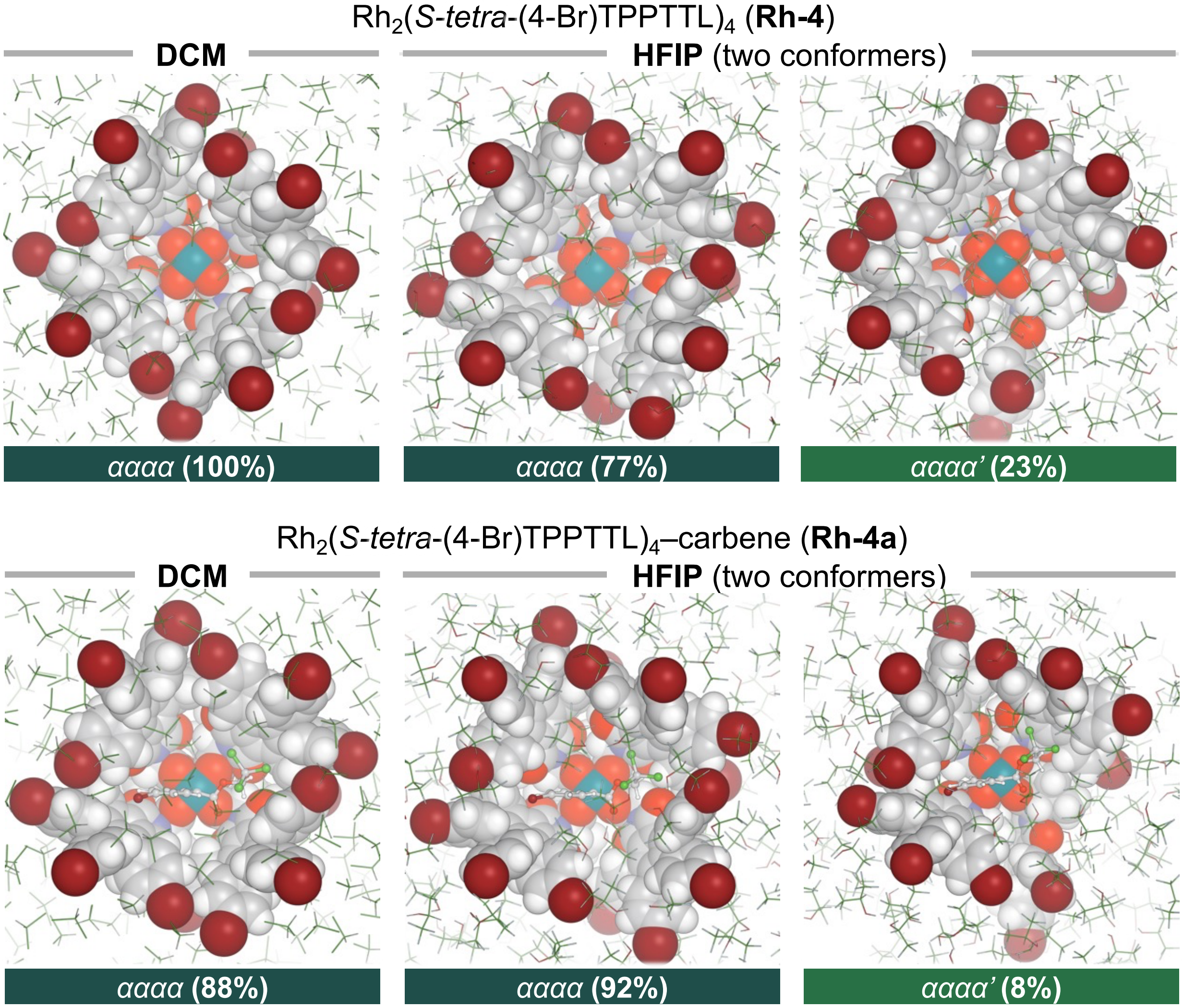

Figure S21: Representative structures of the dirhodium tetracarboxylate catalyst Rh_2(*S*-*tetra*-(4-Br)TPPTTL)$_4$, **Rh-4**, and its carbene complex **Rh-4a** in DCM and HFIP from classical MD simulations.

References

1. Sharland, J. C.; Dunstan, D.; Majumdar, D.; Gao, J.; Tan, K.; Malik, H. A.; Davies, H. M. L. Hexafluoroisopropanol for the selective deactivation of poisonous nucleophiles enabling catalytic asymmetric cyclopropanation of complex molecules. *ACS Catal.* **2022**, *12*, 12530-12542.
2. Sharland, J. C.; Wei, B.; Hardee, D. J.; Hodges, T. R.; Gong, W.; Voight, E. A.; Davies, H. M. L. Asymmetric synthesis of pharmaceutically relevant 1-aryl-2-heteroaryl- and 1,2-diheteroarylcyclopropane-1-carboxylates. *Chem. Sci.* **2021**, *12*, 11181-11190.
3. Wei, B.; Hatridge, T. A.; Jones, C. W.; Davies, H. M. L. Copper(II) acetate-induced oxidation of hydrazones to diazo compounds under flow conditions followed by dirhodium-catalyzed enantioselective cyclopropanation reactions. *Org. Lett.* **2021**, *23*, 5363-5367.
4. Ghanem, A.; Gardiner, M. G.; Williamson, R. M.; Müller, P. First x-ray structure of a n-naphthaloyl-tethered chiral dirhodium(II) complex: Structural basis for tether substitution improving asymmetric control in olefin cyclopropanation. *Chem. Eur. J.* **2010**, *16*, 3291-3295.
5. Liao, K.; Pickel, T. C.; Boyarskikh, V.; Bacsa, J.; Musaev, D. G.; Davies, H. M. L. Site-selective and stereoselective functionalization of non-activated tertiary C–H bonds. *Nature* **2017**, *551*, 609-613.
6. Fu, J.; Ren, Z.; Bacsa, J.; Musaev, D. G.; Davies, H. M. L. Desymmetrization of cyclohexanes by site- and stereoselective C–H functionalization. *Nature* **2018**, *564*, 395-399.
7. Garlets, Z. J.; Boni, Y. T.; Sharland, J. C.; Kirby, R. P.; Fu, J.; Bacsa, J.; Davies, H. M. L. Design, synthesis, and evaluation of extended C_4–symmetric dirhodium tetracarboxylate catalysts. *ACS Catal.* **2022**, *12*, 10841-10848.
8. Salomon-Ferrer, R.; Götz, A. W.; Poole, D.; Le Grand, S.; Walker, R. C. Routine microsecond molecular dynamics simulations with amber on GPUs. 2. Explicit solvent particle mesh Ewald. *J. Chem. Theory Comput.* **2013**, *9*, 3878-3888.
9. Case, D. A.; Aktulga, H. M.; Belfon, K.; Ben-Shalom, I. Y.; Berryman, J. T.; Brozell, S. R.; Cerutti, D. S.; Cheatham, T. E., III; Cisneros, G. A.; Cruzeiro, V. W. D.; Darden, T. A.; Forouzesh, N.; Ghazimirsaeed, M.; Giambaşu, G.; Giese, T.; Gilson, M. K.; Gohlke, H.; Goetz, A. W.; Harris, J.; Huang, Z.; Izadi, S.; Izmailov, S. A.; Kasavajhala, K.; Kaymak, M. C.; Kovalenko, A.; Kurtzman, T.; Lee, T. S.; Li, P.; Li, Z.; Lin, C.; Liu, J.; Luchko, T.; Luo, R.; Machado, M.; Manathunga, M.; Merz, K. M.; Miao, Y.; Mikhailovskii, O.; Monard, G.; Nguyen, H.; O'Hearn, K. A.; Onufriev, A.; Pan, F.; Pantano, S.; Rahnamoun, A.; Roe, D. R.; Roitberg, A.; Sagui, C.; Schott-Verdugo, S.; Shajan, A.; Shen, J.; Simmerling, C. L.; Skrynnikov, N. R.; Smith, J.; Swails, J.; Walker, R. C.; Wang, J.; Wang, J.; Wu, X.; Wu, Y.; Xiong, Y.; Xue, Y.; York, D. M.; Zhao, C.; Zhu, Q.; Kollman, P. A., *Amber 2020,* University of California, San Francisco, **2020**. https://ambermd.org/.
10. Li, P.; Merz, K. M., Jr. MCPB.py: A python based metal center parameter builder. *J. Chem. Inf. Model.* **2016**, *56*, 599-604.
11. Fioroni, M.; Burger, K.; Mark, A. E.; Roccatano, D. Model of 1,1,1,3,3,3-hexafluoro-propan-2-ol for molecular dynamics simulations. *J. Phys. Chem. B* **2001**, *105*, 10967-10975.
12. Wang, J.; Wolf, R. M.; Caldwell, J. W.; Kollman, P. A.; Case, D. A. Development and testing of a general amber force field. *J. Comput. Chem.* **2004**, *25*, 1157-1174.

13. Wang, J.; Wang, W.; Kollman, P. A.; Case, D. A. Automatic atom type and bond type perception in molecular mechanical calculations. *J. Mol. Graphics Modell.* **2006**, *25*, 247-260.
14. Singh, U. C.; Kollman, P. A. An approach to computing electrostatic charges for molecules. *J. Comput. Chem.* **1984**, *5*, 129-145.
15. Besler, B. H.; Merz Jr, K. M.; Kollman, P. A. Atomic charges derived from semiempirical methods. *J. Comput. Chem.* **1990**, *11*, 431-439.
16. Bayly, C. I.; Cieplak, P.; Cornell, W.; Kollman, P. A. A well-behaved electrostatic potential based method using charge restraints for deriving atomic charges: The RESP model. *J. Phys. Chem.* **1993**, *97*, 10269-10280.
17. Darden, T.; York, D.; Pedersen, L. Particle mesh Ewald: An N·log(N) method for Ewald sums in large systems. *J. Chem. Phys.* **1993**, *98*, 10089-10092.
18. Grimme, S.; Antony, J.; Ehrlich, S.; Krieg, H. A consistent and accurate ab initio parametrization of density functional dispersion correction (DFT-D) for the 94 elements H-Pu. *J. Chem. Phys.* **2010**, *132*, 154104.
19. Grimme, S.; Ehrlich, S.; Goerigk, L. Effect of the damping function in dispersion corrected density functional theory. *J. Comput. Chem.* **2011**, *32*, 1456-1465.
20. Grimme, S.; Bannwarth, C.; Shushkov, P. A robust and accurate tight-binding quantum chemical method for structures, vibrational frequencies, and noncovalent interactions of large molecular systems parametrized for all spd-block elements (Z = 1–86). *J. Chem. Theory Comput.* **2017**, *13*, 1989-2009.
21. Kühne, T. D.; Iannuzzi, M.; Del Ben, M.; Rybkin, V. V.; Seewald, P.; Stein, F.; Laino, T.; Khaliullin, R. Z.; Schütt, O.; Schiffmann, F.; Golze, D.; Wilhelm, J.; Chulkov, S.; Bani-Hashemian, M. H.; Weber, V.; Borštnik, U.; Taillefumier, M.; Jakobovits, A. S.; Lazzaro, A.; Pabst, H.; Müller, T.; Schade, R.; Guidon, M.; Andermatt, S.; Holmberg, N.; Schenter, G. K.; Hehn, A.; Bussy, A.; Belleflamme, F.; Tabacchi, G.; Glöß, A.; Lass, M.; Bethune, I.; Mundy, C. J.; Plessl, C.; Watkins, M.; VandeVondele, J.; Krack, M.; Hutter, J. CP2K: An electronic structure and molecular dynamics software package - Quickstep: Efficient and accurate electronic structure calculations. *J. Chem. Phys.* **2020**, *152*, 194103.
22. *CP2K version 2023.1,* The CP2K developers group, **2023**. https://www.cp2k.org/.
23. Byrd, R. H.; Lu, P.; Nocedal, J.; Zhu, C. A limited memory algorithm for bound constrained optimization. *SIAM J. Sci. Comput.* **1995**, *16*, 1190-1208.
24. Bussi, G.; Donadio, D.; Parrinello, M. Canonical sampling through velocity rescaling. *J. Chem. Phys.* **2007**, *126*, 014101.
25. Laino, T.; Mohamed, F.; Laio, A.; Parrinello, M. An efficient real space multigrid QM/MM electrostatic coupling. *J. Chem. Theory Comput.* **2005**, *1*, 1176-1184.
26. Essmann, U.; Perera, L.; Berkowitz, M. L.; Darden, T.; Lee, H.; Pedersen, L. G. A smooth particle mesh Ewald method. *J. Chem. Phys.* **1995**, *103*, 8577-8593.
27. Marx, D.; Hutter, J. *Ab Initio Molecular Dynamics: Basic Theory and Advanced Methods*; Cambridge University Press: Cambridge, U.K., **2009** DOI: 10.1017/CBO9780511609633.
28. Roe, D. R.; Cheatham, T. E., III. Ptraj and cpptraj: Software for processing and analysis of molecular dynamics trajectory data. *J. Chem. Theory Comput.* **2013**, *9*, 3084-3095.
29. Becke, A. D. Density-functional thermochemistry. III. The role of exact exchange. *J. Chem. Phys.* **1993**, *98*, 5648-5652.

30. Lee, C.; Yang, W.; Parr, R. G. Development of the Colle-Salvetti correlation-energy formula into a functional of the electron density. *Phys. Rev. B* **1988**, *37*, 785-789.
31. Andrae, D.; Häußermann, U.; Dolg, M.; Stoll, H.; Preuß, H. Energy-adjusted ab initio pseudopotentials for the second and third row transition elements. *Theoret. Chim. Acta* **1990**, *77*, 123-141.
32. Martin, J. M. L.; Sundermann, A. Correlation consistent valence basis sets for use with the Stuttgart–Dresden–Bonn relativistic effective core potentials: The atoms Ga–Kr and In–Xe. *J. Chem. Phys.* **2001**, *114*, 3408-3420.
33. Grimme, S. Supramolecular binding thermodynamics by dispersion-corrected density functional theory. *Chem. Eur. J.* **2012**, *18*, 9955-9964.
34. Luchini, G.; Alegre-Requena, J. V.; Funes-Ardoiz, I.; Paton, R. S. Goodvibes: Automated thermochemistry for heterogeneous computational chemistry data. *F1000Research* **2020**, *9*,
35. Marenich, A. V.; Cramer, C. J.; Truhlar, D. G. Universal solvation model based on solute electron density and on a continuum model of the solvent defined by the bulk dielectric constant and atomic surface tensions. *J. Phys. Chem. B* **2009**, *113*, 6378-6396.
36. Engelage, E.; Schulz, N.; Heinen, F.; Huber, S. M.; Truhlar, D. G.; Cramer, C. J. Refined smd parameters for bromine and iodine accurately model halogen-bonding interactions in solution. *Chem. Eur. J.* **2018**, *24*, 15983-15987.
37. Simlandy, A. K.; Rodphon, W.; Alturaifi, T. M.; Mai, B. K.; Ni, H.-Q.; Gurak, J. A., Jr.; Liu, P.; Engle, K. M. Catalytic addition of nitroalkanes to unactivated alkenes via directed carbopalladation. *ACS Catal.* **2022**, *12*, 13755-13762.
38. Mammen, M.; Shakhnovich, E. I.; Deutch, J. M.; Whitesides, G. M. Estimating the entropic cost of self-assembly of multiparticle hydrogen-bonded aggregates based on the cyanuric acid·melamine lattice. *J. Org. Chem.* **1998**, *63*, 3821-3830.

Cartesian Coordinates

All calculations were carried out with the B3LYP-D3(BJ) functional.

2

6-31G(d) SCF energy in gas phase (au):	-235.866452
6-31G(d) enthalpy in gas phase (au):	-235.691158
6-31G(d) free energy in gas phase (au):	-235.728473
6-311+G(d,p) SCF energy in implicit solvent (DCM) (au):	-235.947892
6-311+G(d,p) enthalpy in implicit solvent (DCM) (au):	-235.772598
6-311+G(d,p) free energy in implicit solvent (DCM) (au):	-235.809913
6-311+G(d,p) SCF energy in implicit solvent (HFIP) (au):	-235.946356
6-311+G(d,p) enthalpy in implicit solvent (HFIP) (au):	-235.771062
6-311+G(d,p) free energy in implicit solvent (HFIP) (au):	-235.808377

Cartesian coordinates

ATOM	X	Y	Z
C	3.070900	-0.203550	-0.443170
C	2.041370	-0.181080	0.403660
H	3.065570	0.382500	-1.360280
H	3.957740	-0.802470	-0.254240
H	2.086450	-0.787380	1.310320
C	0.775310	0.604160	0.206740
C	-0.470030	-0.293620	0.105630
H	0.859200	1.221320	-0.697470
H	0.638060	1.298770	1.049770
C	-1.770890	0.499660	-0.052450
H	-0.348640	-0.979030	-0.744200
H	-0.536040	-0.926900	1.002310
C	-3.007190	-0.397150	-0.155810
H	-1.882200	1.184780	0.799810
H	-1.700530	1.134590	-0.946750
H	-3.923320	0.193370	-0.268910
H	-2.934410	-1.070590	-1.018440
H	-3.118750	-1.019520	0.740480

3

6-31G(d) SCF energy in gas phase (au):	-4723.032951
6-31G(d) enthalpy in gas phase (au):	-4722.697768
6-31G(d) free energy in gas phase (au):	-4722.770882
6-311+G(d,p) SCF energy in implicit solvent (DCM) (au):	-4726.119255
6-311+G(d,p) enthalpy in implicit solvent (DCM) (au):	-4725.784071
6-311+G(d,p) free energy in implicit solvent (DCM) (au):	-4725.857185
6-311+G(d,p) SCF energy in implicit solvent (HFIP) (au):	-4726.113547
6-311+G(d,p) enthalpy in implicit solvent (HFIP) (au):	-4725.778364
6-311+G(d,p) free energy in implicit solvent (HFIP) (au):	-4725.851478

Cartesian coordinates

ATOM	X	Y	Z
C	-0.032140	-0.022540	0.916550
C	1.401690	-0.333400	0.609110
C	-0.967690	-1.160540	0.694970
C	2.381810	-0.324490	1.604920
C	1.781450	-0.641920	-0.704050

O	-2.228060	-0.871010	1.169510
O	-0.698390	-2.206020	0.151240
C	3.716580	-0.601280	1.305730
H	2.104270	-0.104250	2.631170
C	3.107530	-0.921810	-1.021060
H	1.027730	-0.669380	-1.484030
C	-3.269530	-1.751870	0.797770
C	4.066430	-0.894890	-0.008880
H	4.471980	-0.591940	2.083100
H	3.396080	-1.157420	-2.039000
H	-2.861330	-2.658560	0.347400
H	-3.851200	-1.991800	1.690130
C	-4.199220	-1.062500	-0.210570
Br	5.890940	-1.267160	-0.434760
Cl	-5.525210	-2.212310	-0.596240
Cl	-4.886440	0.438490	0.499700
C	0.079370	6.280880	-0.523210
C	0.610210	4.855900	-0.698060
H	-0.934820	6.376370	-0.929720
H	0.038030	6.558900	0.537000
H	1.637490	4.794480	-0.312550
H	0.671390	4.613580	-1.768190
C	-0.255370	3.806980	0.007020
C	0.276140	2.378770	-0.166040
H	-0.317110	4.041060	1.079190
H	-1.283860	3.863850	-0.377440
H	1.300120	2.316510	0.220780
H	0.337780	2.134710	-1.235660
C	-0.600460	1.364850	0.534300
C	-0.388600	1.019950	1.968950
H	-1.631250	1.339500	0.191130
H	-1.249380	0.822960	2.596820
H	0.453160	1.485490	2.473550
H	0.715220	7.011190	-1.035790
Cl	-3.296880	-0.648010	-1.707110

Rh-1

6-31G(d)-SDD(Rh) SCF energy in gas phase (au):	-4426.863784
6-31G(d)-SDD(Rh) enthalpy in gas phase (au):	-4425.531961
6-31G(d)-SDD(Rh) free energy in gas phase (au):	-4425.712910
6-311+G(d,p)-SDD(Rh) SCF energy in implicit solvent (DCM) (au):	-4428.100074
6-311+G(d,p)-SDD(Rh) enthalpy in implicit solvent (DCM) (au):	-4426.768252
6-311+G(d,p)-SDD(Rh) free energy in implicit solvent (DCM) (au):	-4426.949201
6-311+G(d,p)-SDD(Rh) SCF energy in implicit solvent (HFIP) (au):	-4428.106888
6-311+G(d,p)-SDD(Rh) enthalpy in implicit solvent (HFIP) (au):	-4426.775065
6-311+G(d,p)-SDD(Rh) free energy in implicit solvent (HFIP) (au):	-4426.956014

Cartesian coordinates

ATOM	X	Y	Z
Rh	0.000080	-0.000380	-0.275480
Rh	-0.000130	-0.000210	-2.662880
O	-1.113900	1.708740	-0.336810
O	-1.072660	1.743950	-2.600120
O	-3.612960	4.576110	0.642660
O	0.578340	3.978880	-1.085660

O	1.708890	1.113830	-0.337030
O	1.743880	1.072680	-2.600360
O	4.576590	3.612880	0.642840
O	3.978950	-0.577660	-1.087150
O	1.114220	-1.709340	-0.337400
O	1.072360	-1.744530	-2.600720
O	3.613600	-4.577160	0.641190
O	-0.578420	-3.978340	-1.084780
O	-1.708840	-1.114560	-0.337050
O	-1.744230	-1.072870	-2.600360
O	-4.576610	-3.613480	0.642510
O	-3.979440	0.577590	-1.086460
N	-1.522190	4.338550	-0.267160
N	4.338500	1.522500	-0.267600
N	1.522360	-4.338780	-0.267380
N	-4.338770	-1.522840	-0.267520
C	-1.350420	2.240790	-1.468530
C	-2.102350	3.570420	-1.393260
H	-3.096520	3.294180	-1.037150
C	-2.401440	4.748460	0.745210
C	-1.805100	5.398090	1.933460
C	-2.632280	5.882100	2.932320
H	-3.705600	5.796760	2.804150
C	-2.082180	6.468910	4.089530
H	-2.742770	6.848470	4.862920
C	-0.712840	6.554800	4.245710
H	-0.289220	6.997790	5.143320
C	0.165260	6.065490	3.245550
C	1.579390	6.108950	3.368350
H	2.012560	6.537540	4.268510
C	2.389860	5.608520	2.370450
H	3.470030	5.623070	2.469720
C	1.826190	5.060900	1.199650
H	2.471600	4.670200	0.424520
C	0.452440	5.004370	1.046860
C	-0.399050	5.491220	2.067300
C	-0.119680	4.411350	-0.177680
C	-2.327570	4.375330	-2.708090
C	-3.043910	5.690870	-2.345770
H	-3.290410	6.236430	-3.263320
H	-2.406290	6.339720	-1.735400
H	-3.970180	5.508040	-1.792690
C	-1.040240	4.708940	-3.480410
H	-0.473200	3.809210	-3.723250
H	-0.388110	5.374530	-2.909800
H	-1.309010	5.217340	-4.414660
C	-3.271410	3.545320	-3.604000
H	-2.799930	2.617210	-3.931150
H	-3.542370	4.129520	-4.490860
H	-4.196350	3.288830	-3.073600
C	2.240650	1.350760	-1.468820
C	3.570080	2.103120	-1.393260
H	3.293370	3.096930	-1.036580
C	4.748940	2.401320	0.744880
C	5.398980	1.804450	1.932640

C	5.883410	2.631180	2.931670
H	5.798140	3.704560	2.803970
C	6.470500	2.080540	4.088490
H	6.850430	2.740790	4.862000
C	6.556180	0.711130	4.244160
H	6.999280	0.287100	5.141520
C	6.066400	-0.166510	3.243820
C	6.109590	-1.580700	3.366120
H	6.538270	-2.014280	4.266040
C	5.608740	-2.390730	2.368050
H	5.623080	-3.470940	2.466920
C	5.060970	-1.826530	1.197600
H	4.669950	-2.471530	0.422290
C	5.004710	-0.452710	1.045300
C	5.491950	0.398330	2.065920
C	4.411550	0.119930	-0.178900
C	4.374860	2.329410	-2.707970
C	5.689700	3.046890	-2.345440
H	5.506000	3.972900	-1.792240
H	6.235070	3.293970	-3.262940
H	6.339090	2.409760	-1.735110
C	4.709870	1.042480	-3.480400
H	5.375780	0.390810	-2.909620
H	5.218510	1.311850	-4.414350
H	3.810740	0.474720	-3.723750
C	3.544010	3.272680	-3.603710
H	3.286990	4.197420	-3.073220
H	2.616160	2.800600	-3.930720
H	4.127850	3.544070	-4.490680
C	1.350340	-2.241390	-1.469220
C	2.102100	-3.571130	-1.394030
H	3.096550	-3.295040	-1.038610
C	2.402020	-4.748520	0.744750
C	1.806030	-5.396940	1.933840
C	2.633540	-5.880530	2.932640
H	3.706830	-5.795880	2.803780
C	2.083800	-6.466020	4.090680
H	2.744640	-6.845210	4.864060
C	0.714500	-6.551060	4.247740
H	0.291170	-6.993050	5.145970
C	-0.163910	-6.062200	3.247630
C	-1.578000	-6.104850	3.371260
H	-2.010870	-6.532380	4.272060
C	-2.388800	-5.605010	2.373320
H	-3.468910	-5.618980	2.473250
C	-1.825520	-5.058720	1.201730
H	-2.471070	-4.668520	0.426470
C	-0.451820	-5.002880	1.048180
C	0.400020	-5.489230	2.068570
C	0.119880	-4.410870	-0.177050
C	2.326280	-4.376460	-2.708740
C	3.042880	-5.691890	-2.346520
H	3.969610	-5.508880	-1.794280
H	3.288590	-6.237780	-3.264090
H	2.405760	-6.340510	-1.735370

C	1.038280	-4.710230	-3.479870
H	0.386600	-5.375610	-2.908540
H	1.306230	-5.218900	-4.414220
H	0.471100	-3.810510	-3.722480
C	3.269380	-3.546740	-3.605680
H	4.194780	-3.290110	-3.076140
H	2.797650	-2.618710	-3.932730
H	3.539590	-4.131220	-4.492590
C	-2.240930	-1.350970	-1.468780
C	-3.570410	-2.103180	-1.393390
H	-3.293890	-3.097150	-1.036980
C	-4.748960	-2.401960	0.744880
C	-5.398920	-1.805430	1.932870
C	-5.883120	-2.632470	2.931760
H	-5.797730	-3.705800	2.803760
C	-6.470130	-2.082190	4.088780
H	-6.849900	-2.742670	4.862180
C	-6.556000	-0.712820	4.244770
H	-6.999120	-0.289060	5.142250
C	-6.066480	0.165120	3.244580
C	-6.109860	1.579270	3.367200
H	-6.538610	2.012580	4.267220
C	-5.609200	2.389600	2.369290
H	-5.623710	3.469770	2.468430
C	-5.061440	1.825760	1.198650
H	-4.670570	2.471000	0.423470
C	-5.004980	0.451980	1.046040
C	-5.492040	-0.399370	2.066500
C	-4.411850	-0.120320	-0.178360
C	-4.375180	-2.329000	-2.708170
C	-5.690580	-3.045520	-2.345690
H	-6.339600	-2.407880	-1.735520
H	-5.507570	-3.971580	-1.792330
H	-6.236040	-3.292370	-3.263210
C	-4.709040	-1.041950	-3.480820
H	-5.374650	-0.389750	-2.910310
H	-5.217540	-1.311060	-4.414930
H	-3.809410	-0.474870	-3.723950
C	-3.544920	-3.272970	-3.603710
H	-2.616850	-2.801470	-3.930930
H	-4.128990	-3.544320	-4.490530
H	-3.288320	-4.197700	-3.072980

Rh-2

6-31G(d)-SDD(Rh) SCF energy in gas phase (au):	-12094.828600
6-31G(d)-SDD(Rh) enthalpy in gas phase (au):	-12093.378620
6-31G(d)-SDD(Rh) free energy in gas phase (au):	-12093.599860
6-311+G(d,p)-SDD(Rh) SCF energy in implicit solvent (DCM) (au):	-12096.646660
6-311+G(d,p)-SDD(Rh) enthalpy in implicit solvent (DCM) (au):	-12095.196690
6-311+G(d,p)-SDD(Rh) free energy in implicit solvent (DCM) (au):	-12095.417920
6-311+G(d,p)-SDD(Rh) SCF energy in implicit solvent (HFIP) (au):	-12096.657800
6-311+G(d,p)-SDD(Rh) enthalpy in implicit solvent (HFIP) (au):	-12095.207820
6-311+G(d,p)-SDD(Rh) free energy in implicit solvent (HFIP) (au):	-12095.429060

Cartesian coordinates

ATOM	X	Y	Z
Rh	0.007120	0.188530	-2.418150
Rh	-0.086690	-0.024490	-0.040770
Cl	0.981370	-5.182660	1.999970
Cl	1.663200	-5.253930	5.082270
Cl	5.529450	-1.479500	4.323050
Cl	3.925080	-3.417700	6.238720
Cl	-3.096790	-6.770040	3.019570
Cl	-5.532210	-5.491070	4.569820
Cl	-5.731710	-1.431940	0.942550
Cl	-6.837280	-2.837600	3.534120
Cl	6.530280	1.469060	-0.647150
Cl	7.909470	1.220840	2.182080
Cl	3.910970	4.188160	4.397970
Cl	6.583580	2.532390	4.697910
Cl	-6.771130	2.605340	3.656570
Cl	-5.322300	4.447700	5.772450
Cl	-1.086530	5.025360	2.389850
Cl	-2.499220	5.651260	5.141210
O	1.875670	-0.568970	-0.087730
O	1.962080	-0.423450	-2.342540
O	5.344550	-0.813520	1.272810
O	2.090300	-3.553450	-0.435800
O	-0.505180	-1.788040	-2.543370
O	-0.639530	-1.975470	-0.292950
O	-3.488250	-2.154790	-1.152190
O	-1.452040	-5.935130	0.478030
O	0.525800	2.158670	-2.151830
O	0.540360	1.902820	0.097710
O	1.970440	4.787770	1.977910
O	3.588070	2.264080	-1.495370
O	-2.048570	0.569470	-0.129750
O	-1.951680	0.763580	-2.379720
O	-1.968020	3.502600	-0.209320
O	-5.993810	1.535190	0.797970
N	3.888200	-2.159360	0.063250
N	-2.280020	-4.071970	-0.633090
N	2.581570	3.757850	-0.016850
N	-3.989010	2.372520	-0.020620
C	-3.406150	-4.700550	1.284140
C	-3.851570	-5.326690	2.437990
C	-4.937120	-4.742440	3.128230
C	-5.527590	-3.552360	2.658940
C	-5.055740	-2.926280	1.482160
C	-4.004010	-3.529760	0.808230
C	-3.285880	-3.114050	-0.437550
C	2.449370	-0.723990	-1.215930
C	2.886370	-3.100560	0.358920
C	-1.154270	-3.903910	-1.558820
H	-0.328680	-4.451040	-1.087920
C	-0.739480	-2.429500	-1.476090
C	2.999430	-3.372230	1.825910
C	2.256800	-4.213290	2.642790
C	2.561600	-4.222490	4.023160
C	3.577100	-3.394660	4.544450

C	3.579980	2.871600	-0.444620
C	4.552760	2.772370	0.686660
C	5.775850	2.124620	0.764420
C	6.407750	2.056400	2.024890
C	5.818720	2.665170	3.151270
C	4.612410	3.391600	3.032210
C	3.998750	3.429190	1.788870
C	2.733330	4.094030	1.342700
C	4.308330	-2.533940	3.696150
C	4.002050	-2.548570	2.343880
C	1.297920	3.965910	-0.706430
H	0.646030	4.375420	0.069860
C	0.733010	2.566590	-0.971530
C	4.539970	-1.718990	1.225820
C	3.864970	-1.303980	-1.129690
H	4.502520	-0.455930	-0.856850
C	-2.261780	-5.037630	0.381390
C	-5.006510	2.226470	0.930640
C	-4.597050	3.075430	2.094200
C	-5.228780	3.304870	3.306620
C	-4.568540	4.125460	4.249080
C	-3.301180	4.671510	3.963100
C	-2.670260	4.422170	2.722550
C	-3.349150	3.634910	1.803890
C	-2.957670	3.206410	0.426640
C	-3.921280	1.546470	-1.228090
H	-4.615020	0.719410	-1.034240
C	-2.522760	0.910450	-1.258370
C	4.484090	-1.922370	-2.407280
C	3.609480	-3.002660	-3.082440
C	4.761270	-0.783450	-3.423220
C	5.841550	-2.558920	-2.021030
H	2.636620	-2.580840	-3.352030
H	3.416920	-3.820650	-2.382670
C	4.317640	-3.547630	-4.336390
H	5.385520	-0.015190	-2.949110
H	3.823690	-0.299690	-3.705600
C	5.466810	-1.338240	-4.672160
H	5.677450	-3.369680	-1.300800
H	6.473410	-1.811200	-1.522350
C	6.551850	-3.113600	-3.268780
H	3.676140	-4.307330	-4.801800
C	4.570450	-2.402090	-5.332410
C	5.660970	-4.181360	-3.930940
H	5.642890	-0.514560	-5.375490
C	6.809240	-1.971400	-4.267420
H	7.505850	-3.563270	-2.965390
H	3.616030	-1.951700	-5.637010
H	5.050630	-2.790040	-6.240840
H	5.488950	-5.013320	-3.234710
H	6.166220	-4.597130	-4.812920
H	7.331030	-2.356640	-5.153620
H	7.460920	-1.212820	-3.812920
C	-1.376180	-4.518020	-2.959960
C	-2.455930	-3.791380	-3.792550

C	-0.028960	-4.510200	-3.727160
C	-1.811160	-5.995080	-2.785750
H	-2.189930	-2.738950	-3.917760
H	-3.414070	-3.818230	-3.259560
C	-2.604900	-4.470240	-5.167300
H	0.730320	-5.038720	-3.134020
H	0.322470	-3.484570	-3.858860
C	-0.191660	-5.184910	-5.099630
H	-2.765120	-6.037450	-2.245400
H	-1.075230	-6.530880	-2.174120
C	-1.970960	-6.679370	-4.155050
H	-3.364620	-3.932590	-5.748740
C	-1.257780	-4.427190	-5.913220
C	-3.038300	-5.934700	-4.977040
H	0.770010	-5.151090	-5.627970
C	-0.629400	-6.646940	-4.908330
H	-2.282530	-7.719800	-3.998020
H	-0.944680	-3.384940	-6.062900
H	-1.363950	-4.879590	-6.908310
H	-4.007710	-5.975920	-4.462070
H	-3.170010	-6.421560	-5.952620
H	-0.732060	-7.143850	-5.882300
H	0.134400	-7.197400	-4.342830
C	-4.409040	2.268640	-2.513300
C	-3.435110	3.357650	-3.019570
C	-4.638860	1.216540	-3.627780
C	-5.772110	2.942450	-2.213610
H	-2.455690	2.919340	-3.224690
H	-3.287720	4.116360	-2.243870
C	-3.994200	4.019790	-4.291710
H	-5.348500	0.457910	-3.268260
H	-3.703260	0.701770	-3.856510
C	-5.191240	1.891040	-4.895850
H	-5.646560	3.699090	-1.429190
H	-6.480510	2.198280	-1.829190
C	-6.335310	3.615140	-3.478650
H	-3.279830	4.778220	-4.637900
C	-4.189660	2.953970	-5.385370
C	-5.345140	4.684800	-3.974190
H	-5.328820	1.128040	-5.672410
C	-6.540040	2.558930	-4.578610
H	-7.295970	4.084790	-3.232150
H	-3.227470	2.481160	-5.624790
H	-4.558810	3.422500	-6.307490
H	-5.211060	5.459530	-3.207010
H	-5.744460	5.181550	-4.868500
H	-6.952960	3.029890	-5.480650
H	-7.265210	1.803840	-4.246850
C	1.325970	4.993410	-1.855050
C	2.229250	4.610670	-3.047990
C	-0.126610	5.194950	-2.355780
C	1.825010	6.339860	-1.274980
H	1.914190	3.649760	-3.461920
H	3.263950	4.487770	-2.709540
C	2.169600	5.709510	-4.126260

H	-0.771780	5.464490	-1.510470
H	-0.513390	4.257380	-2.762570
C	-0.175600	6.290970	-3.432480
H	2.853160	6.225680	-0.907890
H	1.207880	6.623290	-0.411950
C	1.775810	7.441520	-2.348660
H	2.810010	5.415510	-4.967460
C	0.719630	5.880240	-4.615490
C	2.671160	7.039350	-3.534750
H	-1.212480	6.406040	-3.774960
C	0.326970	7.618650	-2.838810
H	2.136780	8.381880	-1.913050
H	0.356520	4.939370	-5.050800
H	0.675110	6.641740	-5.405730
H	3.712630	6.933450	-3.201990
H	2.655570	7.825820	-4.301180
H	0.280510	8.414670	-3.593980
H	-0.318270	7.925670	-2.004670

Rh-1a

6-31G(d)-SDD(Rh) SCF energy in gas phase (au): -8914.038606
6-31G(d)-SDD(Rh) enthalpy in gas phase (au): -8912.549012
6-31G(d)-SDD(Rh) free energy in gas phase (au): -8912.759260
6-311+G(d,p)-SDD(Rh) SCF energy in implicit solvent (DCM) (au): -8918.253693
6-311+G(d,p)-SDD(Rh) enthalpy in implicit solvent (DCM) (au): -8916.764098
6-311+G(d,p)-SDD(Rh) free energy in implicit solvent (DCM) (au): -8916.974347
6-311+G(d,p)-SDD(Rh) SCF energy in implicit solvent (HFIP) (au): -8918.265756
6-311+G(d,p)-SDD(Rh) enthalpy in implicit solvent (HFIP) (au): -8916.776162
6-311+G(d,p)-SDD(Rh) free energy in implicit solvent (HFIP) (au): -8916.986410

Cartesian coordinates

ATOM	X	Y	Z
Rh	-0.038320	0.781680	0.788050
Rh	-0.132830	2.054520	2.892290
O	1.169100	-0.567870	1.750250
O	1.108960	0.618100	3.684070
O	2.983740	-3.891500	2.578460
O	3.866100	0.582600	2.301340
O	1.640160	1.875250	0.391660
O	1.475500	3.175960	2.232890
O	5.172830	2.852950	-1.043680
O	0.831750	4.291640	-0.964590
O	-1.226320	2.235470	0.016560
O	-1.374340	3.407900	1.950220
O	-2.820790	4.159630	-2.740860
O	-4.016490	2.016090	1.103410
O	-1.723310	-0.200170	1.410730
O	-1.740000	0.879290	3.403720
O	-5.016270	-2.310380	1.392790
O	-0.910950	-2.185260	3.400560
N	3.510400	-1.659620	2.549260
N	2.992740	3.547280	-0.964760
N	-3.554280	3.209790	-0.787670
N	-2.998020	-2.275660	2.479750

C	1.509950	-0.329330	2.958830
C	2.442500	-1.393440	3.542610
H	1.840630	-2.302460	3.533800
C	3.659720	-2.982890	2.106570
C	4.670260	-3.232890	1.053870
C	4.866400	-4.522030	0.590090
H	4.279930	-5.324100	1.024040
C	5.807640	-4.777750	-0.427580
H	5.946900	-5.793420	-0.784280
C	6.543820	-3.746140	-0.974080
H	7.260980	-3.941430	-1.766350
C	6.370970	-2.413170	-0.522510
C	7.075220	-1.311710	-1.076850
H	7.790920	-1.497080	-1.873070
C	6.839090	-0.026340	-0.632490
H	7.359710	0.817180	-1.072410
C	5.895240	0.215340	0.388410
H	5.691140	1.227610	0.711330
C	5.195290	-0.836550	0.951940
C	5.418070	-2.162360	0.509150
C	4.162600	-0.558550	1.972280
C	2.941300	-1.220280	5.006930
C	3.859090	-2.412360	5.342240
H	4.157050	-2.358550	6.395070
H	4.773600	-2.398800	4.738860
H	3.356270	-3.369940	5.174620
C	3.710500	0.086070	5.263980
H	3.114620	0.959800	4.997320
H	4.643860	0.124300	4.695370
H	3.965780	0.144910	6.329160
C	1.705880	-1.289550	5.929660
H	1.047290	-0.432720	5.780770
H	2.031770	-1.301580	6.976100
H	1.124280	-2.200450	5.745210
C	1.962220	2.881380	1.112210
C	3.092030	3.726280	0.505360
H	4.007580	3.186890	0.762200
C	4.075790	2.928110	-1.594650
C	3.830930	2.317920	-2.921580
C	4.877440	1.716870	-3.600400
H	5.875100	1.783600	-3.181830
C	4.635950	1.007370	-4.792200
H	5.462900	0.535390	-5.311990
C	3.349000	0.871970	-5.276460
H	3.161240	0.290460	-6.175170
C	2.254060	1.474100	-4.609670
C	0.908050	1.340330	-5.045880
H	0.703890	0.741430	-5.929840
C	-0.121190	1.950730	-4.361040
H	-1.149880	1.825000	-4.680930
C	0.144760	2.738230	-3.220080
H	-0.662870	3.223880	-2.688790
C	1.442580	2.874940	-2.762020
C	2.512340	2.232630	-3.429650
C	1.698930	3.644270	-1.530240

C	3.301930	5.176610	1.037420
C	4.335170	5.863410	0.125620
H	5.253250	5.272990	0.041900
H	4.590940	6.846790	0.535710
H	3.934820	6.013950	-0.882530
C	2.029960	6.038720	1.098740
H	1.628120	6.235690	0.104180
H	2.276800	6.997910	1.570370
H	1.247460	5.557320	1.688830
C	3.901040	5.056000	2.455010
H	4.833260	4.477500	2.443090
H	3.203480	4.570540	3.140980
H	4.131600	6.054060	2.844170
C	-1.681360	3.173640	0.750610
C	-2.629950	4.086960	-0.026960
H	-1.996680	4.525510	-0.801760
C	-3.511890	3.311240	-2.188050
C	-4.339800	2.359400	-2.961910
C	-4.354670	2.437360	-4.342460
H	-3.768940	3.212970	-4.823820
C	-5.113730	1.520070	-5.098710
H	-5.111490	1.589430	-6.182140
C	-5.856370	0.540690	-4.470820
H	-6.438560	-0.168050	-5.053500
C	-5.867890	0.435430	-3.056130
C	-6.598070	-0.563760	-2.359530
H	-7.191540	-1.270520	-2.932140
C	-6.536360	-0.654850	-0.984480
H	-7.068020	-1.439610	-0.458000
C	-5.749930	0.252210	-0.244290
H	-5.683630	0.164530	0.831820
C	-5.037000	1.246450	-0.889280
C	-5.088180	1.359670	-2.299200
C	-4.181660	2.159530	-0.101610
C	-3.300420	5.278450	0.710580
C	-4.200450	6.011380	-0.303010
H	-3.642330	6.305520	-1.197930
H	-4.614450	6.914970	0.158080
H	-5.041060	5.383650	-0.618580
C	-4.140900	4.880870	1.934850
H	-5.003730	4.271780	1.653090
H	-4.515450	5.790550	2.420450
H	-3.549940	4.315700	2.657210
C	-2.174190	6.239450	1.146160
H	-1.550160	6.531590	0.293030
H	-1.529500	5.782310	1.899240
H	-2.611850	7.149990	1.571190
C	-2.144630	-0.000230	2.599870
C	-3.304220	-0.918650	2.993450
H	-4.133520	-0.586290	2.366700
C	-3.949360	-2.868110	1.643000
C	-3.614550	-4.187550	1.062240
C	-4.564100	-4.866200	0.316220
H	-5.545890	-4.422140	0.196460
C	-4.248520	-6.103340	-0.279130

H	-5.002810	-6.625340	-0.859040
C	-2.983630	-6.639910	-0.144600
H	-2.735760	-7.585550	-0.619510
C	-1.982000	-5.965460	0.598700
C	-0.656020	-6.454760	0.735130
H	-0.391530	-7.390720	0.249710
C	0.282250	-5.752760	1.463350
H	1.300420	-6.115830	1.556280
C	-0.066340	-4.547670	2.107770
H	0.677360	-4.012890	2.682670
C	-1.349340	-4.043050	1.995970
C	-2.318170	-4.729090	1.226360
C	-1.701010	-2.784940	2.684430
C	-3.821110	-0.866950	4.461890
C	-4.940080	-1.915670	4.614100
H	-4.555250	-2.934650	4.496180
H	-5.737550	-1.766560	3.879510
H	-5.375840	-1.840590	5.616410
C	-2.740720	-1.136620	5.522620
H	-2.347590	-2.153670	5.448870
H	-3.183600	-1.016860	6.519080
H	-1.903770	-0.443660	5.426830
C	-4.437180	0.529770	4.689010
H	-3.679790	1.313480	4.642190
H	-4.914370	0.563230	5.675120
H	-5.203240	0.751790	3.936130
C	-0.028090	-0.188420	-0.952650
C	1.077270	-0.694600	-1.700380
C	0.877350	-1.455470	-2.886360
C	2.410460	-0.468770	-1.267600
C	1.944660	-1.997270	-3.579600
H	-0.126290	-1.634740	-3.249960
C	3.483760	-0.974070	-1.983510
H	2.580450	0.127340	-0.382270
C	3.241360	-1.750830	-3.116830
H	1.785080	-2.596000	-4.468590
H	4.497000	-0.766180	-1.677340
Br	4.713520	-2.505500	-4.052400
C	-1.376280	-0.357460	-1.541770
O	-1.953180	0.493690	-2.182330
O	-1.895330	-1.580420	-1.243000
C	-3.210800	-1.866330	-1.701110
H	-3.745810	-2.342900	-0.883520
H	-3.723370	-0.956770	-2.008220
C	-3.158270	-2.836590	-2.889410
Cl	-4.826150	-3.452700	-3.170100
Cl	-2.063860	-4.210040	-2.539460
Cl	-2.591310	-1.987770	-4.376530

Rh-2a

6-31G(d)-SDD(Rh) SCF energy in gas phase (au):	-16581.998320
6-31G(d)-SDD(Rh) enthalpy in gas phase (au):	-16580.390630
6-31G(d)-SDD(Rh) free energy in gas phase (au):	-16580.640800
6-311+G(d,p)-SDD(Rh) SCF energy in implicit solvent (DCM) (au):	-16586.806100
6-311+G(d,p)-SDD(Rh) enthalpy in implicit solvent (DCM) (au):	-16585.198410

```
6-311+G(d,p)-SDD(Rh) free energy in implicit solvent (DCM) (au):      -16585.448580
6-311+G(d,p)-SDD(Rh) SCF energy in implicit solvent (HFIP) (au):      -16586.814990
6-311+G(d,p)-SDD(Rh) enthalpy in implicit solvent (HFIP) (au):        -16585.207300
6-311+G(d,p)-SDD(Rh) free energy in implicit solvent (HFIP) (au):     -16585.457460
```

Cartesian coordinates

ATOM	X	Y	Z
Rh	0.050320	1.548100	2.514500
Rh	-0.019060	0.327410	0.376770
Cl	-1.414910	3.530830	-3.858570
Cl	-1.145570	1.650390	-6.366320
Cl	4.013580	0.536920	-5.015780
Cl	1.533690	0.124140	-6.916510
Cl	-6.659160	2.190560	-4.103980
Cl	-8.682940	-0.118160	-3.379430
Cl	-6.326160	-0.681180	1.497980
Cl	-8.492370	-1.565850	-0.609680
Cl	6.626850	1.915290	0.021840
Cl	8.341300	0.644230	-2.298320
Cl	6.411720	-4.293830	-1.025880
Cl	8.264330	-2.447370	-2.798730
Cl	-4.477310	-6.063240	-0.424080
Cl	-2.296220	-7.119590	-2.439870
Cl	1.557180	-4.383690	0.269020
Cl	0.693790	-6.227480	-2.139110
O	1.471920	1.570370	-0.208240
O	1.549770	2.739090	1.725200
O	4.334750	1.873660	-2.187280
O	0.447110	4.161670	-1.422800
O	-1.284100	2.859100	1.669960
O	-1.440040	1.665810	-0.252000
O	-4.047630	1.479030	1.507860
O	-4.276320	3.565870	-2.578380
O	1.421570	0.155930	3.166380
O	1.454410	-0.874930	1.148070
O	4.275120	-3.732300	1.199760
O	4.190360	0.824510	1.728180
O	-1.516390	-0.816640	1.203130
O	-1.425070	0.276400	3.181780
O	-0.021300	-2.919720	2.566210
O	-4.463130	-3.654110	1.671040
N	2.620230	3.335330	-1.597330
N	-3.964630	2.803010	-0.403310
N	4.073240	-1.496690	1.803200
N	-2.334220	-3.020450	2.341520
C	-5.664320	1.798820	-1.605150
C	-6.599680	1.421770	-2.556960
C	-7.493620	0.377670	-2.227340
C	-7.414830	-0.268010	-0.977840
C	-6.447840	0.125780	-0.024220
C	-5.591840	1.162730	-0.361410
C	-4.471310	1.783850	0.413400
C	1.901950	2.514420	0.542220
C	1.297850	3.557440	-2.039870
C	-2.770850	3.599000	-0.091340

H	-2.354880	3.870800	-1.066460
C	-1.744860	2.635930	0.521360
C	1.167110	2.849620	-3.347050
C	0.075220	2.731440	-4.193780
C	0.205110	1.879060	-5.312720
C	1.409260	1.192010	-5.558670
C	4.583870	-0.268040	1.373960
C	5.637160	-0.595630	0.360070
C	6.495380	0.233740	-0.346930
C	7.300610	-0.355620	-1.348810
C	7.267600	-1.747980	-1.571740
C	6.434590	-2.582440	-0.792180
C	5.622540	-1.978160	0.155380
C	4.605510	-2.572490	1.078830
C	2.516720	1.347530	-4.696920
C	2.362920	2.168450	-3.590240
C	2.969460	-1.667480	2.757250
H	2.565980	-2.658610	2.531420
C	1.854480	-0.695050	2.346400
C	3.273160	2.408850	-2.432470
C	2.969700	3.361970	-0.164670
H	3.878600	2.753280	-0.109720
C	-4.584740	2.839070	-1.659450
C	-3.257120	-3.761050	1.592950
C	-2.444110	-4.623540	0.673120
C	-2.836350	-5.537690	-0.294210
C	-1.844050	-6.038370	-1.166710
C	-0.497520	-5.652590	-1.020230
C	-0.101470	-4.799010	0.033470
C	-1.097800	-4.295910	0.858210
C	-1.009040	-3.340330	2.004040
C	-2.717150	-1.768030	3.012000
H	-3.720980	-1.554230	2.626070
C	-1.806360	-0.663080	2.436460
C	3.332130	4.740730	0.428300
C	2.129910	5.683560	0.655660
C	4.053820	4.507190	1.781840
C	4.327000	5.432090	-0.534410
H	1.406310	5.207330	1.323920
H	1.613660	5.868100	-0.290050
C	2.610890	7.017380	1.255800
H	4.909900	3.836670	1.629370
H	3.378690	4.005610	2.479520
C	4.530660	5.844150	2.373570
H	3.842390	5.605530	-1.502860
H	5.183880	4.769500	-0.721320
C	4.806380	6.770170	0.055970
H	1.740680	7.668280	1.412370
C	3.315350	6.762100	2.599890
C	3.593300	7.692260	0.281150
H	5.031830	5.650960	3.330540
C	5.513320	6.517500	1.399630
H	5.504500	7.241530	-0.647610
H	2.617720	6.292890	3.306500
H	3.637680	7.713190	3.044880

H	3.092990	7.896670	-0.675070
H	3.926150	8.657830	0.684920
H	5.874670	7.466000	1.818960
H	6.392160	5.875680	1.248410
C	-3.065440	4.910640	0.672350
C	-3.675080	4.693140	2.075270
C	-1.746240	5.715070	0.792580
C	-4.061760	5.754750	-0.161670
H	-3.008690	4.078190	2.683520
H	-4.624630	4.149800	1.986510
C	-3.922480	6.052630	2.756590
H	-1.320790	5.870930	-0.206930
H	-1.012050	5.145600	1.365970
C	-2.005240	7.066390	1.478530
H	-5.011870	5.215060	-0.264590
H	-3.668750	5.902740	-1.174950
C	-4.321200	7.112700	0.515260
H	-4.341030	5.877950	3.755880
C	-2.591530	6.818470	2.880950
C	-4.912370	6.879660	1.917270
H	-1.053890	7.607560	1.565430
C	-2.998090	7.889040	0.639870
H	-5.030430	7.684890	-0.096320
H	-1.881140	6.240240	3.486960
H	-2.755010	7.774560	3.396270
H	-5.872310	6.351100	1.838750
H	-5.113270	7.842500	2.405970
H	-3.177870	8.864970	1.110500
H	-2.578720	8.081940	-0.356680
C	-2.832970	-1.870820	4.552040
C	-1.468610	-1.991170	5.265240
C	-3.588330	-0.627110	5.084440
C	-3.676400	-3.122830	4.898490
H	-0.847710	-1.124580	5.027210
H	-0.935500	-2.875670	4.901070
C	-1.673800	-2.091090	6.787090
H	-4.565830	-0.555330	4.585990
H	-3.033950	0.281170	4.838350
C	-3.783500	-0.732700	6.607200
H	-3.167270	-4.023070	4.532950
H	-4.644920	-3.074420	4.384920
C	-3.880420	-3.233170	6.420220
H	-0.692070	-2.165010	7.272860
C	-2.406950	-0.834760	7.291270
C	-2.509720	-3.341260	7.113330
H	-4.306290	0.164570	6.961800
C	-4.616640	-1.983700	6.935020
H	-4.476270	-4.129430	6.634950
H	-1.810940	0.060970	7.070560
H	-2.531180	-0.881810	8.381540
H	-1.984510	-4.243640	6.771860
H	-2.643790	-3.436700	8.199230
H	-4.776770	-2.060840	8.018810
H	-5.607170	-1.909640	6.466170
C	3.422100	-1.677620	4.235610

C	4.021790	-0.339560	4.723170
C	2.204940	-2.051370	5.117430
C	4.494610	-2.782180	4.410700
H	3.297420	0.466710	4.588110
H	4.902970	-0.084460	4.122030
C	4.431060	-0.455270	6.203920
H	1.781000	-3.002840	4.770780
H	1.424460	-1.296250	5.010450
C	2.624460	-2.161810	6.592230
H	5.375930	-2.545110	3.800650
H	4.104670	-3.741600	4.048100
C	4.916100	-2.899550	5.886450
H	4.842140	0.507040	6.534290
C	3.198800	-0.810770	7.056530
C	5.496740	-1.554830	6.360290
H	1.741950	-2.415040	7.193940
C	3.691040	-3.259340	6.745840
H	5.676740	-3.685340	5.977920
H	2.436010	-0.026610	6.959620
H	3.477660	-0.863250	8.117560
H	6.388730	-1.300640	5.771470
H	5.813450	-1.630630	7.409140
H	3.986520	-3.358770	7.798890
H	3.281340	-4.228690	6.431460
C	-0.031570	-0.634110	-1.361470
C	1.064180	-1.311410	-1.975700
C	0.851150	-2.281450	-2.993120
C	2.397480	-0.960800	-1.642590
C	1.917380	-2.887300	-3.633000
H	-0.162300	-2.568600	-3.256870
C	3.461630	-1.501900	-2.342090
H	2.569440	-0.200350	-0.896360
C	3.217740	-2.472720	-3.316270
H	1.756000	-3.645570	-4.389490
H	4.468240	-1.153750	-2.163310
Br	4.684730	-3.205280	-4.258710
C	-1.227040	-0.462260	-2.209950
O	-1.180550	0.300280	-3.155640
O	-2.329970	-1.138820	-1.837270
C	-3.456200	-0.954190	-2.698050
H	-4.103190	-0.180030	-2.284380
H	-3.129470	-0.661670	-3.696140
C	-4.232610	-2.263180	-2.767630
Cl	-5.602780	-1.999470	-3.901620
Cl	-4.846000	-2.709130	-1.158910
Cl	-3.167630	-3.573790	-3.400280

TS1

6-31G(d)-SDD(Rh) SCF energy in gas phase (au):	-9149.924598
6-31G(d)-SDD(Rh) enthalpy in gas phase (au):	-9148.257785
6-31G(d)-SDD(Rh) free energy in gas phase (au):	-9148.483498
6-311+G(d,p)-SDD(Rh) SCF energy in implicit solvent (DCM) (au):	-9154.213231
6-311+G(d,p)-SDD(Rh) enthalpy in implicit solvent (DCM) (au):	-9152.546418
6-311+G(d,p)-SDD(Rh) free energy in implicit solvent (DCM) (au):	-9152.772130
6-311+G(d,p)-SDD(Rh) SCF energy in implicit solvent (HFIP) (au):	-9154.226347

6-311+G(d,p)–SDD(Rh) enthalpy in implicit solvent (HFIP) (au): -9152.559534
6-311+G(d,p)–SDD(Rh) free energy in implicit solvent (HFIP) (au): -9152.785246
imaginary frequency (cm^{-1}): -71.76

Cartesian coordinates

ATOM	X	Y	Z
Rh	-0.162070	-0.330610	-1.086060
Rh	-0.358950	-0.793610	-3.492260
O	1.128840	1.178760	-1.615560
O	0.890620	0.809350	-3.837570
O	2.985450	4.423450	-1.117540
O	3.738080	0.259030	-2.830180
O	1.441170	-1.594150	-1.120380
O	1.239200	-2.081510	-3.322750
O	4.883440	-3.157370	-0.123290
O	0.541250	-4.302660	-1.003910
O	-1.451660	-1.890030	-0.801110
O	-1.590090	-2.359800	-3.016050
O	-3.243880	-4.759800	1.053400
O	-4.186660	-1.290690	-1.766350
O	-1.781300	0.912620	-1.282890
O	-1.948020	0.516220	-3.508320
O	-4.993630	2.910490	-0.387030
O	-1.009180	3.443580	-2.569530
N	3.469250	2.407170	-2.101810
N	2.726120	-3.777540	-0.590740
N	-3.820600	-3.110140	-0.432660
N	-3.039810	3.234470	-1.545000
C	1.382880	1.406060	-2.847340
C	2.333570	2.589360	-3.038880
H	1.783810	3.432280	-2.619880
C	3.676180	3.408130	-1.142190
C	4.763650	3.186320	-0.162010
C	5.047810	4.166460	0.772410
H	4.475030	5.086430	0.749210
C	6.056210	3.962320	1.736230
H	6.262000	4.739720	2.465050
C	6.767200	2.779080	1.763680
H	7.532800	2.614610	2.516820
C	6.507660	1.754260	0.818440
C	7.185950	0.506460	0.824720
H	7.941610	0.326640	1.583850
C	6.868830	-0.475310	-0.091790
H	7.359240	-1.441530	-0.056890
C	5.868520	-0.250900	-1.060430
H	5.599660	-1.030270	-1.761840
C	5.191630	0.955910	-1.097670
C	5.492830	1.972860	-0.160540
C	4.102090	1.150210	-2.075980
C	2.730530	3.010900	-4.481090
C	3.673880	4.224940	-4.374380
H	3.901650	4.602690	-5.377130
H	4.623220	3.956140	-3.897810
H	3.222190	5.037550	-3.795660
C	3.422450	1.908960	-5.299750

H	2.808210	1.009230	-5.355250
H	4.390550	1.633460	-4.872470
H	3.599070	2.279360	-6.317080
C	1.440680	3.459360	-5.200510
H	0.758680	2.621550	-5.353650
H	1.696520	3.884960	-6.177640
H	0.908760	4.224800	-4.623860
C	1.739710	-2.240890	-2.177300
C	2.875440	-3.246810	-1.965290
H	3.776820	-2.632510	-1.902590
C	3.808800	-3.597680	0.279350
C	3.595880	-3.961480	1.698060
C	4.663650	-3.943010	2.576490
H	5.647710	-3.697350	2.194510
C	4.461610	-4.207530	3.946180
H	5.309160	-4.189950	4.623180
C	3.191960	-4.446460	4.431600
H	3.034010	-4.622120	5.492820
C	2.071780	-4.461500	3.562010
C	0.740260	-4.661600	4.013420
H	0.565480	-4.807030	5.076680
C	-0.313280	-4.665200	3.123220
H	-1.333170	-4.798550	3.468200
C	-0.083610	-4.503300	1.741740
H	-0.915690	-4.523780	1.051560
C	1.201220	-4.305900	1.270600
C	2.292090	-4.248930	2.169240
C	1.429770	-4.149070	-0.176710
C	3.153110	-4.299410	-3.079570
C	4.240900	-5.261550	-2.565630
H	5.136750	-4.721890	-2.242470
H	4.524050	-5.955290	-3.365130
H	3.879990	-5.856630	-1.719890
C	1.922290	-5.117110	-3.505010
H	1.548680	-5.737220	-2.687740
H	2.203870	-5.776360	-4.335640
H	1.107500	-4.470510	-3.835100
C	3.714400	-3.540820	-4.300640
H	4.606280	-2.961540	-4.030400
H	2.975410	-2.853930	-4.716790
H	4.003120	-4.257130	-5.078390
C	-1.899030	-2.532020	-1.805680
C	-2.855520	-3.659450	-1.414990
H	-2.238200	-4.341620	-0.828750
C	-3.912930	-3.763150	0.805520
C	-4.878510	-3.213360	1.785290
C	-5.085410	-3.874610	2.982730
H	-4.538330	-4.792120	3.169010
C	-5.991290	-3.359480	3.933000
H	-6.147710	-3.892050	4.865860
C	-6.667670	-2.181970	3.687380
H	-7.353740	-1.777210	4.426570
C	-6.467230	-1.468150	2.478260
C	-7.082500	-0.217920	2.206500
H	-7.748840	0.209230	2.950960

C	-6.820560	0.458100	1.032170
H	-7.264270	1.429170	0.841350
C	-5.955730	-0.098630	0.066410
H	-5.752280	0.432300	-0.853640
C	-5.340340	-1.315400	0.298810
C	-5.570210	-2.009540	1.510560
C	-4.420980	-1.873750	-0.716190
C	-3.497180	-4.515850	-2.544780
C	-4.411380	-5.567630	-1.886250
H	-3.869210	-6.171380	-1.151720
H	-4.811600	-6.237260	-2.655560
H	-5.263060	-5.099900	-1.379660
C	-4.321580	-3.706600	-3.559520
H	-5.194420	-3.241970	-3.092130
H	-4.682830	-4.381950	-4.344970
H	-3.724520	-2.918840	-4.020590
C	-2.355130	-5.257350	-3.271210
H	-1.734760	-5.819240	-2.563210
H	-1.708190	-4.563530	-3.809930
H	-2.779420	-5.968700	-3.989300
C	-2.270400	1.107900	-2.444890
C	-3.409710	2.130420	-2.463100
H	-4.224790	1.628080	-1.940500
C	-3.923090	3.502550	-0.488440
C	-3.504800	4.525240	0.496320
C	-4.373990	4.889240	1.509080
H	-5.359170	4.437760	1.534640
C	-3.968050	5.806950	2.499880
H	-4.660480	6.083240	3.288880
C	-2.692740	6.336880	2.480510
H	-2.374320	7.029830	3.254870
C	-1.778380	5.993130	1.452100
C	-0.455640	6.504990	1.389050
H	-0.119850	7.185590	2.167120
C	0.393090	6.140620	0.364170
H	1.409920	6.516580	0.326170
C	-0.042990	5.259480	-0.646120
H	0.635260	4.968440	-1.436700
C	-1.324450	4.739330	-0.612940
C	-2.207090	5.086570	0.436830
C	-1.749940	3.781760	-1.655530
C	-3.990420	2.574290	-3.838280
C	-5.065800	3.647490	-3.580210
H	-4.629460	4.562820	-3.165280
H	-5.833090	3.292010	-2.885060
H	-5.552890	3.911590	-4.525310
C	-2.947210	3.139440	-4.816890
H	-2.507960	4.069320	-4.447550
H	-3.438070	3.353950	-5.774280
H	-2.137500	2.429400	-4.990750
C	-4.678520	1.344630	-4.468150
H	-3.956340	0.563670	-4.709890
H	-5.192910	1.644350	-5.388440
H	-5.425370	0.916780	-3.788330
C	0.011720	-0.050680	1.021120

C	1.260360	-0.398740	1.711090
C	-1.244060	-0.432580	1.714400
C	2.490070	-0.149890	1.068430
C	1.295950	-0.884570	3.036990
O	-2.174690	0.568730	1.841520
O	-1.461030	-1.573060	2.072650
C	3.697100	-0.318830	1.735310
H	2.485450	0.206170	0.048130
C	2.498390	-1.049680	3.715220
H	0.371860	-1.134290	3.541840
C	-3.326280	0.258340	2.604660
C	3.688880	-0.733190	3.063260
H	4.632290	-0.125960	1.237960
H	2.511260	-1.405670	4.738130
H	-3.397640	-0.816360	2.770600
H	-4.198930	0.625310	2.064460
C	-3.266350	0.970090	3.965420
Br	5.343850	-0.806390	4.011490
Cl	-1.777710	0.503640	4.865890
Cl	-4.709960	0.477600	4.913970
Cl	-3.287390	2.752180	3.738280
C	3.527770	5.707340	3.861720
C	3.080830	4.244000	3.849210
H	2.749960	6.355830	4.283120
H	3.736970	6.064480	2.845880
H	3.887910	3.613310	3.455850
H	2.897250	3.904470	4.877660
C	1.819180	4.024520	3.010820
C	1.426160	2.530660	2.939940
H	1.976190	4.389230	1.988570
H	0.988710	4.612450	3.423870
H	2.220880	1.995460	2.410380
H	1.365330	2.120760	3.955780
C	0.125890	2.346090	2.240290
C	-0.003780	2.124160	0.900490
H	-0.771950	2.400850	2.844990
H	-0.977240	2.151920	0.432080
H	0.848720	2.195590	0.236050
H	4.435730	5.845520	4.459820

TS2

6-31G(d)-SDD(Rh) SCF energy in gas phase (au): -9149.922014
6-31G(d)-SDD(Rh) enthalpy in gas phase (au): -9148.255329
6-31G(d)-SDD(Rh) free energy in gas phase (au): -9148.481674
6-311+G(d,p)-SDD(Rh) SCF energy in implicit solvent (DCM) (au): -9154.211754
6-311+G(d,p)-SDD(Rh) enthalpy in implicit solvent (DCM) (au): -9152.545069
6-311+G(d,p)-SDD(Rh) free energy in implicit solvent (DCM) (au): -9152.771414
6-311+G(d,p)-SDD(Rh) SCF energy in implicit solvent (HFIP) (au): -9154.220148
6-311+G(d,p)-SDD(Rh) enthalpy in implicit solvent (HFIP) (au): -9152.553463
6-311+G(d,p)-SDD(Rh) free energy in implicit solvent (HFIP) (au): -9152.779808
imaginary frequency (cm^{-1}): -111.32

Cartesian coordinates

ATOM	X	Y	Z
Rh	0.149280	0.017870	-1.043640

Rh	0.277150	0.057340	-3.499370
O	1.593800	-1.426430	-1.124730
O	1.666430	-1.457480	-3.388850
O	4.489770	-3.585250	0.071140
O	0.383880	-3.997490	-1.886060
O	-1.340610	-1.380790	-1.257530
O	-1.187780	-1.368470	-3.521030
O	-3.832690	-4.302840	-0.416290
O	-3.775760	-0.199660	-2.421000
O	-1.250230	1.508200	-1.198900
O	-1.128090	1.564950	-3.460660
O	-4.306660	3.656720	-0.274260
O	-0.023150	4.080770	-1.814060
O	1.646470	1.422580	-1.067920
O	1.736250	1.491810	-3.332500
O	3.900900	4.258400	0.237450
O	4.244960	0.337110	-2.078130
N	2.470310	-3.897590	-0.967940
N	-3.907070	-2.309270	-1.551280
N	-2.194550	3.970320	-1.114080
N	4.193310	2.362410	-1.021440
C	1.970000	-1.897020	-2.248250
C	2.942630	-3.070220	-2.103640
H	3.855730	-2.612910	-1.718270
C	3.343560	-4.030110	0.117910
C	2.823460	-4.704510	1.327340
C	3.662650	-4.890160	2.412220
H	4.700460	-4.589340	2.324200
C	3.162220	-5.431380	3.613570
H	3.830480	-5.567850	4.458000
C	1.827430	-5.766030	3.724130
H	1.437260	-6.164920	4.657040
C	0.937530	-5.580600	2.635220
C	-0.453970	-5.851920	2.719650
H	-0.854890	-6.241750	3.652030
C	-1.286180	-5.608140	1.646730
H	-2.353690	-5.789690	1.716830
C	-0.763910	-5.100160	0.439430
H	-1.429040	-4.894400	-0.388540
C	0.587570	-4.832870	0.321810
C	1.456410	-5.059040	1.414230
C	1.102360	-4.230560	-0.922660
C	3.366040	-3.853790	-3.379400
C	4.281490	-5.012410	-2.939360
H	4.677110	-5.524970	-3.823270
H	3.734180	-5.750290	-2.342980
H	5.126320	-4.653910	-2.341840
C	2.195650	-4.421960	-4.198940
H	1.493340	-3.637960	-4.486300
H	1.642680	-5.181020	-3.641150
H	2.592500	-4.889290	-5.108910
C	4.191180	-2.890900	-4.259050
H	3.581430	-2.062100	-4.622390
H	4.593510	-3.434410	-5.121680
H	5.037660	-2.471160	-3.701370

C	-1.697730	-1.735300	-2.428850
C	-2.818750	-2.774820	-2.441000
H	-2.392830	-3.632240	-1.920030
C	-4.365210	-3.209920	-0.575790
C	-5.545440	-2.792650	0.214360
C	-6.145330	-3.695590	1.074040
H	-5.732710	-4.695800	1.145740
C	-7.271090	-3.315660	1.832630
H	-7.738590	-4.038110	2.494230
C	-7.762640	-2.028920	1.755830
H	-8.612270	-1.727480	2.362540
C	-7.155960	-1.069350	0.905710
C	-7.573700	0.285380	0.845060
H	-8.397460	0.607290	1.476570
C	-6.935210	1.185820	0.015700
H	-7.239900	2.226880	-0.005830
C	-5.875440	0.764850	-0.814100
H	-5.379500	1.470710	-1.467750
C	-5.441670	-0.548460	-0.776780
C	-6.051910	-1.476250	0.100340
C	-4.322240	-0.972380	-1.643800
C	-3.317140	-3.296600	-3.822180
C	-4.424070	-4.341210	-3.576240
H	-4.079620	-5.151990	-2.926980
H	-4.733580	-4.774280	-4.533770
H	-5.311570	-3.891290	-3.117200
C	-3.876490	-2.194000	-4.736800
H	-4.773960	-1.734480	-4.311390
H	-4.155680	-2.636350	-5.700990
H	-3.141220	-1.407580	-4.910110
C	-2.135380	-4.009020	-4.513440
H	-1.691910	-4.766570	-3.857090
H	-1.350320	-3.305020	-4.791000
H	-2.492480	-4.511170	-5.420180
C	-1.538110	1.984410	-2.349320
C	-2.545180	3.133130	-2.285570
H	-3.477290	2.651390	-1.985120
C	-3.185560	4.137620	-0.135340
C	-2.816380	4.923870	1.063930
C	-3.786950	5.239050	1.998260
H	-4.806560	4.918060	1.817170
C	-3.445460	5.956060	3.163080
H	-4.219990	6.212190	3.879050
C	-2.133770	6.312040	3.406090
H	-1.868190	6.841500	4.317300
C	-1.109490	5.987290	2.480180
C	0.260480	6.286520	2.703510
H	0.542740	6.783490	3.628070
C	1.218490	5.943060	1.772070
H	2.268550	6.147230	1.952810
C	0.848390	5.316320	0.564810
H	1.608040	5.054390	-0.159630
C	-0.477700	5.010090	0.315670
C	-1.471540	5.312540	1.276210
C	-0.846780	4.337200	-0.946050

C	-2.866820	3.917070	-3.591190
C	-3.849000	5.050590	-3.237490
H	-4.743190	4.668090	-2.734500
H	-4.162490	5.562760	-4.153770
H	-3.383960	5.796140	-2.583170
C	-1.635750	4.520700	-4.287540
H	-1.158790	5.287910	-3.672780
H	-1.952680	4.988900	-5.227650
H	-0.889950	3.756680	-4.511890
C	-3.579810	2.942450	-4.552300
H	-4.459210	2.490080	-4.077920
H	-2.915600	2.136780	-4.868170
H	-3.918660	3.486920	-5.441160
C	2.122520	1.824700	-2.182690
C	3.223890	2.872570	-2.018700
H	2.728670	3.698500	-1.508450
C	4.453480	3.171760	0.093610
C	5.443220	2.665120	1.072210
C	5.827180	3.466930	2.132540
H	5.395910	4.458410	2.215210
C	6.761050	2.997600	3.079180
H	7.058140	3.640930	3.901540
C	7.286240	1.725640	2.972210
H	7.990290	1.357090	3.713190
C	6.905580	0.871130	1.906760
C	7.378650	-0.460950	1.775910
H	8.062400	-0.847760	2.526330
C	6.962180	-1.261240	0.731640
H	7.299720	-2.289060	0.656070
C	6.072800	-0.759200	-0.240330
H	5.738590	-1.387640	-1.055670
C	5.590680	0.534810	-0.142310
C	5.983440	1.363900	0.935760
C	4.637920	1.035310	-1.154270
C	3.871250	3.477310	-3.297840
C	4.925560	4.511320	-2.855950
H	5.757440	4.036750	-2.323570
H	4.494220	5.274180	-2.199920
H	5.340180	5.010560	-3.738580
C	4.545210	2.441630	-4.212890
H	5.398250	1.963570	-3.722700
H	4.918420	2.948840	-5.111140
H	3.845930	1.660980	-4.514480
C	2.763680	4.219950	-4.074750
H	2.021810	3.526210	-4.472600
H	3.210740	4.771080	-4.910200
H	2.242080	4.938980	-3.432630
C	-0.004610	-0.207400	1.023120
C	-1.273730	-0.486830	1.692060
C	1.168440	-0.959750	1.526860
C	-2.462560	0.117530	1.224050
C	-1.362110	-1.311510	2.839440
O	2.307480	-0.245690	1.754520
O	1.126910	-2.170690	1.623200
C	-3.661070	-0.022340	1.912160

H	-2.424560	0.732020	0.333600
C	-2.567620	-1.497500	3.504740
H	-0.483040	-1.829150	3.198570
C	3.419950	-1.008670	2.201200
C	-3.700260	-0.821260	3.055610
H	-4.548470	0.498090	1.577810
H	-2.620880	-2.129990	4.383350
H	3.262550	-2.068560	2.014530
H	4.297860	-0.659560	1.665180
C	3.651650	-0.803970	3.702540
Br	-5.311080	-0.953970	4.059960
Cl	2.208270	-1.291930	4.657500
Cl	5.052420	-1.833410	4.171490
Cl	4.035840	0.915350	4.057590
C	-4.166210	2.664810	4.694380
C	-2.768980	2.088070	4.927210
H	-4.142340	3.759320	4.672390
H	-4.573490	2.322080	3.735970
H	-2.840890	0.996510	5.009430
H	-2.375150	2.443190	5.890400
C	-1.781040	2.436680	3.809080
C	-0.424480	1.753670	4.008180
H	-2.213430	2.131770	2.852810
H	-1.639410	3.522300	3.754000
H	-0.592210	0.678240	4.201160
H	0.057840	2.127470	4.922360
C	0.556930	1.837680	2.886730
C	0.288070	2.025450	1.568470
H	1.590840	1.656740	3.160190
H	1.090950	2.140240	0.851300
H	-0.703100	2.280320	1.216620
H	-4.861630	2.352620	5.480980

TS3

6-31G(d)-SDD(Rh) SCF energy in gas phase (au): -16817.891080
6-31G(d)-SDD(Rh) enthalpy in gas phase (au): -16816.106100
6-31G(d)-SDD(Rh) free energy in gas phase (au): -16816.372230
6-311+G(d,p)-SDD(Rh) SCF energy in implicit solvent (DCM) (au): -16822.770730
6-311+G(d,p)-SDD(Rh) enthalpy in implicit solvent (DCM) (au): -16820.985760
6-311+G(d,p)-SDD(Rh) free energy in implicit solvent (DCM) (au): -16821.251880
6-311+G(d,p)-SDD(Rh) SCF energy in implicit solvent (HFIP) (au): -16822.779540
6-311+G(d,p)-SDD(Rh) enthalpy in implicit solvent (HFIP) (au): -16820.994560
6-311+G(d,p)-SDD(Rh) free energy in implicit solvent (HFIP) (au): -16821.260690
imaginary frequency (cm^{-1}): -73.44

Cartesian coordinates

ATOM	X	Y	Z
C	-4.565610	1.586810	0.548160
C	-1.218270	-3.844790	1.525170
C	4.443040	-0.624220	1.803630
C	1.166160	4.116250	-0.869680
N	-4.029230	2.774990	0.040080
N	-2.542500	-3.399080	1.532050
N	3.911300	-1.914800	1.930400
N	2.486770	3.893960	-0.445470

O	-1.435770	1.723300	0.100880
O	-1.567820	-1.004670	0.915280
O	1.366440	-1.111540	1.154500
O	1.505870	1.632100	0.333000
C	-5.644930	1.185070	-0.411850
C	-1.131820	-4.862440	0.429900
C	5.565870	-0.742310	0.819080
C	1.182010	3.993900	-2.358050
O	-1.480080	2.427360	2.254380
O	-1.668750	-0.397950	3.093100
O	1.162930	-0.503070	3.327120
O	1.350950	2.331460	2.480970
C	-6.499440	0.092350	-0.398810
C	-0.039750	-5.571480	-0.048010
C	6.440310	0.220660	0.336150
C	0.145160	4.085430	-3.275170
O	-4.191460	1.009040	1.547050
O	-0.335120	-3.444680	2.254970
O	4.021190	0.371190	2.352860
O	0.223320	4.331050	-0.137200
C	-7.390930	-0.061650	-1.486230
C	-0.243950	-6.427890	-1.152220
C	7.337350	-0.158310	-0.689850
C	0.444540	3.804530	-4.627100
O	-4.273180	4.082260	-1.869720
O	-4.449280	-3.700000	0.236840
O	4.185110	-3.982060	0.901400
O	4.472700	3.177310	-1.418180
C	-7.408310	0.876270	-2.536600
C	-1.512420	-6.531980	-1.756080
C	7.347400	-1.479700	-1.182480
C	1.763890	3.518880	-5.027000
C	-6.529490	1.983110	-2.529770
C	-2.606750	-5.787610	-1.260790
C	6.459250	-2.448700	-0.662010
C	2.813250	3.488820	-4.083600
C	-5.661940	2.113520	-1.456980
C	-2.389440	-4.960090	-0.170260
C	5.582720	-2.054770	0.338000
C	2.487390	3.688650	-2.751870
C	-4.600320	3.136040	-1.187310
C	-3.302610	-3.981920	0.506410
C	4.507820	-2.822300	1.045140
C	3.324290	3.551750	-1.519970
C	-2.896880	3.504300	0.627090
C	-3.024400	-2.240140	2.296120
C	2.713520	-2.254910	2.711430
C	2.826660	3.477290	0.922920
C	-1.850900	2.463430	1.051980
C	-1.994210	-1.114190	2.111340
C	1.655450	-1.188610	2.397490
C	1.800700	2.400930	1.308720
C	-3.316190	4.564780	1.675340
C	-3.479040	-2.580280	3.735470
C	2.999910	-2.580360	4.195150

C	3.088780	4.632680	1.913090
C	-3.992280	3.968960	2.931090
C	-2.318040	-2.970030	4.678600
C	3.567830	-1.391720	5.002600
C	1.821550	5.420560	2.312960
C	-4.362550	5.095280	3.914860
C	-2.863420	-3.290610	6.082150
C	3.814540	-1.817460	6.462310
C	2.188680	6.546230	3.296660
C	-5.352920	6.063260	3.243760
C	-3.843520	-4.474460	5.995650
C	4.837540	-2.966840	6.498950
C	3.179740	7.513390	2.624090
C	-4.698100	6.674320	1.992050
C	-5.015010	-4.101490	5.068500
C	4.286720	-4.165730	5.705800
C	4.456030	6.746680	2.229920
C	-4.314910	5.548490	1.014370
C	-4.470540	-3.768740	3.667890
C	4.027330	-3.738910	4.249750
C	4.089240	5.613940	1.254730
C	-2.060660	5.374550	2.088640
C	-4.237160	-1.362160	4.322320
C	1.687960	-3.078310	4.853540
C	3.755100	4.052110	3.186580
C	-2.443580	6.489890	3.074840
C	-4.772910	-1.692400	5.725920
C	1.945800	-3.494650	6.310940
C	4.115520	5.183640	4.164480
C	-3.436600	7.453660	2.403710
C	-5.750540	-2.876960	5.640600
C	2.970530	-4.641410	6.346420
C	5.104960	6.151180	3.492000
C	-3.091310	5.863250	4.323390
C	-3.594110	-2.059030	6.647220
C	2.491560	-2.287240	7.095370
C	2.834560	5.943430	4.557130
Cl	-8.457640	-1.420090	-1.525740
Cl	1.087340	-7.341250	-1.776400
Cl	8.416500	1.015090	-1.351390
Cl	-0.829980	3.802980	-5.796560
Cl	-6.480010	-1.051280	0.895380
Cl	1.539560	-5.347320	0.612820
Cl	6.440250	1.827680	0.965020
Cl	-1.457560	4.491580	-2.792090
Cl	-8.496430	0.663660	-3.862860
Cl	-1.731830	-7.564550	-3.126690
Cl	8.451210	-1.923140	-2.435030
Cl	2.108120	3.173870	-6.690670
Cl	-6.518450	3.118950	-3.831710
Cl	-4.168110	-5.906030	-1.997620
Cl	6.445170	-4.064680	-1.276180
Cl	4.446760	3.204550	-4.571350
Rh	-0.166180	0.987370	2.860430
Rh	-0.015960	0.282270	0.504300

H	-2.439220	4.032530	-0.213180
H	-4.825900	4.650730	4.804910
H	-5.405830	7.350150	1.495140
H	-1.534680	7.034050	3.363540
H	-3.910690	-1.907310	1.745970
H	2.333840	-3.168090	2.246230
H	3.773750	2.938990	0.805270
H	-4.901010	3.425970	2.640950
H	-3.326500	3.248410	3.409970
H	-1.791110	-3.844780	4.281890
H	-1.590520	-2.156330	4.730440
H	4.512590	-1.056560	4.557320
H	2.876670	-0.547180	4.961550
H	1.091360	4.744670	2.765700
H	1.349770	5.846800	1.422120
H	-2.022530	-3.553890	6.737060
H	4.205140	-0.957650	7.021180
H	1.274180	7.087410	3.573080
H	-5.641400	6.858590	3.944140
H	-6.271840	5.528740	2.966190
H	-4.221720	-4.728720	6.994860
H	-3.326030	-5.363690	5.610330
H	5.039200	-3.263430	7.537000
H	5.791660	-2.635020	6.067030
H	3.431680	8.335630	3.307290
H	2.720090	7.962630	1.733280
H	-5.709050	-4.947950	4.989060
H	5.018910	-4.983270	5.712240
H	5.160650	7.428710	1.737010
H	-3.876100	5.969540	0.101710
H	-5.223330	5.011590	0.712380
H	-3.965020	-4.651510	3.256210
H	-5.296250	-3.527850	2.985430
H	4.972430	-3.420690	3.790850
H	3.659560	-4.590820	3.663730
H	4.992050	5.070920	0.942920
H	3.644930	6.039140	0.346380
H	-1.322550	4.713600	2.546080
H	-1.593390	5.803300	1.193400
H	-5.067430	-1.093440	3.655460
H	-3.573400	-0.496730	4.367730
H	0.930810	-2.292110	4.820220
H	1.292240	-3.926590	4.281320
H	4.660110	3.496570	2.904440
H	3.081080	3.339230	3.666980
H	-5.292150	-0.811440	6.124010
H	0.999430	-3.828120	6.756570
H	4.575290	4.747110	5.060260
H	-3.704770	8.266600	3.091890
H	-2.973850	7.915470	1.521200
H	-6.153850	-3.111150	6.634890
H	-6.603090	-2.615500	4.999130
H	2.579590	-5.512010	5.802650
H	3.150460	-4.960220	7.381870
H	5.383370	6.954750	4.187000

H	6.028780	5.619530	3.225870
H	-3.342850	6.645890	5.051900
H	-2.381600	5.181450	4.810900
H	-2.899230	-1.211900	6.721480
H	-3.959370	-2.269710	7.661410
H	1.758390	-1.469690	7.082380
H	2.653720	-2.561570	8.146490
H	2.129790	5.260140	5.049670
H	3.073160	6.738360	5.276660
C	0.178320	-0.159990	-1.533130
C	1.446220	-0.093520	-2.245260
C	-1.025130	0.281210	-2.283030
C	2.648260	-0.323680	-1.541100
C	1.535100	0.144810	-3.640160
O	-2.116180	-0.515490	-2.117610
O	-1.054840	1.330080	-2.895640
C	3.870610	-0.330400	-2.194170
H	2.600020	-0.517260	-0.479400
C	2.758750	0.145250	-4.299390
H	0.635160	0.338880	-4.209940
C	-3.304470	-0.111510	-2.773590
C	3.920920	-0.110810	-3.569230
H	4.784720	-0.505910	-1.651180
H	2.811170	0.331360	-5.365750
H	-3.198340	0.896090	-3.178670
H	-4.113880	-0.156920	-2.046120
C	-3.631800	-1.089830	-3.906880
Br	5.603610	-0.208120	-4.441680
Cl	-2.303350	-1.104980	-5.122870
Cl	-5.149340	-0.530890	-4.684380
Cl	-3.865150	-2.739780	-3.244130
C	2.047130	-6.218370	-5.602660
C	2.285160	-5.034330	-4.663000
H	1.458920	-5.916930	-6.477840
H	1.495860	-7.016520	-5.091270
H	2.898010	-5.357520	-3.810690
H	2.862250	-4.254320	-5.178970
C	0.979880	-4.435530	-4.135320
C	1.213760	-3.262050	-3.168100
H	0.404880	-5.218280	-3.627580
H	0.356820	-4.094220	-4.973630
H	1.940440	-3.535800	-2.393560
H	1.659190	-2.425750	-3.732790
C	-0.047880	-2.786100	-2.540150
C	-0.175520	-2.441370	-1.236590
H	-0.911600	-2.689190	-3.193870
H	-1.138970	-2.200710	-0.813540
H	0.646730	-2.566580	-0.539780
H	2.992140	-6.639330	-5.963000

TS4

6-31G(d)-SDD(Rh) SCF energy in gas phase (au):	-16817.896490
6-31G(d)-SDD(Rh) enthalpy in gas phase (au):	-16816.111390
6-31G(d)-SDD(Rh) free energy in gas phase (au):	-16816.376590
6-311+G(d,p)-SDD(Rh) SCF energy in implicit solvent (DCM) (au):	-16822.773340

6-311+G(d,p)–SDD(Rh) enthalpy in implicit solvent (DCM) (au): -16820.988240
6-311+G(d,p)–SDD(Rh) free energy in implicit solvent (DCM) (au): -16821.253450
6-311+G(d,p)–SDD(Rh) SCF energy in implicit solvent (HFIP) (au): -16822.781310
6-311+G(d,p)–SDD(Rh) enthalpy in implicit solvent (HFIP) (au): -16820.996220
6-311+G(d,p)–SDD(Rh) free energy in implicit solvent (HFIP) (au): -16821.261420
imaginary frequency (cm^{-1}): -18.71 (The very low imaginary frequency is along the reaction pathway, corresponding to bond formation).

Cartesian coordinates

ATOM	X	Y	Z
C	-4.742650	0.607670	0.893500
C	-0.689640	-4.395390	0.113460
C	4.482320	-0.854510	1.185370
C	0.580720	4.284750	0.527230
N	-4.327660	1.938860	0.778240
N	-2.065350	-4.200820	0.312500
N	4.152210	-2.211630	1.120950
N	1.922070	4.047640	0.840490
O	-1.662560	1.163870	0.544110
O	-1.403920	-1.682660	0.516130
O	1.520820	-1.455600	0.664310
O	1.263180	1.444250	0.678530
C	-5.805700	0.424560	-0.147030
C	-0.529370	-4.760550	-1.327870
C	5.608340	-0.662820	0.214610
C	0.592130	5.123670	-0.711490
O	-1.697640	1.195600	2.809370
O	-1.520520	-1.757030	2.774870
O	1.311840	-1.557010	2.916900
O	1.132970	1.370430	2.936600
C	-6.564100	-0.692210	-0.468350
C	0.624120	-4.901680	-2.085200
C	6.267870	0.491270	-0.181340
C	-0.458800	5.574630	-1.496940
O	-4.292030	-0.210630	1.667240
O	0.167040	-4.247640	0.959880
O	3.935440	-0.023960	1.881760
O	-0.371540	3.873350	1.156090
C	-7.464880	-0.585540	-1.552340
C	0.476650	-5.059960	-3.480680
C	7.262570	0.373200	-1.180730
C	-0.138330	6.344350	-2.638020
O	-4.685270	3.721710	-0.672200
O	-3.961230	-4.065370	-1.019730
O	4.769160	-4.060560	-0.148600
O	4.004660	4.548070	-0.064670
C	-7.589380	0.622650	-2.267120
C	-0.800010	-5.065680	-4.074890
C	7.566580	-0.880260	-1.747320
C	1.201510	6.658130	-2.942270
C	-6.811820	1.749510	-1.918040
C	-1.958390	-4.909160	-3.285430
C	6.862980	-2.039450	-1.348360
C	2.254070	6.183860	-2.127920
C	-5.924300	1.620780	-0.860950

C	-1.794710	-4.759080	-1.918470
C	5.886920	-1.899060	-0.374470
C	1.923440	5.399220	-1.033930
C	-4.937900	2.597050	-0.296340
C	-2.783790	-4.327650	-0.884110
C	4.913290	-2.899340	0.166640
C	2.797110	4.654070	-0.071070
C	-3.265540	2.555310	1.580140
C	-2.629880	-3.460190	1.448610
C	3.039700	-2.842600	1.842790
C	2.382200	3.148030	1.902930
C	-2.113300	1.545010	1.674280
C	-1.774840	-2.195190	1.624240
C	1.858210	-1.861160	1.824560
C	1.511180	1.881610	1.852940
C	-3.776170	3.188760	2.898930
C	-2.924820	-4.316540	2.701200
C	3.445400	-3.431610	3.218240
C	2.543110	3.843260	3.281460
C	-4.353070	2.163700	3.901670
C	-1.660470	-4.770240	3.465380
C	3.887170	-2.361080	4.242340
C	1.196680	4.226680	3.937830
C	-4.825250	2.883560	5.179050
C	-2.058610	-5.609790	4.692550
C	4.258240	-3.031130	5.578780
C	1.443440	4.899240	5.300110
C	-5.932050	3.892310	4.824510
C	-2.829330	-6.859980	4.231600
C	5.430050	-4.004420	5.362140
C	2.266540	6.183560	5.099010
C	-5.377430	4.928610	3.830620
C	-4.100660	-6.427890	3.477240
C	5.012190	-5.084850	4.348660
C	3.617920	5.825540	4.454390
C	-4.892310	4.208980	2.559020
C	-3.705650	-5.577640	2.256230
C	4.626660	-4.414930	3.017040
C	3.373220	5.139100	3.097910
C	-2.611020	3.968620	3.559330
C	-3.838220	-3.501700	3.652390
C	2.251130	-4.243910	3.782730
C	3.339240	2.905640	4.225030
C	-3.093620	4.676160	4.835710
C	-4.226540	-4.348270	4.876600
C	2.634100	-4.899520	5.119660
C	3.572310	3.587580	5.584220
C	-4.202250	5.681070	4.479780
C	-4.994970	-5.599130	4.416250
C	3.809000	-5.867600	4.902740
C	4.391340	4.873710	5.383350
C	-3.639730	3.627670	5.822090
C	-2.950010	-4.772050	5.627160
C	3.042350	-3.804580	6.123260
C	2.212470	3.933410	6.220250

Cl	-8.402960	-1.960210	-2.015750
Cl	1.889250	-5.220080	-4.472550
Cl	8.093310	1.788510	-1.724260
Cl	-1.409240	6.902330	-3.669570
Cl	-6.393880	-2.173030	0.403290
Cl	2.193020	-4.815650	-1.367630
Cl	5.825980	2.043020	0.438320
Cl	-2.095620	5.166580	-1.129930
Cl	-8.686240	0.725770	-3.597860
Cl	-0.952430	-5.223870	-5.793260
Cl	8.789960	-1.003060	-2.961390
Cl	1.568260	7.617100	-4.334650
Cl	-6.955850	3.235470	-2.787420
Cl	-3.526080	-4.817420	-4.013550
Cl	7.202550	-3.576700	-2.057930
Cl	3.900680	6.572750	-2.485840
Rh	-0.198900	-0.187490	2.934550
Rh	-0.060620	-0.138200	0.476730
H	-2.877500	3.364220	0.956010
H	-5.216660	2.137670	5.882420
H	-6.166990	5.639950	3.556630
H	-2.244870	5.204340	5.290080
H	-3.595170	-3.101210	1.073080
H	2.725900	-3.673990	1.204930
H	3.377800	2.825780	1.580250
H	-5.201260	1.636350	3.447140
H	-3.599950	1.411370	4.144070
H	-1.017340	-5.361090	2.805420
H	-1.079930	-3.898140	3.776610
H	4.756140	-1.814290	3.853440
H	3.089360	-1.631460	4.393080
H	0.575230	3.338300	4.064750
H	0.645200	4.912470	3.284550
H	-1.147440	-5.912680	5.225000
H	4.550930	-2.253850	6.296240
H	0.474130	5.146990	5.752300
H	-6.292590	4.396150	5.731380
H	-6.791070	3.368440	4.383290
H	-3.099120	-7.481170	5.096240
H	-2.193720	-7.473380	3.578620
H	5.717850	-4.471530	6.313590
H	6.308740	-3.458770	4.991630
H	2.430710	6.685870	6.061720
H	1.716490	6.886030	4.457900
H	-4.646660	-7.315330	3.132430
H	5.850340	-5.770520	4.170210
H	4.203280	6.738850	4.288160
H	-4.522280	4.937410	1.826990
H	-5.741610	3.692420	2.093670
H	-3.085090	-6.176050	1.577860
H	-4.600490	-5.281250	1.691930
H	5.498280	-3.876080	2.624310
H	4.359250	-5.173550	2.272030
H	4.328680	4.906810	2.611700
H	2.838860	5.833510	2.437130

H	-1.794040	3.286260	3.799200
H	-2.215770	4.702640	2.846200
H	-4.741590	-3.189020	3.109780
H	-3.325990	-2.591250	3.971570
H	1.387420	-3.592760	3.924840
H	1.958030	-5.011040	3.054050
H	4.302710	2.656920	3.758380
H	2.798660	1.967930	4.364930
H	-4.861640	-3.746200	5.538790
H	1.765580	-5.449130	5.505720
H	4.120210	2.897220	6.238020
H	-4.544750	6.207400	5.380840
H	-3.813070	6.440950	3.788720
H	-5.292150	-6.203860	5.283530
H	-5.916740	-5.304220	3.896530
H	3.518310	-6.659980	4.200000
H	4.081190	-6.356350	5.847930
H	4.579200	5.361240	6.349340
H	5.369880	4.632470	4.946760
H	-3.961440	4.115190	6.752290
H	-2.847710	2.914140	6.086250
H	-2.403690	-3.882670	5.968870
H	-3.213650	-5.354560	6.520290
H	2.202810	-3.115830	6.287950
H	3.288040	-4.255310	7.094300
H	1.627180	3.017060	6.374890
H	2.363790	4.392550	7.206430
C	0.051780	-0.313950	-1.568840
C	1.277260	-0.591010	-2.286060
C	-1.184390	-0.926240	-2.112620
C	2.508340	-0.120810	-1.771770
C	1.302950	-1.322080	-3.502810
O	-2.274420	-0.127960	-2.176150
O	-1.221430	-2.116340	-2.363510
C	3.695260	-0.345420	-2.452000
H	2.510500	0.448730	-0.850950
C	2.494430	-1.593680	-4.159270
H	0.380930	-1.704850	-3.918640
C	-3.485240	-0.769240	-2.561330
C	3.683530	-1.086110	-3.634760
H	4.624460	0.067170	-2.090080
H	2.501190	-2.172310	-5.074780
H	-3.400760	-1.852700	-2.485830
H	-4.264290	-0.407460	-1.895100
C	-3.863040	-0.384480	-3.994010
Br	5.312870	-1.361470	-4.566770
Cl	-2.593220	-0.904280	-5.153810
Cl	-5.408370	-1.213520	-4.374880
Cl	-4.084310	1.396100	-4.113390
C	4.741440	2.966130	-3.648790
C	3.481010	2.527130	-4.397820
H	4.722140	4.036430	-3.421790
H	4.833510	2.438630	-2.691980
H	3.569330	1.465670	-4.660710
H	3.400520	3.070740	-5.349140

C	2.203570	2.730860	-3.576950
C	0.968100	2.093590	-4.221540
H	2.361900	2.302430	-2.582110
H	2.022120	3.801460	-3.423740
H	1.213640	1.061430	-4.528020
H	0.721330	2.611290	-5.159320
C	-0.262690	2.015610	-3.372170
C	-0.308670	2.126470	-2.027990
H	-1.189200	1.794350	-3.898660
H	-1.242890	2.083400	-1.484720
H	0.571840	2.361100	-1.443890
H	5.645060	2.758790	-4.232190

DCM

6-31G(d) SCF energy in gas phase (au):	-959.695414
6-31G(d) enthalpy in gas phase (au):	-959.661255
6-31G(d) free energy in gas phase (au):	-959.689575
6-311+G(d,p) SCF energy in implicit solvent (DCM) (au):	-959.783073
6-311+G(d,p) enthalpy in implicit solvent (DCM) (au):	-959.748914
6-311+G(d,p) free energy in implicit solvent (DCM) (au):	-959.777234

Cartesian coordinates

ATOM	X	Y	Z
C	-0.000000	0.768690	-0.000000
H	0.000000	1.380380	-0.899290
H	-0.000010	1.380380	0.899290
Cl	1.493960	-0.216850	0.000000
Cl	-1.493960	-0.216850	-0.000000

HFIP

6-31G(d) SCF energy in gas phase (au):	-789.793708
6-31G(d) enthalpy in gas phase (au):	-789.720911
6-31G(d) free energy in gas phase (au):	-789.762233
6-311+G(d,p) SCF energy in implicit solvent (HFIP) (au):	-790.081232
6-311+G(d,p) enthalpy in implicit solvent (HFIP) (au):	-790.008435
6-311+G(d,p) free energy in implicit solvent (HFIP) (au):	-790.044584

Cartesian coordinates

ATOM	X	Y	Z
H	-0.000000	0.471050	-1.642200
C	0.000000	0.528120	-0.551090
O	0.000000	1.883840	-0.197980
H	0.000020	1.935150	0.773410
F	-2.354140	0.437240	-0.595100
F	-1.379450	-0.024660	1.295760
F	1.317510	-1.458570	-0.349700
F	2.354140	0.437240	-0.595100
F	-1.317510	-1.458570	-0.349700
F	1.379450	-0.024660	1.295760
C	-1.280350	-0.151480	-0.046500
C	1.280350	-0.151480	-0.046510

Rh-1a•(DCM) carbonyl

6-31G(d)–SDD(Rh) SCF energy in gas phase (au):	-9873.755113

6-31G(d)–SDD(Rh) enthalpy in gas phase (au): -9872.228954
6-31G(d)–SDD(Rh) free energy in gas phase (au): -9872.450799
6-311+G(d,p)–SDD(Rh) SCF energy in implicit solvent (DCM) (au): -9878.052803
6-311+G(d,p)–SDD(Rh) enthalpy in implicit solvent (DCM) (au): -9876.526644
6-311+G(d,p)–SDD(Rh) free energy in implicit solvent (DCM) (au): -9876.748489

Cartesian coordinates

ATOM	X	Y	Z
Cl	0.430880	-3.662870	-3.636240
Cl	2.303140	-5.909380	-3.141650
C	1.814030	-4.246000	-2.662610
H	1.511080	-4.262560	-1.620380
H	2.655480	-3.579830	-2.824660
Rh	-0.107110	0.256770	1.182930
O	1.016510	-1.337040	1.747770
O	0.948520	-0.814340	3.953540
O	2.620420	-4.728650	1.353780
O	3.718320	-0.482300	2.624380
N	3.279870	-2.661500	2.100510
C	1.339800	-1.498550	2.971410
C	2.244620	-2.717370	3.161810
H	1.617600	-3.560440	2.866370
C	3.336100	-3.742630	1.208070
C	4.307240	-3.646630	0.094190
C	4.480920	-4.725070	-0.753100
H	3.917970	-5.631810	-0.566640
C	5.359370	-4.634220	-1.853710
H	5.467310	-5.484850	-2.518140
C	6.060630	-3.470410	-2.093700
H	6.729880	-3.396690	-2.946670
C	5.925490	-2.351500	-1.231150
C	6.629720	-1.134260	-1.426210
H	7.300310	-1.049760	-2.276490
C	6.457440	-0.072070	-0.562680
H	6.974430	0.866450	-0.727970
C	5.579490	-0.181580	0.534950
H	5.440030	0.655940	1.204490
C	4.873530	-1.351250	0.751580
C	5.034170	-2.452060	-0.121690
C	3.934220	-1.436650	1.888660
C	2.770090	-3.046840	4.586360
C	3.656300	-4.303280	4.483500
H	3.122260	-5.136390	4.014940
H	3.969900	-4.615040	5.485870
H	4.561320	-4.108340	3.897610
C	3.577570	-1.915540	5.243060
H	3.849980	-2.217250	6.262010
H	3.000000	-0.991350	5.293490
H	4.501330	-1.708250	4.696440
C	1.544250	-3.385390	5.460690
H	1.880060	-3.739550	6.442050
H	0.937890	-4.177010	5.004430
H	0.905900	-2.512210	5.606700
C	-0.012280	-0.236340	-0.745730
C	-1.083570	-0.376200	-1.679570

C	-0.834380	-0.621750	-3.058810
C	-2.434840	-0.330940	-1.243590
C	-1.871960	-0.850520	-3.945440
H	0.182770	-0.637190	-3.428620
C	-3.474400	-0.601680	-2.118170
H	-2.647160	-0.137300	-0.203260
C	-3.183070	-0.862640	-3.456970
H	-1.675170	-1.043610	-4.993380
H	-4.493530	-0.631610	-1.761510
Br	-4.608700	-1.264390	-4.648320
C	1.354480	-0.552810	-1.215290
O	1.875090	-1.643600	-1.097180
O	1.963500	0.538830	-1.747340
C	3.319450	0.409770	-2.156920
H	3.871560	1.252980	-1.746560
H	3.746240	-0.526940	-1.800740
C	3.406710	0.453230	-3.687610
Cl	5.145900	0.593940	-4.131250
Cl	2.504490	1.855070	-4.335680
Cl	2.732630	-1.058720	-4.398900
Rh	-0.214570	0.846140	3.570460
O	1.600450	1.391210	1.069980
O	1.457720	1.992300	3.251500
O	5.060690	2.828910	-0.100250
O	0.860320	4.210590	1.055600
N	2.998810	3.609120	0.528780
C	1.956740	2.059590	2.097890
C	3.165830	2.964790	1.852810
H	3.993560	2.270690	1.700770
C	4.044060	3.459050	-0.388130
C	3.875660	4.085620	-1.716940
C	4.927630	4.069790	-2.617690
H	5.862770	3.611020	-2.317230
C	4.774620	4.636380	-3.898190
H	5.608440	4.621510	-4.592690
C	3.567400	5.188940	-4.276800
H	3.444540	5.609370	-5.271460
C	2.463090	5.205100	-3.387150
C	1.188200	5.719080	-3.743220
H	1.048080	6.124390	-4.742050
C	0.142870	5.694890	-2.843200
H	-0.837260	6.069040	-3.119900
C	0.329860	5.185360	-1.541470
H	-0.497180	5.177070	-0.844770
C	1.560040	4.680370	-1.160490
C	2.635770	4.661530	-2.079390
C	1.745150	4.166690	0.211850
C	3.608600	3.926630	2.996670
C	4.800970	4.761690	2.490130
H	4.507830	5.429010	1.672150
H	5.618490	4.126660	2.134850
H	5.179970	5.386590	3.306150
C	2.504140	4.882360	3.478720
H	2.887380	5.472880	4.320010
H	1.618860	4.337470	3.809040

H	2.199730	5.577350	2.691910
C	4.099150	3.058180	4.174460
H	3.284320	2.480840	4.612940
H	4.528290	3.702980	4.949940
H	4.877510	2.356360	3.851080
O	-1.280460	1.897770	0.822890
O	-1.370280	2.458730	3.019420
O	-2.757290	4.833330	-1.142070
O	-4.104290	1.593340	1.783440
N	-3.524350	3.303360	0.384640
C	-1.675280	2.609890	1.807600
C	-2.537800	3.797120	1.374830
H	-1.856130	4.407960	0.782530
C	-3.488290	3.879640	-0.895300
C	-4.369290	3.288090	-1.927100
C	-4.341310	3.789840	-3.216820
H	-3.670220	4.611920	-3.439040
C	-5.168030	3.235240	-4.214650
H	-5.128270	3.632730	-5.223970
C	-6.024530	2.194940	-3.914650
H	-6.659130	1.765090	-4.684380
C	-6.084750	1.660870	-2.602790
C	-6.932910	0.579240	-2.246410
H	-7.576270	0.147420	-3.007790
C	-6.926060	0.068860	-0.964620
H	-7.558380	-0.772680	-0.703690
C	-6.065280	0.608500	0.014970
H	-6.027430	0.179430	1.007270
C	-5.228090	1.663410	-0.299190
C	-5.231870	2.214590	-1.602930
C	-4.266920	2.157740	0.708420
C	-3.116460	4.739780	2.468940
C	-3.946520	5.829650	1.763020
H	-4.830590	5.407070	1.272860
H	-3.358730	6.357310	1.005120
H	-4.294230	6.560930	2.500920
C	-3.999580	4.037600	3.513610
H	-4.909820	3.628920	3.066560
H	-4.299670	4.768500	4.274600
H	-3.466290	3.221410	4.002820
C	-1.922290	5.420390	3.171420
H	-1.259700	5.906490	2.445770
H	-1.330150	4.700740	3.738700
H	-2.292680	6.188080	3.860510
O	-1.798650	-0.815270	1.538690
O	-1.902880	-0.331310	3.746990
O	-5.144440	-2.216830	0.558190
O	-1.347940	-3.406880	2.806180
N	-3.302710	-2.891130	1.743990
C	-2.300100	-0.919890	2.710520
C	-3.548310	-1.804480	2.723260
H	-4.306050	-1.189050	2.232940
C	-4.130280	-2.909780	0.612160
C	-3.719180	-3.771760	-0.515450
C	-4.493250	-3.808030	-1.662990

H	-5.403970	-3.220820	-1.695620
C	-4.088560	-4.578220	-2.770980
H	-4.695770	-4.578800	-3.670190
C	-2.921840	-5.315050	-2.719470
H	-2.604300	-5.904370	-3.575230
C	-2.106270	-5.304310	-1.559970
C	-0.887410	-6.025220	-1.468500
H	-0.567660	-6.625610	-2.314670
C	-0.098600	-5.938040	-0.339260
H	0.846530	-6.467920	-0.286180
C	-0.495200	-5.134370	0.749470
H	0.142950	-5.043100	1.618640
C	-1.689590	-4.436880	0.701380
C	-2.512440	-4.508340	-0.448480
C	-2.069660	-3.568920	1.831140
C	-4.166330	-2.223090	4.086760
C	-5.336450	-3.180890	3.793150
H	-4.981610	-4.123870	3.363650
H	-6.052060	-2.738050	3.092160
H	-5.864690	-3.414340	4.724200
C	-3.187600	-2.901500	5.058870
H	-2.314630	-2.273880	5.247660
H	-2.835280	-3.861050	4.675260
H	-3.701260	-3.080980	6.011530
C	-4.732470	-0.944680	4.741050
H	-5.440480	-0.437260	4.074330
H	-3.936920	-0.239660	4.989810
H	-5.265400	-1.207720	5.661830

Rh-1a•(DCM) ester

6-31G(d)-SDD(Rh) SCF energy in gas phase (au):	-9873.756656
6-31G(d)-SDD(Rh) enthalpy in gas phase (au):	-9872.230888
6-31G(d)-SDD(Rh) free energy in gas phase (au):	-9872.452907
6-311+G(d,p)-SDD(Rh) SCF energy in implicit solvent (DCM) (au):	-9878.055406
6-311+G(d,p)-SDD(Rh) enthalpy in implicit solvent (DCM) (au):	-9876.529638
6-311+G(d,p)-SDD(Rh) free energy in implicit solvent (DCM) (au):	-9876.751657

Cartesian coordinates

ATOM	X	Y	Z
Cl	-0.079130	4.750440	1.423320
Cl	1.843910	3.102490	2.958850
C	0.656650	3.136710	1.616120
H	1.167900	2.878100	0.692540
H	-0.135200	2.428910	1.842740
Rh	-0.074400	-0.609880	-1.009530
O	-0.951880	-2.420410	-0.681170
O	-1.220120	-2.912590	-2.875510
O	-1.898930	-5.485010	1.324350
O	-3.859870	-2.283870	-1.269210
N	-2.985730	-3.985880	-0.026290
C	-1.388920	-3.129760	-1.646130
C	-2.115260	-4.385700	-1.159330
H	-1.331960	-4.981260	-0.685090
C	-2.737080	-4.596120	1.213000
C	-3.532420	-4.129810	2.370030

C	-3.357330	-4.731540	3.602880
H	-2.654470	-5.553640	3.683540
C	-4.080670	-4.278390	4.725540
H	-3.930410	-4.758080	5.687790
C	-4.973450	-3.232600	4.606510
H	-5.527330	-2.880010	5.472460
C	-5.184020	-2.598020	3.355210
C	-6.082710	-1.512840	3.179270
H	-6.638070	-1.149650	4.039300
C	-6.236000	-0.915770	1.945100
H	-6.901340	-0.068600	1.820280
C	-5.500610	-1.376980	0.834120
H	-5.609730	-0.895220	-0.127930
C	-4.614540	-2.430310	0.970040
C	-4.446530	-3.061020	2.225330
C	-3.814580	-2.867440	-0.193070
C	-2.784660	-5.316210	-2.211280
C	-3.468580	-6.470030	-1.452550
H	-2.767440	-6.989060	-0.790940
H	-3.867310	-7.195750	-2.169960
H	-4.305560	-6.109320	-0.844680
C	-3.821690	-4.622680	-3.110290
H	-4.181700	-5.341360	-3.856890
H	-3.390640	-3.765340	-3.629480
H	-4.684330	-4.272380	-2.538100
C	-1.658460	-5.909770	-3.083650
H	-2.079140	-6.654640	-3.768820
H	-0.901510	-6.409090	-2.466840
H	-1.162450	-5.137530	-3.674380
C	0.129960	-0.408440	0.962010
C	1.328310	-0.274240	1.723790
C	1.335260	-0.451260	3.135640
C	2.543350	0.092430	1.086460
C	2.481210	-0.228720	3.877630
H	0.429280	-0.767790	3.638450
C	3.691280	0.312000	1.826370
H	2.558210	0.211280	0.013470
C	3.641400	0.181260	3.214320
H	2.479250	-0.343380	4.954720
H	4.613110	0.594400	1.343590
Br	5.180170	0.639530	4.227330
C	-1.128090	-0.606990	1.714700
O	-1.547300	-1.675920	2.103330
O	-1.770780	0.582910	1.872100
C	-3.046250	0.569430	2.506870
H	-3.771180	1.017680	1.827140
H	-3.339710	-0.450660	2.751910
C	-2.973760	1.395320	3.793110
Cl	-4.636430	1.480990	4.479290
Cl	-2.379720	3.049010	3.459050
Cl	-1.877300	0.595080	4.979270
Rh	-0.329320	-1.130390	-3.400310
O	-1.931540	0.253220	-1.076750
O	-2.157580	-0.207760	-3.288330
O	-5.222310	1.712950	0.146760

O	-1.867830	2.773610	-2.751020
N	-3.681120	2.320390	-1.438540
C	-2.534420	0.305100	-2.202750
C	-3.881290	1.022160	-2.123180
H	-4.447730	0.432100	-1.403700
C	-4.444470	2.561110	-0.290440
C	-4.282350	3.875280	0.363270
C	-5.068290	4.192510	1.457970
H	-5.781880	3.459330	1.816610
C	-4.929840	5.442130	2.093120
H	-5.547250	5.675210	2.954720
C	-4.012480	6.363500	1.628350
H	-3.904460	7.326580	2.120170
C	-3.189450	6.071220	0.511170
C	-2.220270	6.976990	0.005440
H	-2.108510	7.945500	0.485620
C	-1.420610	6.631460	-1.064510
H	-0.665580	7.319730	-1.429160
C	-1.554530	5.369510	-1.677290
H	-0.911700	5.088150	-2.502070
C	-2.499250	4.468600	-1.219020
C	-3.330190	4.802200	-0.123310
C	-2.626600	3.148480	-1.866940
C	-4.755060	1.109210	-3.408070
C	-6.061540	1.842410	-3.044490
H	-5.875870	2.885650	-2.765930
H	-6.580200	1.356350	-2.211480
H	-6.732220	1.846060	-3.910580
C	-4.082030	1.845590	-4.578270
H	-4.743420	1.805390	-5.452420
H	-3.126310	1.389620	-4.839330
H	-3.904270	2.899460	-4.345410
C	-5.113010	-0.330270	-3.833340
H	-4.231370	-0.880770	-4.163920
H	-5.835040	-0.297230	-4.657400
H	-5.564440	-0.889240	-3.006160
O	0.766960	1.177000	-1.565910
O	0.557960	0.685220	-3.771450
O	1.830620	4.881330	-1.409030
O	3.492190	0.835540	-2.730350
N	2.728160	2.911680	-2.164240
C	0.932200	1.410120	-2.815370
C	1.585990	2.763380	-3.095200
H	0.857840	3.488360	-2.727460
C	2.696220	4.020870	-1.298880
C	3.737650	4.082260	-0.249670
C	3.723150	5.123300	0.661920
H	2.940430	5.869320	0.584890
C	4.698570	5.191970	1.677490
H	4.665140	6.006170	2.394170
C	5.683000	4.229730	1.768780
H	6.432460	4.281730	2.553780
C	5.731030	3.149930	0.849700
C	6.715430	2.128410	0.906410
H	7.473790	2.176700	1.682780

C	6.697180	1.076970	0.011230
H	7.434150	0.283840	0.075950
C	5.690470	0.995140	-0.973670
H	5.647670	0.144860	-1.642190
C	4.722100	1.980820	-1.061850
C	4.730710	3.075970	-0.164680
C	3.629980	1.843740	-2.048250
C	1.882570	3.156510	-4.571820
C	2.550670	4.545480	-4.567480
H	3.533560	4.517650	-4.084460
H	1.938650	5.287400	-4.044900
H	2.697470	4.883420	-5.599190
C	2.793950	2.169540	-5.320130
H	3.796160	2.134120	-4.884800
H	2.892690	2.497380	-6.362300
H	2.383510	1.158980	-5.308370
C	0.531030	3.273770	-5.307390
H	-0.149950	3.954490	-4.783600
H	0.035310	2.305720	-5.390460
H	0.698090	3.670010	-6.315600
O	1.767340	-1.460930	-1.182540
O	1.519790	-2.055540	-3.350770
O	5.528570	-2.134100	-0.404700
O	1.525570	-4.291720	-0.928010
N	3.543880	-3.244150	-0.695370
C	2.130400	-2.058850	-2.251900
C	3.480170	-2.769910	-2.099690
H	4.217320	-1.963150	-2.129700
C	4.592380	-2.755850	0.092540
C	4.511240	-3.004020	1.550980
C	5.579110	-2.669970	2.364590
H	6.483170	-2.285300	1.906060
C	5.474470	-2.804690	3.763480
H	6.321250	-2.544600	4.389620
C	4.291250	-3.221590	4.340220
H	4.202400	-3.288620	5.421450
C	3.171550	-3.557590	3.538740
C	1.915340	-3.944010	4.077600
H	1.799830	-3.980440	5.158060
C	0.858370	-4.260880	3.249300
H	-0.106550	-4.536550	3.661290
C	1.017720	-4.245570	1.847700
H	0.194660	-4.524750	1.204250
C	2.226300	-3.866700	1.293060
C	3.307060	-3.482540	2.120880
C	2.379270	-3.850110	-0.173010
C	3.913870	-3.785780	-3.196610
C	5.224300	-4.449710	-2.733570
H	5.064020	-5.068380	-1.844070
H	5.991060	-3.705640	-2.495670
H	5.606830	-5.099700	-3.528360
C	2.877150	-4.879220	-3.502330
H	1.915200	-4.450040	-3.789000
H	2.711720	-5.530060	-2.641620
H	3.245550	-5.493960	-4.332930

C	4.202980	-2.979360	-4.480390
H	4.953460	-2.200220	-4.297930
H	3.298950	-2.501140	-4.862330
H	4.595680	-3.648530	-5.254340

Rh-1a•(DCM) conformer A

6-31G(d)-SDD(Rh) SCF energy in gas phase (au):	-9873.758639
6-31G(d)-SDD(Rh) enthalpy in gas phase (au):	-9872.232838
6-31G(d)-SDD(Rh) free energy in gas phase (au):	-9872.453981
6-311+G(d,p)-SDD(Rh) SCF energy in implicit solvent (DCM) (au):	-9878.055037
6-311+G(d,p)-SDD(Rh) enthalpy in implicit solvent (DCM) (au):	-9876.529236
6-311+G(d,p)-SDD(Rh) free energy in implicit solvent (DCM) (au):	-9876.750379

Cartesian coordinates

ATOM	X	Y	Z
Rh	-0.239360	-0.833500	-0.849680
Rh	-0.520060	-1.961850	-3.021820
O	1.186640	0.362900	-1.739240
O	0.924780	-0.689880	-3.732640
O	3.633990	3.362420	-2.525760
O	3.622240	-1.205770	-2.373950
O	1.230690	-2.212490	-0.509380
O	0.894330	-3.351780	-2.432730
O	4.530990	-3.843520	0.889640
O	0.017080	-4.544940	0.674650
O	-1.657840	-2.123010	-0.179330
O	-1.946700	-3.175120	-2.165380
O	-3.587400	-3.918810	2.473540
O	-4.349530	-1.462780	-1.292130
O	-1.719180	0.449380	-1.417540
O	-1.941470	-0.535510	-3.449570
O	-4.436760	3.045810	-1.071990
O	-0.667330	2.296700	-3.534140
N	3.699570	1.070400	-2.548560
N	2.269390	-4.167330	0.747730
N	-4.124250	-2.796200	0.548100
N	-2.617240	2.746620	-2.434980
C	1.479890	0.135950	-2.963690
C	2.577160	1.050410	-3.517330
H	2.150650	2.050950	-3.445770
C	4.143680	2.324060	-2.109130
C	5.232190	2.342460	-1.107000
C	5.752980	3.553500	-0.685300
H	5.372910	4.466570	-1.129280
C	6.743490	3.593750	0.316290
H	7.132220	4.552540	0.643330
C	7.198890	2.427520	0.895450
H	7.947590	2.459960	1.682060
C	6.699060	1.166830	0.479740
C	7.129570	-0.061560	1.046880
H	7.870070	-0.038420	1.841630
C	6.610320	-1.263210	0.607890
H	6.925720	-2.201180	1.051190
C	5.641090	-1.290710	-0.416450
H	5.224190	-2.234370	-0.741640

C	5.190410	-0.110040	-0.980750
C	5.707780	1.132840	-0.545500
C	4.132240	-0.154900	-2.010390
C	3.010380	0.860710	-5.001910
C	4.114050	1.889020	-5.319750
H	3.787180	2.912850	-5.114330
H	4.380550	1.818260	-6.379900
H	5.023560	1.699620	-4.738750
C	3.542450	-0.545790	-5.324100
H	2.810550	-1.316480	-5.080510
H	4.463050	-0.764350	-4.775190
H	3.771720	-0.602580	-6.395210
C	1.790780	1.178840	-5.892690
H	1.371500	2.163300	-5.654700
H	0.999270	0.439260	-5.767890
H	2.100510	1.186900	-6.944070
C	1.399510	-3.210140	-1.290390
C	2.366140	-4.263890	-0.730070
H	3.361300	-3.865310	-0.942770
C	3.426920	-3.780980	1.428780
C	3.262170	-3.245350	2.799060
C	4.380210	-2.871390	3.525410
H	5.361010	-3.055430	3.102230
C	4.236370	-2.238530	4.774730
H	5.119660	-1.943670	5.331130
C	2.979590	-1.954610	5.273410
H	2.872010	-1.433860	6.221240
C	1.813530	-2.322140	4.558500
C	0.499040	-2.023540	5.008610
H	0.378720	-1.483520	5.944410
C	-0.603330	-2.402720	4.272530
H	-1.604610	-2.151380	4.605240
C	-0.448180	-3.114030	3.063360
H	-1.316090	-3.417050	2.493110
C	0.818450	-3.408250	2.592320
C	1.966260	-3.002080	3.314070
C	0.967800	-4.097680	1.297490
C	2.351310	-5.692230	-1.355390
C	3.243820	-6.594080	-0.483120
H	3.346360	-7.577150	-0.956070
H	2.805720	-6.743470	0.509400
H	4.242970	-6.166180	-0.352620
C	0.959130	-6.332410	-1.484470
H	0.275350	-5.694530	-2.047960
H	0.511860	-6.525390	-0.508640
H	1.058010	-7.286830	-2.016210
C	2.988680	-5.578680	-2.756790
H	2.391350	-4.943310	-3.414230
H	3.063190	-6.573580	-3.209950
H	4.001430	-5.160550	-2.698400
C	-2.231070	-2.953720	-0.958100
C	-3.308540	-3.758750	-0.231430
H	-2.756320	-4.310350	0.532630
C	-4.149370	-2.967880	1.941930
C	-4.896850	-1.964730	2.732420

C	-4.991840	-2.111560	4.104150
H	-4.521000	-2.972090	4.566840
C	-5.685170	-1.155960	4.875710
H	-5.746050	-1.280470	5.952420
C	-6.284040	-0.069220	4.271280
H	-6.814410	0.669190	4.866270
C	-6.208780	0.110710	2.866320
C	-6.784070	1.222040	2.195620
H	-7.328690	1.958460	2.779270
C	-6.632700	1.383360	0.834350
H	-7.044520	2.252900	0.333970
C	-5.913880	0.433500	0.080340
H	-5.781290	0.564930	-0.985630
C	-5.357540	-0.672140	0.697570
C	-5.495410	-0.853660	2.094640
C	-4.583980	-1.643030	-0.103400
C	-4.122020	-4.814910	-1.029740
C	-5.128630	-5.463940	-0.060080
H	-5.655160	-6.279680	-0.567810
H	-5.880560	-4.742510	0.278170
H	-4.629580	-5.873150	0.824380
C	-4.883190	-4.250280	-2.240260
H	-4.210990	-3.738160	-2.930310
H	-5.659590	-3.543110	-1.936220
H	-5.371610	-5.076680	-2.771370
C	-3.130660	-5.899690	-1.500360
H	-2.560470	-6.308150	-0.657540
H	-2.422310	-5.502740	-2.229800
H	-3.682380	-6.724390	-1.966010
C	-2.189910	0.360550	-2.602500
C	-3.198590	1.467630	-2.911320
H	-4.014150	1.282940	-2.211300
C	-3.339680	3.445050	-1.460430
C	-2.726970	4.673990	-0.912580
C	-3.432710	5.438060	0.001420
H	-4.431390	5.123380	0.283380
C	-2.852910	6.594840	0.559040
H	-3.415620	7.180060	1.279200
C	-1.572820	6.973010	0.206950
H	-1.120750	7.857550	0.647680
C	-0.820230	6.213450	-0.724420
C	0.509570	6.542410	-1.094480
H	0.974310	7.419110	-0.651300
C	1.207570	5.757620	-1.989690
H	2.230170	6.001040	-2.259110
C	0.606000	4.615990	-2.557700
H	1.161560	3.997410	-3.250000
C	-0.688400	4.266040	-2.218050
C	-1.418640	5.052170	-1.296520
C	-1.287200	3.042770	-2.787800
C	-3.844420	1.520450	-4.324390
C	-4.805960	2.724370	-4.358880
H	-4.265540	3.673210	-4.270060
H	-5.541830	2.676920	-3.549220
H	-5.344720	2.737010	-5.312670

C	-2.837990	1.650810	-5.479420
H	-3.382150	1.614950	-6.431290
H	-2.106240	0.841980	-5.463150
H	-2.295570	2.599000	-5.438880
C	-4.673840	0.231330	-4.502530
H	-5.239680	0.287060	-5.439540
H	-5.389330	0.101340	-3.681950
H	-4.035760	-0.653110	-4.532240
C	-0.117830	-0.020620	0.972710
C	1.043740	0.196030	1.772410
C	0.959020	0.822060	3.046900
C	2.331780	-0.148330	1.281530
C	2.096340	1.132380	3.770730
H	-0.009110	1.094850	3.448650
C	3.471120	0.132010	2.018600
H	2.413610	-0.645570	0.325710
C	3.345450	0.789750	3.243820
H	2.027470	1.642960	4.723650
H	4.447560	-0.149020	1.654500
Br	4.918400	1.248930	4.199740
C	-1.437140	0.286980	1.579490
O	-2.126280	-0.541720	2.134260
O	-1.796760	1.587580	1.402490
C	-3.091870	1.978820	1.849300
H	-3.531660	2.597440	1.068530
H	-3.716950	1.106870	2.033380
C	-2.993880	2.796180	3.145220
Cl	-4.623610	3.491030	3.475250
Cl	-1.813010	4.128060	2.982710
Cl	-2.515010	1.741080	4.526860
C	2.484270	3.398940	0.553200
H	2.754490	2.350680	0.460310
H	2.696020	3.949600	-0.358360
Cl	3.474680	4.097430	1.882540
Cl	0.742040	3.492030	0.883670

Rh-1a•(HFIP)

6-31G(d)–SDD(Rh) SCF energy in gas phase (au): -9703.869852
6-31G(d)–SDD(Rh) enthalpy in gas phase (au): -9702.305783
6-31G(d)–SDD(Rh) free energy in gas phase (au): -9702.535825
6-311+G(d,p)–SDD(Rh) SCF energy in implicit solvent (HFIP) (au): -9708.369523
6-311+G(d,p)–SDD(Rh) enthalpy in implicit solvent (HFIP) (au): -9706.805454
6-311+G(d,p)–SDD(Rh) free energy in implicit solvent (HFIP) (au): -9707.030322

Cartesian coordinates

ATOM	X	Y	Z
Rh	0.473420	-1.060760	0.808220
Rh	0.999160	-2.445270	2.769460
O	-1.107200	-0.292400	1.879280
O	-0.630060	-1.599750	3.668160
O	-4.077670	2.037830	3.252930
O	-3.221870	-2.313110	2.135430
O	-0.744720	-2.594750	0.238670
O	-0.171610	-3.946450	1.959110
O	-3.694470	-4.492280	-1.506760

O	0.869430	-4.511470	-1.192660
O	2.083160	-1.977740	-0.042780
O	2.610260	-3.233020	1.765890
O	4.330800	-2.966630	-2.905250
O	4.604300	-0.959480	1.179250
O	1.712210	0.365920	1.567710
O	2.158350	-0.869310	3.417480
O	3.860490	3.429230	1.610160
O	0.411690	1.609990	3.979320
N	-3.693560	-0.168060	2.759320
N	-1.412100	-4.500330	-1.319860
N	4.636780	-2.041330	-0.830490
N	2.191740	2.594710	2.944480
C	-1.341370	-0.771350	3.041650
C	-2.576710	-0.185310	3.731370
H	-2.331980	0.868350	3.869260
C	-4.368080	1.053570	2.584230
C	-5.437120	1.089040	1.561060
C	-6.156170	2.254850	1.368610
H	-5.938620	3.108090	1.999900
C	-7.128510	2.331960	0.351190
H	-7.676910	3.257330	0.205860
C	-7.376100	1.246590	-0.463970
H	-8.118120	1.310240	-1.255280
C	-6.672420	0.028360	-0.283200
C	-6.897790	-1.121840	-1.084920
H	-7.635280	-1.067170	-1.881090
C	-6.189730	-2.286690	-0.864920
H	-6.352100	-3.161660	-1.485010
C	-5.221000	-2.350770	0.158720
H	-4.651090	-3.258120	0.308190
C	-4.967450	-1.242670	0.948130
C	-5.689360	-0.041750	0.748640
C	-3.907970	-1.310310	1.976740
C	-2.951590	-0.740840	5.140020
C	-4.228500	-0.025300	5.623700
H	-4.465720	-0.359110	6.639810
H	-5.089890	-0.262640	4.989690
H	-4.106160	1.061400	5.636210
C	-3.204180	-2.258010	5.165950
H	-2.341300	-2.815880	4.801090
H	-4.069490	-2.532160	4.555750
H	-3.412220	-2.566160	6.197930
C	-1.808200	-0.379240	6.111650
H	-0.889680	-0.914710	5.869600
H	-2.104620	-0.637490	7.134830
H	-1.589840	0.694770	6.083470
C	-0.726000	-3.714530	0.854660
C	-1.521420	-4.808680	0.128810
H	-2.566260	-4.583900	0.357030
C	-2.601600	-4.215650	-1.996940
C	-2.490370	-3.531660	-3.306690
C	-3.632470	-3.283360	-4.048150
H	-4.581420	-3.661090	-3.684750
C	-3.557490	-2.531570	-5.236990

H	-4.460090	-2.342830	-5.808380
C	-2.352150	-2.002100	-5.655370
H	-2.304320	-1.393920	-6.554900
C	-1.164030	-2.227960	-4.917410
C	0.092600	-1.668680	-5.274100
H	0.150450	-1.035960	-6.156330
C	1.217480	-1.915110	-4.514330
H	2.172480	-1.471840	-4.776540
C	1.147280	-2.754940	-3.382550
H	2.035740	-2.962470	-2.802470
C	-0.063030	-3.307440	-3.005410
C	-1.239150	-3.032700	-3.741980
C	-0.128370	-4.156100	-1.800960
C	-1.308860	-6.291390	0.555920
C	-2.084420	-7.180370	-0.433890
H	-3.135360	-6.882570	-0.504830
H	-2.041140	-8.223630	-0.101870
H	-1.648650	-7.130020	-1.437480
C	0.158450	-6.749160	0.601390
H	0.612930	-6.746700	-0.390610
H	0.196590	-7.771830	0.996470
H	0.759240	-6.106910	1.248210
C	-1.937230	-6.454050	1.956500
H	-2.994940	-6.162780	1.952300
H	-1.416650	-5.845900	2.699160
H	-1.880180	-7.503670	2.265970
C	2.824810	-2.783040	0.608720
C	4.030340	-3.244730	-0.210930
H	3.589850	-3.779450	-1.055100
C	4.704990	-2.012040	-2.232140
C	5.265190	-0.793210	-2.856800
C	5.419670	-0.740690	-4.230450
H	5.132870	-1.606320	-4.817720
C	5.943510	0.416150	-4.843810
H	6.057770	0.444220	-5.923040
C	6.310080	1.506930	-4.081640
H	6.707640	2.400680	-4.554630
C	6.164460	1.487130	-2.671130
C	6.503890	2.588660	-1.842030
H	6.907810	3.484510	-2.304560
C	6.302590	2.536620	-0.478700
H	6.531250	3.393280	0.145640
C	5.771310	1.375140	0.118850
H	5.606040	1.336280	1.187220
C	5.442640	0.277920	-0.656550
C	5.630440	0.315200	-2.058960
C	4.867830	-0.923300	-0.016300
C	5.044580	-4.224830	0.443260
C	6.149040	-4.519870	-0.590280
H	5.731560	-4.890420	-1.532110
H	6.830600	-5.279770	-0.192370
H	6.741530	-3.625020	-0.810370
C	5.693650	-3.698410	1.733750
H	6.309830	-2.815100	1.545470
H	6.343760	-4.478110	2.149490

H	4.942400	-3.433800	2.479220
C	4.292110	-5.539520	0.737870
H	3.804680	-5.927490	-0.164580
H	3.527690	-5.399090	1.504160
H	5.001390	-6.297150	1.090240
C	2.222040	0.179930	2.727300
C	3.018380	1.392540	3.208410
H	3.828450	1.477630	2.483740
C	2.723840	3.547660	2.065560
C	1.855880	4.686320	1.697730
C	2.344190	5.671510	0.853010
H	3.368200	5.597520	0.502570
C	1.518340	6.741180	0.456630
H	1.909230	7.496920	-0.217210
C	0.218880	6.826370	0.916660
H	-0.422910	7.643700	0.600040
C	-0.310700	5.842450	1.787010
C	-1.651360	5.864710	2.248940
H	-2.294890	6.684970	1.945040
C	-2.143780	4.845970	3.036560
H	-3.179910	4.843320	3.355820
C	-1.316100	3.768120	3.406230
H	-1.712930	2.962030	4.008750
C	0.002240	3.723110	2.988150
C	0.524920	4.754040	2.173000
C	0.843670	2.569710	3.353630
C	3.694090	1.341730	4.606810
C	4.418660	2.684250	4.825100
H	3.709390	3.516180	4.894420
H	5.117420	2.902970	4.010560
H	4.983840	2.649270	5.762920
C	2.725650	1.099310	5.776030
H	2.017310	1.923410	5.892860
H	3.303450	1.017380	6.704980
H	2.154220	0.180650	5.636300
C	4.750950	0.217870	4.569240
H	4.286770	-0.763230	4.457150
H	5.328420	0.227630	5.500710
H	5.449780	0.354900	3.735700
C	0.199070	-0.051650	-0.891040
C	-0.950710	0.009710	-1.732690
C	1.426460	0.611090	-1.383170
C	-2.183700	-0.547190	-1.301660
C	-0.920780	0.691390	-2.983330
O	1.388280	1.973090	-1.159810
O	2.368530	0.053910	-1.891940
C	-3.333000	-0.395230	-2.058080
H	-2.216910	-1.081850	-0.363510
C	-2.073560	0.863890	-3.730040
H	0.013150	1.095880	-3.357150
C	2.588250	2.706510	-1.430550
C	-3.275530	0.332220	-3.247130
H	-4.271370	-0.811150	-1.724560
H	-2.053170	1.401690	-4.670670
H	3.450620	2.043040	-1.407200

H	2.673730	3.460820	-0.652260
C	2.525500	3.414180	-2.793070
Br	-4.879650	0.637030	-4.210930
Cl	2.629450	2.244160	-4.153810
Cl	3.930530	4.539390	-2.865570
Cl	1.013910	4.355650	-2.940320
H	-0.183710	3.046500	-0.572810
O	-0.827080	3.746680	-0.360100
C	-1.993140	3.559470	-1.100860
H	-1.848550	2.918050	-1.975620
C	-2.465550	4.920680	-1.626140
C	-3.076610	2.891360	-0.238090
F	-1.649520	5.360710	-2.606750
F	-2.487730	5.859790	-0.668240
F	-3.704590	4.824840	-2.149060
F	-4.077020	2.413040	-1.010410
F	-3.615590	3.739960	0.653640
F	-2.544040	1.857690	0.437000

Rh-1a•(DCM)$_2$ conformer B

6-31G(d)-SDD(Rh) SCF energy in gas phase (au): -10833.469370
6-31G(d)-SDD(Rh) enthalpy in gas phase (au): -10831.906930
6-31G(d)-SDD(Rh) free energy in gas phase (au): -10832.140150
6-311+G(d,p)-SDD(Rh) SCF energy in implicit solvent (DCM) (au): -10837.853000
6-311+G(d,p)-SDD(Rh) enthalpy in implicit solvent (DCM) (au): -10836.290560
6-311+G(d,p)-SDD(Rh) free energy in implicit solvent (DCM) (au): -10836.523780

Cartesian coordinates

ATOM	X	Y	Z
Cl	-3.598510	-2.635590	-2.368910
Cl	-1.387000	-3.739190	-4.030300
C	-2.085660	-3.587660	-2.377970
H	-2.303210	-4.583250	-2.002640
H	-1.346390	-3.085940	-1.760910
Cl	2.673820	3.652190	-2.302410
Cl	-0.081380	3.179700	-3.307250
C	1.186050	2.677080	-2.142960
H	0.800080	2.793930	-1.132160
H	1.447330	1.643770	-2.347100
Rh	-0.005360	-0.200380	1.146760
O	-0.304540	-2.213600	1.248500
O	-0.210880	-2.304220	3.510100
O	-1.325420	-5.639700	-0.006810
O	2.222390	-3.570880	1.978580
N	0.488970	-4.741250	1.068240
C	-0.269230	-2.827700	2.366310
C	-0.388380	-4.345620	2.196710
H	-1.394640	-4.499100	1.798930
C	-0.127460	-5.369740	-0.024990
C	0.726760	-5.690560	-1.189250
C	0.189260	-6.378820	-2.261850
H	-0.844810	-6.700200	-2.210940
C	0.972170	-6.647100	-3.403080
H	0.530110	-7.177540	-4.240270
C	2.284240	-6.226330	-3.463710

H	2.884840	-6.421670	-4.348060
C	2.874030	-5.530680	-2.376750
C	4.217660	-5.072490	-2.390780
H	4.822700	-5.254430	-3.274540
C	4.744120	-4.394550	-1.310290
H	5.762040	-4.021510	-1.333220
C	3.951650	-4.146140	-0.171350
H	4.363940	-3.602730	0.667600
C	2.635060	-4.568560	-0.128970
C	2.076910	-5.268940	-1.223490
C	1.802510	-4.247540	1.047750
C	-0.280860	-5.245520	3.461490
C	-0.391310	-6.714340	3.008410
H	-1.299790	-6.888920	2.422670
H	-0.416500	-7.368430	3.886980
H	0.468200	-7.011200	2.397640
C	1.019440	-5.065220	4.262530
H	0.971940	-5.690380	5.162790
H	1.161540	-4.026650	4.564740
H	1.895760	-5.372190	3.686300
C	-1.492100	-4.923110	4.361380
H	-1.508220	-5.609510	5.215660
H	-2.435170	-5.045950	3.814360
H	-1.448430	-3.900170	4.739400
C	-0.131120	-0.299170	-0.846030
C	-1.129080	0.235930	-1.707480
C	-1.233520	-0.183540	-3.064600
C	-2.073240	1.183240	-1.223480
C	-2.247480	0.279370	-3.881420
H	-0.529040	-0.904160	-3.462920
C	-3.095620	1.640840	-2.035230
H	-1.995880	1.535950	-0.206090
C	-3.175850	1.180750	-3.350510
H	-2.335360	-0.062200	-4.905390
H	-3.823640	2.340550	-1.652260
Br	-4.586880	1.813460	-4.452850
C	0.841190	-1.248520	-1.428030
O	0.665620	-2.448970	-1.490520
O	1.974790	-0.623880	-1.836990
C	3.026110	-1.448630	-2.339840
H	3.915860	-1.276450	-1.737430
H	2.741880	-2.498450	-2.290660
C	3.309800	-1.072010	-3.794320
Cl	4.756470	-2.010700	-4.317410
Cl	3.643340	0.679660	-3.945340
Cl	1.905870	-1.511690	-4.829970
Rh	0.089690	-0.265220	3.602130
O	2.032440	-0.476550	1.149860
O	2.106700	-0.578980	3.415220
O	5.553670	-1.401670	-0.094020
O	3.493880	1.916810	2.263390
N	4.660470	0.272530	1.191520
C	2.635370	-0.564890	2.274220
C	4.147740	-0.742690	2.140470
H	4.253430	-1.682000	1.599100

C	5.371860	-0.196790	0.081820
C	5.886940	0.809760	-0.867830
C	6.635890	0.397250	-1.957360
H	6.822510	-0.662220	-2.090340
C	7.134050	1.342390	-2.874750
H	7.710380	1.004760	-3.730100
C	6.888330	2.688860	-2.691830
H	7.272930	3.417920	-3.400130
C	6.128730	3.146840	-1.585710
C	5.844710	4.519120	-1.356210
H	6.230480	5.256800	-2.054960
C	5.082270	4.910990	-0.275710
H	4.846550	5.957880	-0.118330
C	4.568860	3.952430	0.621250
H	3.948480	4.261340	1.451850
C	4.830900	2.607800	0.430770
C	5.617050	2.183990	-0.667120
C	4.266890	1.612860	1.364100
C	4.994040	-0.888920	3.439760
C	6.469140	-1.069860	3.029620
H	6.867070	-0.172100	2.543740
H	6.598360	-1.912650	2.342900
H	7.075400	-1.258180	3.922340
C	4.890720	0.309410	4.398060
H	5.469390	0.091410	5.304080
H	3.856090	0.506160	4.681150
H	5.300860	1.220430	3.953220
C	4.535210	-2.174600	4.159410
H	3.512880	-2.084260	4.528220
H	5.198700	-2.371950	5.009340
H	4.573330	-3.040770	3.489290
O	0.317080	1.831960	1.268450
O	0.393230	1.762100	3.532530
O	1.821530	5.472790	0.543670
O	-1.980150	3.409100	2.026910
N	-0.092200	4.482190	1.324870
C	0.416670	2.364620	2.428270
C	0.687880	3.868980	2.420860
H	1.721840	3.944620	2.079120
C	0.626300	5.249120	0.388230
C	-0.122560	5.750590	-0.782150
C	0.543360	6.476840	-1.754250
H	1.603050	6.662700	-1.626050
C	-0.147500	6.944950	-2.889100
H	0.389290	7.504950	-3.648140
C	-1.494880	6.688200	-3.041890
H	-2.026640	7.046900	-3.919180
C	-2.209900	5.950240	-2.064480
C	-3.594440	5.660290	-2.175350
H	-4.134980	6.014590	-3.049030
C	-4.245710	4.938820	-1.195410
H	-5.303720	4.715310	-1.288230
C	-3.540790	4.459340	-0.072130
H	-4.045720	3.855980	0.671280
C	-2.186390	4.713640	0.058730

C	-1.503190	5.472970	-0.921040
C	-1.448790	4.151970	1.207640
C	0.622990	4.629850	3.783050
C	0.829480	6.132720	3.510430
H	0.007340	6.553230	2.920950
H	1.763710	6.323030	2.974190
H	0.861730	6.672030	4.463440
C	-0.700420	4.445980	4.545640
H	-1.544310	4.877100	4.001320
H	-0.626690	4.957740	5.513110
H	-0.917490	3.392470	4.725730
C	1.798810	4.139060	4.654440
H	2.753600	4.243720	4.125610
H	1.682030	3.091190	4.932570
H	1.850870	4.741040	5.569010
O	-2.023750	0.089680	1.384570
O	-1.925690	0.118100	3.647660
O	-5.259740	1.873690	0.394720
O	-3.894890	-1.985330	2.410270
N	-4.711600	-0.052980	1.509310
C	-2.527340	0.241470	2.549730
C	-3.972660	0.736910	2.516720
H	-3.885890	1.727860	2.067090
C	-5.312120	0.646750	0.456850
C	-6.021190	-0.153160	-0.564030
C	-6.635690	0.486550	-1.626130
H	-6.572400	1.566330	-1.691920
C	-7.327180	-0.258470	-2.602350
H	-7.791730	0.257090	-3.436570
C	-7.411930	-1.632310	-2.501950
H	-7.946870	-2.206360	-3.253740
C	-6.790050	-2.322500	-1.429940
C	-6.818490	-3.734960	-1.300130
H	-7.363180	-4.317220	-2.038420
C	-6.149750	-4.363480	-0.269910
H	-6.168300	-5.445650	-0.188620
C	-5.422770	-3.609860	0.673950
H	-4.871940	-4.094390	1.472530
C	-5.392390	-2.229720	0.588300
C	-6.075930	-1.563840	-0.455610
C	-4.605500	-1.455020	1.567660
C	-4.724730	0.938850	3.865390
C	-6.152730	1.422010	3.543850
H	-6.732130	0.651260	3.023710
H	-6.143600	2.320170	2.917920
H	-6.677450	1.657890	4.476130
C	-4.809880	-0.323120	4.739460
H	-3.819350	-0.728600	4.951010
H	-5.403420	-1.108070	4.263090
H	-5.295070	-0.066490	5.689280
C	-4.001880	2.060590	4.641060
H	-3.897830	2.963090	4.028600
H	-3.003970	1.749850	4.953700
H	-4.581960	2.318620	5.534670

Rh-1a•(HFIP)$_3$ conformer A

6-31G(d)-SDD(Rh) SCF energy in gas phase (au):	-11283.497740
6-31G(d)-SDD(Rh) enthalpy in gas phase (au):	-11281.782550
6-31G(d)-SDD(Rh) free energy in gas phase (au):	-11282.055070
6-311+G(d,p)-SDD(Rh) SCF energy in implicit solvent (HFIP) (au):	-11288.542170
6-311+G(d,p)-SDD(Rh) enthalpy in implicit solvent (HFIP) (au):	-11286.826980
6-311+G(d,p)-SDD(Rh) free energy in implicit solvent (HFIP) (au):	-11287.094320

Cartesian coordinates

ATOM	X	Y	Z
O	-5.768740	2.628940	-3.285420
O	-0.985560	-1.630020	-2.370950
O	7.531410	1.613450	-2.235860
C	-5.153770	3.863880	-3.446060
C	-1.967040	-1.049740	-3.173490
C	6.135230	1.727900	-2.181240
C	-5.741940	4.909090	-2.484520
C	-3.622250	3.776120	-3.333920
C	-2.884350	-0.100700	-2.398470
C	-1.231030	-0.364840	-4.325780
C	5.665070	2.817900	-1.192310
C	5.581470	1.962630	-3.591370
Rh	-0.133490	-0.534310	1.327890
Rh	-1.319290	-1.338460	3.331310
O	-1.290140	-1.920190	0.267060
O	-2.301330	-2.695860	2.128760
O	-3.267640	-3.729650	-2.666000
O	-0.341760	-4.722670	0.695510
O	1.200680	-2.029360	1.703720
O	0.197390	-2.717560	3.612330
O	3.529570	-4.932970	0.684490
O	3.263770	-1.518140	3.709030
O	0.917220	0.661270	2.576560
O	-0.219170	0.017200	4.424950
O	4.215460	2.267580	3.873490
O	-0.257350	3.148170	3.646020
O	-1.616870	0.893410	1.123420
O	-2.773700	0.033090	2.871670
O	-5.135040	1.604700	-0.865580
O	-5.447960	0.483220	3.508640
N	-1.846710	-4.311210	-0.966070
N	3.427180	-3.258630	2.243480
N	1.975480	2.742160	3.841770
N	-5.022330	0.824740	1.272420
C	-2.059280	-2.735780	0.899600
C	-2.799050	-3.748440	0.015650
H	-3.471750	-3.145090	-0.599460
C	-2.180160	-4.186920	-2.329130
C	-1.166880	-4.617580	-3.312420
C	-1.399770	-4.429840	-4.663270
H	-2.337120	-3.984100	-4.975910
C	-0.429560	-4.810520	-5.612020
H	-0.618080	-4.637680	-6.666690
C	0.750060	-5.401280	-5.207030
H	1.496310	-5.698730	-5.938460

C	1.011140	-5.630970	-3.832170
C	2.199470	-6.250630	-3.363310
H	2.944960	-6.565320	-4.088180
C	2.410830	-6.443700	-2.013170
H	3.322520	-6.912350	-1.658550
C	1.461380	-5.996490	-1.070170
H	1.655640	-6.096690	-0.011940
C	0.302660	-5.371660	-1.493860
C	0.041350	-5.208380	-2.875990
C	-0.620690	-4.797280	-0.496800
C	-3.698900	-4.808680	0.724650
C	-4.299370	-5.725860	-0.359010
H	-3.526340	-6.316240	-0.862450
H	-4.839070	-5.153630	-1.119610
H	-4.998940	-6.427570	0.108210
C	-2.963900	-5.678110	1.759040
H	-3.692240	-6.337910	2.246230
H	-2.477810	-5.070870	2.523580
H	-2.203400	-6.308920	1.292020
C	-4.860920	-4.060350	1.406190
H	-5.398840	-3.431930	0.687950
H	-4.513250	-3.429550	2.224840
H	-5.577800	-4.783160	1.810250
C	1.106750	-2.765900	2.744200
C	2.187220	-3.848700	2.799930
H	1.872970	-4.557300	2.030030
C	3.941930	-3.845870	1.079860
C	4.982020	-3.098580	0.341470
C	5.456610	-3.592010	-0.862200
H	5.056480	-4.527230	-1.236900
C	6.425660	-2.875590	-1.592410
H	6.766240	-3.261660	-2.547220
C	6.936120	-1.689370	-1.101520
H	7.685650	-1.135670	-1.661110
C	6.481320	-1.162230	0.135310
C	6.975170	0.049730	0.686230
H	7.744110	0.592670	0.146310
C	6.462060	0.544030	1.867350
H	6.830940	1.478290	2.275830
C	5.439020	-0.143030	2.551860
H	5.010220	0.273650	3.452900
C	4.949070	-1.334840	2.050320
C	5.468680	-1.871570	0.848230
C	3.835820	-2.009650	2.744750
C	2.344020	-4.681910	4.105680
C	3.563650	-5.606620	3.931620
H	3.640690	-6.278630	4.793470
H	4.494830	-5.032950	3.871810
H	3.481810	-6.214910	3.025140
C	2.523050	-3.847360	5.384860
H	2.540010	-4.522680	6.249270
H	1.705010	-3.136810	5.516210
H	3.459110	-3.285230	5.374330
C	1.082320	-5.561560	4.240240
H	1.197490	-6.241450	5.092080

H	0.925200	-6.169440	3.340930
H	0.189550	-4.954150	4.400030
C	0.674600	0.676850	3.828350
C	1.693790	1.509070	4.612830
H	2.615800	0.929190	4.519460
C	3.303320	2.969490	3.450130
C	3.554300	4.095950	2.524450
C	4.833350	4.320740	2.052140
H	5.628500	3.656610	2.367630
C	5.090210	5.389990	1.168840
H	6.095400	5.535960	0.787870
C	4.073740	6.238850	0.783980
H	4.271640	7.064300	0.105680
C	2.751600	6.046800	1.260460
C	1.671790	6.894070	0.905390
H	1.858040	7.728340	0.235200
C	0.401760	6.661160	1.393450
H	-0.416790	7.317170	1.116000
C	0.150930	5.555070	2.230580
H	-0.846050	5.349950	2.599140
C	1.179670	4.697780	2.579430
C	2.495380	4.944240	2.127010
C	0.887790	3.492310	3.381910
C	1.461350	1.711930	6.139480
C	2.576380	2.631470	6.673710
H	2.498280	3.641080	6.255720
H	3.570230	2.241270	6.434080
H	2.490000	2.715270	7.762710
C	0.095580	2.320180	6.499830
H	0.001060	2.359740	7.592080
H	-0.726380	1.723170	6.101280
H	-0.008970	3.337900	6.116040
C	1.609020	0.332090	6.816130
H	1.566210	0.451380	7.904880
H	2.570250	-0.131310	6.563740
H	0.812480	-0.349100	6.511710
C	-2.643470	0.800190	1.880310
C	-3.822330	1.686220	1.459550
H	-3.591820	1.980590	0.441140
C	-5.525840	0.785880	-0.031700
C	-6.489590	-0.276130	-0.371250
C	-6.857320	-0.481300	-1.690950
H	-6.439830	0.147960	-2.467400
C	-7.749530	-1.521550	-2.021460
H	-8.022350	-1.677060	-3.060240
C	-8.270490	-2.338530	-1.038510
H	-8.959080	-3.138490	-1.297130
C	-7.915740	-2.155200	0.322570
C	-8.418090	-2.968260	1.370720
H	-9.109900	-3.769920	1.126560
C	-8.033930	-2.750070	2.678160
H	-8.421690	-3.381130	3.471660
C	-7.133090	-1.712510	2.995550
H	-6.822610	-1.533020	4.018940
C	-6.618380	-0.905820	1.996390

C	-7.003220	-1.109290	0.651110
C	-5.664290	0.171510	2.352900
C	-3.993490	3.026590	2.257980
C	-3.590930	2.929760	3.738380
H	-2.533020	2.682570	3.848940
H	-4.184580	2.181050	4.260650
H	-3.750600	3.905940	4.213220
C	-3.065280	4.037180	1.553070
H	-2.032530	3.675900	1.552510
H	-3.088440	4.997360	2.081220
H	-3.382300	4.210920	0.518380
C	-5.446270	3.511740	2.128850
H	-6.129740	2.869940	2.691490
H	-5.768380	3.549560	1.083590
H	-5.526150	4.524410	2.540220
C	0.944920	0.162320	-0.217290
C	1.693260	-0.573920	-1.179120
C	2.388700	0.086300	-2.230300
C	1.722810	-1.993840	-1.143130
C	3.061680	-0.633790	-3.202060
H	2.378980	1.168350	-2.287710
C	2.427080	-2.716390	-2.089600
H	1.204410	-2.513260	-0.351450
C	3.076280	-2.030260	-3.114850
H	3.566340	-0.135570	-4.017560
H	2.472210	-3.793740	-2.038100
Br	4.012050	-3.026110	-4.435060
C	1.043510	1.641610	-0.235240
O	1.808100	2.270670	0.463020
O	0.132830	2.180120	-1.079090
C	-0.130430	3.576470	-0.989180
H	-1.210980	3.686400	-1.050140
H	0.252800	3.986260	-0.054560
C	0.493900	4.341690	-2.165580
Cl	0.046140	3.579600	-3.719410
Cl	2.281940	4.396110	-2.024660
Cl	-0.168260	6.018740	-2.104540
F	-5.571160	4.550640	-1.194770
F	-7.061570	5.034630	-2.702090
F	-5.175840	6.120410	-2.653700
F	-3.232540	3.497740	-2.066310
F	-3.153740	2.805450	-4.130980
F	-3.036100	4.933670	-3.693780
F	-3.906500	0.308570	-3.168860
F	-2.249090	0.969970	-1.913610
F	-3.414830	-0.773760	-1.340060
F	-0.592810	-1.300460	-5.060970
F	-2.084100	0.287010	-5.138480
F	-0.311120	0.511310	-3.894270
F	5.600120	4.037190	-1.776410
F	6.530730	2.919620	-0.167890
F	4.454730	2.518840	-0.707650
F	6.247300	2.970110	-4.195080
F	5.745080	0.860200	-4.343970
F	4.272140	2.271620	-3.579840

H	-5.552630	2.261040	-2.397190
H	-1.257340	-1.696160	-1.424290
H	7.889740	2.414030	-2.655770
H	-5.366570	4.217770	-4.459810
H	-2.633700	-1.803160	-3.612540
H	5.748570	0.774930	-1.815400

Rh-1a•(HFIP)$_3$ conformer B

6-31G(d)-SDD(Rh) SCF energy in gas phase (au):	-11283.483670
6-31G(d)-SDD(Rh) enthalpy in gas phase (au):	-11281.767940
6-31G(d)-SDD(Rh) free energy in gas phase (au):	-11282.041500
6-311+G(d,p)-SDD(Rh) SCF energy in implicit solvent (HFIP) (au):	-11288.528960
6-311+G(d,p)-SDD(Rh) enthalpy in implicit solvent (HFIP) (au):	-11286.813230
6-311+G(d,p)-SDD(Rh) free energy in implicit solvent (HFIP) (au):	-11287.081610

Cartesian coordinates

ATOM	X	Y	Z
O	-4.321920	0.914690	2.184830
O	-5.979560	4.799470	1.267100
O	7.889900	1.955300	2.160310
C	-3.951660	0.356280	3.398350
C	-6.475780	4.039870	2.322790
C	6.556680	1.555470	2.325750
C	-4.783930	-0.887210	3.759760
C	-2.439940	0.071620	3.432940
C	-7.091860	2.721430	1.802510
C	-5.416640	3.913820	3.434490
C	6.402030	0.021040	2.399080
C	5.958970	2.249300	3.554890
Rh	0.199730	-0.780950	-1.112110
Rh	-0.891260	-1.823630	-3.057820
O	-1.370240	0.571210	-1.088430
O	-2.355850	-0.380050	-2.892770
O	-3.986140	3.319150	0.071560
O	-1.179190	2.477360	-3.419310
O	1.139790	0.422990	-2.463280
O	0.213540	-0.570920	-4.273820
O	2.584000	3.742870	-3.724600
O	3.452990	-0.740810	-3.850170
O	1.685490	-2.140620	-1.378870
O	0.680320	-3.160310	-3.133360
O	5.417190	-3.305480	-1.435420
O	1.245140	-4.940630	-0.587710
O	-0.871850	-2.107970	0.062380
O	-1.986580	-2.946110	-1.728350
O	-4.139720	-1.013220	0.324700
O	-4.110170	-5.218770	-1.316130
N	-2.630310	2.914990	-1.712910
N	3.055030	1.505590	-3.863920
N	3.337660	-4.176960	-1.036850
N	-4.212720	-3.238690	-0.150830
C	-2.252770	0.512400	-2.009220
C	-3.317930	1.616940	-1.929190
H	-3.833210	1.433310	-0.983990
C	-3.030940	3.680780	-0.614830

C	-2.275070	4.913500	-0.320770
C	-2.554190	5.642430	0.824830
H	-3.321400	5.296970	1.505600
C	-1.836880	6.822860	1.104200
H	-2.059830	7.377240	2.010070
C	-0.862690	7.274470	0.236850
H	-0.311840	8.185490	0.453940
C	-0.558410	6.558320	-0.948130
C	0.438720	6.974730	-1.869250
H	0.992420	7.886300	-1.662540
C	0.711410	6.231610	-2.999160
H	1.484850	6.544440	-3.691750
C	0.018840	5.027790	-3.246130
H	0.270150	4.420560	-4.104100
C	-0.947180	4.585240	-2.360630
C	-1.270020	5.351880	-1.214870
C	-1.567700	3.260700	-2.561450
C	-4.421090	1.657900	-3.033240
C	-5.357870	2.848020	-2.741960
H	-4.835080	3.807040	-2.827870
H	-5.795970	2.785320	-1.741890
H	-6.172590	2.852840	-3.474370
C	-3.879420	1.793120	-4.466650
H	-4.720080	1.732930	-5.168600
H	-3.167940	1.001280	-4.703480
H	-3.384430	2.754900	-4.625510
C	-5.257070	0.368260	-2.913660
H	-5.672930	0.267170	-1.907450
H	-4.664290	-0.520210	-3.137290
H	-6.096810	0.410760	-3.616610
C	0.980540	0.257480	-3.720610
C	1.762690	1.276970	-4.551200
H	1.216960	2.207810	-4.385630
C	3.302930	2.802940	-3.396150
C	4.454650	2.990810	-2.488010
C	4.707580	4.242720	-1.954440
H	4.049270	5.063420	-2.215040
C	5.791110	4.434730	-1.073810
H	5.961610	5.416990	-0.646120
C	6.627380	3.383410	-0.752670
H	7.464320	3.529420	-0.074720
C	6.399650	2.087570	-1.286200
C	7.221010	0.970390	-0.978840
H	8.067740	1.113030	-0.315120
C	6.924720	-0.278830	-1.483130
H	7.538430	-1.135500	-1.227670
C	5.808420	-0.468830	-2.322740
H	5.564380	-1.457490	-2.685670
C	4.998080	0.601510	-2.653900
C	5.281010	1.893560	-2.150860
C	3.804180	0.375500	-3.490990
C	1.829730	1.092390	-6.095130
C	2.750070	2.190260	-6.661660
H	2.738950	2.150170	-7.756450
H	3.786050	2.052780	-6.333480

H	2.426020	3.188490	-6.349610
C	2.341760	-0.281950	-6.557520
H	2.270270	-0.337590	-7.650850
H	1.750520	-1.092990	-6.129030
H	3.385230	-0.441350	-6.277420
C	0.405430	1.320240	-6.645490
H	0.428330	1.304410	-7.741130
H	0.008250	2.292300	-6.328970
H	-0.282360	0.546050	-6.301030
C	1.621670	-3.011380	-2.308340
C	2.909360	-3.839730	-2.416590
H	3.652140	-3.111180	-2.748950
C	4.609370	-3.753970	-0.628260
C	4.934110	-3.876000	0.811140
C	6.158520	-3.431340	1.272500
H	6.861810	-3.015450	0.561660
C	6.472060	-3.500920	2.645970
H	7.429800	-3.127800	2.993120
C	5.564170	-4.019700	3.545190
H	5.800990	-4.064010	4.604710
C	4.301730	-4.494130	3.105970
C	3.327530	-5.026470	3.988560
H	3.551750	-5.073720	5.050230
C	2.109190	-5.468150	3.511660
H	1.370690	-5.871420	4.197430
C	1.806550	-5.382100	2.136930
H	0.848250	-5.711180	1.754670
C	2.728950	-4.849400	1.254400
C	3.990420	-4.413150	1.717340
C	2.363030	-4.677020	-0.165510
C	2.955780	-5.005040	-3.446720
C	4.323740	-5.702020	-3.317480
H	4.432480	-6.192560	-2.343930
H	5.150250	-4.994500	-3.433500
H	4.415290	-6.473160	-4.090530
C	1.843490	-6.052820	-3.275970
H	1.921910	-6.788900	-4.085810
H	0.853630	-5.595520	-3.314390
H	1.932310	-6.585860	-2.326160
C	2.863810	-4.376880	-4.854100
H	3.011630	-5.154100	-5.612780
H	3.635370	-3.611190	-4.997490
H	1.890270	-3.912140	-5.020000
C	-1.809230	-2.775530	-0.491710
C	-2.866030	-3.318420	0.474250
H	-2.922480	-2.542710	1.230100
C	-4.815680	-1.986730	-0.019620
C	-6.257170	-1.885520	-0.277980
C	-6.965300	-0.780430	0.157400
H	-6.460140	-0.020710	0.735680
C	-8.334740	-0.654700	-0.150950
H	-8.872490	0.222500	0.195430
C	-8.971900	-1.623000	-0.902090
H	-10.022930	-1.516010	-1.157180
C	-8.273540	-2.774960	-1.352990

C	-8.873590	-3.799920	-2.128460
H	-9.921430	-3.707490	-2.401520
C	-8.140790	-4.898620	-2.532460
H	-8.611290	-5.675290	-3.127270
C	-6.783730	-5.031300	-2.170200
H	-6.207020	-5.900860	-2.465830
C	-6.164960	-4.050480	-1.415190
C	-6.896080	-2.909210	-1.011160
C	-4.752820	-4.237760	-0.989690
C	-2.499840	-4.617440	1.274920
C	-1.772960	-5.697090	0.460020
H	-0.854800	-5.309120	0.014170
H	-2.400970	-6.094250	-0.333760
H	-1.494400	-6.520330	1.131550
C	-1.548730	-4.142320	2.397220
H	-0.652220	-3.682400	1.975470
H	-1.244370	-4.996940	3.011930
H	-2.037780	-3.410650	3.050220
C	-3.767680	-5.189900	1.932230
H	-4.432810	-5.648810	1.196990
H	-4.321940	-4.419900	2.479340
H	-3.480630	-5.968920	2.647710
C	1.190850	0.048110	0.409730
C	1.624980	1.404710	0.527760
C	2.499560	1.815560	1.573070
C	1.194400	2.384050	-0.407080
C	2.914790	3.132540	1.681720
H	2.853400	1.091500	2.297700
C	1.627020	3.697260	-0.315670
H	0.531480	2.090550	-1.205390
C	2.470650	4.059430	0.732710
H	3.564140	3.450570	2.484900
H	1.328550	4.428050	-1.051090
Br	3.022230	5.872480	0.882550
C	1.626190	-0.909920	1.450940
O	2.531760	-1.702790	1.324150
O	0.834030	-0.803930	2.552180
C	0.917670	-1.823000	3.539180
H	-0.105570	-2.045730	3.836440
H	1.411400	-2.711610	3.144620
C	1.680600	-1.342760	4.781870
Cl	1.007650	0.210450	5.373040
Cl	3.425470	-1.140800	4.439600
Cl	1.461530	-2.616320	6.044180
F	-4.385500	-1.994510	3.080810
F	-6.076300	-0.683220	3.461780
F	-4.693810	-1.169540	5.071350
F	-2.043040	-0.611700	2.345480
F	-1.752470	1.223290	3.469730
F	-2.092650	-0.657370	4.522660
F	-6.378710	2.212870	0.781540
F	-8.331400	2.974390	1.322200
F	-7.216260	1.771830	2.743770
F	-4.228050	3.493430	2.972290
F	-5.223080	5.137660	3.971360

F	-5.792460	3.089080	4.433980
F	6.568510	-0.441750	3.660650
F	7.330660	-0.576780	1.634110
F	5.187520	-0.349570	1.972970
F	6.773050	2.103740	4.621210
F	5.823820	3.567500	3.323090
F	4.749040	1.757310	3.879350
H	-4.192670	0.264720	1.457080
H	-5.271110	4.278770	0.817810
H	8.375280	1.756510	2.979200
H	-4.158970	1.094520	4.177150
H	-7.303710	4.591300	2.778540
H	6.008720	1.890920	1.443600

Rh-1a•(DCM)$_4$

6-31G(d)–SDD(Rh) SCF energy in gas phase (au):	-12752.886960
6-31G(d)–SDD(Rh) enthalpy in gas phase (au):	-12751.252260
6-31G(d)–SDD(Rh) free energy in gas phase (au):	-12751.509320
6-311+G(d,p)–SDD(Rh) SCF energy in implicit solvent (DCM) (au):	-12757.437430
6-311+G(d,p)–SDD(Rh) enthalpy in implicit solvent (DCM) (au):	-12755.802740
6-311+G(d,p)–SDD(Rh) free energy in implicit solvent (DCM) (au):	-12756.059800

Cartesian coordinates

ATOM	X	Y	Z
Cl	-2.733550	2.642590	2.525550
Cl	-2.959110	5.573740	2.145650
C	-2.514460	4.000120	1.389580
H	-3.162620	3.850620	0.532650
H	-1.466260	4.034170	1.111930
Cl	3.290260	-3.368930	1.758720
Cl	0.956240	-2.796840	3.498050
C	1.733460	-2.503440	1.912170
H	1.066390	-2.847270	1.124590
H	1.932850	-1.440060	1.817530
Cl	0.687410	4.461790	2.882200
Cl	1.581800	6.866530	4.407340
C	0.191860	5.886310	3.833830
H	-0.360430	5.542780	4.705070
H	-0.422450	6.511490	3.193110
Cl	6.926100	0.673930	4.734940
Cl	9.201180	-0.589080	3.263420
C	7.441270	-0.228010	3.284040
H	7.212480	0.372820	2.409950
H	6.905250	-1.172830	3.258120
Rh	-0.802510	-0.264800	-1.372960
O	-1.299360	1.674790	-1.812820
O	-1.889830	1.212550	-3.950650
O	-2.328840	5.403390	-1.320490
O	0.825280	2.865220	-3.430840
N	-0.746490	4.206730	-2.465950
C	-1.640920	1.999140	-2.998770
C	-1.828180	3.505980	-3.194020
H	-2.723020	3.752920	-2.616870
C	-1.141250	5.158340	-1.514140
C	-0.069480	5.848300	-0.765840

C	-0.395750	6.883140	0.094400
H	-1.434510	7.177880	0.181630
C	0.609900	7.525500	0.844710
H	0.345000	8.339200	1.513090
C	1.925450	7.118620	0.745030
H	2.693060	7.596000	1.346730
C	2.296310	6.074320	-0.139600
C	3.635960	5.629080	-0.290300
H	4.411560	6.088070	0.316700
C	3.949540	4.631940	-1.191450
H	4.971400	4.284420	-1.299190
C	2.941220	4.034070	-1.975440
H	3.197870	3.246690	-2.671200
C	1.621810	4.423850	-1.832530
C	1.278290	5.445960	-0.916430
C	0.573420	3.764910	-2.638330
C	-2.088240	4.032610	-4.639640
C	-2.159860	5.571350	-4.584510
H	-2.905370	5.916530	-3.861370
H	-2.432800	5.958400	-5.572350
H	-1.193490	6.010030	-4.313130
C	-1.018020	3.622780	-5.665770
H	-1.320880	3.982330	-6.656950
H	-0.898100	2.539530	-5.709460
H	-0.044850	4.061140	-5.432140
C	-3.467380	3.505760	-5.088560
H	-3.725360	3.937940	-6.062110
H	-4.250660	3.794700	-4.376720
H	-3.471170	2.418530	-5.180270
C	-0.323800	0.232160	0.500300
C	-0.867450	-0.254690	1.722530
C	-0.544720	0.358440	2.966970
C	-1.791290	-1.335380	1.727530
C	-1.140330	-0.053550	4.144890
H	0.156630	1.184470	2.994460
C	-2.398820	-1.742080	2.902550
H	-2.033870	-1.828520	0.798280
C	-2.072230	-1.095650	4.094980
H	-0.898700	0.422170	5.087630
H	-3.119170	-2.546110	2.894650
Br	-2.900470	-1.675900	5.702800
C	0.642930	1.347000	0.572580
O	0.357840	2.525680	0.510260
O	1.908400	0.864080	0.665750
C	2.983780	1.797900	0.622700
H	3.523370	1.668090	-0.315710
H	2.613060	2.819680	0.700810
C	3.920860	1.500570	1.793060
Cl	5.364390	2.562150	1.614280
Cl	4.421690	-0.220740	1.758850
Cl	3.106180	1.842580	3.352850
Rh	-1.467390	-0.778600	-3.683100
O	1.104070	0.054440	-2.082560
O	0.469270	-0.386560	-4.215760
O	4.744870	1.425310	-2.355210

O	2.428150	-2.447420	-3.021680
N	3.681620	-0.546360	-2.846840
C	1.319550	-0.102740	-3.332220
C	2.761340	0.178740	-3.752300
H	2.904400	1.228220	-3.497480
C	4.690640	0.201830	-2.230970
C	5.690530	-0.539190	-1.436800
C	6.781350	0.135870	-0.915740
H	6.881120	1.196250	-1.117010
C	7.731440	-0.549360	-0.133790
H	8.588600	-0.016400	0.265400
C	7.575220	-1.896140	0.133950
H	8.304360	-2.415920	0.749570
C	6.471280	-2.618360	-0.386910
C	6.268580	-4.003570	-0.146380
H	6.988180	-4.538470	0.467840
C	5.176940	-4.658640	-0.676490
H	5.014970	-5.711860	-0.473220
C	4.239570	-3.963630	-1.468600
H	3.370330	-4.477610	-1.857900
C	4.408170	-2.615130	-1.726180
C	5.521690	-1.922200	-1.192170
C	3.424800	-1.901570	-2.566540
C	3.127550	0.035860	-5.261140
C	4.619360	0.387380	-5.430210
H	5.267630	-0.336610	-4.924030
H	4.848530	1.382950	-5.037160
H	4.877740	0.370930	-6.494520
C	2.885880	-1.371760	-5.831670
H	3.116800	-1.366580	-6.903910
H	1.850150	-1.685480	-5.697250
H	3.531930	-2.116640	-5.357720
C	2.303010	1.074080	-6.051220
H	1.238740	0.837850	-6.034890
H	2.642150	1.090130	-7.093460
H	2.430330	2.079870	-5.635610
O	-0.342250	-2.269950	-1.161600
O	-0.967080	-2.721520	-3.293520
O	1.694050	-5.600870	-0.225830
O	-2.577030	-4.098090	-0.864980
N	-0.463450	-4.909060	-0.573670
C	-0.537110	-3.050190	-2.158260
C	-0.095880	-4.497630	-1.944220
H	0.993720	-4.435520	-1.918080
C	0.567670	-5.432640	0.228450
C	0.229340	-5.746990	1.631610
C	1.211630	-6.238640	2.473720
H	2.207700	-6.389300	2.074790
C	0.916860	-6.515550	3.823220
H	1.699480	-6.890750	4.474800
C	-0.353620	-6.303490	4.318580
H	-0.579310	-6.513540	5.360670
C	-1.387260	-5.809300	3.482290
C	-2.711550	-5.585710	3.940550
H	-2.946430	-5.790630	4.981540

C	-3.686420	-5.116240	3.083400
H	-4.696390	-4.943650	3.440950
C	-3.376800	-4.826850	1.738360
H	-4.130660	-4.413440	1.080930
C	-2.089390	-5.015330	1.265780
C	-1.081080	-5.524920	2.118510
C	-1.763100	-4.640460	-0.124220
C	-0.447520	-5.538080	-3.055270
C	0.020340	-6.929520	-2.584600
H	-0.535280	-7.266790	-1.702660
H	1.086030	-6.937860	-2.338410
H	-0.155590	-7.658450	-3.383160
C	-1.946460	-5.614830	-3.394050
H	-2.535020	-5.972610	-2.545230
H	-2.087340	-6.320970	-4.221570
H	-2.341760	-4.643770	-3.694360
C	0.357380	-5.171590	-4.320500
H	1.427390	-5.086490	-4.097270
H	0.026820	-4.223970	-4.746580
H	0.229910	-5.957890	-5.073500
O	-2.746820	-0.660570	-0.878990
O	-3.361700	-1.193950	-2.992840
O	-5.259610	-2.348380	1.444470
O	-5.014010	1.105540	-1.530550
N	-5.281440	-0.640400	-0.083780
C	-3.574330	-1.081460	-1.758440
C	-4.880000	-1.588030	-1.146410
H	-4.567610	-2.479880	-0.598610
C	-5.394040	-1.147930	1.216510
C	-5.682810	-0.179780	2.295500
C	-5.795090	-0.626830	3.600440
H	-5.665840	-1.683500	3.803080
C	-6.066130	0.281520	4.643360
H	-6.135660	-0.083460	5.662870
C	-6.237300	1.624220	4.373060
H	-6.447590	2.324400	5.177080
C	-6.125460	2.118740	3.048670
C	-6.255170	3.493010	2.724520
H	-6.469030	4.201490	3.519860
C	-6.090520	3.932730	1.426700
H	-6.176130	4.988940	1.194820
C	-5.782640	3.018800	0.398390
H	-5.631920	3.355190	-0.621920
C	-5.655870	1.669360	0.679910
C	-5.832580	1.196570	2.000950
C	-5.294130	0.730020	-0.400380
C	-6.024760	-2.047710	-2.095400
C	-7.224870	-2.462710	-1.222180
H	-7.646840	-1.606360	-0.684780
H	-6.943540	-3.221080	-0.484370
H	-8.014540	-2.877470	-1.858170
C	-6.482590	-0.980130	-3.102310
H	-5.649350	-0.622510	-3.709440
H	-6.932800	-0.118040	-2.603550
H	-7.238140	-1.417550	-3.766540

C	-5.525800	-3.296270	-2.854100
H	-5.165830	-4.064770	-2.160480
H	-4.709680	-3.049050	-3.534840
H	-6.349600	-3.722510	-3.438200

Rh-1a•(HFIP)$_4$

6-31G(d)-SDD(Rh) SCF energy in gas phase (au):	-12073.325240
6-31G(d)-SDD(Rh) enthalpy in gas phase (au):	-12071.534820
6-31G(d)-SDD(Rh) free energy in gas phase (au):	-12071.827490
6-311+G(d,p)-SDD(Rh) SCF energy in implicit solvent (HFIP) (au):	-12078.645870
6-311+G(d,p)-SDD(Rh) enthalpy in implicit solvent (HFIP) (au):	-12076.855450
6-311+G(d,p)-SDD(Rh) free energy in implicit solvent (HFIP) (au):	-12077.142950

Cartesian coordinates

ATOM	X	Y	Z
O	5.410430	-0.536250	-2.879050
O	5.829460	4.343210	-1.556110
O	0.881060	2.355180	-0.912840
O	-7.862310	0.122390	-4.948780
C	5.681240	-1.535950	-3.802150
C	6.513430	3.309930	-2.190540
C	1.501620	1.987290	-2.107220
C	-6.669370	0.316590	-4.242630
C	6.699520	-2.561980	-3.272480
C	4.392610	-2.213000	-4.308970
C	6.855050	2.172810	-1.214840
C	5.749240	2.821740	-3.431410
C	2.156750	0.606490	-2.013190
C	0.468250	2.073590	-3.234950
C	-6.306340	-0.962310	-3.472120
C	-5.561040	0.803160	-5.183670
Rh	-0.609280	-0.460280	1.558260
Rh	0.468710	-1.113030	3.676440
O	1.000030	0.850270	1.297770
O	1.918500	0.305000	3.285780
O	3.673950	3.387270	-0.252460
O	0.742800	3.139260	3.218410
O	-1.516490	0.989440	2.677160
O	-0.694080	0.283010	4.660160
O	-3.146070	4.498730	3.674080
O	-3.952020	0.000800	3.764660
O	-2.073590	-1.770610	2.039680
O	-1.079630	-2.444310	3.959910
O	-5.713590	-2.985610	2.136260
O	-1.490140	-4.665010	1.793130
O	0.475730	-1.983690	0.652650
O	1.597920	-2.408520	2.572420
O	4.446790	-1.800270	-0.701250
O	3.469940	-4.446360	2.886100
N	2.270350	3.314050	1.534740
N	-3.512960	2.239730	3.710410
N	-3.627230	-3.921360	2.022430
N	3.836550	-2.990930	1.141350
C	1.859430	1.001730	2.246200
C	2.936780	2.063290	1.975030

H	3.452840	1.716120	1.077300
C	2.711350	3.881580	0.336580
C	1.997650	5.060710	-0.178870
C	2.383020	5.629880	-1.381370
H	3.238840	5.228910	-1.913590
C	1.667110	6.724980	-1.906090
H	1.972260	7.154270	-2.854800
C	0.587610	7.248220	-1.223630
H	0.034940	8.089800	-1.631820
C	0.179610	6.701470	0.020000
C	-0.918180	7.207670	0.764750
H	-1.474020	8.052090	0.366910
C	-1.282660	6.632620	1.965710
H	-2.127600	7.012180	2.529440
C	-0.580180	5.516370	2.465350
H	-0.898380	5.044760	3.384960
C	0.486180	4.990480	1.756910
C	0.895580	5.582490	0.537570
C	1.147570	3.765780	2.246900
C	4.039070	2.266560	3.060140
C	4.996690	3.379800	2.589340
H	4.485300	4.343930	2.495480
H	5.463870	3.142070	1.630440
H	5.791860	3.502600	3.332710
C	3.487490	2.645360	4.444690
H	4.323280	2.720970	5.150620
H	2.781960	1.901740	4.816330
H	2.981430	3.614810	4.424310
C	4.853790	0.960220	3.145870
H	5.270460	0.698530	2.166970
H	4.251860	0.124650	3.506700
H	5.695160	1.094790	3.834110
C	-1.449850	0.997350	3.953240
C	-2.349030	2.053750	4.608500
H	-1.790960	2.987190	4.499380
C	-3.727910	3.524360	3.201550
C	-4.655570	3.654090	2.055670
C	-4.925830	4.907100	1.532840
H	-4.491960	5.776460	2.013200
C	-5.730190	5.038440	0.384560
H	-5.929230	6.025240	-0.019450
C	-6.238410	3.919130	-0.245170
H	-6.838880	4.023840	-1.145170
C	-5.981880	2.621170	0.260760
C	-6.461770	1.434940	-0.355750
H	-7.073430	1.522900	-1.250650
C	-6.178760	0.195040	0.176050
H	-6.531180	-0.705390	-0.310360
C	-5.424180	0.081580	1.362020
H	-5.223780	-0.889610	1.792570
C	-4.941230	1.215670	1.986260
C	-5.191610	2.497470	1.442080
C	-4.133070	1.075550	3.211150
C	-2.653460	1.918080	6.133010
C	-3.717740	2.968720	6.500640

H	-3.880020	2.960080	7.584150
H	-4.676350	2.749820	6.018170
H	-3.409910	3.976000	6.204090
C	-3.148970	0.529180	6.569020
H	-3.252120	0.517500	7.661030
H	-2.447390	-0.255840	6.281700
H	-4.119190	0.290800	6.130090
C	-1.352750	2.257310	6.891730
H	-1.543930	2.250660	7.970710
H	-0.987100	3.256000	6.622240
H	-0.565190	1.532730	6.675690
C	-2.005590	-2.469890	3.106080
C	-3.269720	-3.308330	3.324730
H	-4.050000	-2.559640	3.482710
C	-4.884080	-3.611300	1.484920
C	-5.161440	-4.064730	0.103150
C	-6.375400	-3.759440	-0.483190
H	-7.107680	-3.208190	0.096480
C	-6.645400	-4.155470	-1.809930
H	-7.594260	-3.889710	-2.262880
C	-5.709240	-4.865540	-2.533030
H	-5.917130	-5.167790	-3.555780
C	-4.461260	-5.215460	-1.955230
C	-3.470710	-5.963150	-2.643090
H	-3.671330	-6.287140	-3.660170
C	-2.269560	-6.271710	-2.036540
H	-1.520490	-6.844510	-2.573790
C	-1.995860	-5.827100	-0.726680
H	-1.047990	-6.044940	-0.250000
C	-2.936130	-5.085340	-0.033760
C	-4.187340	-4.789610	-0.621670
C	-2.608890	-4.562430	1.308320
C	-3.326410	-4.264140	4.550750
C	-4.678530	-5.002270	4.511600
H	-4.745210	-5.670050	3.645650
H	-5.518960	-4.302740	4.465740
H	-4.786720	-5.615680	5.412930
C	-2.191130	-5.299570	4.608100
H	-2.281500	-5.873980	5.538360
H	-1.210580	-4.821360	4.586680
H	-2.241700	-6.003430	3.773530
C	-3.290610	-3.387420	5.820410
H	-3.449060	-4.015900	6.704230
H	-4.080720	-2.627620	5.800100
H	-2.330690	-2.878940	5.926940
C	1.429380	-2.503550	1.325870
C	2.516980	-3.191290	0.489090
H	2.595620	-2.558350	-0.391520
C	4.734040	-2.188430	0.432920
C	5.993670	-1.797190	1.088760
C	6.916860	-1.026680	0.403250
H	6.751890	-0.790400	-0.637260
C	8.057860	-0.532290	1.067050
H	8.754530	0.092280	0.518570
C	8.268120	-0.813920	2.401530

H	9.139100	-0.416870	2.916300
C	7.357410	-1.626360	3.126010
C	7.528120	-1.954550	4.495180
H	8.392410	-1.562450	5.024660
C	6.615990	-2.760750	5.146730
H	6.756890	-3.003780	6.195290
C	5.502070	-3.283690	4.457680
H	4.791280	-3.935950	4.953020
C	5.296870	-2.968230	3.125890
C	6.211030	-2.132690	2.445870
C	4.134740	-3.546160	2.410170
C	2.206110	-4.616850	-0.087290
C	1.582520	-5.602430	0.911350
H	0.673930	-5.196630	1.362250
H	2.273130	-5.859830	1.711410
H	1.310350	-6.521660	0.375090
C	1.210030	-4.394150	-1.242440
H	0.291800	-3.937340	-0.866860
H	0.959530	-5.353560	-1.709610
H	1.627050	-3.740280	-2.014450
C	3.510070	-5.193760	-0.663050
H	4.231160	-5.414930	0.130230
H	3.975420	-4.509340	-1.379090
H	3.292600	-6.132880	-1.184480
C	-1.689380	0.046380	-0.069800
C	-2.159000	1.332550	-0.462780
C	-2.979930	1.497530	-1.615670
C	-1.747360	2.502110	0.231500
C	-3.313800	2.754150	-2.083690
H	-3.333130	0.628750	-2.154720
C	-2.106370	3.760940	-0.215230
H	-1.144940	2.402110	1.121120
C	-2.853660	3.875380	-1.386020
H	-3.898820	2.872630	-2.985780
H	-1.793820	4.645680	0.316550
Br	-3.223500	5.607980	-2.068710
C	-2.215650	-1.110460	-0.836050
O	-3.288380	-1.630070	-0.600110
O	-1.350540	-1.524040	-1.786660
C	-1.732560	-2.646790	-2.576380
H	-1.192900	-3.525710	-2.226380
H	-2.807100	-2.819440	-2.507760
C	-1.350950	-2.376570	-4.033330
Cl	0.388110	-2.026240	-4.204390
Cl	-2.321220	-1.008750	-4.671340
Cl	-1.762590	-3.873340	-4.962040
F	6.166480	-3.392260	-2.351550
F	7.730450	-1.919770	-2.690080
F	7.198540	-3.317490	-4.268770
F	3.599790	-2.600150	-3.288590
F	3.686030	-1.357380	-5.068590
F	4.661190	-3.299890	-5.059730
F	5.753110	1.540590	-0.768790
F	7.495210	2.672210	-0.137940
F	7.668040	1.258050	-1.780800

F	4.537320	2.329060	-3.106930
F	5.550370	3.860300	-4.265880
F	6.429240	1.877150	-4.103390
F	2.627250	0.209390	-3.202000
F	1.299930	-0.326250	-1.559850
F	3.185270	0.657600	-1.139900
F	-0.166020	3.261380	-3.171780
F	1.052660	1.981920	-4.442240
F	-0.463330	1.104740	-3.149650
F	-6.246760	-2.017460	-4.310710
F	-7.273830	-1.211110	-2.566530
F	-5.138020	-0.871770	-2.821590
F	-5.257620	-0.138150	-6.097810
F	-5.963530	1.904140	-5.830550
F	-4.432720	1.111360	-4.507840
H	5.006040	-0.928020	-2.071070
H	5.021320	3.985430	-1.119840
H	0.982700	1.678100	-0.202460
H	-7.718150	-0.597040	-5.587620
H	6.149110	-1.063640	-4.671240
H	7.467520	3.707640	-2.550170
H	2.302840	2.691350	-2.357060
H	-6.843480	1.106650	-3.508720

Rh-2a·(DCM) carbonyl

6-31G(d)–SDD(Rh) SCF energy in gas phase (au):	-17541.717030
6-31G(d)–SDD(Rh) enthalpy in gas phase (au):	-17540.072850
6-31G(d)–SDD(Rh) free energy in gas phase (au):	-17540.334800
6-311+G(d,p)–SDD(Rh) SCF energy in implicit solvent (DCM) (au):	-17546.605290
6-311+G(d,p)–SDD(Rh) enthalpy in implicit solvent (DCM) (au):	-17544.961110
6-311+G(d,p)–SDD(Rh) free energy in implicit solvent (DCM) (au):	-17545.223060

Cartesian coordinates

ATOM	X	Y	Z
Cl	2.289190	2.546280	-4.456290
Cl	3.966840	0.919620	-2.611670
C	2.875820	2.329150	-2.777610
Rh	-0.158230	0.080150	3.025810
Rh	0.110190	-0.027990	0.576960
O	1.200620	1.706880	0.710800
O	0.974010	1.801000	2.962370
O	3.520090	4.749530	-0.416680
O	-0.795310	3.940560	0.910990
O	-1.803160	1.234680	2.690840
O	-1.660340	1.049270	0.438140
O	-4.120390	-0.626360	1.538180
O	-5.092390	3.107700	-0.949690
O	1.501640	-1.118610	3.222330
O	1.818040	-1.087840	0.980420
O	4.870310	-3.191880	-0.076730
O	4.108050	0.578660	2.411160
O	-0.993760	-1.728030	0.674060
O	-1.314070	-1.626510	2.915350
O	0.468260	-4.334250	1.554500
O	-3.199400	-3.771380	-1.118910

N	1.472440	4.331180	0.595980
N	-4.449420	1.451830	0.552150
N	4.425300	-1.530960	1.482950
N	-1.628640	-4.198380	0.547060
C	-6.058260	0.863770	-0.998130
C	-7.012380	0.846820	-2.003890
C	-7.641000	-0.384610	-2.292820
C	-7.331340	-1.539040	-1.547160
C	-6.386520	-1.488380	-0.496560
C	-5.750430	-0.280170	-0.256590
C	-4.690100	0.084190	0.737250
C	1.327050	2.270010	1.849480
C	0.123530	4.314180	0.213400
C	-3.500960	2.257290	1.331280
H	-3.214880	3.077210	0.668230
C	-2.215760	1.436600	1.519180
C	0.088260	4.822840	-1.191370
C	-0.971650	4.940640	-2.076350
C	-0.685440	5.372740	-3.389790
C	0.628290	5.709170	-3.768890
C	4.637730	-0.147900	1.597780
C	5.585610	0.208680	0.497060
C	6.170850	1.423010	0.175050
C	6.991020	1.476080	-0.974030
C	7.192440	0.328080	-1.765500
C	6.581670	-0.898440	-1.421440
C	5.793850	-0.933040	-0.281230
C	5.013400	-2.055290	0.325650
C	1.690560	5.592390	-2.846490
C	1.397060	5.127040	-1.573950
C	3.399930	-2.271190	2.229170
H	3.180620	-3.138520	1.596720
C	2.127690	-1.416210	2.176990
C	2.306370	4.754500	-0.444820
C	2.004970	3.648350	1.781860
H	3.050960	3.444550	1.525240
C	-5.188470	1.979310	-0.511710
C	-2.092510	-4.128580	-0.784990
C	-0.945000	-4.562900	-1.644080
C	-0.836320	-4.679360	-3.022710
C	0.392430	-5.135960	-3.550540
C	1.456890	-5.485360	-2.695790
C	1.342600	-5.309780	-1.300040
C	0.136940	-4.838900	-0.805270
C	-0.253840	-4.464630	0.587130
C	-2.311030	-3.417240	1.591850
H	-3.217660	-3.055490	1.095990
C	-1.464060	-2.153030	1.783890
C	2.010830	4.514110	3.064130
C	0.602020	4.780040	3.640820
C	2.886950	3.814320	4.134080
C	2.665910	5.878130	2.732260
H	0.107070	3.833020	3.867870
H	-0.015970	5.292380	2.895520
C	0.705840	5.643590	4.910730

H	3.891400	3.639090	3.723770
H	2.467200	2.836710	4.379900
C	2.979340	4.683560	5.399940
H	2.068720	6.401570	1.975070
H	3.661770	5.718030	2.298620
C	2.766460	6.751940	3.995600
H	-0.303680	5.809960	5.309100
C	1.563970	4.916040	5.961800
C	1.355470	6.994780	4.562630
H	3.591340	4.161000	6.145930
C	3.625840	6.035390	5.052030
H	3.229010	7.710830	3.729130
H	1.101910	3.954570	6.223050
H	1.617430	5.511710	6.883030
H	0.739820	7.529070	3.826190
H	1.414210	7.630250	5.456400
H	3.712910	6.658820	5.951970
H	4.642780	5.878230	4.667790
C	-4.134360	2.882730	2.603830
C	-4.588110	1.838760	3.650140
C	-3.113250	3.859470	3.241360
C	-5.377040	3.711770	2.188760
H	-3.744520	1.214090	3.949280
H	-5.341600	1.173970	3.208560
C	-5.191690	2.548450	4.877030
H	-2.812290	4.606040	2.496040
H	-2.210990	3.320810	3.533320
C	-3.726250	4.552900	4.468630
H	-6.133810	3.054740	1.741300
H	-5.098940	4.445940	1.423090
C	-5.993830	4.419710	3.409140
H	-5.490460	1.790960	5.612760
C	-4.141520	3.487460	5.499820
C	-6.421570	3.367540	4.447650
H	-2.973970	5.220390	4.909260
C	-4.957630	5.367210	4.038430
H	-6.869330	4.993620	3.079650
H	-3.262440	2.909950	5.815990
H	-4.553250	3.969630	6.396680
H	-7.185340	2.703520	4.020030
H	-6.873850	3.860350	5.318700
H	-5.396530	5.883590	4.902710
H	-4.663510	6.139170	3.314630
C	-2.774820	-4.212320	2.830830
C	-1.637510	-4.693620	3.759160
C	-3.748130	-3.319640	3.645060
C	-3.559250	-5.450770	2.332660
H	-1.056200	-3.835370	4.110020
H	-0.948190	-5.339010	3.206980
C	-2.225330	-5.470040	4.953170
H	-4.556430	-2.967310	2.993260
H	-3.221740	-2.431670	4.002820
C	-4.329790	-4.103670	4.832970
H	-2.893170	-6.100230	1.751240
H	-4.363350	-5.133450	1.653990

C	-4.144250	-6.235140	3.519890
H	-1.402520	-5.798100	5.601230
C	-3.181020	-4.563320	5.748860
C	-2.997120	-6.698400	4.436970
H	-5.009440	-3.448200	5.392360
C	-5.101750	-5.329430	4.314600
H	-4.689560	-7.107940	3.138420
H	-2.637290	-3.690470	6.134290
H	-3.581490	-5.105640	6.616110
H	-2.319440	-7.363920	3.885120
H	-3.400240	-7.275000	5.280290
H	-5.538210	-5.887830	5.153560
H	-5.933770	-5.006190	3.673930
C	3.864080	-2.820520	3.593120
C	4.187810	-1.730230	4.637920
C	2.757700	-3.756600	4.143480
C	5.138330	-3.670150	3.361270
H	3.310960	-1.099810	4.804000
H	4.985120	-1.078170	4.261930
C	4.637040	-2.386550	5.957250
H	2.528960	-4.530850	3.399630
H	1.836740	-3.191080	4.306460
C	3.215960	-4.403000	5.461510
H	5.940900	-3.033940	2.966390
H	4.939180	-4.439760	2.603830
C	5.599310	-4.322650	4.676460
H	4.851920	-1.598920	6.690470
C	3.515930	-3.297870	6.491060
C	5.904750	-3.224360	5.711280
H	2.413630	-5.050710	5.837130
C	4.484610	-5.237780	5.214380
H	6.503970	-4.913520	4.484170
H	2.610080	-2.706700	6.680740
H	3.818120	-3.745000	7.447690
H	6.717010	-2.579800	5.348400
H	6.248150	-3.678290	6.650500
H	4.810590	-5.719200	6.146100
H	4.273140	-6.038570	4.492960
C	0.266490	-0.142150	-1.429050
C	1.073250	-0.932300	-2.297400
C	1.030350	-0.731250	-3.709720
C	2.003320	-1.885410	-1.795020
C	1.894870	-1.395660	-4.556560
H	0.334730	-0.019090	-4.134770
C	2.883130	-2.543640	-2.636940
H	2.043660	-2.061130	-0.730450
C	2.834670	-2.279340	-4.010740
H	1.874250	-1.215140	-5.624300
H	3.618990	-3.227440	-2.231720
Br	4.083530	-3.101200	-5.165470
C	-0.623140	0.834800	-2.096830
O	-0.308980	1.984660	-2.336170
O	-1.812410	0.277180	-2.389300
C	-2.769220	1.069790	-3.082530
H	-3.495300	1.468810	-2.375100

H	-2.286850	1.895900	-3.606710
C	-3.476140	0.157490	-4.086380
Cl	-4.763300	1.126560	-4.872930
Cl	-4.185090	-1.259070	-3.257540
Cl	-2.298400	-0.408240	-5.327860
H	2.005160	2.168300	-2.146430
H	3.440830	3.212800	-2.495310
Cl	5.895910	2.832850	1.135430
Cl	7.721110	2.974620	-1.428630
Cl	8.168670	0.422370	-3.188470
Cl	6.778980	-2.302220	-2.413230
Cl	2.672390	-5.647150	-0.249780
Cl	2.926990	-6.111380	-3.354310
Cl	0.593600	-5.255560	-5.264530
Cl	-2.148820	-4.253530	-4.062170
Cl	-6.054590	-2.887170	0.460600
Cl	-8.115840	-3.035320	-1.912990
Cl	-8.801380	-0.467980	-3.571010
Cl	-7.441110	2.288150	-2.856120
Cl	3.312290	5.994620	-3.299220
Cl	0.953930	6.242350	-5.380380
Cl	-1.966800	5.465190	-4.550060
Cl	-2.581150	4.517060	-1.616060

Rh-2a•(DCM) ester

6-31G(d)–SDD(Rh) SCF energy in gas phase (au):	-17541.725140
6-31G(d)–SDD(Rh) enthalpy in gas phase (au):	-17540.080890
6-31G(d)–SDD(Rh) free energy in gas phase (au):	-17540.341840
6-311+G(d,p)–SDD(Rh) SCF energy in implicit solvent (DCM) (au):	-17546.610620
6-311+G(d,p)–SDD(Rh) enthalpy in implicit solvent (DCM) (au):	-17544.966360
6-311+G(d,p)–SDD(Rh) free energy in implicit solvent (DCM) (au):	-17545.227320

Cartesian coordinates

ATOM	X	Y	Z
Cl	4.890400	-2.354020	-2.112110
Cl	2.491370	-2.443310	-0.356890
C	4.020800	-3.241280	-0.818390
Rh	-1.409930	1.255650	2.649130
Rh	-0.515040	0.537010	0.466030
O	-2.290950	1.158520	-0.304380
O	-3.119830	1.883630	1.674160
O	-4.705990	1.976360	-3.251730
O	-4.600270	-0.805360	0.388840
O	-2.205810	-0.636980	2.825870
O	-1.341590	-1.297960	0.834810
O	0.115700	-2.809020	3.439930
O	-2.026470	-4.837110	-0.059560
O	-0.615770	3.125870	2.295050
O	0.219970	2.449680	0.305660
O	2.637450	5.298470	-0.797250
O	-1.749900	4.005240	-1.232150
O	1.189270	-0.055240	1.447620
O	0.364470	0.588250	3.457810
O	3.055450	2.228760	2.160320
O	4.663440	-2.069550	2.182110

N	-4.766170	0.869540	-1.211150
N	-1.311390	-3.794020	1.888580
N	0.374680	4.797680	-0.689560
N	3.672770	0.012050	2.489680
C	0.248480	-5.111270	0.797600
C	0.935060	-5.938310	-0.077660
C	2.292750	-6.207670	0.203240
C	2.903420	-5.696340	1.366280
C	2.176350	-4.878320	2.258560
C	0.867110	-4.569330	1.925400
C	-0.096950	-3.620930	2.564810
C	-3.194960	1.661420	0.434900
C	-4.746670	-0.452480	-0.763900
C	-2.488460	-2.927610	2.057830
H	-3.073550	-3.097770	1.149930
C	-1.983300	-1.484400	1.925690
C	-4.880540	-1.302940	-1.987340
C	-4.994290	-2.678020	-2.119310
C	-4.996650	-3.219250	-3.424760
C	-4.852000	-2.378580	-4.547170
C	-0.589430	4.195690	-1.521630
C	0.126050	3.841300	-2.786470
C	-0.320390	3.124660	-3.888930
C	0.627860	2.813280	-4.889240
C	1.959140	3.261930	-4.788700
C	2.385560	3.999160	-3.662590
C	1.452980	4.261210	-2.670430
C	1.629170	4.864780	-1.313290
C	-4.764420	-0.976720	-4.388700
C	-4.806140	-0.465710	-3.101900
C	0.297470	4.773970	0.777050
H	1.339130	4.911310	1.092990
C	-0.076380	3.334260	1.177770
C	-4.744840	0.948250	-2.612650
C	-4.452720	2.030110	-0.367050
H	-4.110670	2.790900	-1.077990
C	-1.162710	-4.613170	0.763240
C	4.627960	-0.873410	1.980100
C	5.548120	-0.042000	1.141110
C	6.682770	-0.396390	0.427690
C	7.318570	0.603110	-0.342270
C	6.833500	1.925850	-0.339150
C	5.696790	2.274960	0.425960
C	5.065080	1.268400	1.141330
C	3.819250	1.304260	1.974290
C	2.557620	-0.424840	3.334730
H	2.512740	-1.508500	3.188370
C	1.255850	0.104890	2.712960
C	-5.664600	2.613880	0.390190
C	-6.228370	1.684700	1.488250
C	-5.246580	3.964310	1.024280
C	-6.788720	2.897140	-0.637080
H	-5.447540	1.450870	2.216710
H	-6.547300	0.734320	1.045680
C	-7.427640	2.358300	2.181830

H	-4.848200	4.626600	0.243210
H	-4.445990	3.801590	1.748770
C	-6.448910	4.628000	1.716180
H	-7.107700	1.956720	-1.104070
H	-6.404680	3.537690	-1.441430
C	-7.994060	3.563300	0.050160
H	-7.808220	1.688500	2.963500
C	-6.980740	3.689160	2.814760
C	-8.535570	2.629450	1.147850
H	-6.122380	5.575120	2.165200
C	-7.556020	4.896460	0.682540
H	-8.774940	3.747520	-0.698660
H	-6.197210	3.505080	3.562070
H	-7.824110	4.160000	3.337630
H	-8.875110	1.683580	0.704150
H	-9.406220	3.087840	1.635850
H	-8.414280	5.385220	1.162840
H	-7.187500	5.579510	-0.094600
C	-3.392060	-3.303770	3.251870
C	-2.734610	-3.126340	4.638740
C	-4.681420	-2.446560	3.181770
C	-3.795380	-4.790870	3.089680
H	-2.417530	-2.088800	4.770260
H	-1.835050	-3.747220	4.710870
C	-3.724020	-3.534970	5.746640
H	-5.151230	-2.569040	2.199200
H	-4.429690	-1.387690	3.279900
C	-5.659930	-2.861480	4.292970
H	-2.900500	-5.425260	3.132300
H	-4.249720	-4.947310	2.101790
C	-4.777620	-5.209970	4.197500
H	-3.241120	-3.393520	6.721910
C	-4.988780	-2.661500	5.663830
C	-4.110170	-5.014820	5.571200
H	-6.558380	-2.234920	4.225750
C	-6.045100	-4.340620	4.116000
H	-5.042170	-6.266230	4.059850
H	-4.724730	-1.604420	5.800960
H	-5.685550	-2.929570	6.469550
H	-3.216320	-5.648780	5.649610
H	-4.797130	-5.325420	6.369770
H	-6.759300	-4.644030	4.893150
H	-6.540480	-4.486470	3.146460
C	2.798260	-0.174740	4.846780
C	2.777800	1.320020	5.239690
C	1.725720	-0.940980	5.662840
C	4.184990	-0.754640	5.226050
H	1.817630	1.764810	4.966340
H	3.552690	1.863940	4.686890
C	3.027080	1.471460	6.752370
H	1.747360	-2.002230	5.388460
H	0.730560	-0.570330	5.410490
C	1.982310	-0.777520	7.170260
H	4.973340	-0.229330	4.671890
H	4.241070	-1.811070	4.935310

C	4.445550	-0.601570	6.734900
H	2.996970	2.537410	7.011340
C	1.936930	0.717570	7.535800
C	4.407390	0.891280	7.108970
H	1.201940	-1.316540	7.722410
C	3.362450	-1.355950	7.525250
H	5.433970	-1.017610	6.968620
H	0.948450	1.131690	7.296500
H	2.091170	0.848080	8.615470
H	5.195200	1.435190	6.569940
H	4.605960	1.015300	8.181940
H	3.550290	-1.263410	8.603370
H	3.394990	-2.426840	7.282010
C	-0.517890	5.925210	1.423190
C	-2.049070	5.732760	1.350580
C	-0.093370	6.058210	2.908280
C	-0.153000	7.244790	0.701870
H	-2.329640	4.800140	1.847140
H	-2.364420	5.645230	0.307730
C	-2.765590	6.920030	2.017770
H	0.994500	6.209440	2.962260
H	-0.320210	5.132600	3.443060
C	-0.816740	7.243760	3.570550
H	-0.444720	7.180020	-0.353160
H	0.935390	7.392740	0.724270
C	-0.870880	8.435970	1.362490
H	-3.849570	6.757600	1.956930
C	-2.338590	7.020640	3.492590
C	-2.394020	8.221040	1.283650
H	-0.505650	7.308500	4.620930
C	-0.446490	8.544980	2.837800
H	-0.598410	9.355790	0.829420
H	-2.611140	6.099730	4.025530
H	-2.866280	7.848890	3.984470
H	-2.712790	8.163700	0.234130
H	-2.917260	9.074920	1.734590
H	-0.945030	9.402010	3.310120
H	0.635380	8.721460	2.907520
C	0.103110	-0.013210	-1.353160
C	1.307260	0.329430	-2.026420
C	1.601840	-0.183340	-3.322720
C	2.299670	1.110240	-1.373550
C	2.842800	-0.001300	-3.902780
H	0.854370	-0.751290	-3.861130
C	3.530280	1.317890	-1.967010
H	2.087010	1.532390	-0.401420
C	3.810440	0.728330	-3.202380
H	3.071520	-0.425840	-4.872320
H	4.281010	1.920770	-1.483580
Br	5.553520	0.902270	-3.911530
C	-0.861280	-0.879700	-2.072960
O	-1.871950	-0.483040	-2.608510
O	-0.471510	-2.180080	-2.020400
C	-1.300600	-3.148980	-2.653770
H	-1.314740	-4.026530	-2.010390

H	-2.311090	-2.764700	-2.787550
C	-0.734670	-3.541830	-4.025520
Cl	-1.648420	-4.984050	-4.575660
Cl	1.014920	-3.928170	-3.899220
Cl	-0.957390	-2.212830	-5.222240
H	4.658620	-3.268990	0.062190
H	3.794720	-4.234960	-1.196040
Cl	-1.954840	2.603080	-4.010190
Cl	0.147470	1.835400	-6.234520
Cl	3.092680	2.884070	-6.040380
Cl	4.032210	4.505290	-3.498790
Cl	5.057910	3.877140	0.392510
Cl	7.616140	3.135190	-1.294240
Cl	8.698390	0.193680	-1.298510
Cl	7.317570	-2.004490	0.493230
Cl	2.885180	-4.310380	3.726180
Cl	4.563180	-6.052420	1.698830
Cl	3.214970	-7.172340	-0.901160
Cl	0.164010	-6.602710	-1.475880
Cl	-4.577810	0.064250	-5.755720
Cl	-4.791750	-3.063760	-6.133230
Cl	-5.179300	-4.925990	-3.637750
Cl	-5.141880	-3.704600	-0.734380

Rh-2a·(HFIP)

6-31G(d)–SDD(Rh) SCF energy in gas phase (au):	-17371.841910
6-31G(d)–SDD(Rh) enthalpy in gas phase (au):	-17370.159260
6-31G(d)–SDD(Rh) free energy in gas phase (au):	-17370.429970
6-311+G(d,p)–SDD(Rh) SCF energy in implicit solvent (HFIP) (au):	-17376.923040
6-311+G(d,p)–SDD(Rh) enthalpy in implicit solvent (HFIP) (au):	-17375.240390
6-311+G(d,p)–SDD(Rh) free energy in implicit solvent (HFIP) (au):	-17375.505920

Cartesian coordinates

ATOM	X	Y	Z
Rh	0.480880	-0.848850	3.013520
Rh	-0.041290	-0.410060	0.644450
Cl	-1.822790	-4.197610	-2.715060
Cl	-3.767290	-3.684980	-5.151640
Cl	-7.497750	-1.628040	-1.749870
Cl	-6.648910	-2.563480	-4.651730
Cl	1.484230	-5.826570	-4.782130
Cl	3.856960	-4.558720	-6.438460
Cl	5.588080	-1.945250	-1.981420
Cl	5.968770	-2.732630	-5.014200
Cl	-5.963130	1.237790	1.254520
Cl	-7.549820	2.870530	-0.917650
Cl	-3.629950	6.661390	-0.946480
Cl	-6.387190	5.564610	-2.023290
Cl	5.782350	3.910060	-3.200610
Cl	5.020430	6.959300	-2.918740
Cl	2.732140	5.955990	1.924530
Cl	3.521750	7.977260	-0.363120
O	-1.951820	-1.029070	1.111670
O	-1.474620	-1.433880	3.282890
O	-6.073110	-1.667680	1.057000

O	-2.058360	-3.794360	0.375880
O	0.910370	-2.795220	2.501960
O	0.497810	-2.340820	0.324490
O	3.479720	-2.722500	0.223340
O	0.652340	-5.743830	-1.745930
O	-0.000610	1.117210	3.367190
O	-0.488040	1.540180	1.203810
O	-1.515780	5.592670	1.109010
O	-3.160740	1.580460	2.620240
O	1.923080	0.129420	0.406100
O	2.415880	-0.339330	2.570250
O	2.792510	2.801350	2.145620
O	5.202040	1.345900	-1.466900
N	-3.970990	-2.647080	1.049900
N	1.993920	-4.382910	-0.426050
N	-2.087430	3.580580	2.127520
N	4.151490	1.784190	0.556790
C	2.554240	-4.503610	-2.665040
C	2.640920	-4.793850	-4.016380
C	3.700960	-4.214800	-4.750670
C	4.634130	-3.369360	-4.117870
C	4.496030	-3.047260	-2.748360
C	3.450900	-3.628860	-2.048020
C	3.037390	-3.478810	-0.617990
C	-2.212830	-1.503340	2.265560
C	-3.119190	-3.268130	0.121990
C	1.219480	-4.513420	0.810290
H	0.266040	-4.945290	0.483740
C	0.862980	-3.097040	1.278340
C	-3.817530	-3.161240	-1.197810
C	-3.388250	-3.523260	-2.466740
C	-4.276980	-3.300960	-3.542800
C	-5.557720	-2.756600	-3.323810
C	-3.138810	2.661240	2.066100
C	-4.153320	3.262470	1.144250
C	-5.362520	2.756090	0.692490
C	-6.058470	3.488600	-0.295760
C	-5.533550	4.696240	-0.795830
C	-4.304630	5.197190	-0.312150
C	-3.640020	4.465860	0.658080
C	-2.295420	4.681840	1.278880
C	-5.957630	-2.364120	-2.026620
C	-5.067020	-2.573610	-0.984600
C	-0.792480	3.317520	2.767390
H	-0.079490	3.936450	2.212920
C	-0.400450	1.865190	2.447990
C	-5.167850	-2.219990	0.466720
C	-3.573340	-2.211680	2.390590
H	-4.299670	-1.429210	2.642760
C	1.587740	-4.983750	-1.627030
C	4.696440	2.125710	-0.684840
C	4.506770	3.603950	-0.819670
C	4.904230	4.473650	-1.823610
C	4.576350	5.840390	-1.677080
C	3.901210	6.298250	-0.528100

C	3.526810	5.397610	0.493330
C	3.822240	4.055860	0.311900
C	3.497580	2.865140	1.160870
C	4.159020	0.409580	1.066760
H	4.407830	-0.205180	0.197150
C	2.711750	0.033290	1.405460
C	-3.689210	-3.308870	3.487910
C	-2.536230	-4.337850	3.459510
C	-3.731830	-2.624860	4.878100
C	-5.025050	-4.066820	3.291760
H	-1.578440	-3.827580	3.586240
H	-2.505420	-4.836660	2.486160
C	-2.727130	-5.380060	4.575620
H	-4.564400	-1.906940	4.901950
H	-2.811230	-2.060930	5.043460
C	-3.916440	-3.674000	5.988360
H	-5.025870	-4.568950	2.316510
H	-5.860940	-3.355590	3.287000
C	-5.219220	-5.115960	4.401940
H	-1.893220	-6.092930	4.539610
C	-2.746360	-4.674710	5.943330
C	-4.056660	-6.124940	4.360860
H	-3.929780	-3.164380	6.960090
C	-5.244160	-4.420760	5.774470
H	-6.169550	-5.638320	4.233580
H	-1.795870	-4.149190	6.107260
H	-2.851790	-5.414000	6.748700
H	-4.043120	-6.646010	3.393940
H	-4.194580	-6.888790	5.137790
H	-5.395520	-5.163160	6.569450
H	-6.084960	-3.715990	5.825520
C	1.846160	-5.479950	1.844550
C	3.138620	-4.940760	2.499440
C	0.796700	-5.779640	2.944240
C	2.181140	-6.814520	1.132850
H	2.938540	-3.986670	2.993810
H	3.895590	-4.749290	1.730740
C	3.681850	-5.964440	3.513780
H	-0.117570	-6.171330	2.476780
H	0.525930	-4.857260	3.461230
C	1.349970	-6.799430	3.953350
H	2.927940	-6.638490	0.348430
H	1.284280	-7.208080	0.637670
C	2.733240	-7.841140	2.138410
H	4.591480	-5.558130	3.974530
C	2.624530	-6.227890	4.601980
C	4.011590	-7.281990	2.789360
H	0.591730	-6.981770	4.725990
C	1.681690	-8.114640	3.228370
H	2.965030	-8.771930	1.605340
H	2.389710	-5.294480	5.131110
H	3.017530	-6.933210	5.346420
H	4.779610	-7.107710	2.023460
H	4.423020	-8.012740	3.498460
H	2.063510	-8.856910	3.942000

H	0.772590	-8.535820	2.778450
C	5.250900	0.156240	2.136340
C	5.053910	0.959180	3.442310
C	5.292320	-1.359100	2.458750
C	6.622620	0.548110	1.530670
H	4.081950	0.724970	3.882130
H	5.058220	2.034020	3.225960
C	6.187100	0.638660	4.435240
H	5.443620	-1.924110	1.532210
H	4.333420	-1.680230	2.871830
C	6.422410	-1.664420	3.455440
H	6.633290	1.619240	1.290590
H	6.781790	0.007840	0.588370
C	7.761550	0.239300	2.519200
H	6.021020	1.209190	5.357890
C	6.188880	-0.868070	4.752010
C	7.539970	1.035470	3.818170
H	6.417990	-2.739830	3.675800
C	7.774010	-1.266150	2.838320
H	8.716650	0.530280	2.063630
H	5.229940	-1.157990	5.202130
H	6.974360	-1.098000	5.484540
H	7.555470	2.113590	3.607310
H	8.354830	0.833560	4.526190
H	8.593550	-1.496200	3.532290
H	7.953380	-1.844410	1.921900
C	-0.723760	3.762400	4.249310
C	-1.645150	2.945610	5.182620
C	0.743120	3.663840	4.740160
C	-1.148990	5.250900	4.330800
H	-1.386300	1.885280	5.134130
H	-2.686780	3.038270	4.851010
C	-1.516490	3.460220	6.629010
H	1.390220	4.252810	4.078060
H	1.091790	2.631330	4.683900
C	0.856180	4.174030	6.186490
H	-2.186600	5.360460	3.991500
H	-0.528600	5.853250	3.655780
C	-1.031620	5.771440	5.774300
H	-2.166320	2.859640	7.278040
C	-0.054870	3.328620	7.096220
C	-1.943030	4.937580	6.692820
H	1.898650	4.077000	6.515280
C	0.426240	5.649100	6.250330
H	-1.343190	6.823470	5.798200
H	0.256350	2.275910	7.063510
H	0.037920	3.662360	8.138500
H	-2.990790	5.040900	6.379050
H	-1.877200	5.307480	7.724770
H	0.521020	6.030910	7.275700
H	1.082790	6.259800	5.615630
C	-0.465040	-0.319840	-1.317980
C	-1.721760	-0.119090	-1.948060
C	-1.904300	-0.380610	-3.337210
C	-2.816830	0.418870	-1.217360

C	-3.096030	-0.086210	-3.970350
H	-1.094550	-0.819860	-3.907480
C	-4.010930	0.713570	-1.848040
H	-2.706980	0.608980	-0.160360
C	-4.134640	0.483350	-3.220790
H	-3.229620	-0.279100	-5.027980
H	-4.842280	1.127670	-1.296930
Br	-5.743000	0.973390	-4.080150
C	0.666660	-0.761790	-2.171830
O	0.838830	-1.913420	-2.518520
O	1.492740	0.265620	-2.454580
C	2.670890	-0.021760	-3.200730
H	3.534230	0.289760	-2.611130
H	2.722470	-1.085650	-3.428030
C	2.655920	0.763370	-4.512920
Cl	4.167450	0.343120	-5.383470
Cl	2.596500	2.523770	-4.208850
Cl	1.232060	0.274510	-5.505960
F	0.819510	4.437350	0.040130
F	1.445220	4.712330	-2.028400
F	1.272410	2.710050	-1.183510
F	-0.471760	2.271080	-3.335800
F	-2.313210	3.426980	-3.302130
F	-0.445270	4.370240	-3.913150
O	-1.497700	3.053740	-0.773530
H	-0.942090	2.532840	-0.146470
C	-0.759400	3.898490	-1.605470
H	-1.125590	4.927850	-1.509600
C	0.716360	3.934330	-1.207440
C	-0.989630	3.482310	-3.062850

Rh-2a•$(DCM)_2$

6-31G(d)-SDD(Rh) SCF energy in gas phase (au): -18501.438720
6-31G(d)-SDD(Rh) enthalpy in gas phase (au): -18499.758250
6-31G(d)-SDD(Rh) free energy in gas phase (au): -18500.030830
6-311+G(d,p)-SDD(Rh) SCF energy in implicit solvent (DCM) (au): -18506.404830
6-311+G(d,p)-SDD(Rh) enthalpy in implicit solvent (DCM) (au): -18504.724360
6-311+G(d,p)-SDD(Rh) free energy in implicit solvent (DCM) (au): -18504.996940

Cartesian coordinates

ATOM	X	Y	Z
Cl	2.966210	0.723770	-4.521800
Cl	3.105640	-0.375110	-1.761860
Cl	-3.999960	-0.670080	-3.565720
Cl	-3.401760	2.043340	-2.538300
C	3.608380	-0.573570	-3.459790
C	-3.864760	0.364920	-2.113540
Rh	0.006470	-0.150550	3.112450
Rh	-0.015330	-0.087620	0.643450
O	-2.014450	-0.534720	0.789540
O	-1.979870	-0.645330	3.051080
O	-5.817630	-1.395930	-0.396060
O	-2.177380	-3.497640	1.437930
O	0.496790	-2.149350	2.959870
O	0.548950	-2.056880	0.693970

O	3.621330	-2.316860	2.366120
O	1.997840	-5.054100	-0.936220
O	-0.424230	1.852940	3.090200
O	-0.544070	1.894840	0.827030
O	-1.342650	5.665960	-0.334900
O	-3.404610	2.357070	2.080990
O	1.985230	0.408940	0.787130
O	1.982270	0.382140	3.050360
O	2.239770	3.363510	1.181150
O	5.818080	0.917780	-0.334110
N	-4.063560	-2.283360	0.836410
N	2.593620	-3.883930	0.977830
N	-2.151630	4.090380	1.169470
N	4.086140	2.015370	0.759330
C	4.027590	-3.691250	-0.833390
C	4.631950	-3.719070	-2.080670
C	5.835020	-2.999330	-2.248220
C	6.435690	-2.333960	-1.159810
C	5.814050	-2.339090	0.108380
C	4.586760	-2.974400	0.225780
C	3.605370	-2.988420	1.357440
C	-2.535610	-0.767530	1.930070
C	-3.107380	-3.295460	0.686750
C	1.298420	-4.046070	1.665130
H	0.665310	-4.519630	0.910550
C	0.720330	-2.632570	1.822230
C	-3.484120	-4.027290	-0.562190
C	-2.879200	-5.104280	-1.187620
C	-3.434120	-5.562530	-2.402210
C	-4.572220	-4.937970	-2.950020
C	-3.278330	3.268430	1.289600
C	-4.229770	3.729620	0.231220
C	-5.491400	3.266030	-0.103090
C	-6.138700	3.850840	-1.213970
C	-5.502170	4.858080	-1.964990
C	-4.205000	5.299910	-1.620530
C	-3.598120	4.729180	-0.513270
C	-2.230650	4.939190	0.058330
C	-5.182800	-3.849400	-2.289440
C	-4.611010	-3.407390	-1.105970
C	-0.918630	3.928110	1.944870
H	-0.135450	4.355960	1.311250
C	-0.607870	2.426200	1.986060
C	-4.962440	-2.243160	-0.234410
C	-4.000040	-1.221930	1.846890
H	-4.538240	-0.381140	1.396040
C	2.758800	-4.316300	-0.343880
C	4.956670	1.768640	-0.313310
C	4.577510	2.751110	-1.376470
C	5.060990	2.915790	-2.663530
C	4.457070	3.899970	-3.476890
C	3.419740	4.709170	-2.970630
C	2.955160	4.538390	-1.647560
C	3.528410	3.532210	-0.887130
C	3.164650	3.023630	0.470650

C	3.970760	1.093430	1.891820
H	4.563170	0.223570	1.592890
C	2.517740	0.596810	1.933900
C	-4.722160	-1.571300	3.169710
C	-4.046620	-2.715100	3.960110
C	-4.795300	-0.298090	4.050420
C	-6.173230	-2.003290	2.839270
H	-3.011660	-2.451350	4.190050
H	-4.017730	-3.624210	3.347370
C	-4.829950	-2.991900	5.257060
H	-5.284160	0.507970	3.486930
H	-3.788640	0.048780	4.291600
C	-5.574700	-0.589350	5.344010
H	-6.159830	-2.905950	2.215430
H	-6.672270	-1.220330	2.254290
C	-6.961810	-2.290160	4.129340
H	-4.327990	-3.796140	5.810050
C	-4.868000	-1.716840	6.119860
C	-6.267330	-3.419910	4.911250
H	-5.599000	0.320970	5.956400
C	-7.009890	-1.020720	4.997500
H	-7.981340	-2.596180	3.862180
H	-3.846340	-1.409220	6.379910
H	-5.397080	-1.915620	7.061670
H	-6.251840	-4.339770	4.310770
H	-6.828740	-3.642620	5.828530
H	-7.582230	-1.213670	5.914780
H	-7.525080	-0.214270	4.458230
C	1.320890	-4.988210	2.884830
C	2.206770	-4.511920	4.057190
C	-0.135870	-5.167200	3.382780
C	1.840120	-6.367650	2.408660
H	1.877780	-3.526750	4.396000
H	3.244640	-4.405050	3.723320
C	2.145340	-5.531490	5.210430
H	-0.770020	-5.502840	2.553170
H	-0.536130	-4.206180	3.715950
C	-0.184920	-6.185920	4.533550
H	2.871110	-6.270090	2.044010
H	1.235110	-6.720700	1.562350
C	1.790640	-7.390470	3.557330
H	2.772960	-5.170750	6.035170
C	0.691950	-5.683130	5.694680
C	2.667910	-6.894320	4.721220
H	-1.223820	-6.288970	4.871910
C	0.337870	-7.547060	4.041680
H	2.166600	-8.355340	3.193690
H	0.314650	-4.717960	6.058130
H	0.646250	-6.387650	6.536150
H	3.712210	-6.800910	4.393140
H	2.651380	-7.624480	5.541430
H	0.289560	-8.288060	4.850800
H	-0.293960	-7.919150	3.223810
C	4.593790	1.629070	3.208230
C	3.785390	2.774530	3.857350

C	4.732340	0.462500	4.220030
C	6.015990	2.156090	2.891220
H	2.770810	2.434270	4.077660
H	3.695780	3.614240	3.160610
C	4.478980	3.249200	5.147210
H	5.311010	-0.350360	3.764820
H	3.747650	0.055610	4.455920
C	5.424440	0.948110	5.505280
H	5.953030	2.988480	2.179100
H	6.607550	1.367900	2.408310
C	6.714030	2.638980	4.175300
H	3.881700	4.055390	5.593080
C	4.589450	2.076780	6.138090
C	5.887160	3.771840	4.811060
H	5.499230	0.107010	6.206150
C	6.831510	1.470850	5.169750
H	7.714470	3.009850	3.918050
H	3.587960	1.704460	6.392070
H	5.057050	2.415050	7.072640
H	5.817440	4.621840	4.118540
H	6.384670	4.135800	5.719960
H	7.343170	1.803600	6.082790
H	7.438270	0.664930	4.734650
C	-0.908520	4.710650	3.278300
C	-1.953800	4.216950	4.303240
C	0.509540	4.617640	3.895450
C	-1.195630	6.202080	2.972060
H	-1.792680	3.158950	4.521970
H	-2.961890	4.308310	3.880500
C	-1.860870	5.053950	5.593230
H	1.248860	4.972750	3.166170
H	0.751720	3.576050	4.114050
C	0.589310	5.452580	5.183790
H	-2.196840	6.306060	2.534450
H	-0.481470	6.573370	2.226170
C	-1.113050	7.045470	4.257110
H	-2.600930	4.680890	6.312560
C	-0.447900	4.926520	6.193070
C	-2.149780	6.531320	5.272360
H	1.598240	5.356930	5.606260
C	0.297430	6.928070	4.861930
H	-1.324150	8.093570	4.009360
H	-0.236060	3.876310	6.434700
H	-0.384650	5.494770	7.130860
H	-3.163460	6.635740	4.861890
H	-2.109350	7.134320	6.189390
H	0.368140	7.538290	5.772330
H	1.044250	7.313460	4.154710
C	-0.153810	-0.181480	-1.374210
C	-0.172380	0.842560	-2.360230
C	-0.517640	0.554080	-3.713060
C	0.110150	2.194040	-2.016370
C	-0.599450	1.553040	-4.662850
H	-0.739270	-0.461740	-4.012430
C	0.005570	3.200250	-2.959590

H	0.373050	2.433670	-0.995450
C	-0.348460	2.872680	-4.273260
H	-0.873210	1.325730	-5.685810
H	0.168720	4.233540	-2.685950
Br	-0.515060	4.259190	-5.549500
C	-0.512090	-1.548000	-1.844280
O	-1.600000	-2.047790	-1.650900
O	0.504680	-2.158310	-2.501820
C	0.308590	-3.504970	-2.932210
H	1.233350	-4.037170	-2.729150
H	-0.509290	-3.970510	-2.385550
C	0.014000	-3.582570	-4.434900
Cl	0.167230	-5.307790	-4.906170
Cl	1.198980	-2.595730	-5.366860
Cl	-1.641540	-3.000290	-4.804970
H	4.693440	-0.528710	-3.505550
H	-3.097630	-0.053120	-1.464760
H	3.217120	-1.519830	-3.823280
H	-4.835680	0.386000	-1.626710
Cl	-6.233810	1.970960	0.774270
Cl	-7.710440	3.295910	-1.672760
Cl	-6.298910	5.543520	-3.337590
Cl	-3.387660	6.492610	-2.570210
Cl	1.699050	5.534790	-0.998890
Cl	2.690160	5.910330	-3.977600
Cl	4.990560	4.103130	-5.107720
Cl	6.341810	1.917810	-3.266550
Cl	6.590440	-1.636500	1.481710
Cl	7.938340	-1.514550	-1.372640
Cl	6.581750	-2.939930	-3.810480
Cl	3.914740	-4.570780	-3.409510
Cl	-6.602380	-3.102870	-2.931110
Cl	-5.227370	-5.503870	-4.447510
Cl	-2.710550	-6.903970	-3.218700
Cl	-1.457680	-5.846280	-0.527940

Rh-2a•(HFIP)$_2$

6-31G(d)–SDD(Rh) SCF energy in gas phase (au): -18161.667480
6-31G(d)–SDD(Rh) enthalpy in gas phase (au): -18159.909150
6-31G(d)–SDD(Rh) free energy in gas phase (au): -18160.199830
6-311+G(d,p)–SDD(Rh) SCF energy in implicit solvent (HFIP) (au): -18167.020070
6-311+G(d,p)–SDD(Rh) enthalpy in implicit solvent (HFIP) (au): -18165.261740
6-311+G(d,p)–SDD(Rh) free energy in implicit solvent (HFIP) (au): -18165.547240

Cartesian coordinates

ATOM	X	Y	Z
Rh	-0.598910	0.241600	2.907150
Rh	0.125730	0.346960	0.550700
Cl	3.032120	4.197160	-1.711060
Cl	5.205230	3.852650	-3.973210
Cl	8.022240	0.464100	-0.771680
Cl	7.786590	2.163010	-3.424380
Cl	1.096600	7.208100	-2.994180
Cl	-0.928860	7.031020	-5.411260
Cl	-4.312520	4.031490	-2.365460

Cl	-3.644930	5.501930	-5.078860
Cl	6.155120	-2.540890	0.496100
Cl	7.322030	-4.030720	-2.033160
Cl	2.566860	-6.620330	-2.699030
Cl	5.518470	-6.025130	-3.650160
Cl	-6.212350	-0.501960	-4.817710
Cl	-6.355680	-3.541450	-5.656880
Cl	-4.487550	-4.994530	-0.749210
Cl	-5.494040	-5.773510	-3.633460
O	2.032820	0.496550	1.294980
O	1.387320	0.362710	3.455300
O	6.202220	0.152780	1.793220
O	2.796890	3.190890	1.247750
O	-0.653920	2.295850	2.919230
O	-0.081010	2.361760	0.732040
O	-2.744450	3.604990	0.318330
O	1.068310	6.127960	-0.036300
O	-0.362380	-1.798330	2.795900
O	0.369470	-1.727780	0.665760
O	0.819480	-5.647250	-0.252950
O	3.274990	-2.305050	1.714840
O	-1.864240	0.279470	0.031420
O	-2.527150	0.272570	2.198240
O	-3.828470	-2.251090	0.664310
O	-5.137030	1.005900	-2.277170
N	4.338450	1.532930	1.811660
N	-0.810180	4.873710	0.504380
N	1.817240	-3.974570	1.013370
N	-4.499990	-0.391160	-0.533560
C	-0.744790	5.741750	-1.636550
C	-0.404600	6.364190	-2.826250
C	-1.313830	6.273880	-3.903720
C	-2.529390	5.576160	-3.758590
C	-2.846900	4.931480	-2.541820
C	-1.933400	5.021860	-1.504020
C	-1.944660	4.393150	-0.146170
C	2.240910	0.562400	2.549110
C	3.756830	2.503750	0.978400
C	-0.344110	4.431240	1.817500
H	0.727010	4.669200	1.809880
C	-0.389050	2.898630	1.842580
C	4.598620	2.521770	-0.260690
C	4.418730	3.207410	-1.453300
C	5.405560	3.050590	-2.452920
C	6.541570	2.248940	-2.227270
C	3.022320	-3.273950	1.025160
C	3.876480	-3.919920	-0.021040
C	5.189250	-3.667060	-0.389990
C	5.698950	-4.338720	-1.522750
C	4.891220	-5.241930	-2.242490
C	3.570980	-5.516590	-1.821060
C	3.095300	-4.855950	-0.700510
C	1.765380	-4.926580	-0.019660
C	6.674810	1.518430	-1.025010
C	5.687380	1.668160	-0.062750

C	0.693700	-3.761050	1.921140
H	-0.104110	-4.384230	1.497880
C	0.197760	-2.311210	1.800120
C	5.510260	0.997820	1.265110
C	3.681220	0.905480	2.960250
H	4.199270	-0.055410	3.068200
C	-0.000120	5.647810	-0.342030
C	-4.977000	-0.106720	-1.821000
C	-5.209390	-1.432030	-2.471340
C	-5.704470	-1.740680	-3.728000
C	-5.778450	-3.106330	-4.086730
C	-5.391250	-4.109800	-3.176840
C	-4.917610	-3.773430	-1.888000
C	-4.820660	-2.427510	-1.570390
C	-4.320640	-1.753710	-0.334410
C	-4.154640	0.651310	0.440190
H	-4.037090	1.560830	-0.157200
C	-2.736700	0.358270	0.960160
C	3.845970	1.671510	4.304640
C	2.903420	2.889230	4.437340
C	3.585170	0.690100	5.476020
C	5.307240	2.169120	4.420310
H	1.862900	2.566260	4.351410
H	3.088630	3.593510	3.620000
C	3.123830	3.585330	5.792280
H	4.269650	-0.166060	5.388260
H	2.566910	0.300040	5.415140
C	3.800930	1.395330	6.826230
H	5.523950	2.871810	3.606410
H	5.998910	1.324570	4.308230
C	5.532540	2.872670	5.771370
H	2.439340	4.439710	5.867650
C	2.838760	2.593220	6.933980
C	4.579290	4.076360	5.886490
H	3.596760	0.683200	7.635870
C	5.254700	1.888980	6.921690
H	6.573060	3.217690	5.821970
H	1.798120	2.246130	6.877320
H	2.962840	3.089620	7.905780
H	4.784440	4.798350	5.084530
H	4.744100	4.596360	6.839650
H	5.426480	2.381850	7.887910
H	5.945820	1.037260	6.865450
C	-0.981180	5.193980	3.007680
C	-2.449850	4.802350	3.283970
C	-0.131890	4.935840	4.277370
C	-0.928860	6.712700	2.706690
H	-2.518530	3.730730	3.485700
H	-3.062840	5.000060	2.398200
C	-2.994450	5.602660	4.481230
H	0.908950	5.228110	4.080780
H	-0.130200	3.870170	4.514380
C	-0.685010	5.734860	5.468920
H	-1.525980	6.933820	1.813290
H	0.103040	7.013290	2.484590

C	-1.479130	7.519830	3.896620
H	-4.033910	5.301190	4.665070
C	-2.142400	5.310300	5.730010
C	-2.938780	7.107390	4.162330
H	-0.072920	5.520600	6.354660
C	-0.631640	7.238330	5.149850
H	-1.434690	8.588600	3.651610
H	-2.183920	4.239280	5.970140
H	-2.543750	5.853070	6.596260
H	-3.559020	7.328270	3.282980
H	-3.347150	7.688820	4.999710
H	-1.010530	7.821750	5.999580
H	0.407770	7.550310	4.980610
C	-5.275710	0.923880	1.476400
C	-5.575330	-0.275690	2.397080
C	-4.879670	2.152040	2.334390
C	-6.575150	1.283550	0.712430
H	-4.671540	-0.573010	2.929550
H	-5.902360	-1.135180	1.803160
C	-6.691270	0.081520	3.394180
H	-4.679920	3.005670	1.678230
H	-3.957130	1.942780	2.878870
C	-6.000820	2.496960	3.328180
H	-6.893810	0.434500	0.093530
H	-6.388630	2.124330	0.032270
C	-7.703050	1.635990	1.700140
H	-6.869130	-0.786450	4.039090
C	-6.258690	1.288210	4.246270
C	-7.974230	0.431470	2.620130
H	-5.684460	3.359470	3.929720
C	-7.283150	2.845620	2.553970
H	-8.608930	1.880390	1.130720
H	-5.347310	1.041950	4.807520
H	-7.038010	1.530410	4.981510
H	-8.300910	-0.432550	2.025510
H	-8.788180	0.668830	3.318270
H	-8.089860	3.111660	3.250140
H	-7.108020	3.719030	1.911340
C	0.960260	-4.291030	3.369920
C	1.803710	-3.348160	4.262190
C	-0.404470	-4.552250	4.046630
C	1.695810	-5.651320	3.269250
H	1.319040	-2.372480	4.344900
H	2.786190	-3.179900	3.813560
C	1.979830	-3.966170	5.662470
H	-0.976620	-5.247860	3.426370
H	-0.972310	-3.624410	4.110910
C	-0.220680	-5.155090	5.447210
H	2.681210	-5.516140	2.806270
H	1.127290	-6.335250	2.624580
C	1.883780	-6.270690	4.667230
H	2.573140	-3.276300	6.276320
C	0.603170	-4.187310	6.314090
C	2.715040	-5.312900	5.540170
H	-1.210960	-5.307240	5.891330

C	0.512720	-6.500910	5.326450
H	2.410700	-7.227470	4.560450
H	0.075090	-3.229110	6.410930
H	0.725380	-4.594870	7.326680
H	3.707350	-5.159950	5.094290
H	2.871810	-5.752760	6.534070
H	0.644960	-6.955870	6.317160
H	-0.084610	-7.199970	4.725850
C	0.722490	0.667950	-1.349310
C	1.970080	0.379730	-1.964200
C	2.275940	0.851140	-3.274660
C	2.930180	-0.433630	-1.305220
C	3.449600	0.495560	-3.910030
H	1.575890	1.497950	-3.788680
C	4.113560	-0.775500	-1.930950
H	2.733620	-0.781100	-0.302350
C	4.353020	-0.336140	-3.235030
H	3.671770	0.844220	-4.911200
H	4.850780	-1.380310	-1.427110
Br	5.932920	-0.902370	-4.100650
C	-0.205360	1.522560	-2.133000
O	-0.121200	2.734480	-2.157400
O	-1.136710	0.799560	-2.784510
C	-2.081920	1.516600	-3.575370
H	-3.066090	1.432260	-3.114710
H	-1.793470	2.564510	-3.652790
C	-2.130660	0.908290	-4.976340
Cl	-3.363900	1.826000	-5.899610
Cl	-2.577370	-0.824140	-4.908670
Cl	-0.526210	1.081370	-5.779020
F	-1.440690	-3.713660	-0.852420
F	-2.111610	-3.723730	-2.928930
F	-1.537710	-1.856550	-1.967080
F	0.419140	-1.533470	-3.961980
F	1.796650	-3.196920	-4.212820
F	-0.281010	-3.400790	-4.838950
O	1.134580	-2.853490	-1.626530
H	0.717220	-2.462720	-0.821650
C	0.204860	-3.413750	-2.506010
H	0.317840	-4.504690	-2.549070
C	-1.239620	-3.163660	-2.066980
C	0.526580	-2.872740	-3.902390
F	-2.946490	-4.904940	2.090170
F	-4.623140	-6.281320	2.216510
F	-3.273310	-6.074900	3.908380
F	-3.155550	-2.555030	3.603090
F	-4.922730	-2.388600	4.860250
F	-3.386980	-3.849770	5.344200
O	-5.465630	-3.654560	2.408510
H	-4.805000	-3.237040	1.814640
C	-4.797530	-4.281840	3.455940
H	-5.544060	-4.756900	4.099100
C	-3.891230	-5.399710	2.920230
C	-4.046390	-3.259530	4.326530

Rh-2a•(HFIP)$_3$

6-31G(d)-SDD(Rh) SCF energy in gas phase (au):	-18951.496630
6-31G(d)-SDD(Rh) enthalpy in gas phase (au):	-18949.662490
6-31G(d)-SDD(Rh) free energy in gas phase (au):	-18949.972870
6-311+G(d,p)-SDD(Rh) SCF energy in implicit solvent (HFIP) (au):	-18957.120570
6-311+G(d,p)-SDD(Rh) enthalpy in implicit solvent (HFIP) (au):	-18955.286440
6-311+G(d,p)-SDD(Rh) free energy in implicit solvent (HFIP) (au):	-18955.591640

Cartesian coordinates

ATOM	X	Y	Z
Rh	-0.693540	-0.662900	2.901160
Rh	-0.855680	0.250460	0.612120
Cl	-5.297320	2.624820	-1.297220
Cl	-4.343510	4.152620	-3.890050
Cl	-1.136350	7.325380	-0.831870
Cl	-2.349830	6.558070	-3.638960
Cl	-8.010650	0.510020	-2.902030
Cl	-7.702820	-1.377600	-5.414790
Cl	-4.077380	-4.412880	-2.702930
Cl	-5.853630	-3.903700	-5.265020
Cl	1.650060	6.484860	1.485070
Cl	3.092070	8.182200	-0.751380
Cl	5.983050	3.799210	-2.214590
Cl	5.195230	6.825960	-2.637270
Cl	0.779110	-4.202360	-5.444550
Cl	3.855160	-4.467030	-6.116590
Cl	4.984080	-4.771060	-0.788760
Cl	5.949960	-4.771290	-3.801730
O	-1.243460	2.051540	1.519860
O	-1.102280	1.232260	3.620430
O	-1.228720	5.966180	2.017700
O	-4.156420	2.450340	1.612860
O	-2.724890	-0.987780	2.840240
O	-2.841110	-0.169400	0.735160
O	-3.703360	-2.889960	0.024200
O	-6.855430	0.441150	0.029960
O	1.305400	-0.168730	2.848130
O	1.191000	0.663610	0.751460
O	5.118850	1.615690	-0.184480
O	1.610800	3.405220	2.184410
O	-0.532090	-1.666490	-0.062240
O	-0.451780	-2.517540	2.037720
O	2.261750	-3.915650	0.574440
O	-0.869490	-4.208660	-2.751430
N	-2.617900	4.114820	2.154840
N	-5.255900	-1.204960	0.386070
N	3.359890	2.211100	1.224410
N	0.439340	-4.163950	-0.834400
C	-6.212760	-1.147420	-1.717910
C	-6.950460	-0.855370	-2.853570
C	-6.804740	-1.704850	-3.973630
C	-5.947210	-2.821640	-3.919510
C	-5.172560	-3.076330	-2.764750
C	-5.316480	-2.217280	-1.685960
C	-4.629750	-2.202750	-0.356330

C	-1.380820	2.132760	2.786200
C	-3.518190	3.435090	1.313780
C	-4.876690	-0.794690	1.738920
H	-5.235120	0.239050	1.813350
C	-3.347170	-0.667710	1.789300
C	-3.478560	4.157840	0.003640
C	-4.095500	3.849280	-1.201690
C	-3.706250	4.588300	-2.341360
C	-2.799820	5.660530	-2.230210
C	2.581050	3.357170	1.458670
C	3.170610	4.428460	0.595080
C	2.848770	5.772240	0.475210
C	3.498010	6.516880	-0.535900
C	4.448680	5.908310	-1.379170
C	4.798370	4.549390	-1.207550
C	4.147040	3.839990	-0.210090
C	4.301190	2.425220	0.222850
C	-2.244910	6.004760	-0.978170
C	-2.577640	5.219220	0.115730
C	3.236520	0.952770	1.966850
H	3.814910	0.224580	1.391130
C	1.786740	0.450130	1.871990
C	-2.033180	5.215440	1.509190
C	-1.923360	3.484760	3.285840
H	-1.041940	4.121090	3.434140
C	-6.194940	-0.490530	-0.372740
C	0.220370	-4.205650	-2.220040
C	1.573900	-4.278120	-2.848770
C	1.952740	-4.321930	-4.180960
C	3.330660	-4.447550	-4.470130
C	4.275540	-4.568870	-3.430810
C	3.864290	-4.523130	-2.080990
C	2.517280	-4.336490	-1.820570
C	1.795290	-4.129960	-0.529180
C	-0.661110	-4.006310	0.125300
H	-1.544100	-3.888870	-0.509940
C	-0.517720	-2.624210	0.785230
C	-2.699350	3.475870	4.630250
C	-3.787860	2.383090	4.721360
C	-1.683840	3.278510	5.785220
C	-3.371570	4.857050	4.816090
H	-3.336590	1.396880	4.583970
H	-4.516320	2.516400	3.917220
C	-4.494520	2.451020	6.086750
H	-0.916230	4.064040	5.730340
H	-1.175220	2.317940	5.672460
C	-2.397790	3.342520	7.146630
H	-4.098900	5.023530	4.012550
H	-2.617660	5.651880	4.734940
C	-4.084970	4.929030	6.178840
H	-5.258100	1.663590	6.129110
C	-3.468080	2.237000	7.212730
C	-5.160700	3.829060	6.250290
H	-1.658700	3.190310	7.943350
C	-3.064630	4.719160	7.311700

H	-4.555770	5.914930	6.281950
H	-2.996670	1.250440	7.107800
H	-3.968540	2.255380	8.190170
H	-5.908260	3.980530	5.460040
H	-5.689890	3.883750	7.211040
H	-3.566550	4.783700	8.286320
H	-2.304060	5.511180	7.286530
C	-5.569140	-1.613170	2.860690
C	-5.022110	-3.050330	3.003920
C	-5.403250	-0.860550	4.204870
C	-7.085620	-1.698290	2.554330
H	-3.949660	-3.022220	3.207590
H	-5.155450	-3.596810	2.063350
C	-5.756740	-3.786400	4.139190
H	-5.808050	0.155680	4.101990
H	-4.344060	-0.764450	4.450610
C	-6.134830	-1.605250	5.334070
H	-7.243480	-2.232900	1.609210
H	-7.497300	-0.690080	2.424350
C	-7.826460	-2.439790	3.681890
H	-5.344220	-4.799570	4.229750
C	-5.554280	-3.026170	5.462840
C	-7.259050	-3.864790	3.815520
H	-5.986110	-1.058150	6.274070
C	-7.635900	-1.686220	5.009900
H	-8.894140	-2.487500	3.432840
H	-4.484290	-2.974080	5.705390
H	-6.047820	-3.561990	6.284630
H	-7.413650	-4.421720	2.881280
H	-7.790690	-4.409020	4.607470
H	-8.172630	-2.203010	5.816650
H	-8.059440	-0.675690	4.934480
C	-0.921490	-5.252280	1.013080
C	0.226750	-5.624800	1.972130
C	-2.211020	-5.011400	1.837180
C	-1.179030	-6.457310	0.073570
H	0.458410	-4.781350	2.624190
H	1.130530	-5.857270	1.405200
C	-0.144100	-6.866290	2.801970
H	-3.032880	-4.757470	1.162730
H	-2.067320	-4.158940	2.504680
C	-2.572420	-6.259430	2.658140
H	-0.283770	-6.660070	-0.529400
H	-1.990660	-6.216090	-0.626270
C	-1.540660	-7.711830	0.891230
H	0.692180	-7.090570	3.473960
C	-1.417660	-6.591460	3.618940
C	-0.386190	-8.052770	1.851930
H	-3.485120	-6.050050	3.231310
C	-2.817950	-7.443430	1.707200
H	-1.709950	-8.548680	0.201490
H	-1.248010	-5.751690	4.306170
H	-1.671850	-7.467040	4.231640
H	0.528260	-8.269630	1.283160
H	-0.631540	-8.956940	2.425010

H	-3.094070	-8.340510	2.277420
H	-3.655650	-7.216470	1.033840
C	3.892950	1.009530	3.381940
C	3.059500	1.758870	4.447280
C	4.155530	-0.442190	3.849160
C	5.269630	1.712750	3.259370
H	2.073100	1.298540	4.545670
H	2.899810	2.798150	4.143500
C	3.793670	1.733070	5.801850
H	4.772460	-0.945730	3.100270
H	3.214680	-0.989220	3.922810
C	4.876760	-0.454150	5.206730
H	5.134680	2.753940	2.938140
H	5.882100	1.214790	2.498860
C	6.005570	1.702310	4.612370
H	3.179380	2.259770	6.543310
C	4.012970	0.276740	6.248830
C	5.154170	2.439310	5.661420
H	5.026710	-1.494520	5.510600
C	6.235910	0.250720	5.066630
H	6.969810	2.212080	4.490670
H	3.045980	-0.232630	6.357880
H	4.504040	0.254640	7.231050
H	5.005380	3.485940	5.361540
H	5.677020	2.451740	6.626940
H	6.772640	0.239770	6.024770
H	6.858660	-0.280040	4.337010
C	-1.144360	0.983160	-1.242030
C	-0.670650	2.207540	-1.787400
C	-0.737380	2.462660	-3.187580
C	-0.031380	3.172110	-0.962270
C	-0.122480	3.568450	-3.744920
H	-1.231540	1.757660	-3.842080
C	0.528740	4.307490	-1.512410
H	-0.000010	3.017980	0.106810
C	0.525770	4.475450	-2.899750
H	-0.132730	3.728210	-4.816010
H	0.979490	5.058060	-0.884440
Br	1.434360	5.961000	-3.635150
C	-2.031670	0.155150	-2.097900
O	-3.240020	0.189990	-2.012140
O	-1.334110	-0.634680	-2.942940
C	-2.066270	-1.514400	-3.792940
H	-1.556840	-2.476000	-3.771730
H	-3.091790	-1.617320	-3.443000
C	-2.084330	-0.985030	-5.230020
Cl	-2.800460	-2.275200	-6.250280
Cl	-0.423260	-0.605970	-5.781610
Cl	-3.103610	0.499690	-5.348830
F	2.760370	-1.317970	-1.101680
F	3.339310	-1.593130	-3.192070
F	1.329200	-0.941050	-2.684730
F	1.857190	1.129020	-4.345130
F	3.373710	2.549680	-3.711150
F	3.964610	0.644360	-4.590170

O	2.366020	1.459180	-1.549440
H	2.025870	0.998540	-0.746870
C	3.123110	0.625900	-2.375540
H	4.179370	0.591410	-2.077670
C	2.623680	-0.821230	-2.352010
C	3.074570	1.239750	-3.778130
F	7.069700	-0.124310	-1.777720
F	5.858060	-1.857290	-2.275720
F	7.981920	-2.107180	-1.865830
F	7.680180	0.203390	0.935300
F	7.310790	-1.535850	2.190810
F	8.822270	-1.629410	0.626600
O	5.302010	-1.035410	0.255560
H	5.376190	-0.067520	0.101480
C	6.523410	-1.642130	-0.027540
H	6.416720	-2.713920	0.137410
C	6.879650	-1.433110	-1.503700
C	7.607990	-1.144430	0.934080
F	4.747680	-3.065140	2.059370
F	6.202860	-4.668320	2.198880
F	5.877310	-3.359190	3.906450
F	2.408160	-3.339300	3.488780
F	2.198500	-5.162940	4.655440
F	3.607670	-3.630570	5.287140
O	3.612570	-5.634350	2.293120
H	3.196870	-4.982930	1.688680
C	4.168830	-4.954730	3.373240
H	4.647390	-5.689640	4.026960
C	5.262190	-3.990550	2.891070
C	3.084330	-4.252970	4.211240

Rh-2a•(DCM)$_4$

6-31G(d)-SDD(Rh) SCF energy in gas phase (au): -20420.858780
6-31G(d)-SDD(Rh) enthalpy in gas phase (au): -20419.105310
6-31G(d)-SDD(Rh) free energy in gas phase (au): -20419.399290
6-311+G(d,p)-SDD(Rh) SCF energy in implicit solvent (DCM) (au): -20425.992310
6-311+G(d,p)-SDD(Rh) enthalpy in implicit solvent (DCM) (au): -20424.238840
6-311+G(d,p)-SDD(Rh) free energy in implicit solvent (DCM) (au): -20424.532830

Cartesian coordinates

ATOM	X	Y	Z
Cl	-7.014800	-0.495400	-4.734410
Cl	-4.385670	-1.077420	-3.440820
Cl	3.392260	0.032670	-3.941240
Cl	2.972050	-0.742250	-1.101550
Cl	4.605920	-3.024350	-5.541040
Cl	3.546320	-5.821980	-5.626020
Cl	-4.778680	1.660230	-0.605450
Cl	-3.078180	2.318570	-2.947860
C	-5.916320	-0.143450	-3.365260
C	3.626370	-1.230070	-2.686990
C	4.991560	-4.775940	-5.522860
C	-3.166850	1.485270	-1.355370
Rh	-0.485170	0.468960	3.355880
Rh	-0.303280	0.216330	0.909190

O	-2.300370	-0.264870	0.950740
O	-2.478470	-0.010830	3.188970
O	-6.132180	-1.392810	-0.294760
O	-2.376150	-3.139580	1.677100
O	-0.095140	-1.556100	3.490990
O	0.232800	-1.726500	1.253280
O	3.064680	-2.106280	3.275500
O	1.478010	-4.949390	0.053440
O	-0.771400	2.486120	3.047170
O	-0.801130	2.225500	0.798350
O	-0.173540	5.481190	-1.029080
O	-3.588780	4.032850	1.665910
O	1.693790	0.695160	1.136190
O	1.528190	0.880570	3.385530
O	2.583580	3.514020	1.052550
O	5.699360	0.188070	0.493930
N	-4.294050	-2.046700	0.967350
N	2.016270	-3.698550	1.936590
N	-1.667680	4.806970	0.612640
N	4.112240	1.764330	1.119730
C	3.658440	-3.869510	0.310140
C	4.395910	-4.103550	-0.839800
C	5.706210	-3.580300	-0.897100
C	6.266030	-2.902030	0.204180
C	5.499810	-2.687320	1.370560
C	4.187280	-3.132270	1.370940
C	3.084350	-2.893210	2.353110
C	-2.913440	-0.367670	2.061360
C	-3.324920	-3.048640	0.926180
C	0.657540	-3.653960	2.504850
H	0.041860	-4.148560	1.750080
C	0.215100	-2.184070	2.447070
C	-3.719050	-3.956940	-0.193910
C	-3.156410	-5.148930	-0.622480
C	-3.766680	-5.817090	-1.706780
C	-4.915630	-5.280620	-2.321170
C	-3.044660	4.566440	0.724370
C	-3.638310	5.039210	-0.568370
C	-4.959570	5.098220	-0.985180
C	-5.211360	5.471140	-2.324060
C	-4.149220	5.787800	-3.194480
C	-2.814320	5.761890	-2.737270
C	-2.588810	5.393850	-1.420620
C	-1.308820	5.258470	-0.661980
C	-5.488410	-4.077380	-1.850990
C	-4.865000	-3.431170	-0.794160
C	-0.663750	4.436260	1.607940
H	0.284180	4.521890	1.063200
C	-0.782350	2.928310	1.871110
C	-5.225440	-2.167640	-0.073520
C	-4.329980	-0.960800	1.950880
H	-4.940240	-0.185900	1.474340
C	2.263100	-4.267970	0.679720
C	5.049910	1.182610	0.255500
C	5.061120	2.038360	-0.968620

C	5.760110	1.896320	-2.155220
C	5.547220	2.857500	-3.167370
C	4.681980	3.947770	-2.946250
C	3.992190	4.082990	-1.720000
C	4.168640	3.093610	-0.765410
C	3.498450	2.885650	0.558250
C	3.692280	1.106190	2.356630
H	4.104670	0.094730	2.275680
C	2.170360	0.901220	2.303690
C	-5.033210	-1.359310	3.279310
C	-4.206980	-2.334930	4.149680
C	-5.341400	-0.073440	4.088150
C	-6.385170	-2.040200	2.946570
H	-3.236260	-1.894690	4.387870
H	-4.011800	-3.258420	3.594220
C	-4.969930	-2.668620	5.444230
H	-5.948430	0.604560	3.471320
H	-4.413280	0.449280	4.328450
C	-6.098600	-0.421340	5.381940
H	-6.208060	-2.962710	2.379270
H	-6.988760	-1.382950	2.308620
C	-7.152420	-2.386710	4.235670
H	-4.357090	-3.349990	6.048940
C	-5.239390	-1.374770	6.233720
C	-6.306550	-3.345380	5.093240
H	-6.288260	0.503530	5.941000
C	-7.433290	-1.100750	5.032090
H	-8.099780	-2.869560	3.964260
H	-4.289280	-0.890310	6.495940
H	-5.755750	-1.607590	7.174670
H	-6.122130	-4.279560	4.545180
H	-6.851390	-3.610640	6.009100
H	-7.990740	-1.339360	5.947710
H	-8.059080	-0.419250	4.440420
C	0.479430	-4.445940	3.819150
C	1.288130	-3.908910	5.021180
C	-1.027190	-4.451840	4.181330
C	0.912410	-5.909180	3.552960
H	1.020120	-2.867350	5.214370
H	2.358800	-3.925960	4.792570
C	1.023610	-4.777620	6.265750
H	-1.605240	-4.834300	3.332870
H	-1.368330	-3.430010	4.362010
C	-1.279740	-5.318510	5.425130
H	1.978060	-5.940030	3.290510
H	0.359270	-6.309130	2.692090
C	0.659880	-6.782780	4.795020
H	1.601420	-4.374220	7.106960
C	-0.476360	-4.754720	6.610190
C	1.464040	-6.225540	5.983850
H	-2.352100	-5.295790	5.659840
C	-0.840310	-6.765400	5.140400
H	0.978920	-7.810590	4.579730
H	-0.797100	-3.726420	6.824440
H	-0.665550	-5.349260	7.514170

H	2.538970	-6.254330	5.758820
H	1.303460	-6.851580	6.871760
H	-1.031390	-7.401120	6.015260
H	-1.423890	-7.178040	4.306320
C	4.306310	1.727640	3.642770
C	3.671710	3.076240	4.043560
C	4.155980	0.724580	4.815680
C	5.820140	1.955100	3.400360
H	2.600810	2.943700	4.212270
H	3.780720	3.801650	3.230450
C	4.343440	3.621980	5.317310
H	4.606670	-0.234670	4.536970
H	3.098630	0.532470	5.005930
C	4.827280	1.278190	6.083710
H	5.961550	2.671280	2.581150
H	6.294790	1.016510	3.090420
C	6.496660	2.503380	4.669090
H	3.871190	4.575820	5.585150
C	4.166870	2.615210	6.467890
C	5.843740	3.843130	5.055540
H	4.696200	0.554250	6.897950
C	6.326650	1.498190	5.821260
H	7.563950	2.656510	4.464010
H	3.098670	2.460840	6.670940
H	4.618600	3.010760	7.387610
H	5.981280	4.575910	4.248630
H	6.328700	4.254100	5.951040
H	6.822020	1.874950	6.726140
H	6.807060	0.544390	5.563170
C	-0.556380	5.404630	2.811440
C	-1.749340	5.367030	3.791890
C	0.746270	5.073770	3.581670
C	-0.424300	6.843870	2.252590
H	-1.875290	4.354750	4.185550
H	-2.676290	5.622830	3.269850
C	-1.516860	6.367190	4.940960
H	1.595930	5.095970	2.889060
H	0.686080	4.060590	3.984460
C	0.968710	6.075070	4.726210
H	-1.334660	7.113980	1.702240
H	0.408150	6.889520	1.537740
C	-0.201730	7.850430	3.395450
H	-2.370660	6.319850	5.628610
C	-0.224350	6.007880	5.696050
C	-1.394840	7.790520	4.366730
H	1.892410	5.808350	5.255740
C	1.091690	7.496060	4.150710
H	-0.118800	8.859090	2.970830
H	-0.305370	4.998220	6.120700
H	-0.071230	6.701430	6.533930
H	-2.320870	8.065370	3.843630
H	-1.255070	8.516880	5.178560
H	1.266530	8.220260	4.957610
H	1.953050	7.553130	3.471480
C	-0.246320	-0.084330	-1.086490

C	0.050150	0.827110	-2.134060
C	-0.158950	0.474110	-3.499110
C	0.541570	2.131470	-1.845660
C	0.106350	1.367000	-4.518170
H	-0.538830	-0.507240	-3.754070
C	0.788720	3.035290	-2.862160
H	0.709520	2.418560	-0.817260
C	0.580280	2.641970	-4.188870
H	-0.050310	1.091120	-5.553860
H	1.116790	4.039450	-2.636300
Br	0.961990	3.868310	-5.577440
C	-0.753320	-1.422980	-1.488320
O	-1.923100	-1.737700	-1.413790
O	0.232320	-2.238420	-1.933360
C	-0.121440	-3.583940	-2.254400
H	0.679470	-4.216160	-1.876050
H	-1.064140	-3.851980	-1.782870
C	-0.251950	-3.787280	-3.768040
Cl	-0.421960	-5.556290	-4.029300
Cl	1.205270	-3.180940	-4.619050
Cl	-1.702270	-2.951540	-4.423680
H	-6.409900	-0.418880	-2.437020
H	4.695290	-1.394930	-2.585410
H	5.493750	-4.990550	-4.583560
H	-2.984400	0.427100	-1.512950
H	-5.657850	0.911180	-3.397870
H	3.101400	-2.120980	-3.015930
H	5.624580	-4.985330	-6.381850
H	-2.431930	1.934230	-0.694610
Cl	-6.257450	4.755720	0.102270
Cl	-6.840920	5.522710	-2.900690
Cl	-4.477570	6.204260	-4.840840
Cl	-1.497980	6.113820	-3.802240
Cl	2.968690	5.440010	-1.412620
Cl	4.451020	5.131630	-4.184500
Cl	6.352220	2.688120	-4.686640
Cl	6.834080	0.559050	-2.401020
Cl	6.193350	-1.954480	2.771090
Cl	7.892070	-2.332140	0.133840
Cl	6.646420	-3.773040	-2.342390
Cl	3.713050	-4.961430	-2.182740
Cl	-6.931840	-3.450750	-2.562140
Cl	-5.644500	-6.108080	-3.652810
Cl	-3.113540	-7.319480	-2.260990
Cl	-1.742480	-5.801160	0.139100

Rh-2a•(HFIP)$_4$

6-31G(d)-SDD(Rh) SCF energy in gas phase (au):	-19741.320060
6-31G(d)-SDD(Rh) enthalpy in gas phase (au):	-19739.410860
6-31G(d)-SDD(Rh) free energy in gas phase (au):	-19739.742800
6-311+G(d,p)-SDD(Rh) SCF energy in implicit solvent (HFIP) (au):	-19747.221560
6-311+G(d,p)-SDD(Rh) enthalpy in implicit solvent (HFIP) (au):	-19745.312360
6-311+G(d,p)-SDD(Rh) free energy in implicit solvent (HFIP) (au):	-19745.639130

Cartesian coordinates

ATOM	X	Y	Z
Rh	-0.316880	-0.193020	3.149330
Rh	-0.613530	-0.660420	0.740540
Cl	-2.264540	-4.729820	-1.727330
Cl	-3.961200	-4.684000	-4.381510
Cl	-7.977760	-2.074330	-1.790230
Cl	-6.856590	-3.487950	-4.375210
Cl	-0.608180	-8.035690	-1.788660
Cl	1.561330	-8.019760	-4.082250
Cl	4.506920	-4.382630	-1.286850
Cl	4.143130	-6.267600	-3.789980
Cl	-6.923520	1.096210	-0.402390
Cl	-8.033280	2.094690	-3.185920
Cl	-3.720090	5.387290	-3.721950
Cl	-6.384640	4.154620	-4.881990
Cl	5.018950	1.144110	-4.181600
Cl	5.270000	4.198300	-4.928090
Cl	4.142620	5.548330	0.234360
Cl	4.867680	6.395120	-2.727140
O	-2.568390	-1.064200	1.221210
O	-2.301830	-0.698940	3.432270
O	-6.754210	-1.463920	1.067440
O	-2.824610	-3.840670	1.232280
O	0.128370	-2.176380	3.433160
O	-0.125370	-2.576700	1.221400
O	2.674130	-3.635280	1.156700
O	-0.896790	-6.512610	0.960870
O	-0.918440	1.734770	2.745400
O	-1.174860	1.341120	0.538040
O	-2.141810	5.000010	-1.063600
O	-4.218380	1.541820	1.137680
O	1.387000	-0.257390	0.500870
O	1.675400	0.118630	2.718370
O	3.191240	2.809740	1.499820
O	4.527040	-0.400790	-1.469230
N	-4.706330	-2.477320	1.461520
N	0.822590	-5.015190	1.403540
N	-2.969480	3.334110	0.339960
N	3.913020	0.965000	0.298340
C	0.989820	-6.202490	-0.570880
C	0.795750	-7.030640	-1.665460
C	1.776700	-7.025790	-2.681490
C	2.927810	-6.223630	-2.560540
C	3.101780	-5.381210	-1.440530
C	2.111980	-5.377420	-0.471750
C	1.970310	-4.545130	0.766250
C	-2.955120	-1.105470	2.434330
C	-3.817810	-3.325890	0.772430
C	0.230830	-4.447710	2.609730
H	-0.805480	-4.808290	2.581930
C	0.095110	-2.932600	2.420570
C	-4.390000	-3.468440	-0.604570
C	-3.847380	-4.050780	-1.741180
C	-4.616930	-4.018040	-2.925800
C	-5.903390	-3.444240	-2.933380

C	-4.046570	2.432270	0.332700
C	-4.855940	2.779850	-0.875460
C	-6.064730	2.273200	-1.325480
C	-6.542570	2.720670	-2.577710
C	-5.808300	3.658150	-3.331460
C	-4.604110	4.203400	-2.828190
C	-4.157870	3.750020	-1.596330
C	-2.959740	4.132780	-0.799390
C	-6.421000	-2.828090	-1.771830
C	-5.637420	-2.838550	-0.627080
C	-1.970300	3.427960	1.408750
H	-1.175490	4.058710	1.001460
C	-1.313360	2.052790	1.601350
C	-5.836700	-2.167220	0.698060
C	-4.360670	-1.694560	2.652920
H	-5.051280	-0.842590	2.618080
C	0.144300	-5.982660	0.643600
C	4.313840	0.708520	-1.009750
C	4.442720	2.032770	-1.682510
C	4.775770	2.363670	-2.986630
C	4.880900	3.736680	-3.310110
C	4.680510	4.726820	-2.327320
C	4.321950	4.364500	-1.009960
C	4.176680	3.017430	-0.727310
C	3.707760	2.330580	0.510580
C	3.605920	-0.106420	1.258020
H	3.726170	-1.021190	0.669520
C	2.095260	-0.059190	1.546730
C	-4.605790	-2.423120	4.004250
C	-3.521480	-3.470690	4.341380
C	-4.675010	-1.372270	5.141490
C	-5.977090	-3.138800	3.936270
H	-2.541050	-2.988410	4.390510
H	-3.471090	-4.222840	3.547420
C	-3.840810	-4.145610	5.688070
H	-5.458020	-0.636880	4.906010
H	-3.728960	-0.830930	5.206930
C	-4.985930	-2.054480	6.485180
H	-5.959740	-3.894670	3.141770
H	-6.761290	-2.417020	3.675240
C	-6.297830	-3.820130	5.278930
H	-3.058390	-4.880500	5.913170
C	-3.883130	-3.082680	6.799870
C	-5.203680	-4.854780	5.598470
H	-5.015360	-1.290840	7.272660
C	-6.347120	-2.765610	6.398430
H	-7.270640	-4.321510	5.198550
H	-2.909330	-2.579940	6.874490
H	-4.077820	-3.558650	7.770230
H	-5.174380	-5.627010	4.818000
H	-5.430110	-5.361270	6.546180
H	-6.587530	-3.245120	7.356680
H	-7.141110	-2.034770	6.194150
C	0.856620	-4.965810	3.933680
C	2.224530	-4.334140	4.271670

C	-0.133670	-4.692170	5.092580
C	1.037620	-6.500750	3.825640
H	2.120920	-3.249270	4.349170
H	2.939060	-4.530000	3.465760
C	2.758630	-4.907100	5.597390
H	-1.098030	-5.160430	4.855110
H	-0.308070	-3.618320	5.189820
C	0.407770	-5.257830	6.415840
H	1.740510	-6.735240	3.016890
H	0.080690	-6.970660	3.564800
C	1.576920	-7.077700	5.146970
H	3.726450	-4.440300	5.821030
C	1.763810	-4.600000	6.731430
C	2.936600	-6.430310	5.467280
H	-0.308530	-5.034160	7.217000
C	0.587140	-6.779880	6.286970
H	1.699450	-8.162650	5.036660
H	1.639090	-3.513790	6.836810
H	2.152290	-4.976370	7.687240
H	3.659180	-6.657310	4.671700
H	3.341240	-6.846410	6.399610
H	0.960880	-7.200710	7.229950
H	-0.380750	-7.256440	6.080750
C	4.592170	-0.219150	2.447190
C	4.578140	0.961100	3.440960
C	4.253900	-1.527560	3.204690
C	6.022820	-0.354810	1.873660
H	3.572740	1.112960	3.835460
H	4.872610	1.883310	2.934940
C	5.576180	0.706830	4.584600
H	4.258680	-2.370630	2.505610
H	3.245490	-1.461080	3.620210
C	5.264270	-1.770900	4.336760
H	6.297330	0.554720	1.322490
H	6.069390	-1.187360	1.163770
C	7.037190	-0.591350	3.007260
H	5.527720	1.557290	5.275040
C	5.211190	-0.591890	5.324300
C	6.994470	0.585300	3.998910
H	4.996400	-2.701440	4.853260
C	6.679430	-1.893980	3.745480
H	8.038010	-0.675720	2.567070
H	4.204070	-0.508880	5.754890
H	5.907650	-0.761610	6.156550
H	7.270950	1.519080	3.490570
H	7.726520	0.426330	4.802110
H	7.409090	-2.086400	4.543490
H	6.723770	-2.744090	3.051710
C	-2.494450	4.160950	2.683520
C	-3.416140	3.307800	3.585660
C	-1.268660	4.641500	3.497150
C	-3.279410	5.421620	2.238800
H	-2.897390	2.399220	3.902590
H	-4.305470	2.993020	3.031580
C	-3.849590	4.126680	4.816860

H	-0.644400	5.268430	2.855310
H	-0.666940	3.787100	3.808510
C	-1.714120	5.445140	4.729170
H	-4.168030	5.129550	1.663870
H	-2.657090	6.041230	1.582300
C	-3.726310	6.240430	3.464960
H	-4.493680	3.498790	5.445690
C	-2.609490	4.563700	5.616280
C	-4.630840	5.370500	4.355990
H	-0.822390	5.748800	5.286110
C	-2.493420	6.688160	4.269360
H	-4.282600	7.119630	3.115720
H	-2.049390	3.681330	5.953980
H	-2.916060	5.116960	6.514230
H	-5.529380	5.066070	3.801580
H	-4.969080	5.949830	5.225290
H	-2.809540	7.283900	5.136150
H	-1.849460	7.325470	3.651960
C	-0.808970	-1.213620	-1.193830
C	-1.912670	-1.043980	-2.073690
C	-1.821900	-1.399870	-3.451090
C	-3.127620	-0.470110	-1.610780
C	-2.855370	-1.135840	-4.330290
H	-0.918920	-1.856120	-3.834370
C	-4.181950	-0.259000	-2.476980
H	-3.229980	-0.219180	-0.565020
C	-4.026580	-0.551420	-3.834900
H	-2.764720	-1.374620	-5.382790
H	-5.119270	0.136640	-2.119870
Br	-5.435890	-0.117600	-5.016540
C	0.339380	-2.000290	-1.715510
O	0.524890	-3.163360	-1.435120
O	1.133800	-1.263010	-2.521800
C	2.343500	-1.856200	-2.989300
H	3.113360	-1.092450	-2.920370
H	2.614070	-2.713900	-2.376620
C	2.230880	-2.291790	-4.452280
Cl	3.874180	-2.792030	-4.961760
Cl	1.663060	-0.921520	-5.462700
Cl	1.089800	-3.675080	-4.635420
F	6.546540	-1.454870	-3.683540
F	6.927710	-3.567640	-3.339900
F	8.572260	-2.243060	-3.863440
F	7.312080	0.294010	-1.649040
F	8.540150	-0.571610	-0.076790
F	9.323020	-0.415740	-2.099960
O	6.489410	-2.298470	-0.934670
H	5.796620	-1.655530	-1.197470
C	7.654130	-2.071480	-1.667570
H	8.404050	-2.791570	-1.329010
C	7.427370	-2.332900	-3.164090
C	8.219290	-0.671640	-1.379470
F	1.017010	2.923980	-0.876010
F	1.811360	3.468000	-2.836550
F	1.369430	1.392930	-2.370500

F	-0.397260	1.265120	-4.434320
F	-1.938330	2.792650	-4.535520
F	0.139440	3.313620	-4.941420
O	-1.428900	2.129650	-2.002450
H	-1.174280	1.948870	-1.066520
C	-0.484950	2.894520	-2.690260
H	-0.663800	3.972920	-2.578560
C	0.946620	2.656040	-2.199970
C	-0.663660	2.557080	-4.172950
F	-0.186180	7.123970	-2.218270
F	1.698780	6.048010	-2.119250
F	1.668020	8.202060	-1.804120
F	-1.341050	7.791270	0.243750
F	-0.001360	7.679640	1.956220
F	0.393190	9.109080	0.362690
O	0.239710	5.531700	0.107360
H	-0.634680	5.491490	-0.339620
C	0.797790	6.794810	-0.077250
H	1.780010	6.803760	0.394860
C	0.998050	7.062460	-1.572980
C	-0.047190	7.866670	0.620500
F	1.718280	5.269830	2.470490
F	3.107850	6.860670	2.966440
F	1.375920	6.511650	4.235590
F	1.742670	3.018900	4.042190
F	3.116250	3.026760	5.727200
F	1.354250	4.305930	5.758880
O	4.196830	4.396560	3.547100
H	3.805780	3.890350	2.804710
C	3.166720	4.922180	4.322420
H	3.616080	5.492110	5.140740
C	2.319450	5.901690	3.497020
C	2.325050	3.805100	4.967360
